# Antimicrobial activity of new glycoside derivatives of chloroflavones obtained by fungal biotransformation

**DOI:** 10.1038/s41598-025-11079-7

**Published:** 2025-07-16

**Authors:** Agnieszka Krawczyk-Łebek, Barbara Żarowska, Tomasz Janeczko, Edyta Kostrzewa-Susłow

**Affiliations:** Faculty of Biotechnology and Food Science, Wrocław University of Environmental and Life Sciences, Wrocław, Poland

**Keywords:** Biocatalysis, Antimicrobials

## Abstract

Chlorinated flavonoids represent a unique subclass of flavonoids with chlorine substituents. The incorporation of chlorine atoms and glucosyl moieties may influence their bioavailability, bioactivity, and pharmacological potential. In this study, 2′-chloroflavone, 3′-chloroflavone, 4′-chloroflavone, and 6-chloroflavone were synthesized and biotransformed using entomopathogenic fungi cultures (*Isaria fumosorosea* KCH J2 and *Beauveria bassiana* KCH J1.5) to obtain novel glycosylated derivatives. Pharmacokinetic properties and drug-likeness were predicted using cheminformatics tools. Antimicrobial activity was evaluated against several microbial strains. *Enterococcus faecalis* ATCC 19433 showed complete growth inhibition with 4′-chloroflavone and 6-chloroflavone, while 2′-chloroflavone and 3′-chloroflavone significantly inhibited its growth. Flavonoid glycosides and flavone demonstrated lower efficacy. *Staphylococcus aureus* ATCC 29213 was completely or strongly inhibited by all tested compounds. *Lactobacillus acidophilus* ATCC 4356 was moderately inhibited by flavonoid aglycones and slightly inhibited by glycosides. *Escherichia coli* ATCC 25922 was most effectively inhibited by 4′-chloroflavone and 6-chloroflavone, followed by 2′-chloroflavone and 3′-chloroflavone, with flavone and glycosides showing lower activity. *Candida albicans* ATCC 1023 exhibited high sensitivity to all compounds. Overall, chlorinated flavones demonstrated greater antimicrobial activity than non-chlorinated counterparts, with aglycones being more effective than glycosylated derivatives. The position of the chlorine atom significantly influences antimicrobial activity.

## Introduction

Flavonoids with a chlorine atom represent a unique subclass of flavonoids characterized by the presence of chlorine substituents. While relatively rare in nature compared to their non-halogenated counterparts, these compounds have garnered significant attention due to their diverse biological activities and potential therapeutic applications^1–5^. Chlorinated flavonoids have been primarily isolated from microorganisms. Notable examples include the chlorflavonin (3′-chloro-2′,5-dihydroxy-3,7,8-trimethoxy flavone)^6–8^ and structurally related aspergivone A (3′-chloro-2′-hydroxy-3,5,7,8-tetramethoxy flavone)^9^, both isolated from *Aspergillus candidus* fungus. Chlorflavonin demonstrated high activity against fungal species, particularly *Aspergillus fumigatus*, with a minimum inhibitory concentration below 1 µg/ml^6^. Moreover it targets acetohydroxyacid synthase catalytic subunit IlvB1 for synergistic killing of Mycobacterium tuberculosis with MIC_90_ 1.56 μM while exhibiting no cytotoxicity towards the human cell lines MRC-5 and THP-1 up to concentrations of 100 μM^10^. On the other hand, aspergivone A and its non-chlorinated counterpart aspergivone B didn’t show obvious activity at the concentration of 10 μM and 50, respectively^9^.

Chlorinated flavonoids in human diet are not presence but research indicates that during specific pathological states in vivo, particularly inflammatory conditions, flavonoids can undergo conversion to their chlorinated derivatives as a result of their reaction with reactive chlorinating and oxidizing species such as hypochlorous acid. These modified compounds demonstrate increased antioxidant capacity, potentially contributing to cardiovascular protective effects^2,11^. Flavonols exposed to equimolar or moderately excessive concentrations of HOCl demonstrated enhanced antioxidant properties compared to their precursor compounds, while treatment with highly excessive molar ratios of HOCl resulted in diminished antioxidant activity^12^. Another study showed that dietary flavonoids protect hemoglobin from hypochlorous acid (HOCl) damage. Using spectrophotometry, researchers found that flavonoids inhibit HOCl-induced heme damage either by competing with hemoglobin for HOCl or by forming protective hemoglobin-flavonoid complexes^13^. Therefore attempts were made to obtain synthetic flavonoids and investigate their bioactivity. Synthesized chlorinated flavonoids showed anti-inflammatory activity with greater efficiency in modulating neutrophils’ oxidative burst compared to their non-chlorinated counterparts. Some flavonoids induced neutrophil apoptosis through caspase-3 activation. Specifically, 8-chloro-3′,4′,5,7-tetrahydroxyflavone showed promise as an anti-inflammatory agent by suppressing inflammation mechanisms^3,14^. Proença team conducted a comprehensive evaluation of novel chlorinated flavonoids and their parent compounds for their anti-inflammatory potential. Using human whole blood as an in vitro model, they assessed flavonoids effects on key inflammatory processes, including COX-1 and COX-2 activity, cytokine and chemokine production, and reactive species generation. While luteolin showed the highest activity, chlorinated flavonoids exhibited a distinctive anti-inflammatory profile. Notably, 6-chloro-3′,4′,5,7-tetrahydroxyflavone demonstrated significant potential in modulating inflammatory mediators, highlighting the therapeutic relevance of chlorinated flavonoids^4^. In this regard chlorinated flavonoids represent a compelling research target due to their antimicrobial and anti-inflammatory properties, particularly in the framework of studies on structure–activity relationships.

The biological activity and bioavailability of flavonoids can also be modulated through the introduction of a glucose moiety. Such alteration positively affects water solubility of flavonoids and consequently their bioavailability^15–17^. Moreover, glucosides are likely the only glycosides that can be absorbed in the small intestine, allowing them to achieve higher plasma concentrations compared to compounds primarily absorbed in the colon^18,19^. Consequently, glycosylation can be used as a strategy to enhance the bioavailability, and pharmacological efficacy of flavonoids. A promising approach for the glycosylation of flavonoids involves the use of microbial enzymes, particularly through whole-cell biotransformation with filamentous fungi as biocatalysts^20,21^. Xie and colleagues utilized genome mining and heterologous expression techniques to identify functional modules of glycosyltransferase-methyltransferase (GT-MT) in these fungi. These modules demonstrated both substrate promiscuity and regiospecificity, enabling them to successfully methylglucosylate flavonoids. An effective strategy for the glycosylation of flavonoids can be the use of microbial enzymes, among others whole-cell biotransformation using filamentous fungi as biocatalysts possessing functional modules of glycosyltransferase-methyltransferase with substrate promiscuity and regiospecificity, allowing them to methylglucosylate flavonoids^21–26^. *Beauveria bassiana* is an entomopathogenic filamentous fungus that affects a wide variety of arthropods and has been employed for many years as a biological control agent against various arthropod pests. The pathogenicity and virulence of *B. bassiana* isolates are linked to the production of toxic metabolites and primarily extracellular cuticle-degrading enzymes, such as chitinases, lipases, and proteases^27,28^. The extensive enzymatic systems of *B. bassiana* make it possible to biotransform a wide range of exogenic substrates, including cyclic and aliphatic ketones, flavonoids, and steroids^29^. Similarly, *Isaria fumosorosea* is used as a biological control agent and an alternative to chemical pesticides^30^. Both species were previously used as a biocatalyst in studies focused on flavonoid biotransformations^22,25,29,31^.

Considering the above, the primary objective of this study was to synthesize four flavones with a chlorine atom positioned at different sites within the flavonoid core and subsequently subject them to biotransformation using entomopathogenic filamentous fungi to obtain their glycosylated derivatives.

Microbial transformation was carried out using two fungal strains, *I. fumosorosea* KCH J2 and *B. bassiana* KCH J1.5, which were specifically selected due to their extensive enzymatic systems, including diverse glycosyltransferases, methyltransferases, and oxidative enzymes, enabling them to biotransform a wide range of exogenous substrates, particularly flavonoids. It is well established that entomopathogenic fungi such as *Beauveria bassiana* (syn. *Cordyceps bassiana*) and *Isaria fumosorosea* (syn. *Cordyceps fumosorosea*) can produce various secondary metabolites, including beauvericin, bassianolide, oosporein, tenellin, isarolide, and fumosorinone, though these fungi are considered environmentally friendly and do not show harmful effects linked to chemical pesticides^32–35^. Previous studies confirmed their effectiveness in flavonoid modification; thus, these fungi were hypothesized to effectively glycosylate chloroflavones, potentially providing unique derivatives with novel biological properties^20,29,31,36^. The biotransformations performed led to the formation of three new glycosylated flavonoid derivatives. Additionally, we employed computer-aided simulations to assess and compare the physicochemical properties and pharmacokinetics of all obtained compounds. The flavonoid aglycones, biotransformation products, and for comparison a non-chlorinated flavone were evaluated for their antimicrobial activity, enabling us to examine the impact of chlorine substitution on antimicrobial properties.

## Results and discussion

**Fig. 1:**
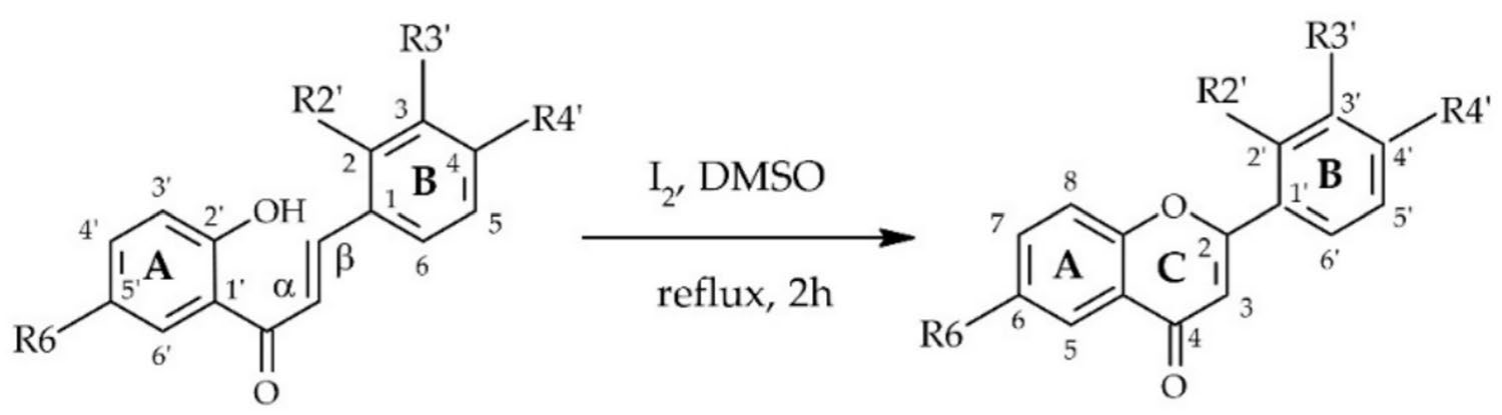
Chemical synthesis of flavones with a chlorine atom by the cyclisation of 2′-hydroxychalcones with a chlorine atom.

**Fig. 2:**
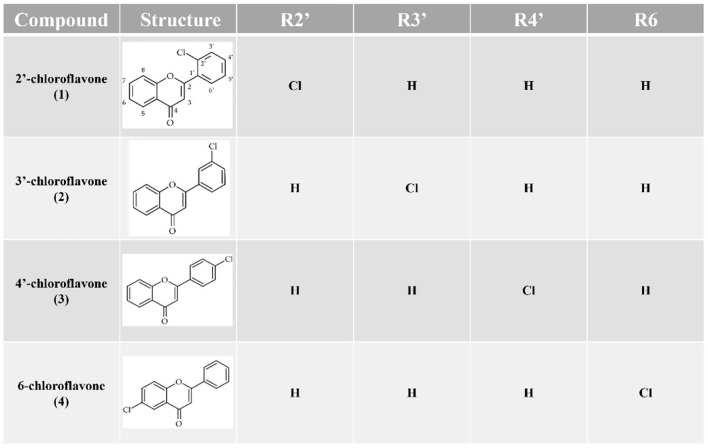
Chemical structures of the obtained flavones with a chlorine atom.

The first stage of study concerned synthesis of four flavones with a chlorine atom, i.e. 2′-chloroflavone, 3′-chloroflavone, 4′-chloroflavone, and 6-chloroflavone. Chloroflavones were obtained by cyclization of the corresponded 2′-hydroxychalcones with a chlorine atom^36,37^ in the presence of iodine excess (Figs. 1 and 2).

The structures of all synthesis products (**1**–**4**) were confirmed based on NMR spectroscopy (Tables 1, 2, 3, 4).

**Table 1:** ^1^H-NMR chemical shifts δ (ppm) and coupling constants *J* (Hz) of 2′-chloroflavone (**1**), 3′-chloroflavone (**2**) and products of their biotransformations **1a** and **2a** in Acetone-d6, 600 MHz (Supplementary Information: Figs. S3–S4, S18–S20, S37–S38, S52–S54).

Proton	Compound			
	**1**	**1a**	**2**	**2a**
H-3	6.56 (s)	6.51 (s)	6.93 (s)	6.86 (s)
H-5	8.16 (dd) *J* = 7.9 *J* = 1.6	8.16 (dd) *J* = 8.0, *J* = 1.7	8.12 (m)	8.11 (dd) *J* = 7.9, *J* = 1.6
H-6	7.53 (m)	7.53 (m)	7.50 (ddd) *J* = 8.0, *J* = 7.1, *J* = 1.1	7.49 (m)
H-7	7.84 (m)	7.84 (m)	7.83 (ddd) *J* = 8.6, *J* = 7.0, *J* = 1.7	7.82 (dd) *J* = 8.5, *J* = 6.9, *J* = 1.7
H-8	7.64 (m)	7.63 (d) *J* = 8.5	7.77 (m)	7.77 (m)
H-2′	–	–	8.13 (td) *J* = 1.8, *J* = 0.7	8.17 (d) *J* = 2.3
H-3′	7.64 (m)	–	–	–
H-4′	7.64 (m)	7.53 (m)	7.63 (m)	-
H-5′	7.57 (td) *J* = 7.5, *J* = 1.3	7.47 (m)	7.63 (m)	7.49 (m)
H-6′	7.84 (m)	7.42 (m)	8.06 (m)	8.02 (dd) *J* = 8.8, *J* = 2.3
H-1″	–	5.16 (d) *J* = 7.8	–	5.23 (d) *J* = 7.8
H-2″	–	3.60 (m)	–	3.61 (dt) *J* = 4.6, *J* = 2.8
H-3″	–	3.70 (m)	–	3.70 (m)
H-4″	–	3.26 (m)	–	3.26 (dd) *J* = 9.5, *J* = 9.0
H-5″	–	3.55 (m)	–	3.56 (m)
H-6″	–	3.86 (m) 3.70 (m)	–	3.86 (m)
4″-OCH_3_	–	3.58 (s)	–	3.58 (s)
2″-OH	–	4.73 (m)	–	4.75 (d) *J* = 4.5
3″-OH	–	4.51 (m)	–	4.52 (d) *J* = 4.0
6″-OH	–	3.86 (m)	–	3.70 (m)

**Table 2:** ^13^C-NMR chemical shifts δ (ppm) and coupling constants *J* (Hz) of of 2′-chloroflavone (**1**), 3′-chloroflavone (2) and products of their biotransformations **1a** and **2a** in Acetone-d6, 151 MHz (Supplementary Information: Figs. S5–S7, S21–23, S39–S41, S55–S57).

Carbon	Compound			
	**1**	**1a**	**2**	**2a**
C-2	163.5	163.7	162.3	162.4
C-3	113.4	113.4	108.8	107.5
C-4	177.6	177.6	177.8	177.8
C-4a	124.7	124.7	124.8	124.8
C-5	126.1	126.1	126.0	125.9
C-6	126.4	126.5	126.3	126.2
C-8	119.3	119.3	119.4	119.3
C-7	135.1	135.1	135.0	134.8
C-8a	157.5	157.4	157.1	157.0
C-1′	133.0	134.4	134.9	127.1
C-2′	133.0	122.5	127.0	128.9
C-3′	131.4	154.7	135.6	124.2
C-4′	133.2	119.3	132.2	156.4
C-5′	128.5	128.9	131.7	117.2
C-6′	132.0	124.7	125.8	127.3
C-1″	–	101.7	–	101.2
C-2″	–	74.8	–	74.7
C-3″	–	78.0	–	78.0
C-4″	–	79.9	–	79.9
C-5″	–	77.2	–	77.3
C-6″	–	62.0	–	61.9
4″-OCH_3_	–	60.6	–	60.6

**Table 3:** ^1^H-NMR chemical shifts δ (ppm) and coupling constants *J* (Hz) of 4’-chloroflavone (**3**), 6-chloroflavone (**4**) and its biotransformation product **4a** in Acetone-d6, 600 MHz (Supplementary Information: Figs. S71–S72, S86–S87, S101–S103).

Proton	Compound		
	**3**	**4**	**4a**
H-3	6.89 (s)	6.92 (s)	6.83 (s)
H-5	8.12 (m)	8.05 (m)	8.03 (t) *J* = 1.4
H-6	7.50 (ddd) *J* = 8.1, *J* = 7.1, *J* = 1.1	–	–
H-7	7.83 (ddd) *J* = 8.7, *J* = 7.1, *J* = 1.7	7.83 (m)	7.80 (m)
H-8	7.74 (dd) *J* = 8.5, *J* = 1.1,	7.83 (m)	7.80 (m)
H-2’	8.12 (m)	8.12 (m)	8.06 (m)
H-3′	7.63 (m)	7.62 (m)	7.24 (m)
H-4′	-	7.62 (m)	-
H-5′	7.63 (m)	7.62 (m)	7.24 (m)
H-6′	8.12 (m)	8.12 (m)	8.06 (m)
H-1″	–	–	5.10 (d) *J* = 7.8
H-2″	–	–	3.52 (m)
H-3″	–	–	3.68 (m)
H-4″	–	–	3.23 (t) *J* = 9.3
H-5″	–	–	3.52 (m)
H-6″	–	–	3.86 (m) 3.86 (m)
4″-OCH_3_	–	–	3.57 (s)
2″-OH	–	–	4.77 (s)
3″-OH	–	–	4.50 (s)
6″-OH	–	–	3.68 (m)

**Table 4:** ^13^C-NMR chemical shifts δ (ppm) and coupling constants *J* (Hz) of 4’-chloroflavone (**3**), 6-chloroflavone (**4**) and its biotransformation product **4a** in Acetone-d6, 151 MHz (Supplementary Information: Figs. S73–S75, S90–38, S104–S106).

Carbon	Compound		
	**3**	**4**	**4a**
C-2	162.7	164.3	164.1
C-3	108.2	107.9	106.7
C-4	177.9	176.8	176.7
C-4a	124.8	126.0	125.9
C-5	126.0	125.1	125.1
C-6	126.3	131.4	131.2
C-8	119.3	121.7	121.6
C-7	135.0	134.8	134.6
C-8a	157.1	155.7	155.6
C-1′	131.6	132.5	125.8
C-2′	128.9	127.3	129.0
C-3′	130.1	130.0	117.7
C-4′	138.0	132.7	161.6
C-5′	130.1	130.0	117.7
C-6′	128.9	127.3	129.0
C-1″	–	–	101.1
C-2″	–	–	74.8
C-3″	–	–	77.9
C-4″	–	–	80.0
C-5″	–	–	77.2
C-6″	–	–	62.0
4″-OCH_3_	–	–	60.6

Entomopathogenic filamentous fungi strains *Isaria fumosorosea* KCH J2 and *Beauveria bassiana* KCH J1.5 were employed as biocatalysts to transform 2′-chloroflavone (**1**), 3′-chloroflavone (**2**), 4′-chloroflavone (**3**), and 6-chloroflavone (**4**) through microbial processes. This resulted in the synthesis of three novel glycosylated flavonoids, derivatives of compounds **1**, **2**, and **4**. However, compound **3** was not successfully transformed by either of the two strains. The biotransformation products were extracted from the reaction mixture and subsequently purified using preparative thin-layer chromatography (TLC). Transformation yields were calculated based on the quantities of isolated products. Structural elucidation of these compounds was accomplished through NMR spectroscopy and corroborated by LC–MS analysis. To assess the biological activity of these new compounds, computational methods leveraging structure–activity relationships were utilized. Furthermore, antimicrobial assays were conducted on the biotransformation substrates (**1**–**4**), their biotransformation products (**1a**, **2a**, and **4a**), and a non-chlorinated flavone. These tests involved monitoring the growth of selected microbial strains using automated measurement turbidimetric techniques.

### Biotransformation of 2′-chloroflavone (1) in culture of *I. fumosorosea* KCH J2

**Fig. 3:**
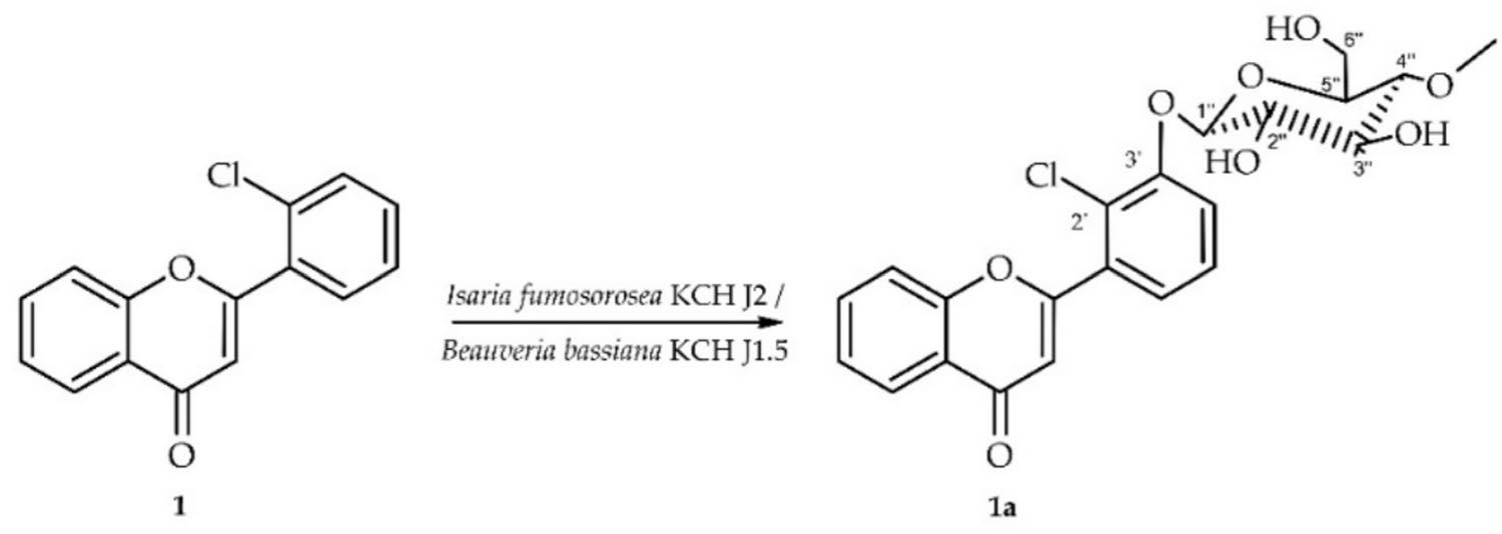
Biotransformation of 2′-chloroflavone (**1**) in *I. fumosorosea* KCH J2 or *B. bassiana* KCH J1.5 culture.

2′-Chloroflavone (**1**) was biotransformed in culture of *I. fumosorosea* KCH J2 into 2′-chloroflavone 3′-*O*-*β*-D-(4″-*O*-methyl)-glucopyranoside (**1a**) yielding 10.8% (9.4 mg) (Fig. 3).

**Fig. 4:**
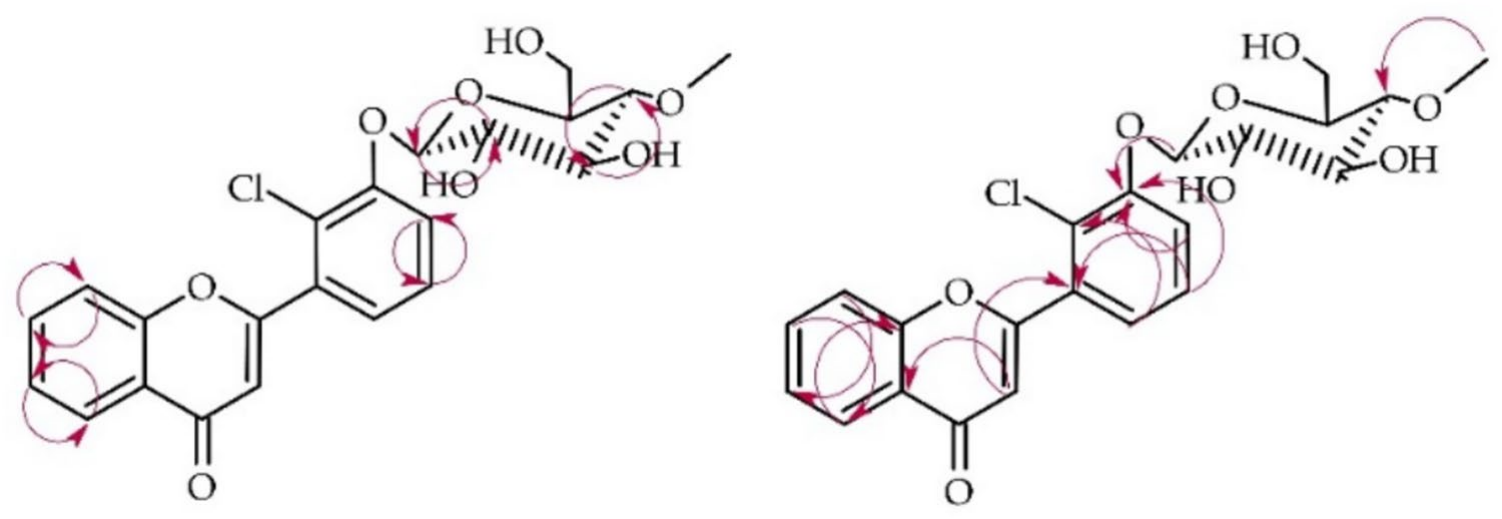
Key COSY (on the left) and HMBC (on the right) correlations for the structure elucidation of product **1a**.

The structure of product **1a** was determined using NMR spectroscopy (see Tables 1 and 2). Key COSY and HMBC correlations are illustrated in Fig. 4. The molecular mass of the product was confirmed through LC–MS analysis (detailed in "Materials and methods". Materials and Methods and Supplementary Information: Fig. S16).

The biotransformation product **1a** was confirmed to contain a glucose moiety based on NMR spectroscopy data. The ^13^C-NMR spectrum contained five characteristic carbon signals between δ = 79.9 ppm and δ = 62.0 ppm (Supplementary Information: Figure S23). Complementary evidence was found in the ^1^H-NMR spectrum, which showed corresponding proton signals ranging from δ = 3.86 ppm to δ = 3.26 ppm (Supplementary Information: Fig. S20). The *β*-configuration of the glucose was established by a one-proton doublet at δ = 5.16 ppm in the ^1^H NMR spectrum, belonging to the anomeric carbon atom. This signal had a coupling constant of *J* = 7.8 Hz, characteristic of glucose *β*-configuration (Supplementary Information: Fig. S20). Additionally, *O*-methylation at the C-4″ position of the glucose was indicated by a three-proton singlet at δ = 3.58 ppm in the ^1^H-NMR spectrum, with a corresponding signal at δ = 60.6 ppm in the ^13^C-NMR spectrum (Supplementary Information: Figs. S20 and S23). The HMBC experiment provided further evidence for the glucose unit’s structure. A correlation was observed between the –O–CH_3_ moiety and the C-4″ signal (δ = 79.9 ppm). This correlation confirms the position of the methoxy group substitution on the glucose unit (Supplementary Information: Fig. S33). The substitution position of the 4″-*O*-methylglucosyl moiety was determined mainly using two key spectroscopic methods. A comparison of ^1^H NMR spectra of the biotransformation substrate (**1**) and product (**1a**) revealed protons of ring B shift differences (H-4′ δ = 7.64 → 7.53 ppm, H-5′ δ = 7.57 → 7.47 ppm, H-6′ δ = 7.84 → 7.42 ppm) and H-3′ signal disappearance indicating substitution at this position. Additionally, HMBC data analysis provided further evidence because the signals from H-1″ at δ = 5.16 ppm, H-4′ at δ = 7.53 ppm, and H-5′ at δ = 7.47 ppm correlated with the shifted signal from C-3′ at δ = 154.7 ppm (Supplementary Information: Fig. S32).

### Biotransformation of 2′-chloroflavone (1) in culture of *B. bassiana* KCH J1.5

2′-Chloroflavone (**1**) was also biotransformed in culture of *B. bassiana* KCH J1.5 with the same biotransformation product, i.e., 2′-chloroflavone 3′-*O*-*β*-D-(4″-*O*-methyl)-glucopyranoside (**1a**) yielding 15.2% (13.2 mg) (Fig. 3).

### Biotransformation of 3′-chloroflavone (2) in culture of *I. fumosorosea* KCH J2

**Fig. 5:**
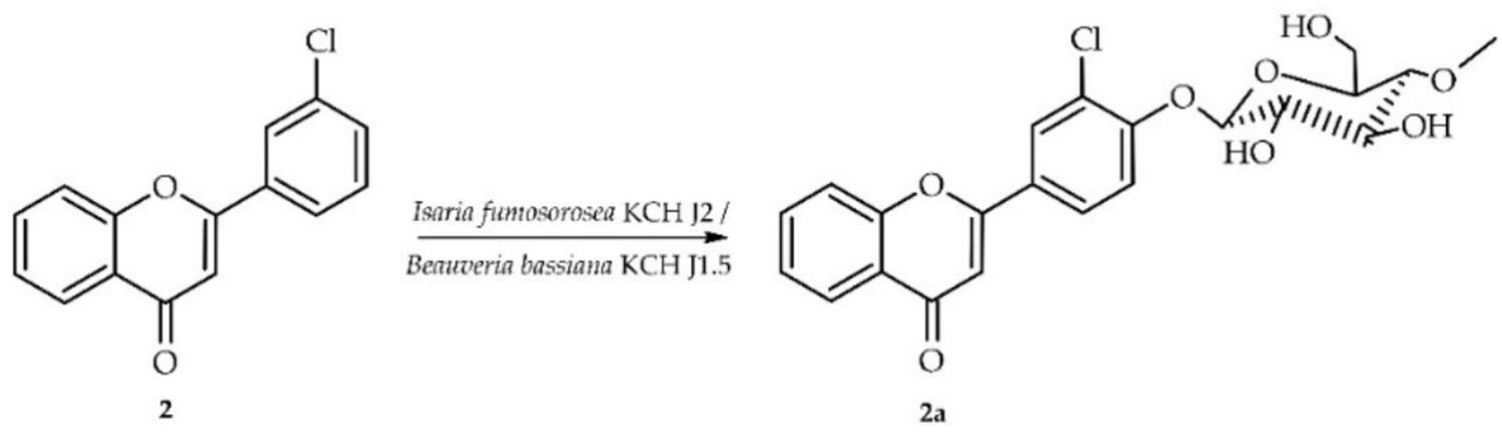
Biotransformation of 3′-chloroflavone (**2**) in *I. fumosorosea* KCH J2 or *B. bassiana* KCH J1.5 culture.

Biotransformation of 3′-chloroflavone (**2**) in culture of *I. fumosorosea* KCH J2 resulted in the formation of one product, i.e. 3′-chloroflavone 4′-*O*-*β*-D-(4″-*O*-methyl)-glucopyranoside (**2a**) yielding 40.5% (35.4 mg) (Fig. 5).

**Fig. 6:**
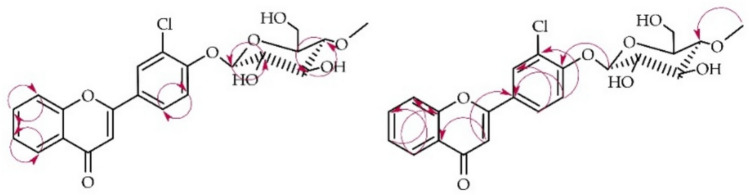
Key COSY (on the left) and HMBC (on the right) correlations for the structure elucidation of product **2a**.

The structure of product **2a** was also elucidated based on NMR spectroscopy (Tables 1 and 2, Fig. 6 below with key COSY and HMBC correlations). Its molecular mass was confirmed using LC–MS (detailed in "Materials and methods". Materials and Methods and Supplementary Information: Fig. S50).

This biotransformation product was also 4″-O-methylglucosylated because of characteristic signals observed in the ^1^H NMR and ^13^C NMR spectra, similar to the above mentioned for product **1a** (Supplementary Information: Figs. S54 and S57). The chemical shifts of signals from rings A and B in the ^1^H NMR reviled that in this case substitution also occurred in ring B because signals from ring A remained intact. On the other hand, shift of the H-2′ signal in the ^1^H NMR spectrum (from δ = 8.13 ppm in **2** to δ = 8.17 ppm in **2a**) and the change in its multiplicity (from triplet of doublets in **2** to doublet in **2a**) indicated substitution in its neighborhood at H-4′. It was also confirmed by shift of signal from two protons at δ = 7.63 ppm (H-4′ and H-5′) in compound **2** to δ = 7.49 ppm (only one proton H-5′) in **1a** (Supplementary Information: Fig. S53). Moreover, proton at δ = 7.49 ppm in HMBC experiment correlated with signals from C-3′ (δ = 124.2 ppm) and C-5′ (δ = 117.2 ppm) confirming that it belongs to H-5′ and substitution occurred at C-4′ (Supplementary Information: Fig. S65). Furthermore, the signal from proton of anomeric carbon atom H-1″ at δ = 5.23 ppm correlated with the signal of C-4′ at δ = 156.4 ppm (Supplementary Information: Fig. S66).

### Biotransformation of 3′-chloroflavone (2) in culture of *B. bassiana* KCH J1.5

Biotransformation of the same substrate 3′-chloroflavone (**2**) in culture of another fungal strain *B. bassiana* KCH J1.5 resulted in the formation of the same product **2a** yielding 48.6% (42.5 mg) (Fig. 5).

### Biotransformation of 4′-chloroflavone (3) in cultures of *I. fumosorosea* KCH J2 and *B. bassiana* KCH J1.5

Biotransformation of 4′-chloroflavone (**3**) in both used fungi strains, i.e. *I. fumosorosea* KCH J2 and *B. bassiana* KCH J1.5 was not successful. We did not observe substrate conversion. It may suggest that substitution with a chlorine atom at the position C-4′ of the flavone ring B prevent action of enzymes resposible for its *O*-glucosylation.

### Biotransformation of 6-chloroflavone (4) in cultures of *I. fumosorosea* KCH J2

**Fig. 7:**
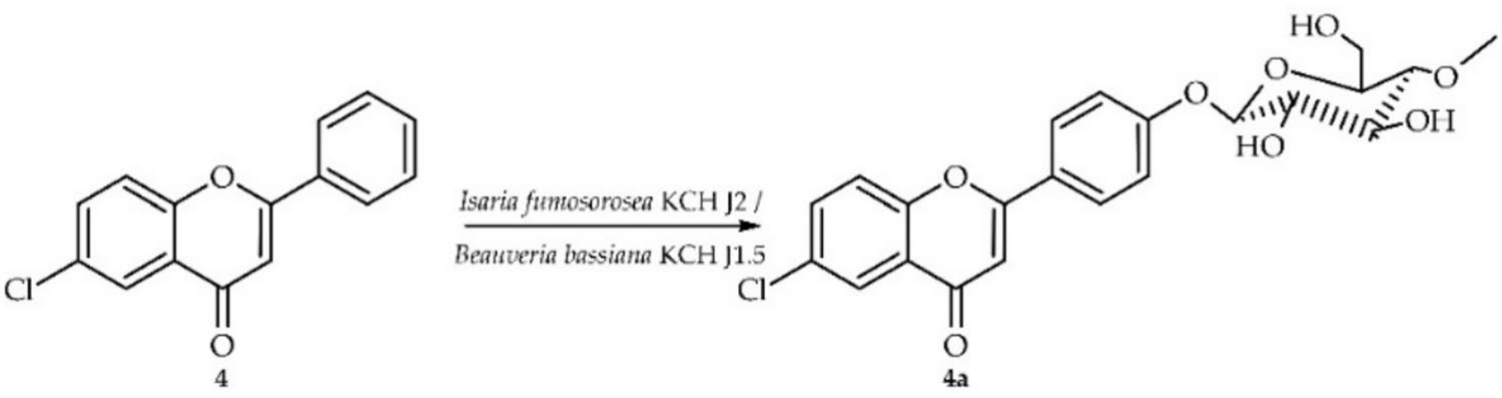
Biotransformation of 6-chloroflavone (**4**) in *I. fumosorosea* KCH J2 or *B. bassiana* KCH J1.5 culture.

6-Chloroflavone (**4**) was biotransformed in culture of *I. fumosorosea* KCH J2 into 6-chloroflavone 4′-*O*-*β*-D-(4″-*O*-methyl)-glucopyranoside (**4a**) yielding 62% (54 mg) (Fig. 7).

**Fig. 8:**
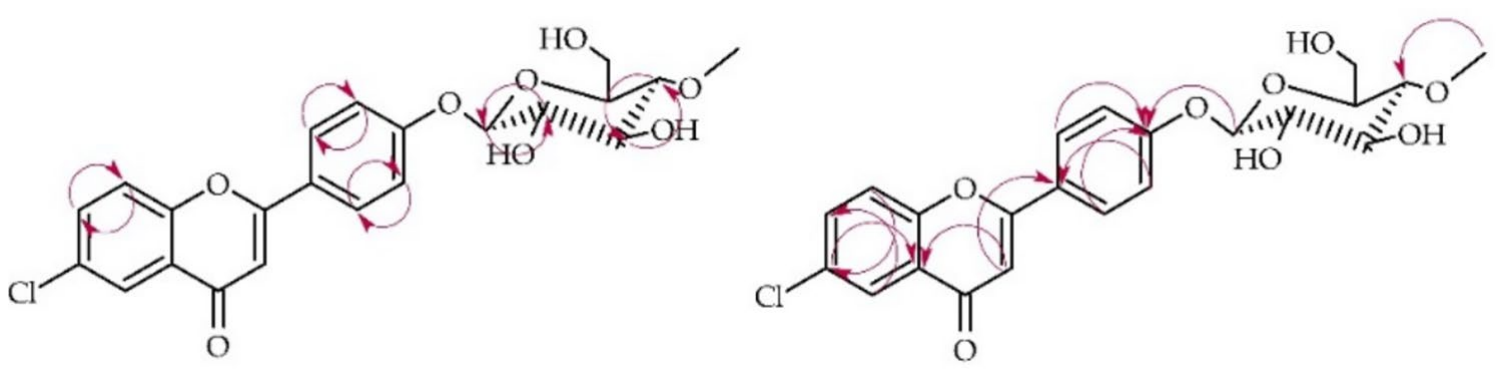
Key COSY (on the left) and HMBC (on the right) correlations for the structure elucidation of product **4a**.

Compound **4a** underwent structural analysis using NMR spectroscopy, with key data presented in Tables 1 and 2. Figure 8 below illustrates the crucial COSY and HMBC correlations observed. LC–MS analysis corroborated the molecular mass of **4a**, as detailed in "Materials and methods". Materials and Methods and Fig. S99 of the Supplementary Information.

In this case, biotransformation product **4a** was also obtained as a result of the attachment of a 4″-O-methylglucosyl moiety to the flavonoid core, which was observed in the ^1^H NMR and ^13^C NMR spectra as the appearance of characteristic signals from this moiety (Supplementary Information: Figs. S103 and S106). In the ^1^H NMR spectrum, protons from ring A remained unshifted, but signals from ring B changed—multiplets from protons at C-2′ and C-6′ (δ = 8.06 ppm) and from protons at C-3′ and C-5′ (δ = 7.24 ppm) can be observed, which is characteristic of an AA’BB’ coupling system, indicating the presence of 4′-substitution in the 6-chloroflavone ring B (Supplementary Information: Fig. S103). This was confirmed in the HMBC experiments by the presence of characteristic correlations, notably the correlations between the protons at C-2′ and C-6′ (δ = 8.06 ppm) and the protons at C-3′ and C-5′ (δ = 7.24 ppm) with the shifted signal of C-4′ (δ = 161.6 ppm). Additionally, the signal from the proton at C-1″ (δ = 5.06 ppm) also correlated with the signal of C-4′ (δ = 161.6 ppm) (Supplementary Information: Fig. S115).

### Biotransformation of 6-chloroflavone (4) in cultures of *B. bassiana* KCH J1.5

6-Chloroflavone (**4**) was biotransformed in the second strain B. bassiana KCH J1.5 into the same prodcut as in the case of *I. fumosorosea* KCH J2—**4a** yielding 12.6% (11.0 mg) (Fig. 7).

Biotransformation substrates, i.e. 2′-chloroflavone (**1**), 3′-chloroflavone (**2**), and 6-chloroflavone (**4**) were successfully biotransformed in cultures of fungi strains *I. fumosorosea* KCH J2 and *B. bassiana* KCH J1.5 into glycosylated flavones. Only 4′-chloroflavone (**3**) was not biotransformed, probably due to steric hindrance preventing enzymatic O-glucosylation by substitution at the C-4′ position of the flavone. The results reveal that a slight change in the position of the substituent can result in a significant improvement in process yield or, conversely, no product formation. The main products were obtained by attaching a 4″-*O*-methylglucosyl moiety to the B ring in all three chloroflavones. The regioselectivity of biotransformations was consistent across both fungal strains, indicating no difference in the action of glycosyltransferase-methyltransferase functional modules of the two strains. The 2′-chloroflavone was glycosylated at the ortho position, while the two other biotransformation substrates with chlorine atoms at the B ring (C-3′) and A ring (C-6) were glycosylated at the same position on the B ring—C-4′.

Prior research on flavone glycosylation by filamentous fungi has demonstrated diverse glycosylation patterns. Specifically, studies on the strains: *Beauveria bassiana* AM 278, *Absidia coerulea* AM 93 and *Absidia glauca* AM 177 showed that their enzymatic systems catalyzed the methylglucose and glucose group attachment reactions to the flavonoid molecules (flavones: chrysin, apigenin, luteolin, diosmetin and flavanones: pinocembrin, naringenin, eriodictyol, hesperetin) at positions C7 and C3′^38^. The entomopathogenic filamentous fungus *Isaria fumosorosea* ACCC 37814 biotransformed flavonoids such as naringenin, luteolin, diosmetin, and formononetin into their 4′-O-(methyl)glucopyranosides. Glycosylation occurred at sites C-7 on ring A and at C-3′ and C-4′ on ring B^21^. Biotransformation of 6-methyl-8-nitroflavone in the culture of *B. bassiana* KCH J1.5 also resulted in the attachment of a 4″-*O*-methylglucosyl moiety at C-4′^24^. Similarly, biotransformation of 8-bromo-6-chloroflavone in the cultures of *Isaria farinosa* J2.6 also gave a derivative product with the attached a 4″-*O*-methylglucosyl moiety^24^. Other halogenated compounds, i.e. 6-chloro-8-nitroflavone and 6-bromo-8-nitroflavone were also glycosylated at C-4′ in the *B. bassiana* KCH J1.5 cultures^39^.

In a distinct approach, the UDP-glycosyltransferase (YjiC) from *Bacillus licheniformis* DSM-13 exhibited remarkable versatility by transforming phloretin into five different glucoside derivatives, with glucose moieties positioned at various locations including C-2′, C-4′, C-4, C-6′, and multiple combination sites^40^. Since flavonoids naturally occur in plants, numerous plant-derived glycosyltransferases have been identified along with their substrate specificity toward particular flavonoid aglycones^41^. However, the presented studies demonstrate the biotransformation potential of entomopathogenic filamentous fungi *I. fumosorosea* KCH J2 and *B. bassiana* KCH J1.5 strains on flavonoids with diverse substituents, highlighting the significance of exploring novel compound modifications.

### Pharmacokinetics and drug-likeness prediction of compounds 1, 1a, 2, 2a, 3, 4, 4a

The SwissADME website (http://www.swissadme.ch), developed and maintained by the Swiss Institute of Bioinformatics (SIB) Molecular Modeling Group^42^, was utilized to predict the pharmacokinetics, aqueous solubility, and drug-likeness of compounds **1**, **1a**, **2**, **2a**, **3**, **4**, **4a**, as well as flavone (**5**) without a chlorine atom for comparison. These predictions were accessed on July 28th, and the corresponding prediction screens can be found in the Supplementary Information (Figs. S15, S34, S49, S68, S83, S98, S117, and S118). The Brain Or IntestinaL EstimateD permeation method (BOILED-Egg)^43^, a predictive model that assesses the lipophilicity and polarity of small molecules, indicated high gastrointestinal absorption for all tested compounds. Water solubility predictions, based on the Estimated SOLubility (ESOL) method, revealed significant differences between glycosylated derivatives and their aglycone counterparts. Glycosylated derivatives **1a** and **2a** showed a 16-fold increase in water solubility compared to their respective aglycone forms **1** and **2**. Compound **4a** demonstrated an even more pronounced improvement, with a 29-fold higher water solubility than its aglycone form **4**. These findings highlight the substantial impact of glycosylation on the water solubility of these molecules, potentially influencing their pharmacokinetic properties. The biotransformation products (**1a**, **2a**, and **4a**) exhibited altered membrane permeation properties compared to their aglycones (**1**, **2**, and **4**). While they lost the ability to passively penetrate the blood–brain barrier, they gained susceptibility to active transport by P-glycoprotein. Computational simulations revealed distinct cytochrome P450 enzyme inhibition profiles for the tested compounds. Flavonoid aglycones 1, 2, 3, and 4 may inhibit CYP1A2, CYP2C19 but they do not inhibit other cytochrome P450 isozymes (CYP2D6, CYP3A4, and CYP2C9—except for compound 4). However, their glycosylated derivatives (1a, 2a, and 4a) likely do not inhibit CYP1A2, CYP2C9, CYP2C19, and CYP2D6 but may inhibit CYP3A4. 0All examined molecules passed multiple drug-likeness estimators (Lipinski, Ghose, Veber, Egan, and Muegge) employed by the Swiss-ADME platform without any violations. The Abbott bioavailability score (ABS) for all tested compounds was 0.55, indicating a 55% probability of achieving either > 10% oral bioavailability in rats or measurable Caco-2 permeability. In medicinal chemistry simulations, none of the compounds triggered alerts for PAINS (Pan-Assay Interference Compounds), suggesting a low risk of false positive results in biochemical screening. These prediction results are summarized in Table 5.

**Table 5:** Pharmacokinetics, drug-likeness, and biological activity prediction data from the SwissADME online tool of compounds **1**, **1a**,** 2**, **2a**,** 3**, **4** and **4a**.

Activity/parameter	1	1a	2	2a	3	4	4a	5
Lipophilicity consensus Log Po/w	3.70	1.89	3.71	1.84	3.71	3.78	1.82	3.18
Water solubility [mg/ml]	0.00558	0.0892	0.00558	0.0892	0.00566	0.00308	0.0892	0.0180
Gastrointestinal absorption	High	High	High	High	High	High	High	High
BBB permeant	Yes	No	Yes	No	Yes	Yes	No	Yes
P-gp substrate	No	Yes	No	Yes	No	No	Yes	No
CYP1A2 inhibitor	Yes	No	Yes	No	Yes	Yes	No	Yes
CYP2C9 inhibitor	No	No	No	No	No	Yes	No	No
CYP2C19 inhibitor	Yes	No	Yes	No	Yes	Yes	No	Yes
CYP2D6 inhibitor	No	No	No	No	No	No	No	No
CYP3A4 inhibitor	No	Yes	No	Yes	No	No	Yes	No
Log Kp (skin permeation) [cm/s]	− 4.89	− 7.88	− 4.89	− 7.88	− 4.90	− 4.60	− 7.88	− 5.13
Drug-likeness (Lipinski, Ghose, Veber, Egan, and Muegge)	Yes	Yes	Yes	Yes	Yes	Yes	Yes	Yes
Abbott bioavailability score (ABS)	0.55	0.55	0.55	0.55	0.55	0.55	0.55	0.55
PAINS	0 alert	0 alert	0 alert	0 alert	0 alert	0 alert	0 alert	0 alert

From a practical standpoint, glycosylated derivatives (**1a**, **2a**, and **4a**) showed significantly improved aqueous solubility compared to their aglycone counterparts, suggesting higher potential for formulation as orally administered pharmaceuticals. However, their inability to passively cross the blood–brain barrier and increased susceptibility to efflux transporters like P-glycoprotein should be taken into account when considering therapeutic applications, particularly for central nervous system disorders. Therefore, further studies involving in vitro and in vivo bioavailability assays are recommended to confirm their pharmaceutical potential. These effects are consistent with literature reports indicating that glycosylation typically enhances flavonoid water solubility and alters membrane permeability, thereby modulating pharmacokinetic behavior and bioavailability^25,44^. The effects of flavonoid glycosylation on water solubility, membrane permeability, and bioavailability are well-documented across multiple studies. Glycosylation generally enhances water solubility by introducing polar sugar moieties, which reduces hydrophobicity. For example, (+)-catechin’s glycosylated form exhibits 40-fold higher water solubility than its aglycone^26^. This increased solubility facilitates better dissolution in biological fluids, a critical factor for absorption^45,46^. Membrane permeability is modulated through interactions with intestinal transporters. Flavonoid glycosides like quercetin-3-*O*-glucoside and cyanidin-3-*O*-glucoside are actively transported via sodium-glucose co-transporter 1 (SGLT1) and glucose transporter 2 (GLUT2)^44,47^. Experiments using SGLT1 inhibitors (e.g., phloridzin) and knockdown models confirmed these transporters’ roles, with diglycosides like cyanin showing stronger inhibition effects than monoglycosides^47^. Bioavailability improvements arise from both solubility and absorption mechanisms: glycosylated flavonoids exhibit enhanced thermal and oxidative stability compared to aglycones (e.g., rutin degrades slower than quercetin under metal-catalyzed conditions)^45^. Sugar moieties enable active transport across intestinal epithelium, bypassing passive diffusion limitations of aglycones^48^. For instance, quercetin glucosides achieve faster absorption than rhamnosides due to *β*-glucosidase activity in the small intestine^45^. Phase 2 metabolism (e.g., glucuronidation) is reduced for glycosides, preserving bioactive forms in systemic circulation^49^. These combined effects explain why glycosylation is a key strategy to optimize flavonoid pharmacokinetics for therapeutic applications^44,49,50^.

### Antimicrobial activity of compounds 1, 1a, 2, 2a, 3, 4, 4a

Antimicrobial activity tests of compounds **1**, **1a**, **2**, **2a**, **3**, **4**, **4a** were conducted using turbidimetric method on a Synergy H1 microplate reader (BioTek Instruments, Winooski, VT, USA) to evaluate the impact of a chlorine atom introduction, its position, and 4′-O-methylglucopyranose attachment on flavone activity. The tests were carried out against three strains of Gram positive bacteria: *Enterococcus faecalis* ATCC 19433, *Staphylococcus aureus* ATCC 29213, *Lactobacillus acidophilus* ATCC 4356 (lactic acid bacteria), one strain of Gram negative bacteria *Escherichia coli* ATCC 25922, and yeast strain: *Candida albicans* ATCC 1023. In all cases, the results refer specifically to the tested strains identified by their ATCC numbers, and not to the species as a whole. The obtained data on the growth of microbiological cultures, both control (only with the tested strain) and with the addition of the tested flavonoids or antibiotics, expressed as an increase in optical density (ΔOD), are presented in Table 6.

**Table 6:** Antimicrobial activity of compounds **1**, **1a**, **2**, **2a**, **3**, **4**, **4a** against microbial strains: *Enterococcus faecalis* ATCC 19433, *Staphylococcus aureus* ATCC 29213, *Lactobacillus acidophilus* ATCC 4356, *Escherichia coli* ATCC 25922, and *Candida albicans* ATCC 1023.

Compound	*E. faecalis* (Gram+)		*S. aureus* (Gram+)		*L. acidophilus* (Gram+)		*E. coli* (Gram−)		*C. albicans* (yeast)	
	ΔOD (0.05%)	ΔOD (0.1%)	ΔOD (0.05%)	ΔOD (0.1%)	ΔOD (0.05%)	ΔOD (0.1%)	ΔOD (0.05%)	ΔOD (0.1%)	ΔOD (0.05%)	ΔOD (0.1%)
Control	1.76		1.37		1.28		1.56		1.30	
Oxytetracycline	0	0	0	0	0	0	0	0	–	–
Cycloheximide	–	–	–	–	–	–	–	–	0	0
1	0.84	0.36	0.75	0.11	0.14	0.36	0.62	0.16	0.22	0.26
1a	1.72	0.96	1.37	0.46	0.22	1.03	1.07	1.04	0.34	0.13
2	0.54	0.27	0.52	0.04	0	0.36	0.46	0.35	0.91	0
2a	1.90	1.28	1.11	0.62	0.48	1.17	1.28	1.13	0.59	0.18
3	0.24	0	0.50	0.03	0.04	0.37	0.27	0.01	0.15	0
4	0.24	0.03	0.39	0	0	0.40	0.09	0	0	0
4a	1.45	1.14	1.00	0.50	0.16	1.04	1.23	1.13	0.58	0.18
5	1.04	0.97	0.83	0.26	0.25	0.79	0.54	0.65	0.40	0

**Fig. 9:**
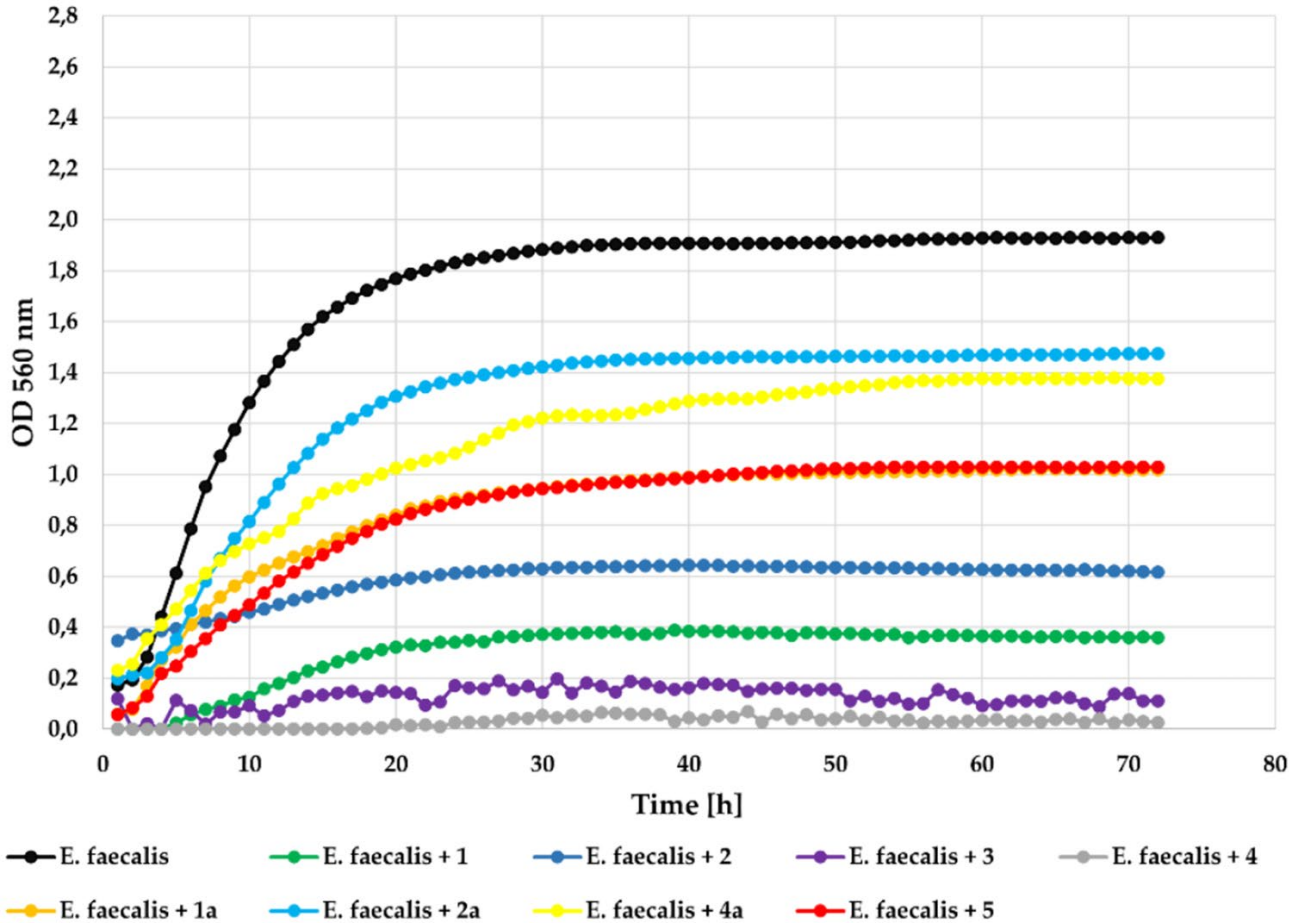
The effect of the action of compounds **1**, **1a**, **2**, **2a**, **3**, **4**, **4a**, and **5** in concentration 0.1% on the growth of *E. faecalis* ATCC 19433.

Biological assays of the obtained flavonoids allowed us to evaluate how the introduction of a chlorine atom and 4’-*O*-methylglucosyl moiety into the flavone structure affected antimicrobial activity. The strongest inhibitory effect against tested *E. faecalis* strain, (Gram+) in concentration 0,1% was observed for flavonoid aglycones **3** and **4** (ΔOD = 0). For comparison, ΔOD was 1.37 in the case of the control with the bacterial strain alone without the addition of a flavonoid compound. Other compounds also significantly inhibited bacterial growth, compound **2** with **Δ**OD of just 0.27 and **1** with **Δ**OD = 0.36. The flavonoid glycosides (**1a**, **2a**, **4a**) and flavone (**5**) were less effective but they notably inhibited bacterial growth. The growth of the *E. faecalis* strain in the presence of the tested compounds at a concentration 0.1% is shown in Fig. 9.

**Fig. 10:**
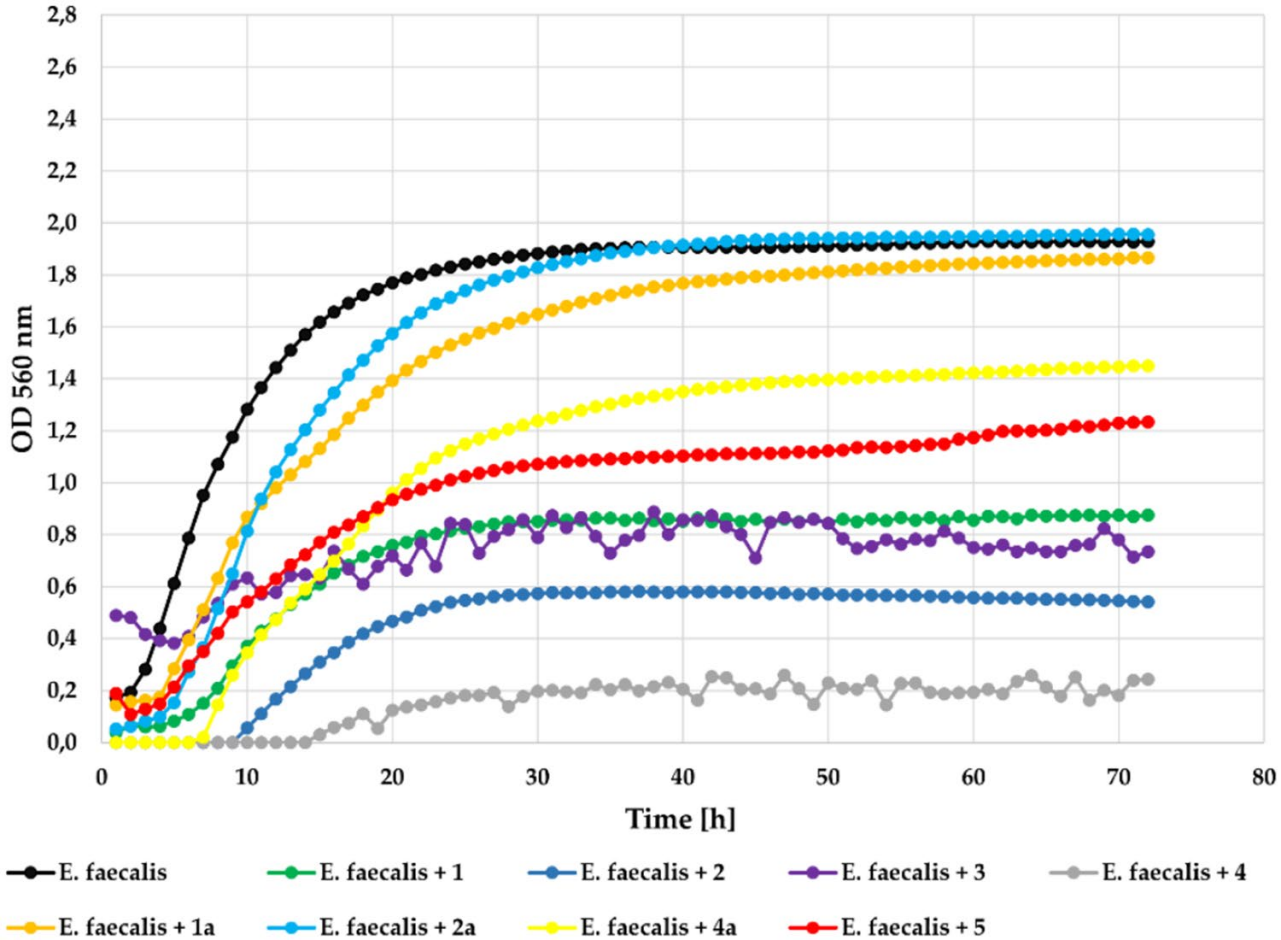
The effect of the action of compounds **1**, **1a**, **2**, **2a**, **3**, **4**, **4a**, and **5** in concentration 0.05% on the growth of *E. faecalis* ATCC 19433.

At the lower concentration (0.05%) all compounds showed correspondingly lower inhibitory activity with **Δ**OD = 0.24 in the case of compounds **3** and **4**, **Δ**OD = 0,54 in the case of compound **2**, and **Δ**OD = 0.84 in the case of compound **1**. The flavone (**5**) was less effective with **Δ**OD = 1.04. While flavonoid glycosides (**1a**, **2a**, **4a**) did not inhibit the growth of the tested strain. The growth of the tested *E. faecalis* strain in the presence of the tested compounds at a concentration 0.05% is shown in Fig. 10.

**Fig. 11:**
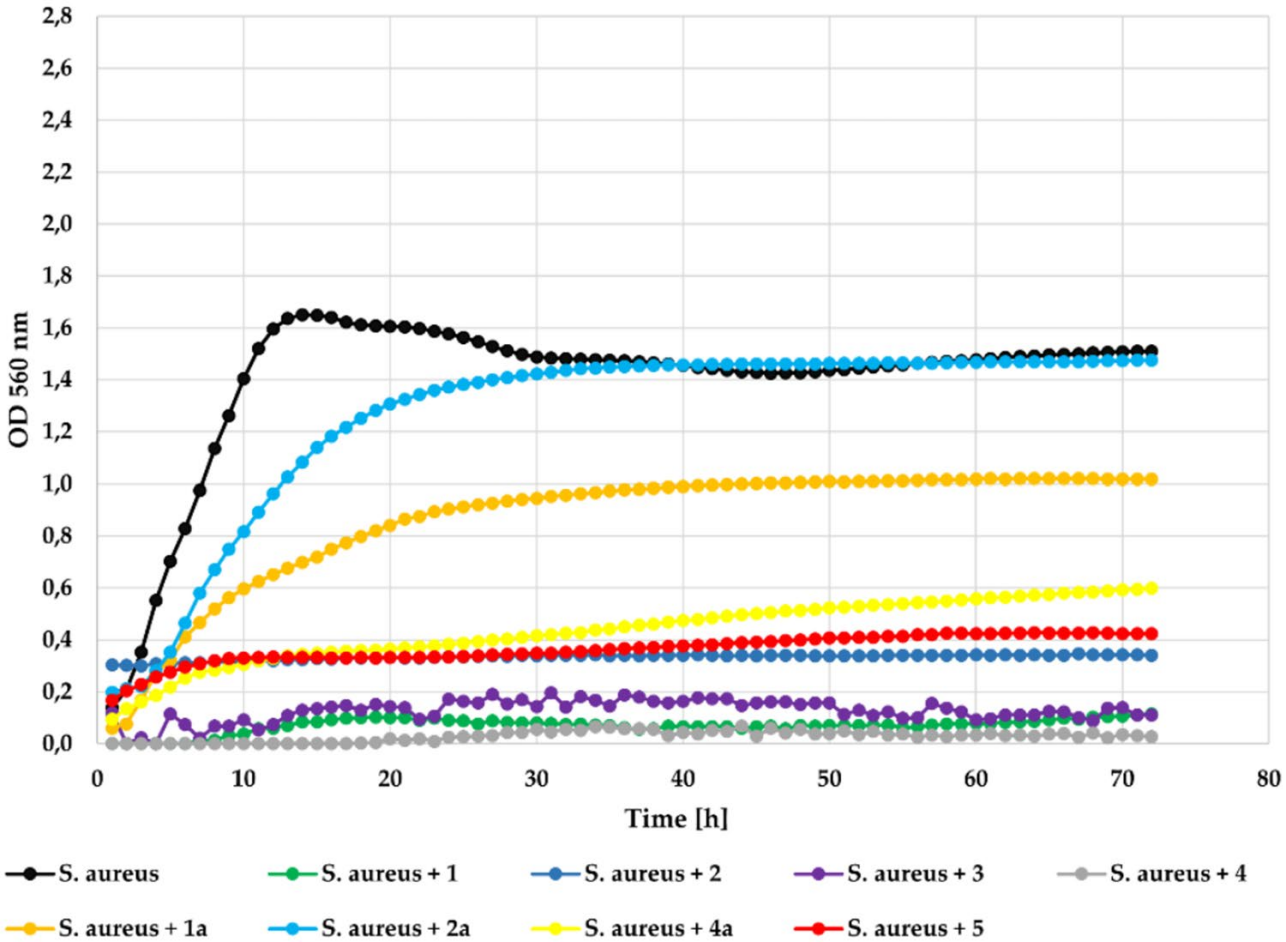
The effect of the action of compounds **1**, **1a**, **2**, **2a**,** 3**, **4**, **4a**, and **5** in concentration 0.1% on the growth of *S. aureus* ATCC 29213.

Another Gram + bacteria strain *S. aureus* was also completely inhibited by compounds 2, 3, and 4 applied at 0.1% concentration. It was strongly inhibited by the compound **1** with **Δ**OD = 0.11 and slightly less inhibited by flavone (**5**) with **Δ**OD = 0.26 in comparison to the **Δ**OD = 1.37 in the control. The flavonoid glycosides (**1a**, **2a**, **4a**) also notably inhibited bacterial growth with **Δ**OD = 0.46, **Δ**OD = 0.62, and **Δ**OD = 0.52, respectively. The growth of the tested *S. aureus* strain in the presence of the tested compounds at a concentration 0.1% is shown in Fig. 11.

**Fig. 12:**
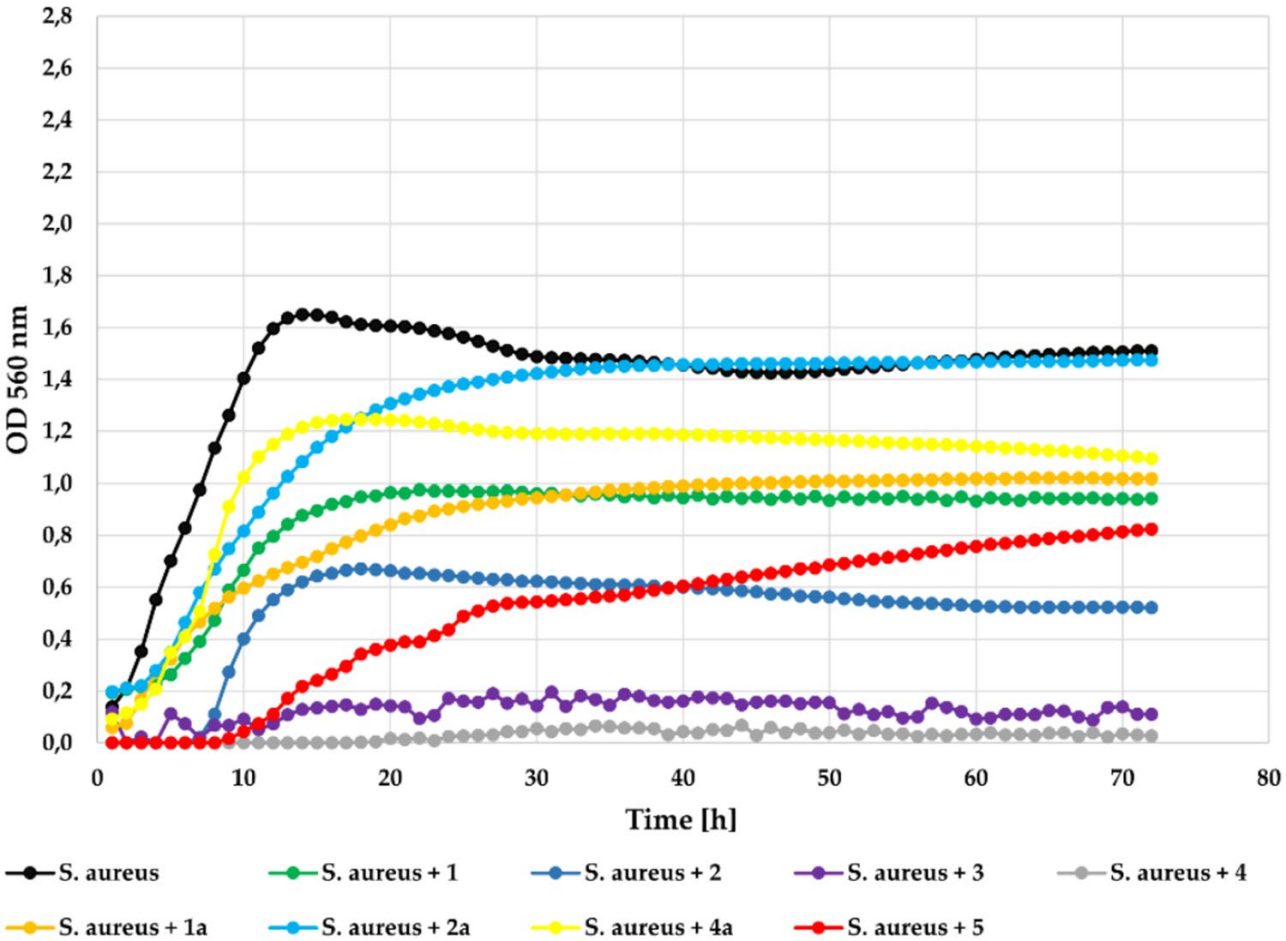
The effect of the action of compounds **1**, **1a**, **2**, **2a**, **3**,** 4**, **4a**, and **5** in concentration 0.05% on the growth of *S. aureus* ATCC 29213.

At the lower concentration (0.05%) all compounds also showed correspondingly lower inhibitory activity against strain *S. aureus* ATCC 29213. The flavone (5) was less effective with **Δ**OD = 1.04. The flavonoid glycosides (**1a**, **2a**, **4a**) almost did not inhibit their growth at all. The growth of the *S. aureus* strain in the presence of the tested compounds at a concentration 0.05% is shown in Fig. 12.

**Fig. 13:**
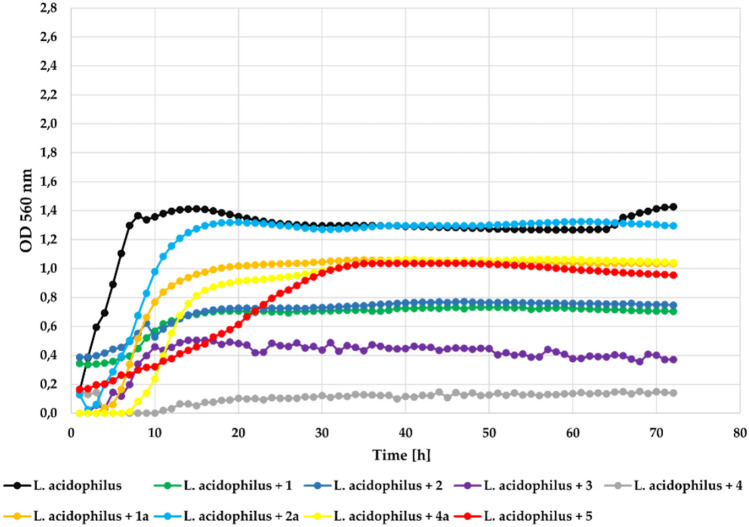
The effect of the action of compounds **1**, **1a**, **2**, **2a**, **3**, **4**, **4a**, and **5** in concentration 0.1% on the growth of *L. acidophilus* ATCC 4356.

In the case of the lactic acid bacteria—*Lactobacillus acidophilus* ATCC 4356 strain, flavonoid aglycones **1**, **2**, **3**, and **4** significantly inhibited its growth with **Δ**OD about 0.4. Compound **5** inhibited its growth in a little less effective manner with **Δ**OD = 0.79. The flavonoid glycosides (**1a**, **2a**, **4a**) only slightly inhibited its growth with **Δ**OD = 1.03, **Δ**OD = 1.17, and **Δ**OD = 1.04, respectively, which was similar to the result in the control culture. The growth of the *L. acidophilus* strain in the presence of the tested compounds at a concentration 0.1% is shown in Fig. 13. The inclusion of thise probiotic strain *L*. *acidophilus* in the test panel aimed to assess whether chlorinated flavones selectively inhibit pathogenic strains while sparing beneficial commensals. Further studies assessing potential prebiotic effects of the glycosylated derivatives on probiotic strains are warranted.

**Fig. 14:**
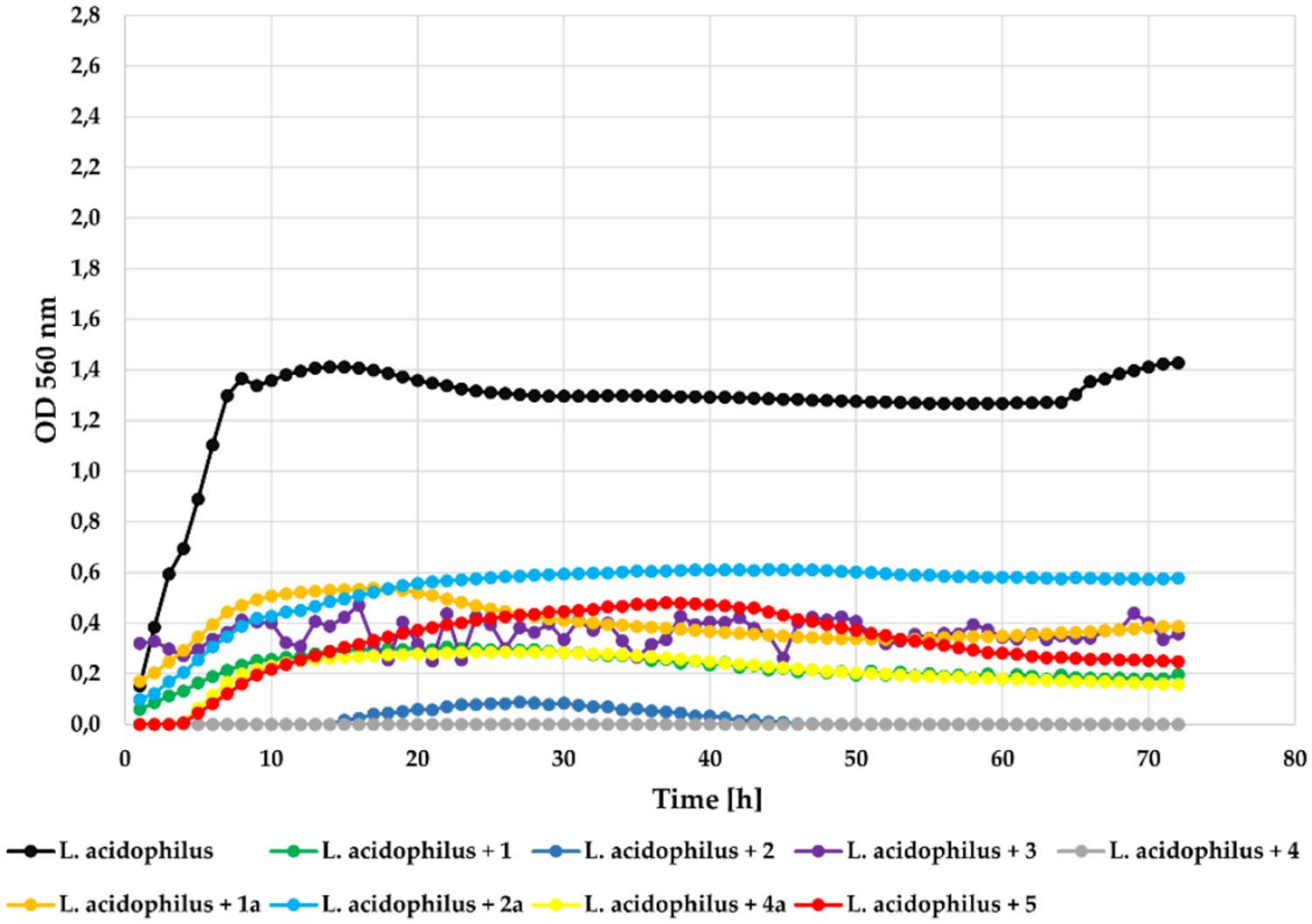
The effect of the action of compounds **1**, **1a**, **2**, **2a**, **3**, **4**, **4a**, and **5** in concentration 0.05% on the growth of *L. acidophilus* ATCC 4356.

Interestingly, at the lower concentration (0.05%) all compounds also showed higher inhibitory activity against strain *L. acidophilus*. Compounds** 2**, **3**, and **4** completely inhibited its growth. The remaining compounds also inhibited its growth to a large extent. The growth of the tested *L. acidophilus* in the presence of the tested compounds at a concentration 0.05% is shown in Fig. 14.

**Fig. 15:**
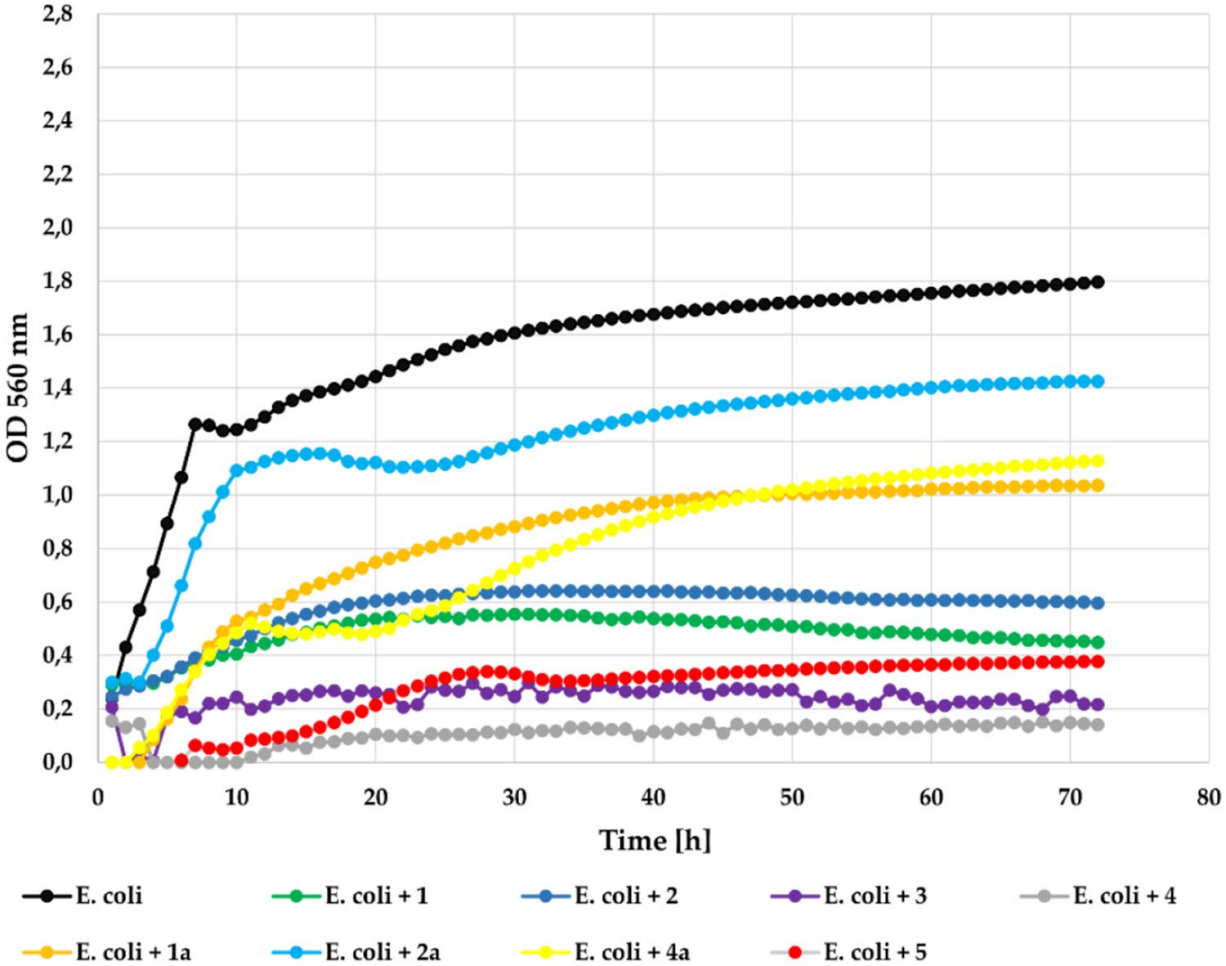
The effect of the action of compounds **1**, **1a**, **2**, **2a**, **3**, **4**, **4a**, and **5** in concentration 0.1% on the growth of *E. coli* ATCC 25922.

In the case of the *E. coli* ATCC 25922 strain (Gram-) also the most effective were compounds **3** and **4** that completely inhibited its growth at concentration 0.1%. Compounds **1** and **2** also strongly inhibited its growth with **Δ**OD = 0.16 and **Δ**OD = 0.35, respectively in relation to the control with **Δ**OD = 1.56. In this case compund 5 without a chlorine atom also was less effective with **Δ**OD = 0.65. The flavonoid glycosides (**1a**, **2a**, **4a**) were much less effective in the inhibition of the bacterial growth with **Δ**OD = 1.04, **Δ**OD = 1.13, **Δ**OD = 1.13, respectively. The growth of the *E. coli* strain against the tested compounds at a concentration 0.1% is shown in Fig. 15.

**Fig. 16:**
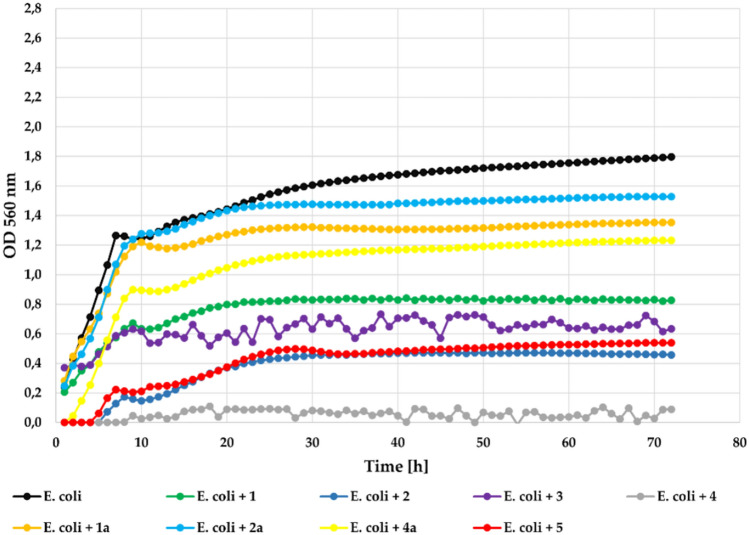
The effect of the action of compounds **1**, **1a**, **2**, **2a**, **3**, **4**, **4a**, and **5** in concentration 0.05% on the growth of *E. coli* ATCC 25922.

At the lower concentration (0.05%) all compounds showed correspondingly lower inhibitory activity. Only compound **4** showed strong inhibition of the bacteria strain with **Δ**OD = 0.09. The growth of *E. coli* strain in the presence of the tested compounds at a concentration 0.05% is shown in Fig. 16.

**Fig. 17:**
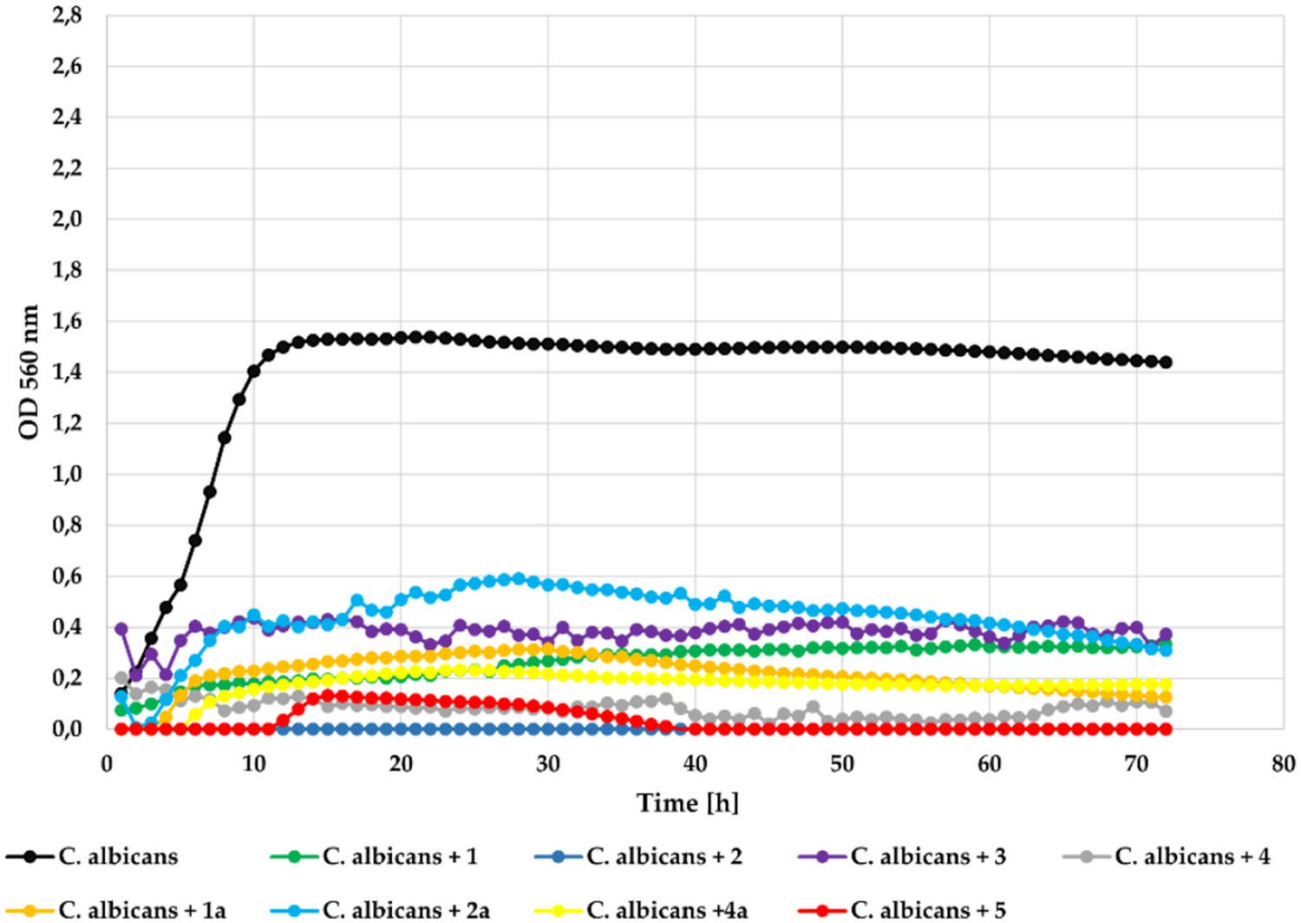
The effect of the action of compounds **1**, **1a**, **2**, **2a**, **3**, **4**, **4a**, and **5** in concentration 0.1% on the growth of *C. albicans* ATCC 1023.

The last microbial strain—yeast *Candida albicans* ATCC 1023 was very senstive to the action of all tested flavonoids. The measured **Δ**OD = 1.30 in the control dropped to 0 as a result of using compounds **2**, **3**, **4**, and **5**, which completely inhibited yeast growth at concentration 0.1%. Compounds **1**, **1a**, **2a**, and **4a** also strongly inhibited its growth with **Δ**OD = 0.26, **Δ**OD = 0.13, **Δ**OD = 0.18, and **Δ**OD = 0.18, respectively. The effect of the action of the tested compounds on *Candida albicans* at concentration 0.1% is shown in Fig. 17.

**Fig. 18:**
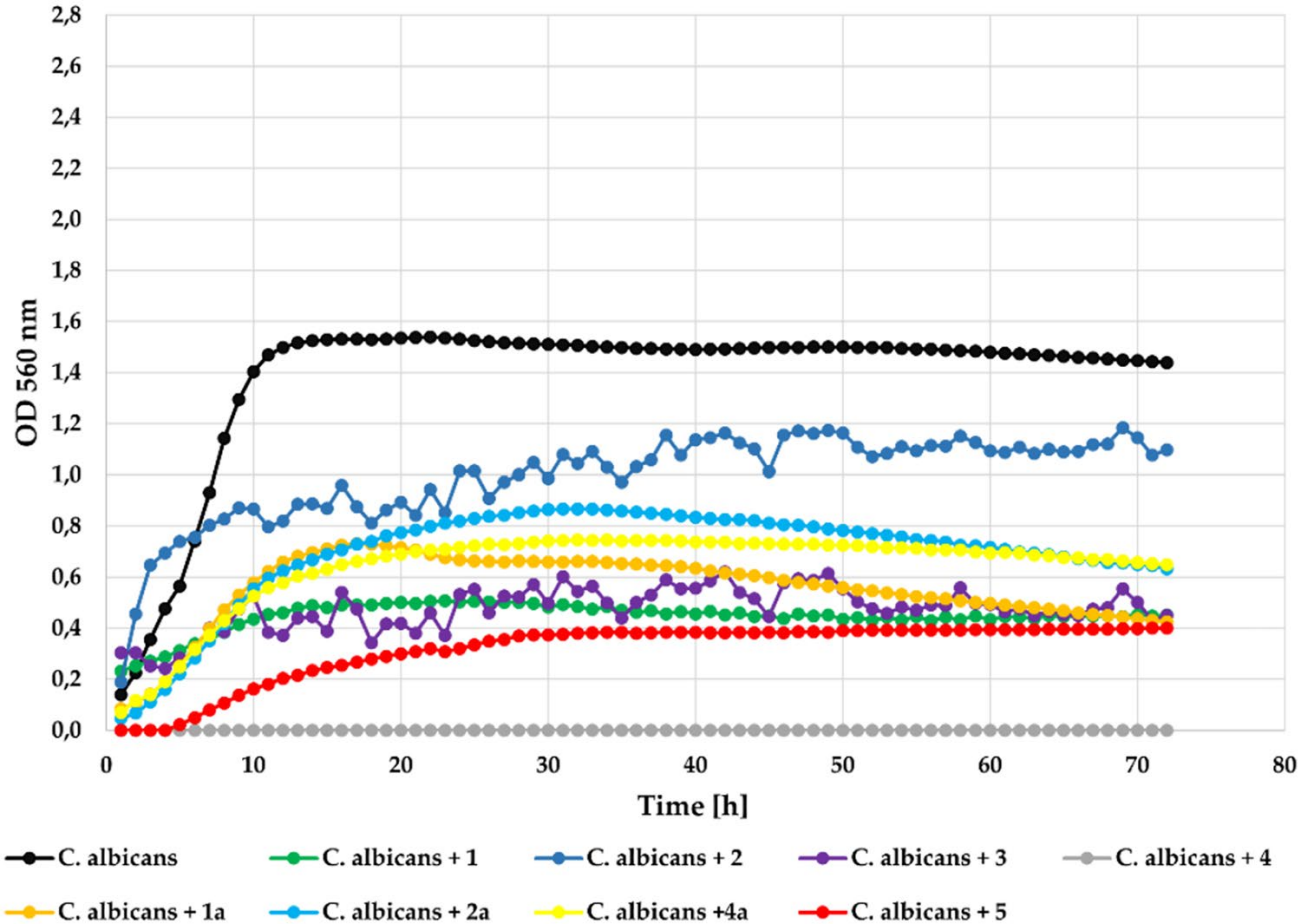
The effect of the action of compounds **1**, **1a**, **2**, **2a**,** 3**, **4**, **4a**, and **5** in concentration 0.05% on the growth of *C. albicans* ATCC 1023.

At the lower concentration (0.05%) all compounds showed correspondingly lower inhibitory activity, but still strongly inhibited yeast strain growth. The strongest inihbitory activity showed again compound **4** that completely inhibited growth of the *Candida albicans*. The growth of this strain in the presence of the tested compounds at a concentration 0.05% is shown in Fig. 18.

The antimicrobial activity screening of 2′-chloroflavone (**1**), 3′-chloroflavone (**2**), 4′-chloroflavone (**3**), 6-chloroflavone (**4**) flavone (**5**) revealed that adding a chlorine atom to the flavonoid structure enhanced their inhibitory properties against tested *E. faecalis*, *S. aureus*, *L. acidophilus*, *E. coli*, and *C. albicans* strains. On the other hand, flavones with a chlorine atom 4″-*O*-methylglycosides (**1a**, **2a**, and **4a**) were less effective than their aglycones and flavone without chlorine substituents. The literature regarding chlorine-substituted flavonoids remains limited and mostly focuses on chalcones with halogen atoms^51^. Chlorflavonin and aspergivone A, both containing a chlorine atom at the C-3′ position and differing only in the replacement of a hydroxyl group in chlorflavonin with a methoxy group in aspergivone A, exhibited significant variations in antimicrobial efficacy in previous investigations. In the literature, chlorflavonin is reported to possess antibacterial activity against *Mycobacterium tuberculosis* with an MIC90 of 1.56 μM^10^ and antifungal properties against species including *Aspergillus fumigatus*, *Aspergillus amstelodami*, *Aspergillus ochraceous*, and *Paecilomyces variotti*^6^, while such activity has not been established for aspergivone A^9^. These findings suggest that subtle structural modifications can result in substantial differences in biological activity. Our previous studies concerning 2′-hydroxychalcone also showed that an introduction of a chlorine atom positively affect antimicrobial activity of flavonoids. The effect of the methylglucosyl moiety on antimicrobial activity was ambiguous, with glycosides showing similar, slightly lower, or in some cases higher efficacy compared to their aglycone forms^36,37^. Other studies also showed that the presence of chlorine, bromine, and nitro groups has a significant effect on their antimicrobial properties^39,52^. Further comprehensive evaluation of the antimicrobial properties of these compounds is warranted, specifically elucidating their mechanistic pathways, quantifying the influence of chlorine and glucosyl moieties, and conducting in vivo investigations. The impact of glycosylation on flavonoid bioactivity remains inconclusive based on current literature. Moreover, the differential effects of flavonoid glycosylation may exhibit substantial variance between in vitro and in vivo experimental models. Comparing our results with literature data highlights the significant influence of chlorine substitution on flavonoid antimicrobial activity. For instance, chlorflavonin exhibits strong antimicrobial effects against fungi and *Mycobacterium tuberculosis*^10^, which aligns with our findings regarding chlorinated aglycones’ superiority over glycosides. Similarly, previous reports indicated variable biological activities based on chlorine substituent position, suggesting that minor structural differences profoundly influence bioactivity. Our findings further expand this understanding, demonstrating a similar trend in bacterial and yeast models and emphasizing the potential of chloroflavones as antimicrobial agents.

The exact molecular mechanisms of action of chlorinated flavonoids are still under investigation^39^. Some studies suggest that halogenated flavonoids may disrupt bacterial membranes or interfere with key enzymatic systems^53–55^. Recent studies demonstrate that halogenated flavonoids disrupt bacterial membranes by interacting with lipid head groups and hydrocarbon chains, increasing membrane permeability and causing structural deformities like shrinkage and adhesion^54^. These compounds also penetrate deeper membrane layers, altering fluidity and enabling intracellular component leakage^54,56^. Enzymatic interference appears equally critical, with chlorinated flavonoids inhibiting DNA gyrase by binding to its ATP pocket through strategic hydroxyl group positioning (C-3, C-5, C-7)^56^ and suppressing oxidative phosphorylation genes (QoxC, QoxD), disrupting energy metabolism^3,57^. Structural features drive these effects: halogen atoms enhance lipophilicity for membrane penetration^54^, while specific hydroxylation patterns (e.g., 3′,4′-dihydroxyl in ring B) facilitate hydrogen bonding with enzymatic targets^58^. The potential role of membrane interaction and enzyme inhibition needs further exploration in future studies using molecular modeling and targeted biochemical assays.

## Materials and methods

### Substrates

Biotransformation substrates, i.e., 2′-chloroflavone (**1**), 3′-chloroflavone (**2**), 4′-chloroflavone (**3**) and 6-chloroflavone (**4**) were obtained using chemical synthesis from corresponding 2′-hydroxychalcones with a chlorine atom from our previous studies^36,37^. The Figures of chemical reactions are shown above in the Sect.  2. Results and discussion (Fig. 1). Briefly, flavones were obtained by cyclization of 2′-hydroxychalcones with a chlorine atom in the presence of iodine excess with yields: 83,3% (**1**), 68,7% (**2**), 81,0% (**3**), 92,8% (**4**)^36,37,59^.

The physical data of synthesized flavones (**1**–**4**), including color and form, molecular ion mass, molecular formula, melting point (°C), retention time t_R_ (min), and NMR spectral data are presented below, in Tables 1, 2, 3, 4, and the Supplementary Information.

2′-Chloroflavone (**1**): white crystals, ESISMS m/z 257.0 ([M + H]^+^, C_15_H_9_ClO_2_), mp = 117–119 °C^60^, t_R_ = 16.43, ^1^H-NMR, see Table 1, ^13^C-NMR, see Table 2; Supplementary Information: Figs. S1–S15.

3′-Chloroflavone (**2**): light-yellow crystals, ESISMS m/z 257.0 ([M + H]^+^, C_15_H_9_ClO_2_), mp = 123–124 °C^61^, t_R_ = 17.01, ^1^H-NMR, see Table 3, ^13^C-NMR, see Table 4, Supplementary Information: Figs. S35–S49.

4′-Chloroflavone (**3**): whitish green crystals, ESISMS m/z 257.0 ([M + H]^+^, C_15_H_9_ClO_2_), mp = 185–187 °C^62^, t_R_ = 17.04, ^1^H-NMR, see Table 3, ^13^C-NMR, see Table 4, Supplementary Information: Figs. S69–S83.

6-Chloroflavone (**4**): white crystals, ESISMS m/z 257.0 ([M + H]^+^, C_15_H_9_ClO_2_), mp = 182–183 °C^63^, t_R_ = 17.27, ^1^H-NMR, see Table 3, ^13^C-NMR, see Table 4, Supplementary Information: Figs. S84–S98.

### Microorganisms

Two fungal strains from the *Cordycipitaceae* family—*Isaria fumosorosea* KCH J2 and *Beauveria bassiana* KCH J1.5—were used to study the biotransformation of chloroflavones **1–4**. These strains were obtained from the culture collection maintained at the Faculty of Biotechnology and Food Microbiology, Wrocław University of Environmental and Life Sciences (Poland). The methods for isolation, cultivation, and molecular identification of these entomopathogenic fungi have been previously documented in our earlier publications^20,29^.

### Analysis

The progress of biotransformation was monitored using thin-layer chromatography (TLC) and high-performance liquid chromatography (HPLC) to analyze substrate conversion.

TLC analyses were performed on Silica gel 60/Kieselguhr F254 plates (0.2 mm thickness; Merck, Darmstadt, Germany). A chloroform:methanol (9:1 *v/v*) mixture served as the mobile phase, using chloroform from POCH (Gliwice, Poland) and methanol from Chempur (Piekary Śląskie, Poland). The compounds were visualized under UV light at wavelengths of 254 nm and 365 nm without additional visualization^36^.

HPLC analyses were conducted using a Dionex Ultimate 3000 system (Thermo Fisher Scientific, Waltham, MA, USA) equipped with a DAD-3000 diode array detector. Separation was achieved on an ODS 2 analytical column (4.6 × 250 mm, Waters, Milford, MA, USA) with a corresponding pre-column. The mobile phase consisted of two solvents: (A) 0.1% formic acid in water *v*/*v* (Honeywall, Charlotte, North Carolina, US; water from Supelco, Darmstadt, Germany) and (B) 0.1% formic acid in acetonitrile *v*/*v* (Supelco, Darmstadt, Germany). The gradient elution program was set as follows: initial conditions—32.5% B in A, 4 min—40% B in A, 8 min—40% B in A, 10 min—45% B in A, 15 min—95% B in A, 18 min—95% B in A, 19 min—32.5% B in A, 23 min—32.5% B in A. Samples were prepared at 1 mg/mL in acetonitrile, with an injection volume of 10 µL. The flow rate was maintained at 1 mL/min. Detection was performed at 254 nm for flavonoid glycosides and 280 nm for flavonoid aglycones^36^.

The biotransformation products were separated and isolated using preparative TLC on silica gel plates (500 µm and 1000 µm thickness; Analtech, Gehrden, Germany). Separation was achieved using a chloroform:methanol (9:1 *v*/*v*) mobile phase. The compounds of interest were recovered by extracting the scraped silica gel bands three times using 20 mL ethyl acetate (Stanlab, Lublin, Poland), followed by solvent removal under reduced pressure using a rotary evaporator (Heidolph, Schwabach, Germany)^36^.

Structural characterization was carried out using a Bruker DRX Avance 600 MHz spectrometer through a series of NMR experiments (^1^H, ^13^C, COSY, HSQC, and HMBC) with deuterated acetone as the samples solvent. The optical rotation measurements were conducted on an ABL&E-JASCO P-2000-Na digital polarimeter (Kraków, Poland).

Molecular formula confirmation for all compounds (**1**, **1a**, **2**, **2a**, **3**, **4**, and **4a**) was performed using a SHIMADZU LC–MS 8045 Triple Quadrupole mass spectrometry system equipped with an electrospray ionization (ESI) source (Shimadzu, Kyoto, Japan), following previously described methods. The injection volume was 10 µL of samples in a concentration of 1 µg/mL (dissolved in methanol). The principal operating parameters for the LC–MS were set as follows: nebulizing gas flow: 3 L/min, heating gas flow: 10 L/min, interface temperature: 300 °C, drying gas flow: 10 L/min, data acquisition range, *m*/*z* 100–500 Da; ionization mode, negative (flavonoid glycosides) and positive (flavonoid aglycones). Data were collected with LabSolutions version 5.97 (Shimadzu, Kyoto, Japan) software.

### Screening procedure

A preliminary screening was conducted to evaluate both the biotransformation potential and optimal conversion time of substrates **1–4** before proceeding with semipreparative-scale experiments. The experiments were performed using a modified Sabouraud medium (10 g aminobac and 30 g glucose from BTL, Warsaw, Poland, per 1 L distilled water). The biotransformation process consisted of two cultivation stages. Initially, fungal strains were transferred from potato slants to modified Sabouraud liquid medium (100 mL) in 300 mL Erlenmeyer flasks. These cultures were incubated for 72 h at 25 °C with shaking at 140 rpm (DHN shaker, Warsaw, Poland). Subsequently, 1 mL of these pre-cultures was transferred to fresh medium (100 mL) in new 300 mL Erlenmeyer flasks and incubated under identical conditions. The biotransformation was initiated by adding 10 mg of one of substrates **1–4** (dissolved in 0.5 mL dimethyl sulfoxide; Chempur, Piekary Śląskie, Poland) to cultures at the end of their logarithmic growth phase of either *I. fumosorosea* KCH J2 or *B. bassiana* KCH J1.5, achieving a final substrate concentration of 0.39 mM. Samples were collected at 3, 6, and 8 days post-substrate addition. Products were extracted with ethyl acetate (30 mL), dried over anhydrous magnesium sulfate (Chempur, Piekary Śląskie, Poland), and concentrated using a rotary evaporator at 55 °C. The experiments were terminated after 8 days when either complete substrate conversion was achieved or no further reaction progress was observed, as monitored by TLC and HPLC. Control experiments included substrate stability tests under biotransformation conditions and microorganism cultivation without substrate addition^36^.

### The semipreparative biotransformation

The large-scale biotransformations were performed in 2 L flasks containing 500 mL of modified Sabouraud medium. This scale enabled isolation of sufficient product quantities for structural characterization by NMR. The process commenced with the transfer of 5 mL preincubation culture of either *I. fumosorosea* KCH J2 or *B. bassiana* KCH J1.5 into individual flasks, followed by a 72-h incubation under conditions matching the screening protocol.

Each substrate (**1–4**) was dissolved in 2.0 mL dimethyl sulfoxide and added separately to the fungal cultures at 0.39 mM final concentration, consistent with screening conditions. Although initially planned for 8-day incubations based on screening results, the experiments were terminated upon confirmation of complete substrate conversion.

Product isolation involved triple extraction of reaction mixtures using 300 mL ethyl acetate portions. The combined organic phases underwent brief drying over anhydrous magnesium sulfate (5 min), filtration, and solvent evaporation. Product purification employed preparative TLC plates as previously described. Product bands were detected under UV light, isolated, and extracted three times with 20 mL ethyl acetate. The purified compounds underwent spectroscopic analysis for structural elucidation. Product yields were calculated based on the isolated masses of product^36^. Below Table 7 summarize the biotransformation yields and key reaction conditions.

**Table 7:** Summary of the biotransformation yields and key reaction conditions.

Substrate	Fungal strain	Biotransformation yield (%)
2′-Chloroflavone (**1**)	*I. fumosorosea* KCH J2	10.8
	*B. bassiana* KCH J1.5	15.2
3′-Chloroflavone (**2**)	*I. fumosorosea* KCH J2	40.5
	*B. bassiana* KCH J1.5	48.6
6-Chloroflavone (**4**)	*I. fumosorosea* KCH J2	62.0
	*B. bassiana* KCH J1.5	12.6

Reaction conditions: flask volume: 2 L, medium: modified Sabouraud medium, medium volume: 500 mL, inoclumu: 5 mL preculture, substrate concentration: 0.39 mM, substrate solvent: DMSO (2.0 mL)

### Fungal biotransformation products

The characterization data for fungal biotransformation products **1a**, **2a**, and **4a** are detailed below, above in Tables 1, 2, 3, 4 and the Supplementary Information. This includes their physical properties (color, form, melting point), chromatographic retention time tR, molecular characteristics (mass, formula), and complete NMR spectral data.

2′-Chloroflavone 3′-*O*-*β*-D-(4″-*O*-methyl)-glucopyranoside (**1a**): white crystals, ESI/MS m/z 449.1 ([M + H]^+^, C_22_H_21_ClO_8_), mp = 96–98 °C, t_R_ = 4.94; ^1^H-NMR, see Table 1, ^13^C-NMR, see Table 2, Supplementary Information: Figs. S16–S34.

3′-Chloroflavone 4′-*O*-*β*-D-(4″-*O*-methyl)-glucopyranoside (**2a**): white crystals, ESI/MS m/z 449.1 ([M + H]^+^, C_22_H_2a_ClO_8_), mp = 114–116 °C, t_R_ = 5.25; ^1^H-NMR, see Table 1, ^13^C-NMR, see Table 2, Supplementary Information: Figs. S50–S68.

6-Chloroflavone 4′-*O*-*β*-D-(4′’-*O*-methyl)-glucopyranoside (**4a**): white crystals, ESI/MS m/z 449.1 ([M + H]^+^, C_22_H_25_ClO_9_), mp = 121–123 °C, t_R_ = 6.44; ^1^H-NMR, see Table 1, ^13^C-NMR, see Table 2, Supplementary Information: Figs. S99–S117.

### Pharmacokinetics, drug nature, biological activity predictions

The structural formulae of flavonoids **1**, **1a**, **2**, **2a**, **3**, **4**, **4a**, and **5** were analyzed using a computational platform to predict their properties. SwissADME (http://www.swissadme.ch) was used to evaluate pharmacokinetics, physicochemical properties, and drug-like characteristics (accessed on January 10, 2025). The molecular structures were initially constructed using ACD Chemsketch 2021.2.0 and exported as .mol files for input into SwissADME. The prediction results are shown in the Supplementary Information: Figures S15 (**1**), S34 (**1a**), S49 (**2**), S68 (**2a**), S83 (**3**), S98 (**4**), S117 (**4a**), S118 (**5**).

### Antimicrobial activity assays

Antimicrobial activity evaluation of compounds **1, 1a, 2, 2a, 3, 4, 4a**, and also** 5** (purchased from Sigma-Aldrich, Sant Louis, MO, USA) was conducted using the BioTek microplate reader (Winooski, Vermont, United States). The test panel comprised bacterial strains: *Enterococcus faecalis* ATCC 19433, *Staphylococcus aureus* ATCC 29213, *Lactobacillus acidophilus* ATCC 4356, *Escherichia coli* ATCC 25922, and yeast strain *Candida albicans* ATCC 1023. All microorganisms were sourced from the collection of the Faculty of Biotechnology and Food Microbiology, Wrocław University of Environmental and Life Sciences. Prior to analysis, the microorganisms underwent 48-h cultivation in the Mueller–Hinton broth purchased from Merck (Darmstadt, Germany). Assays were performed in 96-well microtiter plates with 300 μL total volume per well, comprising 240 μL culture medium, 50 μL microbial suspension (standardized cell density), and 10 μL flavonoid solution (0.1% m/v and 0.05% in dimethyl sulfoxide). The growth monitoring was conducted at 37 °C for 72 h with OD measurements at 560 nm taken every 60 min. All experiments were conducted in triplicate with continuous shaking of the microplate. As positive controls, oxytetracycline and cycloheximide were employed, both acquired from Sigma-Aldrich (Saint Louis, MO, USA). The data were analysed using a software Microsoft Excel 365. Growth curves were generated using mean absorbance values, and antimicrobial activity was expressed as the change in optical density (**Δ**OD) relative to control cultures containing only dimethyl sulfoxide.

## Conclusions

The incorporation of a chlorine atom and a glucosyl moiety can significantly modify the biological activity and bioavailability of flavonoids. In our research, we demonstrated the remarkable ability of entomopathogenic fungi strains *Isaria fumosorosea* KCH J2 and *Beauveria bassiana* KCH J1.5 to synthesize 4-*O*-methylglucosides from synthetic chloroflavones with chlorine atoms positioned at various locations on the flavonoid skeleton (2′, 3′, 4′, and 6). Both microbial strains transformed the chloroflavones through identical pathways, differing only in conversion efficiency. 2′-Chloroflavone underwent biotransformation to yield 2′-chloroflavone 3′-*O*-*β*-D-(4″-*O*-methyl)-glucopyranoside (**1a**), while 3′-chloroflavone was converted to 3′-chloroflavone 4′-*O*-*β*-D-(4″-*O*-methyl)-glucopyranoside (**2a**). Interestingly, 4′-chloroflavone remained resistant to biotransformation by either strain, likely due to steric hindrance affecting the microbial glycosylation enzymes. Conversely, 6-chloroflavone was successfully transformed into 6-chloroflavone 4′-*O*-*β*-D-(4″-*O*-methyl)-glucopyranoside (**4a**). The maximum conversion efficiency achieved in our study was 62% for the formation of **4a**. To our knowledge, all biotransformation products obtained in this study represent novel compounds not previously documented in the literature.

The methodologies outlined in this paper facilitate the efficient and economical production of chlorinated glycoside derivatives in quantities sufficient for comprehensive analysis of their biological properties and bioavailability. Moreover, we conducted computational investigations of the obtained compounds based on structure–activity relationships to preliminarily evaluate their biological potential and streamline screening protocols for subsequent research. Nevertheless, comprehensive in vitro and in vivo studies remain essential for fully characterizing their biological activities, pharmacokinetics, and molecular mechanisms of action as potential therapeutic agents. Our antimicrobial screening revealed that the introduction of a chlorine atom into the flavone structure enhances antimicrobial efficacy against the tested microorganisms, with activity varying according to the chlorine atom’s position. Contrary to the predictions from Pass Online simulations, we did not observe enhanced activity of flavonoid glycosides compared to their corresponding aglycones against Gram-positive bacteria. Additional experiments are necessary to elucidate the precise mechanisms of antimicrobial action. Our findings may contribute to improving the training algorithms utilized in cheminformatics tools for predicting the biological potential of chemical entities. Importantly, compounds **1a**, **2a**, and **4a** obtained through biotransformation constitute completely novel chemical structures, significantly contributing to the existing knowledge about chlorinated flavonoids and their glycosylation pathways.

Future studies should focus on elucidating the precise molecular mechanisms underlying the observed antimicrobial activity. In particular, assessing cytotoxicity profiles, determining in vivo efficacy, and performing pharmacokinetic studies would greatly enhance the understanding of the therapeutic potential of these novel glycosylated chloroflavones. Additionally, exploring broader enzyme panels from other entomopathogenic strains might lead to further valuable derivatives with distinct bioactivities. Future research should include comprehensive safety assessment of secondary metabolites produced by the fungal strains, with routine monitoring protocols implemented for any potential scale-up applications. Further studies including in vivo antimicrobial efficacy and safety evaluations are warranted to confirm the therapeutic potential of the synthesized glycosylated derivatives. Additionally, future studies should include testing against multi-drug resistant clinical isolates to further assess the broad-spectrum potential of the chlorinated flavonoids. Future research will also expand the antimicrobial testing panel to include multi-drug resistant clinical isolates to better evaluate the broad-spectrum potential of the compounds. Further investigation of other biological properties of the glycosylated derivatives, such as anti-inflammatory and antioxidant activity, is warranted to explore their full pharmacological potential.

## Supplementary Information

Supplementary Information.

## Data Availability

The original data presented in the study are included in the article and Supplementary Information. Raw data are openly available in the Wrocław University of Environmental and Life Sciences Repository (https://bazawiedzy.upwr.edu.pl/info/researchdata/UPWRc660874f38224b378a034d96b48cfba5/).
