# Supplementary material for: Antimicrobial activity of new glycoside derivatives of chloroflavones obtained by fungal biotransformation
Source: Sci Rep. 2025 Jul 16;15:25821. doi: 10.1038/s41598-025-11079-7 (PMC12267686; doi:10.1038/s41598-025-11079-7)
Supplement: Supplementary file 1 — Supplementary Information. [file 41598_2025_11079_MOESM1_ESM.pdf]

Antimicrobial activity of new glycoside derivatives of chloroflavones obtained by fungal biotransformation, and their antimicrobial potential

Agnieszka Krawczyk-Łebek[1]*, Tomasz Janeczko[1], Edyta Kostrzewa-Susłow[1]

[1]Department of Food Chemistry and Biocatalysis, Faculty of Biotechnology and Food Science, Wrocław University of Environmental and Life Sciences, Wrocław, Poland

Table of contents

Figure S1. MS analysis of 2′-chloroflavone (**1**).
Figure S2. HPLC analysis of 2′-chloroflavone (**1**).
Figure S3. ^{1}H NMR spectrum (*δ*, acetone-d6, 600 MHz) of 2′-chloroflavone (**1**).
Figure S4. ^{1}H NMR spectrum expansion (*δ*, acetone-d6, 600 MHz) of 2′-chloroflavone (**1**).
Figure S5. ^{13}C NMR spectrum (*δ*, acetone-d6, 151 MHz) of 2′-chloroflavone (**1**).
Figure S6. ^{13}C NMR spectrum expansion (*δ*, acetone-d6, 151 MHz) of 2′-chloroflavone (**1**).
Figure S7. ^{13}C NMR spectrum expansion (*δ*, acetone-d6, 151 MHz) of 2′-chloroflavone (**1**).
Figure S8. COSY contour map – ^{1}H x ^{1}H of 2′-chloroflavone (**1**).
Figure S9. COSY contour map – ^{1}H x ^{1}H expansion of 2′-chloroflavone (**1**).
Figure S10. HMQC contour map – ^{1}H x ^{13}C of 2′-chloroflavone (**1**).
Figure S11. HMQC contour map – ^{1}H x ^{13}C expansion of 2′-chloroflavone (**1**).
Figure S12. HMBC contour map – ^{1}H x ^{13}C of 2′-chloroflavone (**1**).
Figure S13. HMBC contour map – ^{1}H x ^{13}C expansion of 2′-chloroflavone (**1**).
Figure S14. HMBC contour map – ^{1}H x ^{13}C expansion of 2′-chloroflavone (**1**).
Figure S15. 2′-Chloroflavone (**1**) physicochemical and ADME parameters prediction using the SwissADME modelling.

Figure S16. MS analysis of 2′-chloroflavone 3′-*O*-*β*-D-(4′′-*O*-methyl)-glucopyranoside (**1a**).
Figure S17. HPLC analysis of 2′-chloroflavone 3′-*O*-*β*-D-(4′′-*O*-methyl)-glucopyranoside (**1a**).
Figure S18. ^{1}H NMR spectrum (*δ*, acetone-d6, 600 MHz) of 2′-chloroflavone 3′-*O*-*β*-D-(4′′-*O*-methyl)-glucopyranoside (**1a**).
Figure S19. ^{1}H NMR spectrum expansion (*δ*, acetone-d6, 600 MHz) of 2′-chloroflavone 3′-*O*-*β*-D-(4′′-*O*-methyl)-glucopyranoside (**1a**).
Figure S20. ^{1}H NMR spectrum expansion (*δ*, acetone-d6, 600 MHz) of 2′-chloroflavone 3′-*O*-*β*-D-(4′′-*O*-methyl)-glucopyranoside (**1a**).
Figure S21. ^{13}C NMR spectrum (*δ*, acetone-d6, 151 MHz) of 2′-chloroflavone 3′-*O*-*β*-D-(4′′-*O*-methyl)-glucopyranoside (**1a**).
Figure S22. ^{13}C NMR spectrum expansion (*δ*, acetone-d6, 151 MHz) of 2′-chloroflavone 3′-*O*-*β*-D-(4′′-*O*-methyl)-glucopyranoside (**1a**).
Figure S23. ^{13}C NMR spectrum expansion (*δ*, acetone-d6, 151 MHz) of 2′-chloroflavone 3′-*O*-*β*-D-(4′′-*O*-methyl)-glucopyranoside (**1a**).
Figure S24. COSY contour map – ^{1}H x ^{1}H of 2′-chloroflavone 3′-*O*-*β*-D-(4′′-*O*-methyl)-glucopyranoside (**1a**).

Figure S25. COSY contour map – ^{1}H x ^{1}H expansion of 2′-chloroflavone 3′-*O*-*β*-D-(4′′-*O*-methyl)-glucopyranoside (**1a**).
Figure S26. COSY contour map – ^{1}H x ^{1}H expansion of 2′-chloroflavone 3′-*O*-*β*-D-(4′′-*O*-methyl)-glucopyranoside (**1a**).
Figure S27. HMQC contour map – ^{1}H x ^{13}C of 2′-chloroflavone 3′-*O*-*β*-D-(4′′-*O*-methyl)-glucopyranoside (**1a**).
Figure S28. HMQC contour map – ^{1}H x ^{13}C expansion of 2′-chloroflavone 3′-*O*-*β*-D-(4′′-*O*-methyl)-glucopyranoside (**1a**).
Figure S29. HMQC contour map – ^{1}H x ^{13}C expansion of 2′-chloroflavone 3′-*O*-*β*-D-(4′′-*O*-methyl)-glucopyranoside (**1a**).
Figure S30. HMBC contour map – ^{1}H x ^{13}C of 2′-chloroflavone 3′-*O*-*β*-D-(4′′-*O*-methyl)-glucopyranoside (**1a**).
Figure S31. HMBC contour map – ^{1}H x ^{13}C expansion of 2′-chloroflavone 3′-*O*-*β*-D-(4′′-*O*-methyl)-glucopyranoside (**1a**).
Figure S32. HMBC contour map – ^{1}H x ^{13}C expansion of 2′-chloroflavone 3′-*O*-*β*-D-(4′′-*O*-methyl)-glucopyranoside (**1a**).
Figure S33. HMBC contour map – ^{1}H x ^{13}C expansion of 2′-chloroflavone 3′-*O*-*β*-D-(4′′-*O*-methyl)-glucopyranoside (**1a**).
Figure S34. 2′-Chloroflavone 3′-*O*-*β*-D-(4′′-*O*-methyl)-glucopyranoside (**1a**) physicochemical and ADME parameters prediction using the SwissADME modelling.

Figure S35. MS analysis of 3′-chloroflavone (**2**).
Figure S36. HPLC analysis of 3′-chloroflavone (**2**).
Figure S37. ^{1}H NMR spectrum (*δ*, acetone-d6, 600 MHz) of 3′-chloroflavone (**2**).
Figure S38. ^{1}H NMR spectrum expansion (*δ*, acetone-d6, 600 MHz) of 3′-chloroflavone (**2**).
Figure S39. ^{13}C NMR spectrum (*δ*, acetone-d6, 151 MHz) of 3′-chloroflavone (**2**).
Figure S40. ^{13}C NMR spectrum expansion (*δ*, acetone-d6, 151 MHz) of 3′-chloroflavone (**2**).
Figure S41. ^{13}C NMR spectrum expansion (*δ*, acetone-d6, 151 MHz) of 3′-chloroflavone (**2**).
Figure S42. COSY contour map – ^{1}H x ^{1}H of 3′-chloroflavone (**2**).
Figure S43. COSY contour map – ^{1}H x ^{1}H expansion of 3′-chloroflavone (**2**).
Figure S44. HMQC contour map – ^{1}H x ^{13}C of 3′-chloroflavone (**2**).
Figure S45. HMQC contour map – ^{1}H x ^{13}C expansion of 3′-chloroflavone (**2**).
Figure S46. HMBC contour map – ^{1}H x ^{13}C of 3′-chloroflavone (**2**).
Figure S47. HMBC contour map – ^{1}H x ^{13}C expansion of 3′-chloroflavone (**2**).
Figure S48. HMBC contour map – ^{1}H x ^{13}C expansion of 3′-chloroflavone (**2**).
Figure S49. 3′-Chloroflavone (**2**) physicochemical and ADME parameters prediction using the SwissADME modelling.

Figure S50. MS analysis of 3′-chloroflavone 4′-*O*-*β*-D-(4′′-*O*-methyl)-glucopyranoside (**2a**).
Figure S51. HPLC analysis of 3′-chloroflavone 4′-*O*-*β*-D-(4′′-*O*-methyl)-glucopyranoside (**2a**).
Figure S52. ^{1}H NMR spectrum (*δ*, acetone-d6, 600 MHz) of 3′-chloroflavone 4′-*O*-*β*-D-(4′′-*O*-methyl)-glucopyranoside (**2a**).
Figure S53. ^{1}H NMR spectrum expansion (*δ*, acetone-d6, 600 MHz) of 3′-chloroflavone 4′-*O*-*β*-D-(4′′-*O*-methyl)-glucopyranoside (**2a**).
Figure S54. ^{1}H NMR spectrum expansion (*δ*, acetone-d6, 600 MHz) of 3′-chloroflavone 4′-*O*-*β*-D-(4′′-*O*-methyl)-glucopyranoside (**2a**).
Figure S55. ^{13}C NMR spectrum (*δ*, acetone-d6, 151 MHz) of 3′-chloroflavone 4′-*O*-*β*-D-(4′′-*O*-methyl)-glucopyranoside (**2a**).
Figure S56. ^{13}C NMR spectrum expansion (*δ*, acetone-d6, 151 MHz) of 3′-chloroflavone 4′-*O*-*β*-D-(4′′-*O*-methyl)-glucopyranoside (**2a**).

Figure S57. ^{13}C NMR spectrum expansion (*δ*, acetone-d6, 151 MHz) of 3′-chloroflavone 4′-*O*-*β*-D-(4′′-*O*-methyl)-glucopyranoside (**2a**).
Figure S58. COSY contour map – ^{1}H x ^{1}H of 3′-chloroflavone 4′-*O*-*β*-D-(4′′-*O*-methyl)-glucopyranoside (**2a**).
Figure S59. COSY contour map – ^{1}H x ^{1}H expansion of 3′-chloroflavone 4′-*O*-*β*-D-(4′′-*O*-methyl)-glucopyranoside (**2a**).
Figure S60. COSY contour map – ^{1}H x ^{1}H expansion of 3′-chloroflavone 4′-*O*-*β*-D-(4′′-*O*-methyl)-glucopyranoside (**2a**).
Figure S61. HMQC contour map – ^{1}H x ^{13}C of 3′-chloroflavone 4′-*O*-*β*-D-(4′′-*O*-methyl)-glucopyranoside (**2a**).
Figure S62. HMQC contour map – ^{1}H x ^{13}C expansion of 3′-chloroflavone 4′-*O*-*β*-D-(4′′-*O*-methyl)-glucopyranoside (**2a**).
Figure S63. HMQC contour map – ^{1}H x ^{13}C expansion of 3′-chloroflavone 4′-*O*-*β*-D-(4′′-*O*-methyl)-glucopyranoside (**2a**).
Figure S64. HMBC contour map – ^{1}H x ^{13}C of 3′-chloroflavone 4′-*O*-*β*-D-(4′′-*O*-methyl)-glucopyranoside (**2a**).
Figure S65. HMBC contour map – ^{1}H x ^{13}C expansion of 3′-chloroflavone 4′-*O*-*β*-D-(4′′-*O*-methyl)-glucopyranoside (**2a**).
Figure S66. HMBC contour map – ^{1}H x ^{13}C expansion of 3′-chloroflavone 4′-*O*-*β*-D-(4′′-*O*-methyl)-glucopyranoside (**2a**).
Figure S67. HMBC contour map – ^{1}H x ^{13}C expansion of 3′-chloroflavone 4′-*O*-*β*-D-(4′′-*O*-methyl)-glucopyranoside (**2a**).
Figure S68. 3′-Chloroflavone 4′-*O*-*β*-D-(4′′-*O*-methyl)-glucopyranoside (**2a**) physicochemical and ADME parameters prediction using the SwissADME modelling.

Figure S69. MS analysis of 4′-chloroflavone (**3**).
Figure S70. HPLC analysis of 4′-chloroflavone (**3**).
Figure S71. ^{1}H NMR spectrum (*δ*, acetone-d6, 600 MHz) of 4′-chloroflavone (**3**).
Figure S72. ^{1}H NMR spectrum expansion (*δ*, acetone-d6, 600 MHz) of 4′-chloroflavone (**3**).
Figure S73. ^{13}C NMR spectrum (*δ*, acetone-d6, 151 MHz) of 4′-chloroflavone (**3**).
Figure S74. ^{13}C NMR spectrum expansion (*δ*, acetone-d6, 151 MHz) of 4′-chloroflavone (**3**).
Figure S75. ^{13}C NMR spectrum expansion (*δ*, acetone-d6, 151 MHz) of 4′-chloroflavone (**3**).
Figure S76. COSY contour map – ^{1}H x ^{1}H of 4′-chloroflavone (**3**).
Figure S77. COSY contour map – ^{1}H x ^{1}H expansion of 4′-chloroflavone (**3**).
Figure S78. HMQC contour map – ^{1}H x ^{13}C of 4′-chloroflavone (**3**).
Figure S79. HMQC contour map – ^{1}H x ^{13}C expansion of 4′-chloroflavone (**3**).
Figure S80. HMBC contour map – ^{1}H x ^{13}C of 4′-chloroflavone (**3**).
Figure S81. HMBC contour map – ^{1}H x ^{13}C expansion of 4′-chloroflavone (**3**).
Figure S82. HMBC contour map – ^{1}H x ^{13}C expansion of 4′-chloroflavone (**3**).
Figure S83. 4′-Chloroflavone (**3**) physicochemical and ADME parameters prediction using the SwissADME modelling.

Figure S84. MS analysis of 6-chloroflavone (**4**).
Figure S85. HPLC analysis of 6-chloroflavone (**4**).
Figure S86. ^{1}H NMR spectrum (*δ*, acetone-d6, 600 MHz) of 6-chloroflavone (**4**).
Figure S87. ^{1}H NMR spectrum expansion (*δ*, acetone-d6, 600 MHz) of 6-chloroflavone (**4**).
Figure S88. ^{13}C NMR spectrum (*δ*, acetone-d6, 151 MHz) of 6-chloroflavone (**4**).
Figure S89. ^{13}C NMR spectrum expansion (*δ*, acetone-d6, 151 MHz) of 6-chloroflavone (**4**).
Figure S90. ^{13}C NMR spectrum expansion (*δ*, acetone-d6, 151 MHz) of 6-chloroflavone (**4**).
Figure S91. COSY contour map – ^{1}H x ^{1}H of 6-chloroflavone (**4**).
Figure S92. COSY contour map – ^{1}H x ^{1}H expansion of 6-chloroflavone (**4**).

Figure S93. HMQC contour map – ^{1}H x ^{13}C of 6-chloroflavone (**4**).
Figure S94. HMQC contour map – ^{1}H x ^{13}C expansion of 6-chloroflavone (**4**).
Figure S95. HMBC contour map – ^{1}H x ^{13}C of 6-chloroflavone (**4**).
Figure S96. HMBC contour map – ^{1}H x ^{13}C expansion of 6-chloroflavone (**4**).
Figure S97. HMBC contour map – ^{1}H x ^{13}C expansion of 6-chloroflavone (**4**).
Figure S98. 6-Chloroflavone (**4**) physicochemical and ADME parameters prediction using the SwissADME modelling.

Figure S99. MS analysis of 6-chloroflavone 3′-*O*-*β*-D-(4′′-*O*-methyl)-glucopyranoside (**4a**).
Figure S100. HPLC analysis of 6-chloroflavone 3′-*O*-*β*-D-(4′′-*O*-methyl)-glucopyranoside (**4a**).
Figure S101. ^{1}H NMR spectrum (*δ,* acetone-d6, 600 MHz) of 6-chloroflavone 3′-*O*-*β*-D-(4′′-*O*-methyl)-glucopyranoside (**4a**).
Figure S102. ^{1}H NMR spectrum expansion (*δ,* acetone-d6, 600 MHz) of 6-chloroflavone 3′-*O*-*β*-D-(4′′-*O*-methyl)-glucopyranoside (**4a**).
Figure S103. ^{1}H NMR spectrum expansion (*δ,* acetone-d6, 600 MHz) of 6-chloroflavone 3′-*O*-*β*-D-(4′′-*O*-methyl)-glucopyranoside (**4a**).
Figure S104. ^{13}C NMR spectrum (*δ,* acetone-d6, 151 MHz) of 6-chloroflavone 3′-*O*-*β*-D-(4′′-*O*-methyl)-glucopyranoside (**4a**).
Figure S105. ^{13}C NMR spectrum expansion (*δ,* acetone-d6, 151 MHz) of 6-chloroflavone 3′-*O*-*β*-D-(4′′-*O*-methyl)-glucopyranoside (**4a**).
Figure S106. ^{13}C NMR spectrum expansion (*δ,* acetone-d6, 151 MHz) of 6-chloroflavone 3′-*O*-*β*-D-(4′′-*O*-methyl)-glucopyranoside (**4a**).
Figure S107. COSY contour map – ^{1}H x ^{1}H of 6-chloroflavone 3′-*O*-*β*-D-(4′′-*O*-methyl)-glucopyranoside (**4a**).
Figure S108. COSY contour map – ^{1}H x ^{1}H expansion of 6-chloroflavone 3′-*O*-*β*-D-(4′′-*O*-methyl)-glucopyranoside (**4a**).
Figure S109. COSY contour map – ^{1}H x ^{1}H expansion of 6-chloroflavone 3′-*O*-*β*-D-(4′′-*O*-methyl)-glucopyranoside (**4a**).
Figure S110. HMQC contour map – ^{1}H x ^{13}C of 6-chloroflavone 3′-*O*-*β*-D-(4′′-*O*-methyl)-glucopyranoside (**4a**).
Figure S111. HMQC contour map – ^{1}H x ^{13}C expansion of 6-chloroflavone 3′-*O*-*β*-D-(4′′-*O*-methyl)-glucopyranoside (**4a**).
Figure S112. HMQC contour map – ^{1}H x ^{13}C expansion of 6-chloroflavone 3′-*O*-*β*-D-(4′′-*O*-methyl)-glucopyranoside (**4a**).
Figure S113. HMBC contour map – ^{1}H x ^{13}C of 6-chloroflavone 3′-*O*-*β*-D-(4′′-*O*-methyl)-glucopyranoside (**4a**).
Figure S114. HMBC contour map – ^{1}H x ^{13}C expansion of 6-chloroflavone 3′-*O*-*β*-D-(4′′-*O*-methyl)-glucopyranoside (**4a**).
Figure S115. HMBC contour map – ^{1}H x ^{13}C expansion of 6-chloroflavone 3′-*O*-*β*-D-(4′′-*O*-methyl)-glucopyranoside (**4a**).
Figure S116. HMBC contour map – ^{1}H x ^{13}C expansion of 6-chloroflavone 3′-*O*-*β*-D-(4′′-*O*-methyl)-glucopyranoside (**4a**).
Figure S117. 6-Chloroflavone 3′-*O*-*β*-D-(4′′-*O*-methyl)-glucopyranoside (**4a**) physicochemical and ADME parameters prediction using the SwissADME modelling.
Figure S118. Flavone (**5**) physicochemical and ADME parameters prediction using the SwissADME modelling.

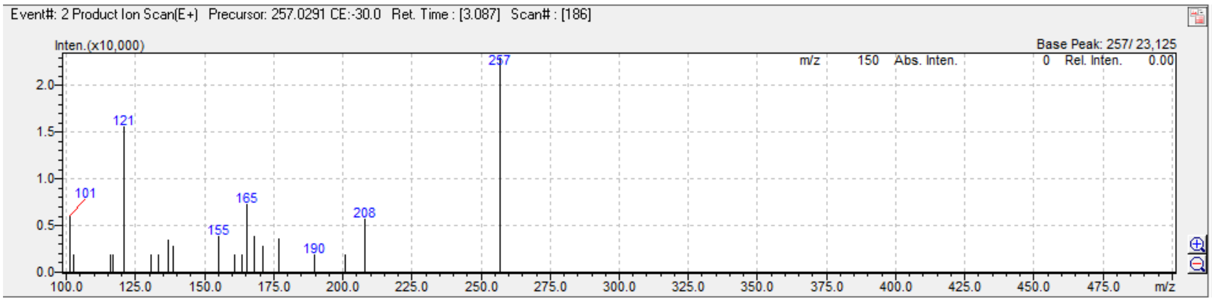

Figure S1. MS analysis of 2′-chloroflavone (**1**).

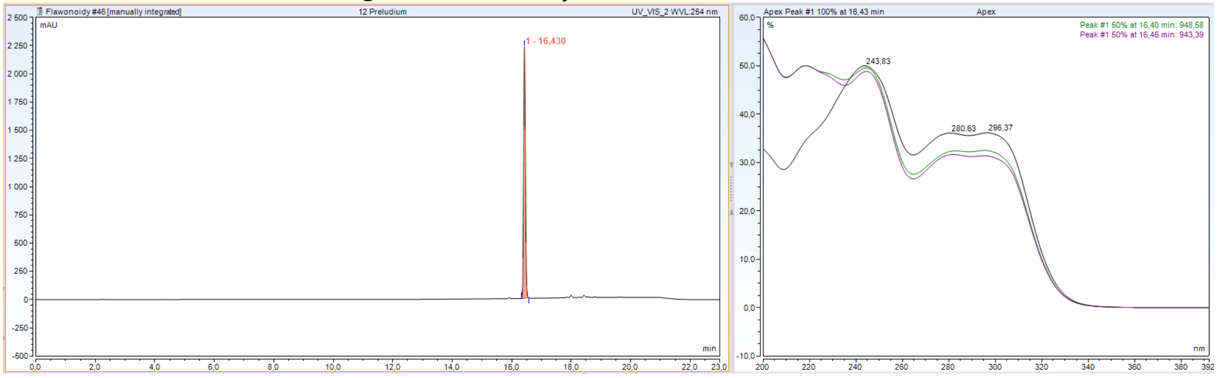

Figure S2. HPLC analysis of 2′-chloroflavone (**1**).

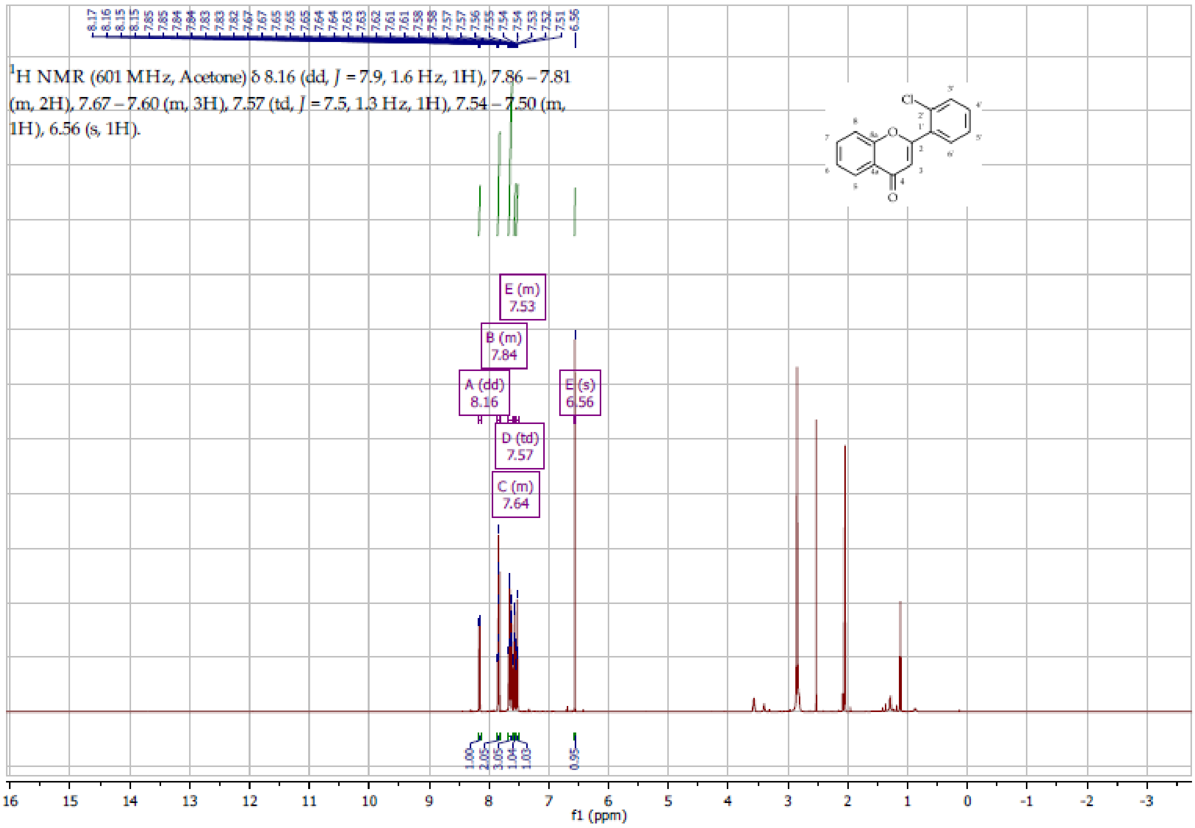

Figure S3. ^{1}H NMR spectrum (δ, acetone-d6, 600 MHz) of 2′-chloroflavone (**1**).

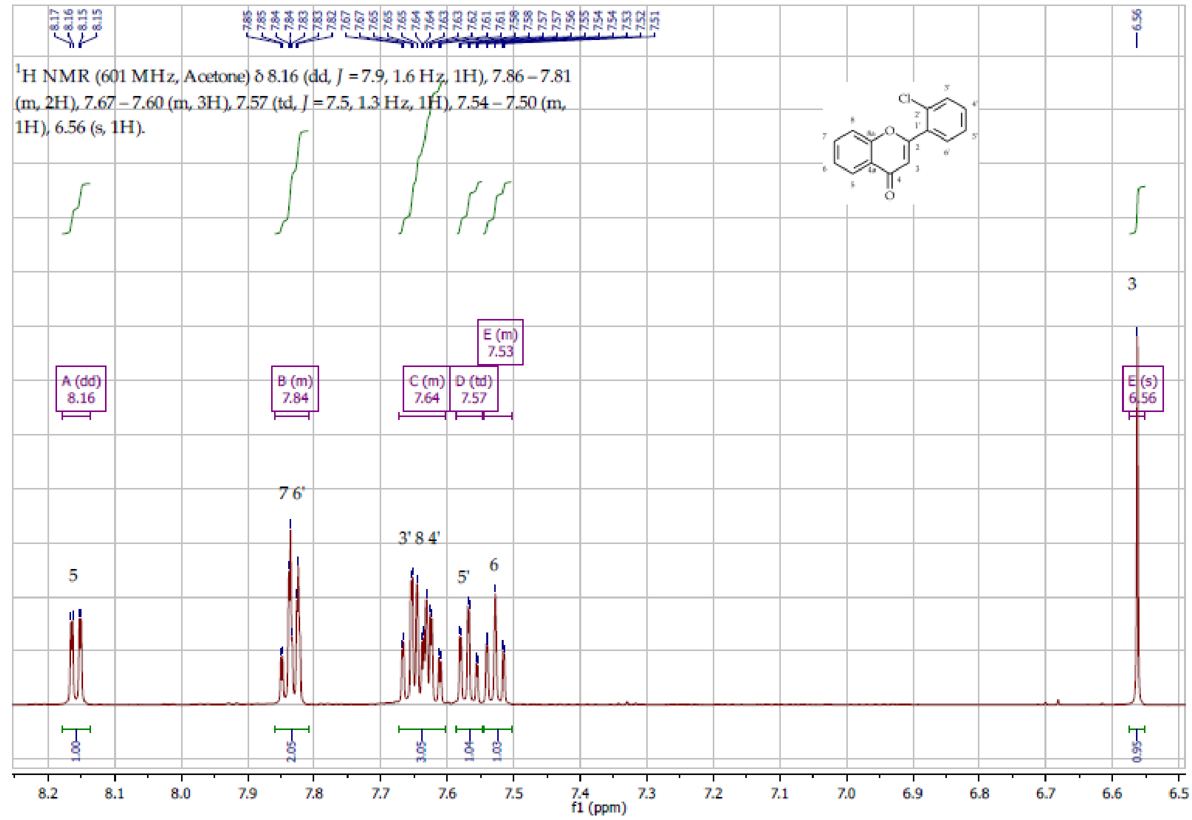

Figure S4 ^{1}H NMR spectrum expansion (*δ*, acetone-d6, 600 MHz) of 2′-chloroflavone (**1**).

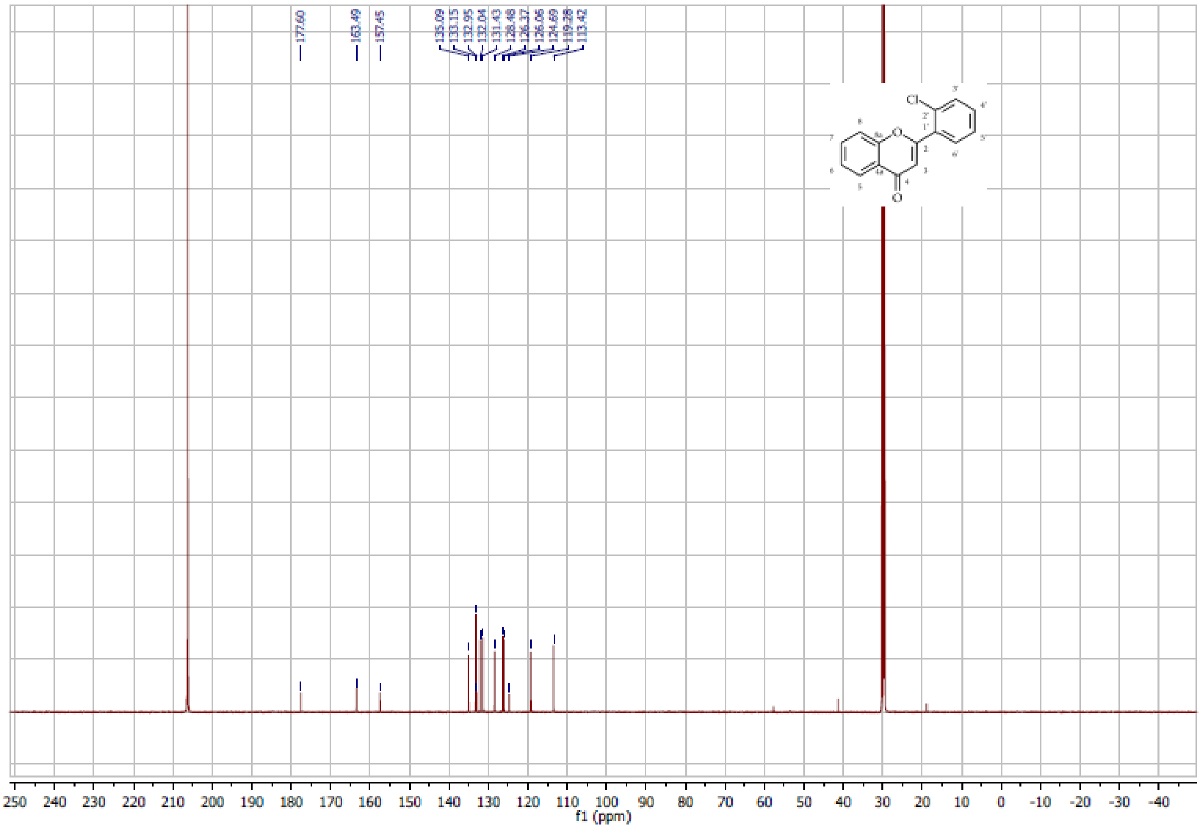

Figure S5. ^{13}C NMR spectrum (*δ*, acetone-d6, 151 MHz) of 2′-chloroflavone (**1**).

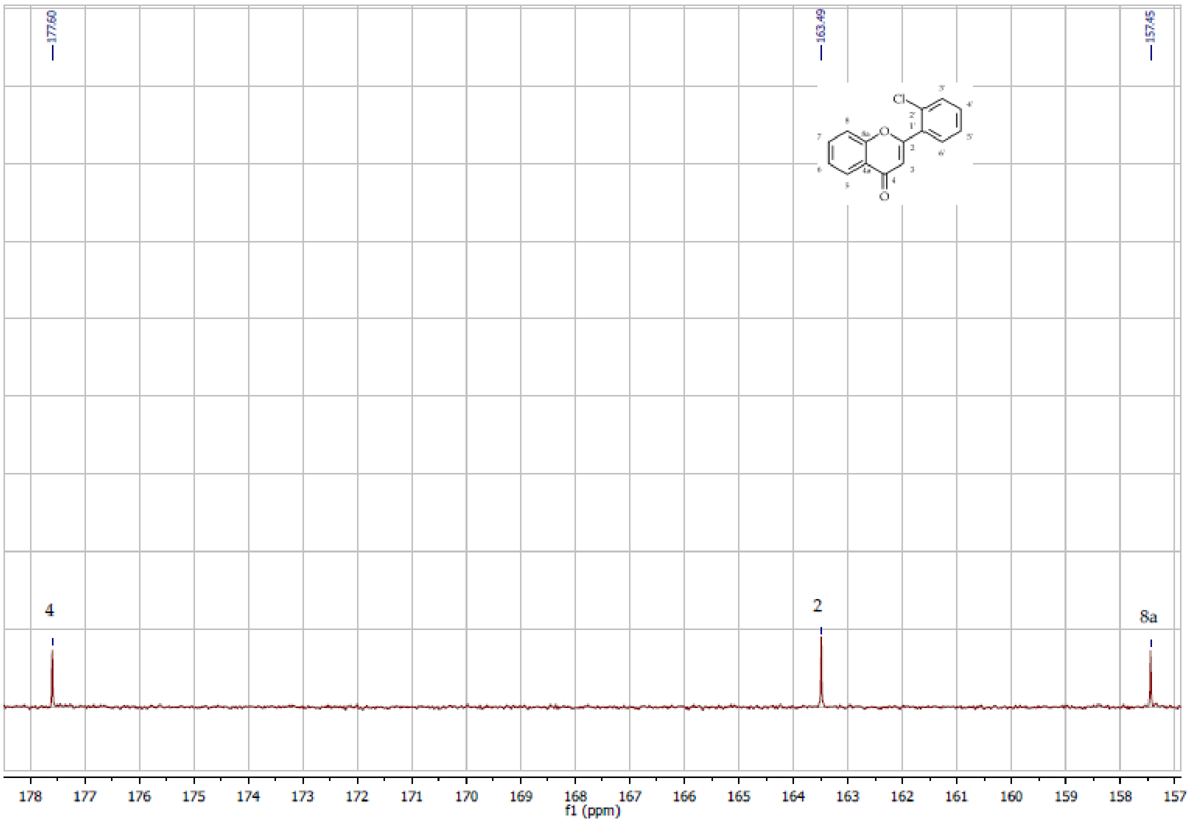

Figure S6. ^{13}C NMR spectrum expansion (δ, acetone-d6, 151 MHz) of 2′-chloroflavone (**1**).

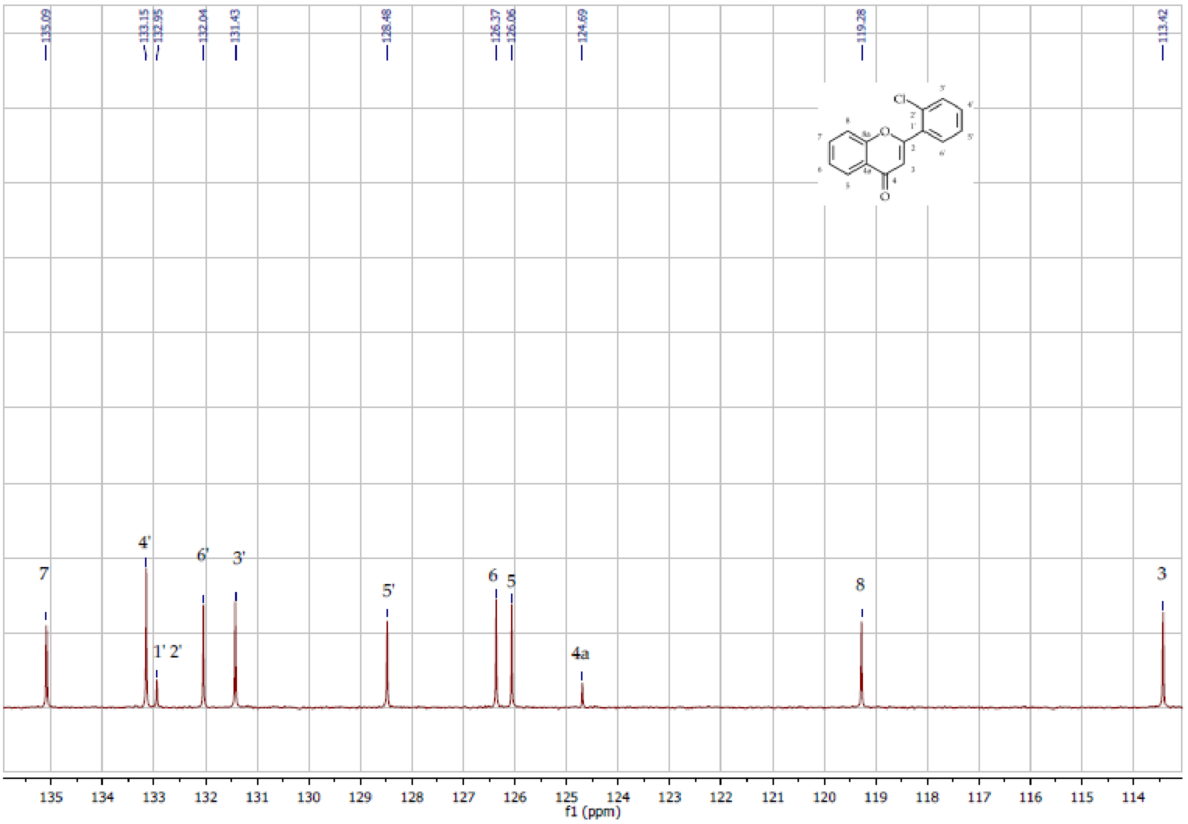

Figure S7. ^{13}C NMR spectrum expansion (δ, acetone-d6, 151 MHz) of 2′-chloroflavone (**1**).

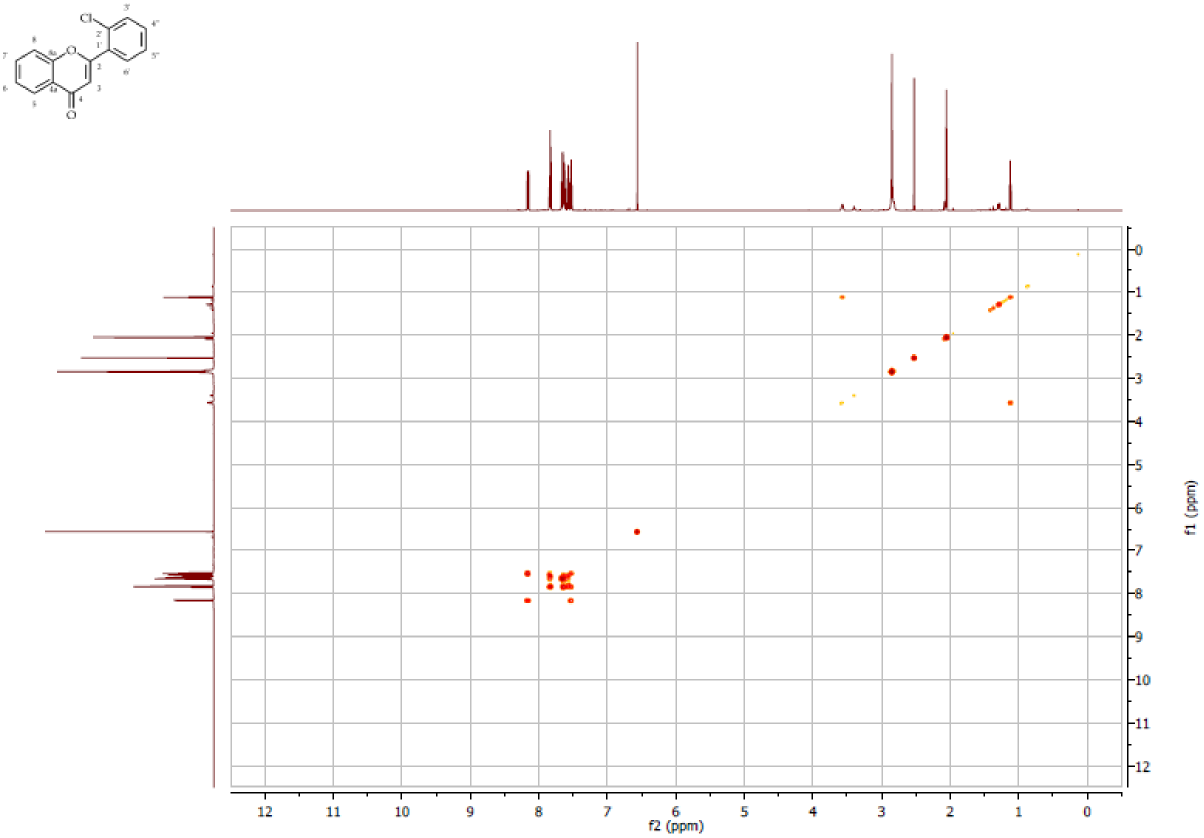

Figure S8. COSY contour map – ^{1}H x ^{1}H of 2′-chloroflavone (**1**).

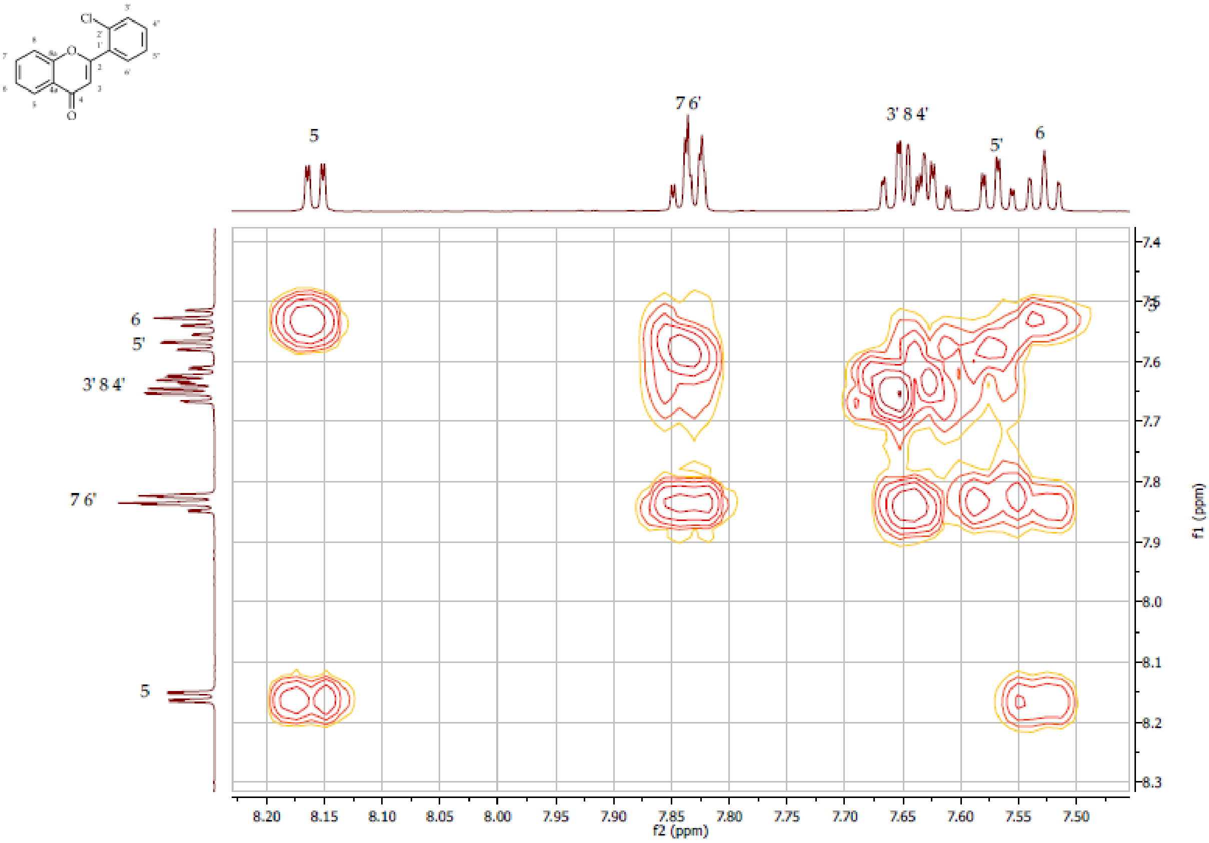

Figure S9. COSY contour map – ^{1}H x ^{1}H expansion of 2′-chloroflavone (**1**).

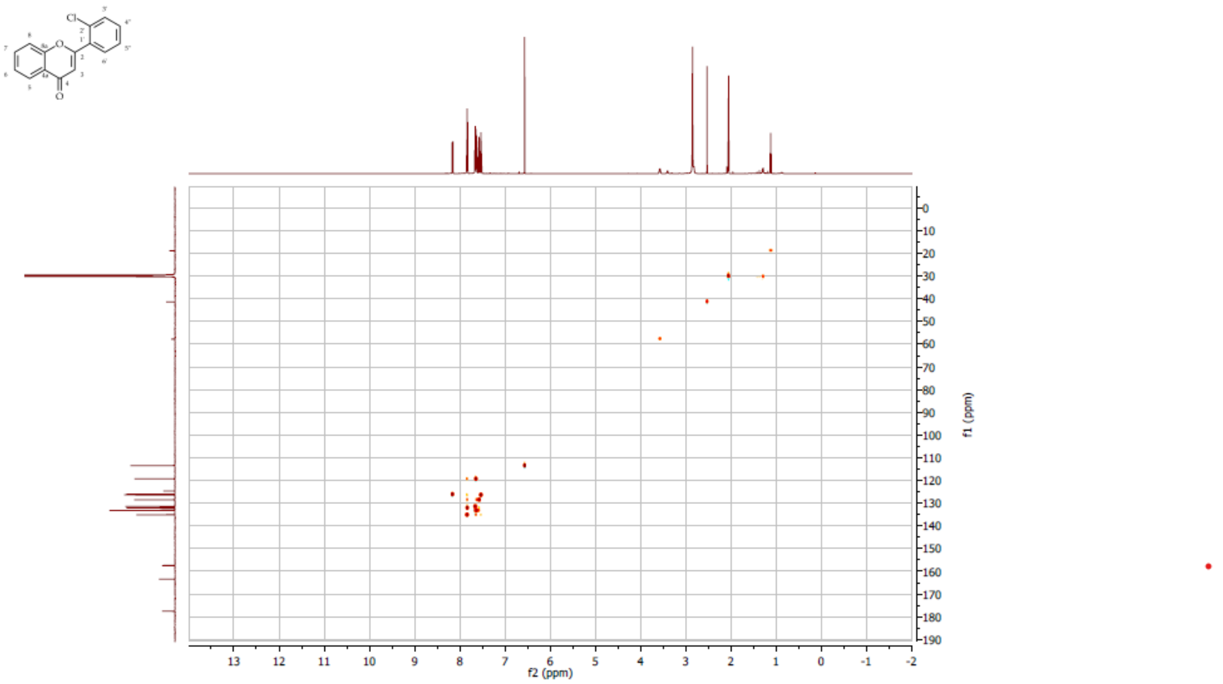

Figure S10. HMQC contour map – ^{1}H x ^{13}C of 2′-chloroflavone (**1**).

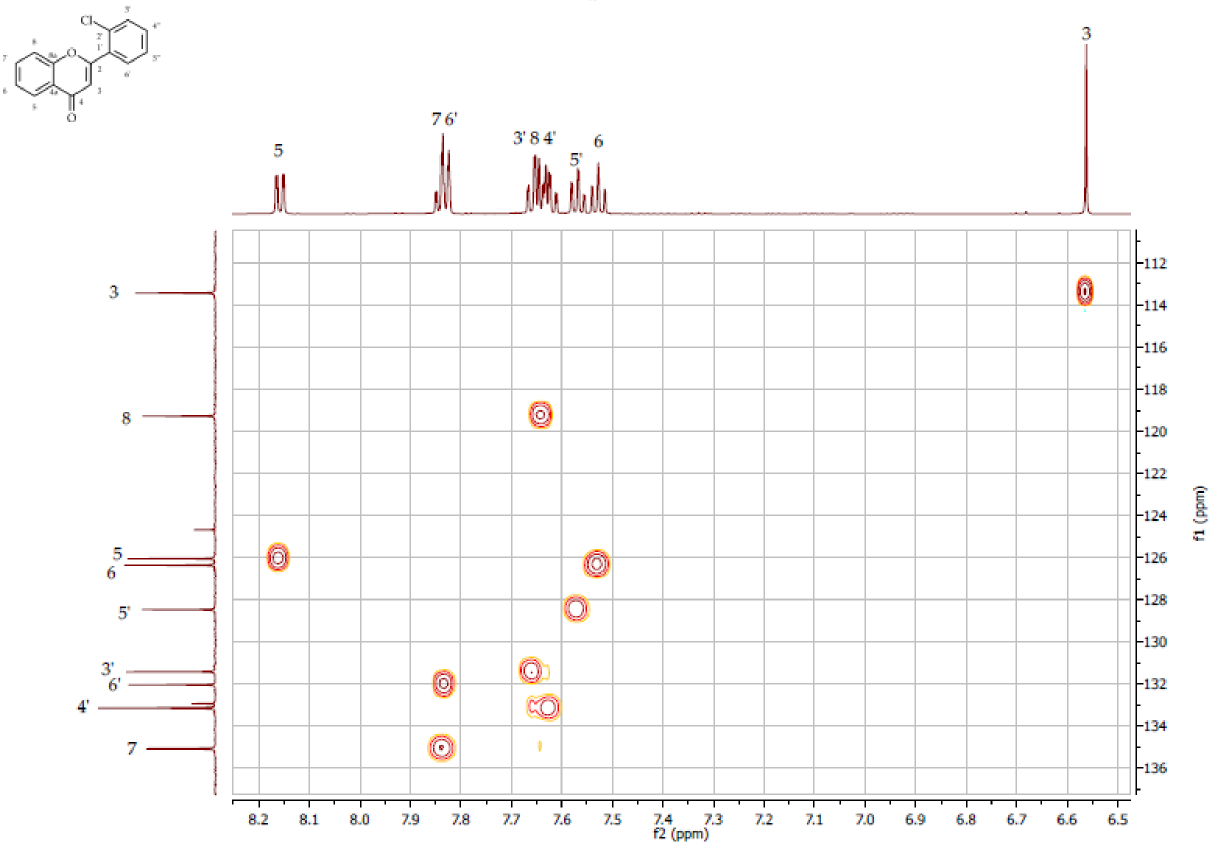

Figure S11. HMQC contour map – ^{1}H x ^{13}C expansion of 2′-chloroflavone (**1**).

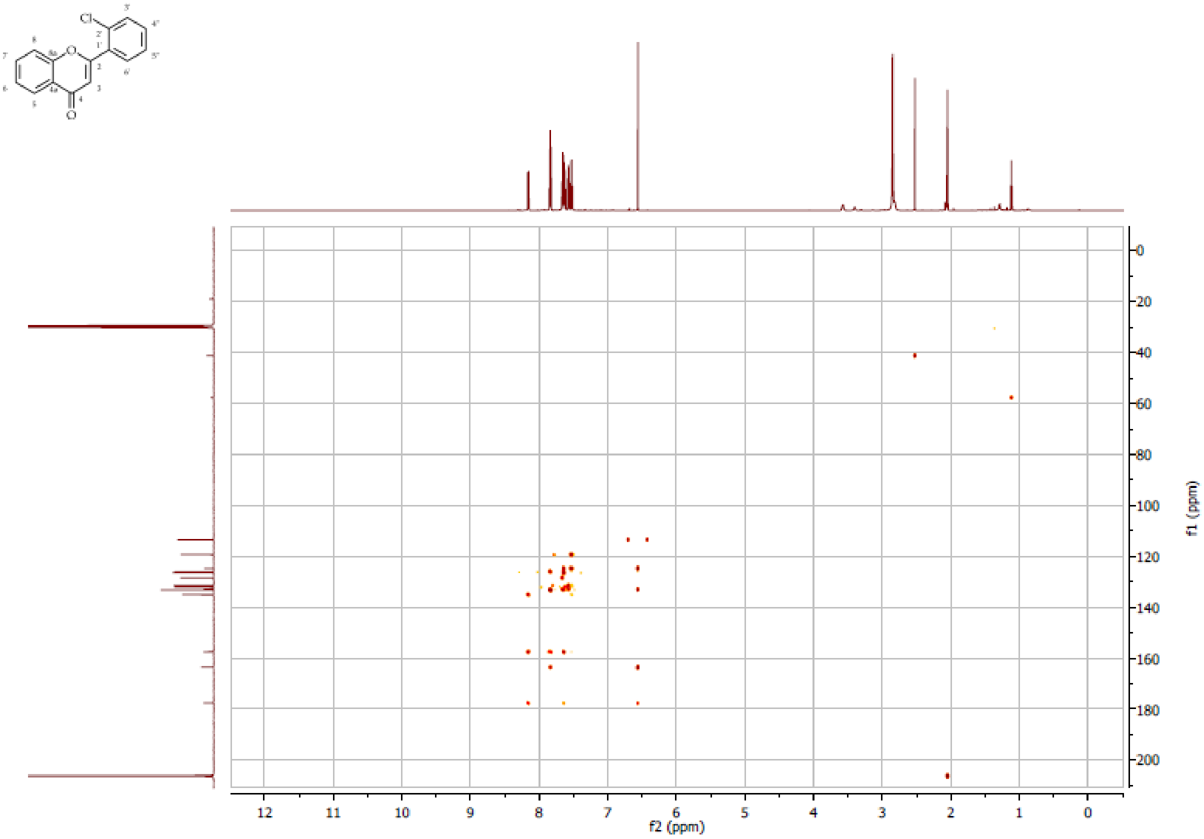

Figure S12. HMBC contour map – ¹H x ¹³C of 2′-chloroflavone (**1**).

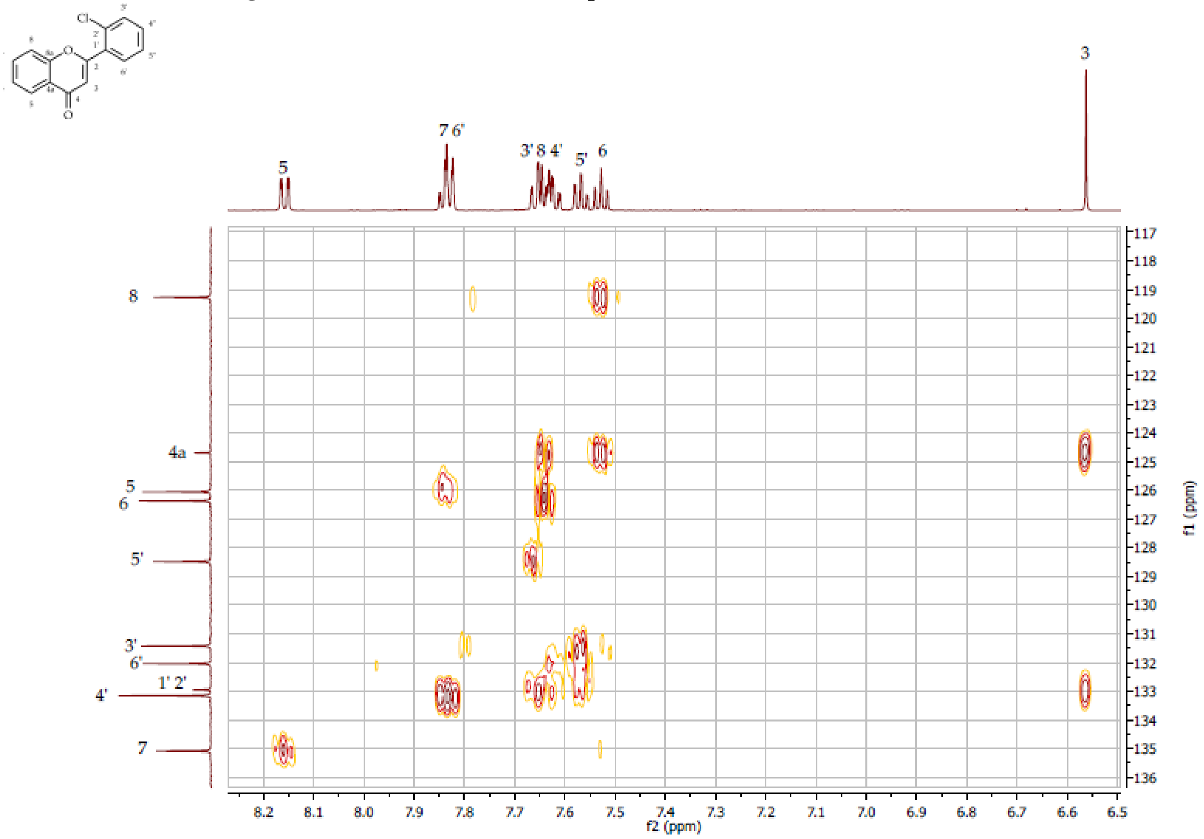

Figure S13. HMBC contour map – ^{1}H x ^{13}C expansion of 2′-chloroflavone (**1**).

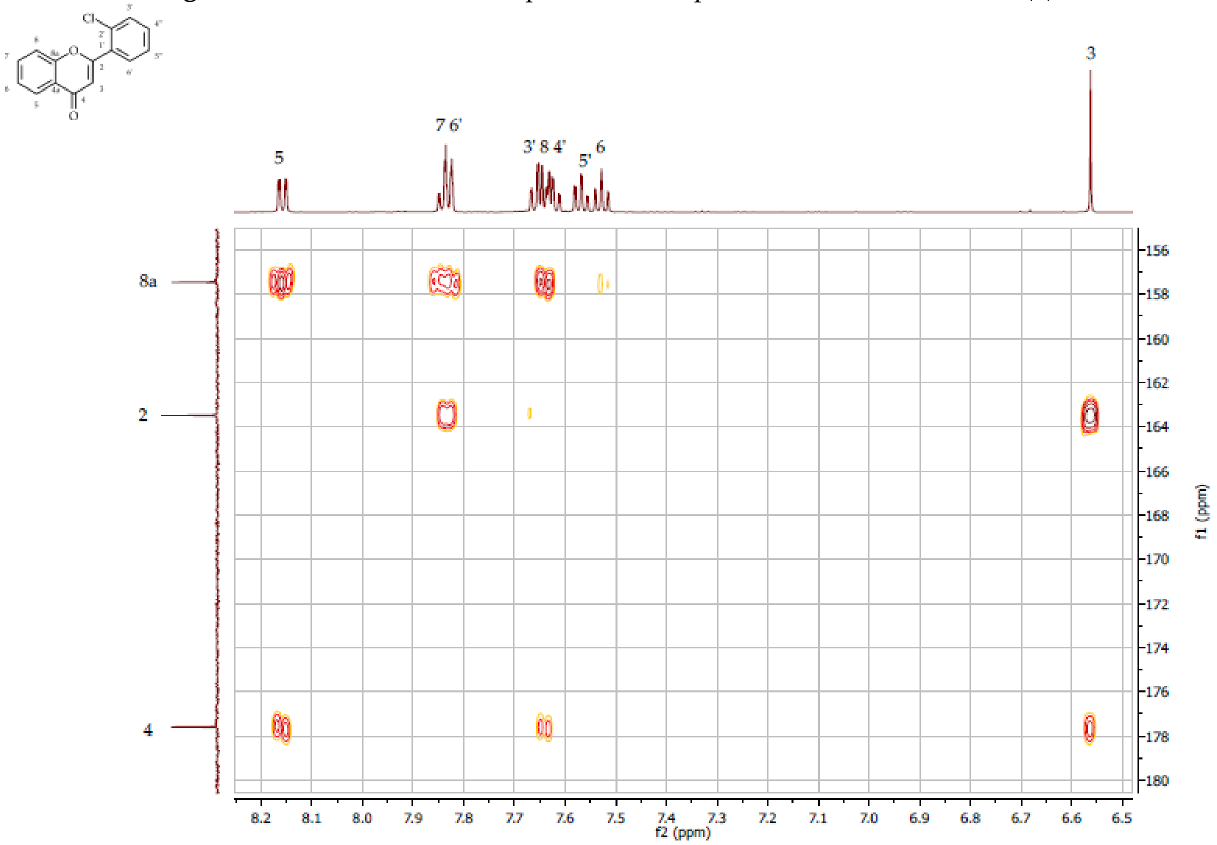

Figure S14. HMBC contour map – ^{1}H x ^{13}C expansion of 2′-chloroflavone (**1**).

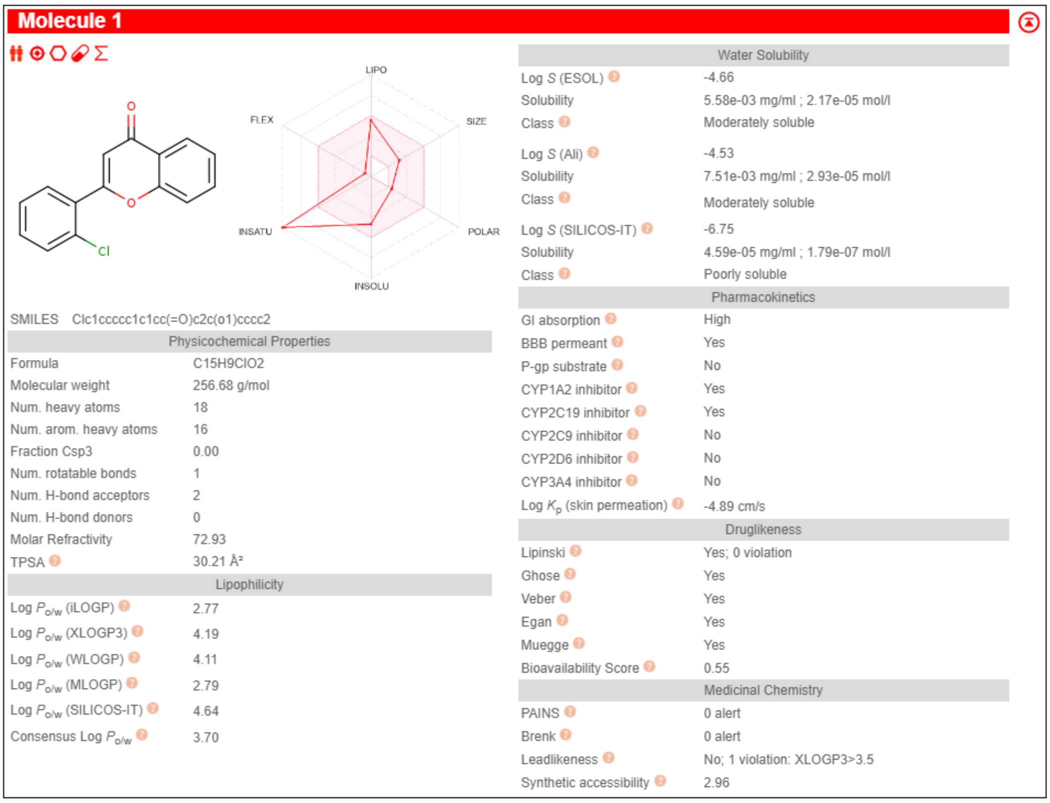

Figure S15. 2′-Chloroflavone (**1**) physicochemical and ADME parameters prediction using the SwissADME modelling.

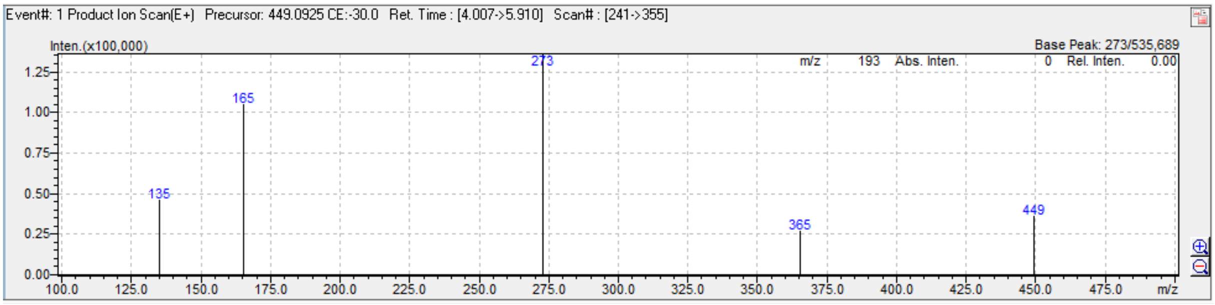

Figure S16. MS analysis of 2′-chloroflavone 3′-*O*-*β*-D-(4′′-*O*-methyl)-glucopyranoside (**1a**).

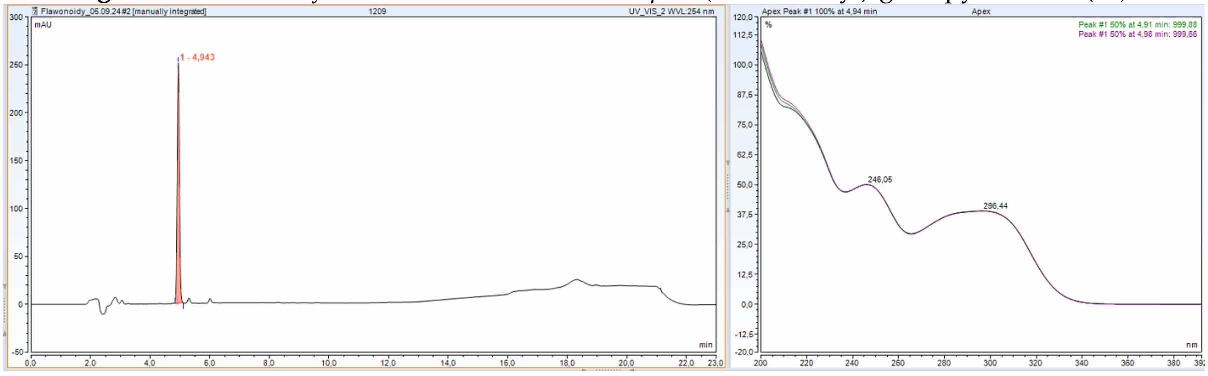

Figure S17. HPLC analysis of 2′-chloroflavone 3′-*O*-*β*-D-(4′′-*O*-methyl)-glucopyranoside (**1a**).

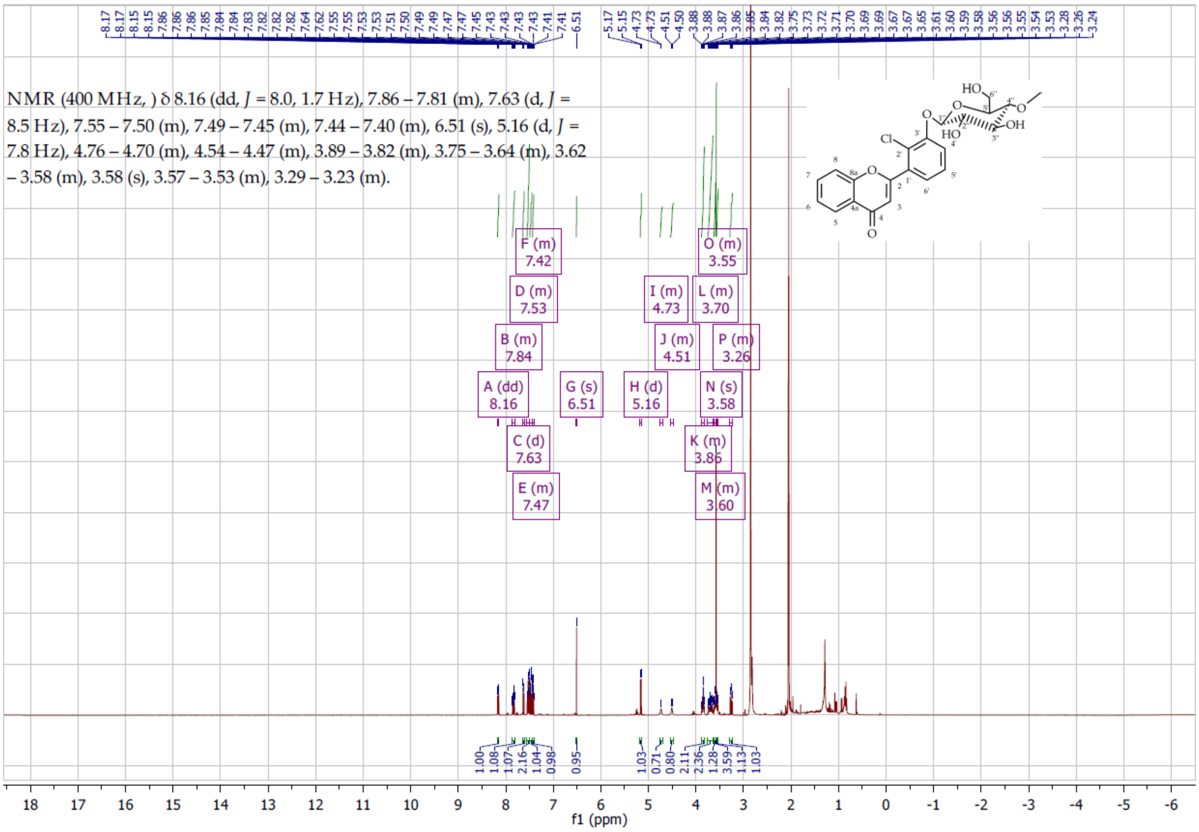

Figure S18. ^{1}H NMR spectrum (*δ*, acetone-d6, 600 MHz) of 2′-chloroflavone 3′-*O*-*β*-D-(4′′-*O*-methyl)-glucopyranoside (**1a**).

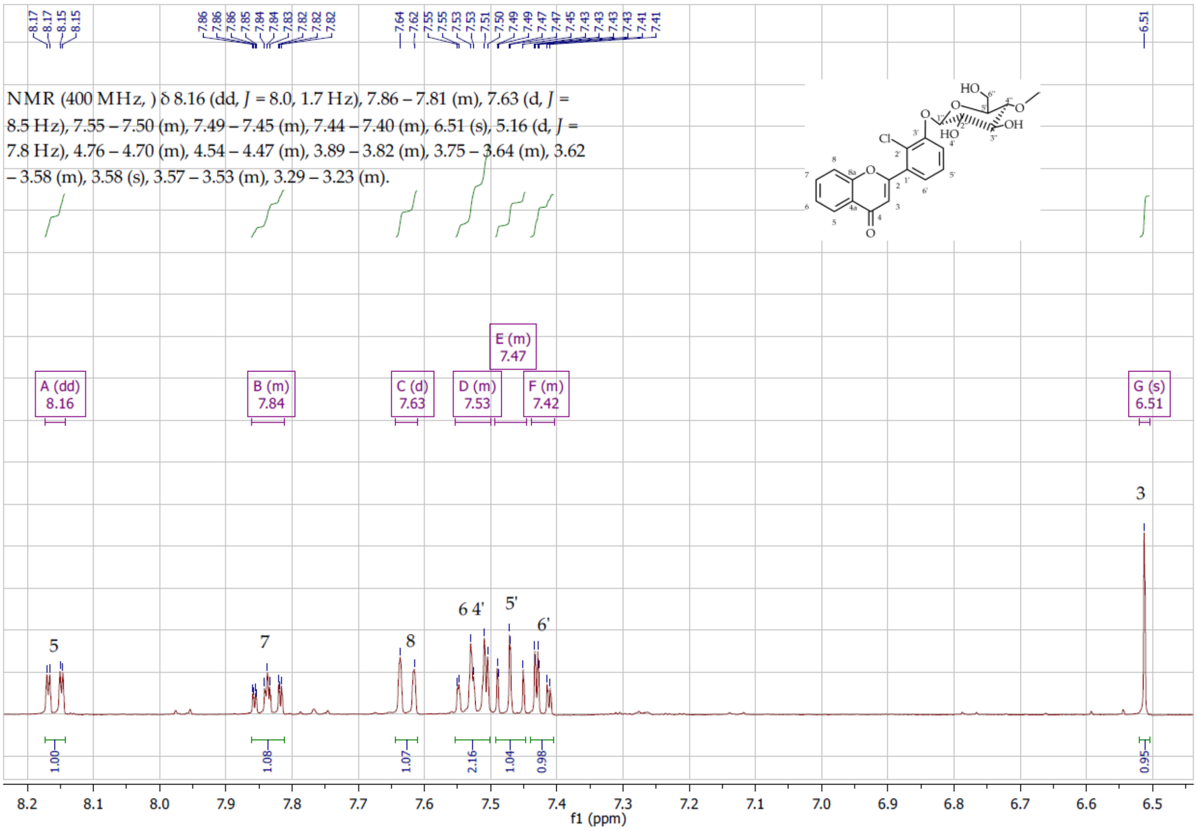

Figure S19. ^{1}H NMR spectrum expansion (*δ*, acetone-d6, 600 MHz) of 2′-chloroflavone 3′-*O*-*β*-D-(4′′-*O*-methyl)-glucopyranoside (**1a**).

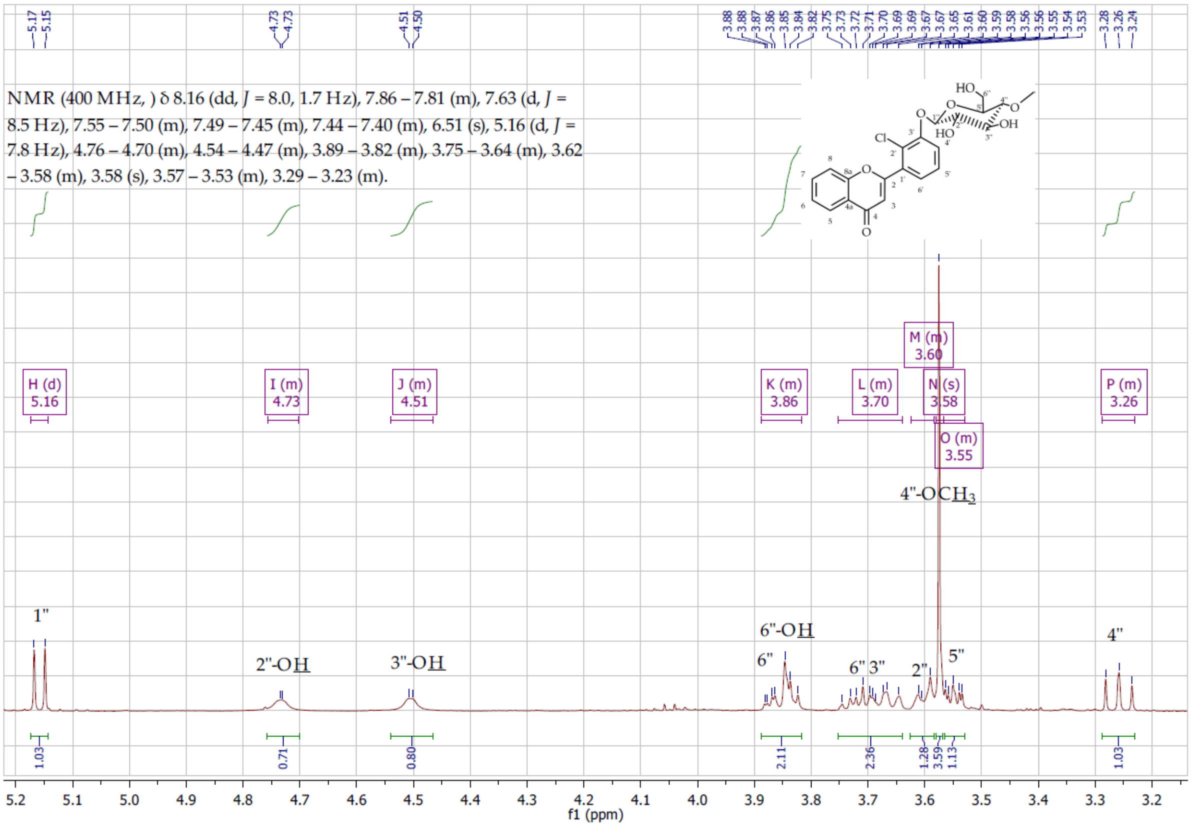

Figure S20. ^{1}H NMR spectrum expansion (*δ*, acetone-d6, 600 MHz) of 2′-chloroflavone 3′-*O*-*β*-D-(4′′-*O*-methyl)-glucopyranoside (**1a**).

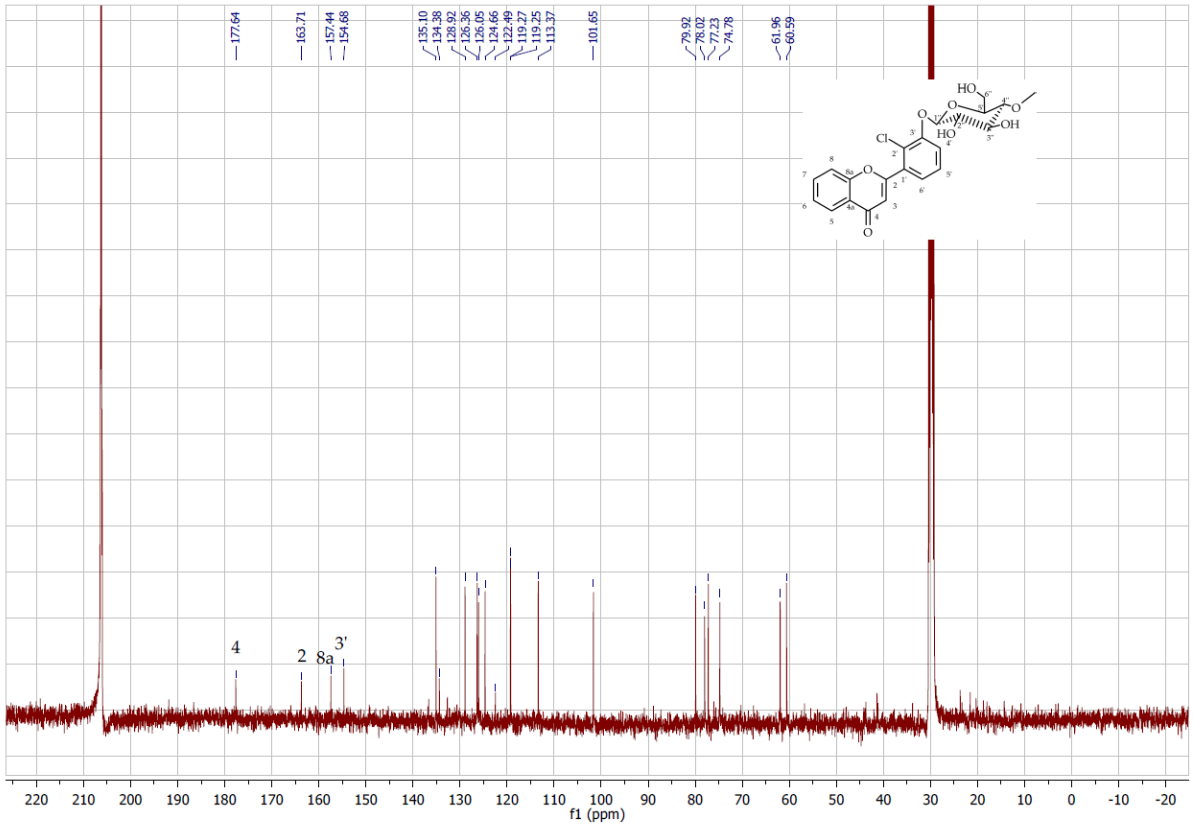

Figure S21. ^{13}C NMR spectrum (*δ*, acetone-d6, 151 MHz) of 2′-chloroflavone 3′-*O*-*β*-D-(4′′-*O*-methyl)-glucopyranoside (**1a**).

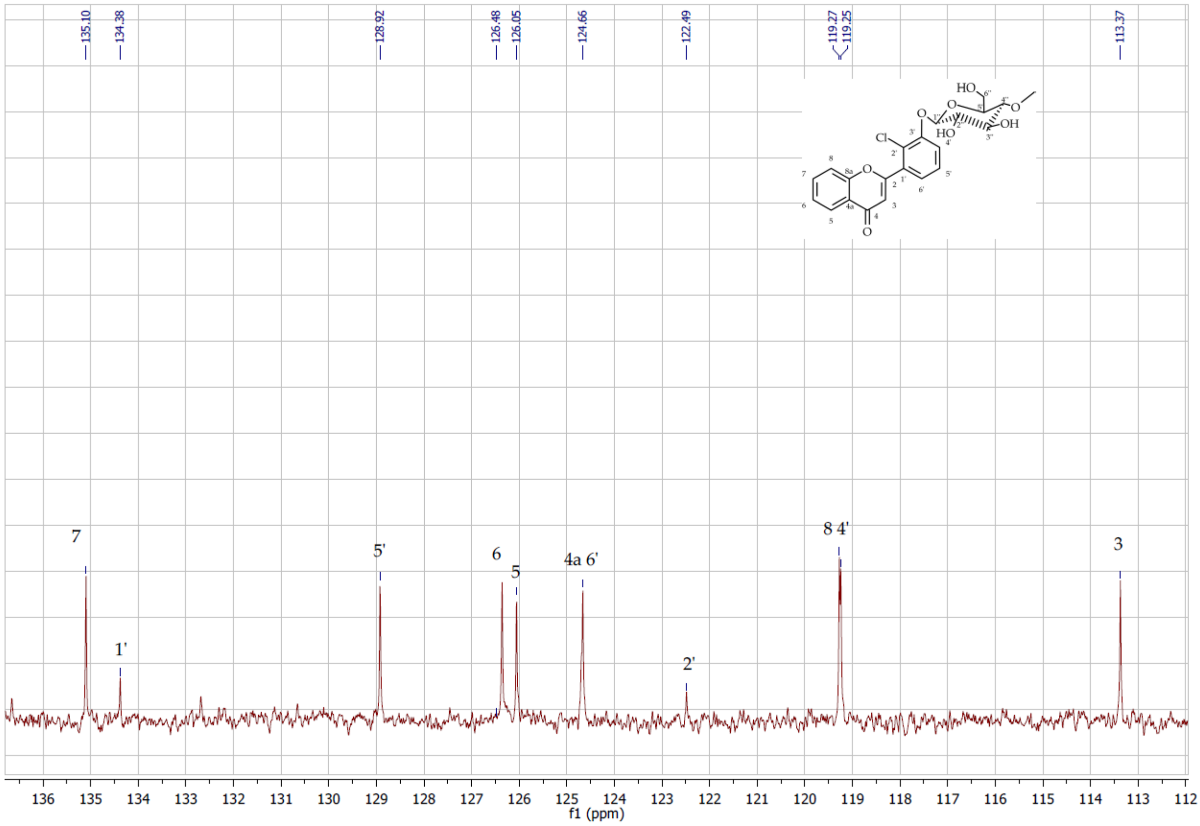

Figure S22. ^{13}C NMR spectrum expansion (*δ*, acetone-d6, 151 MHz) of 2′-chloroflavone 3′-*O*-*β*-D-(4′′-*O*-methyl)-glucopyranoside (**1a**).

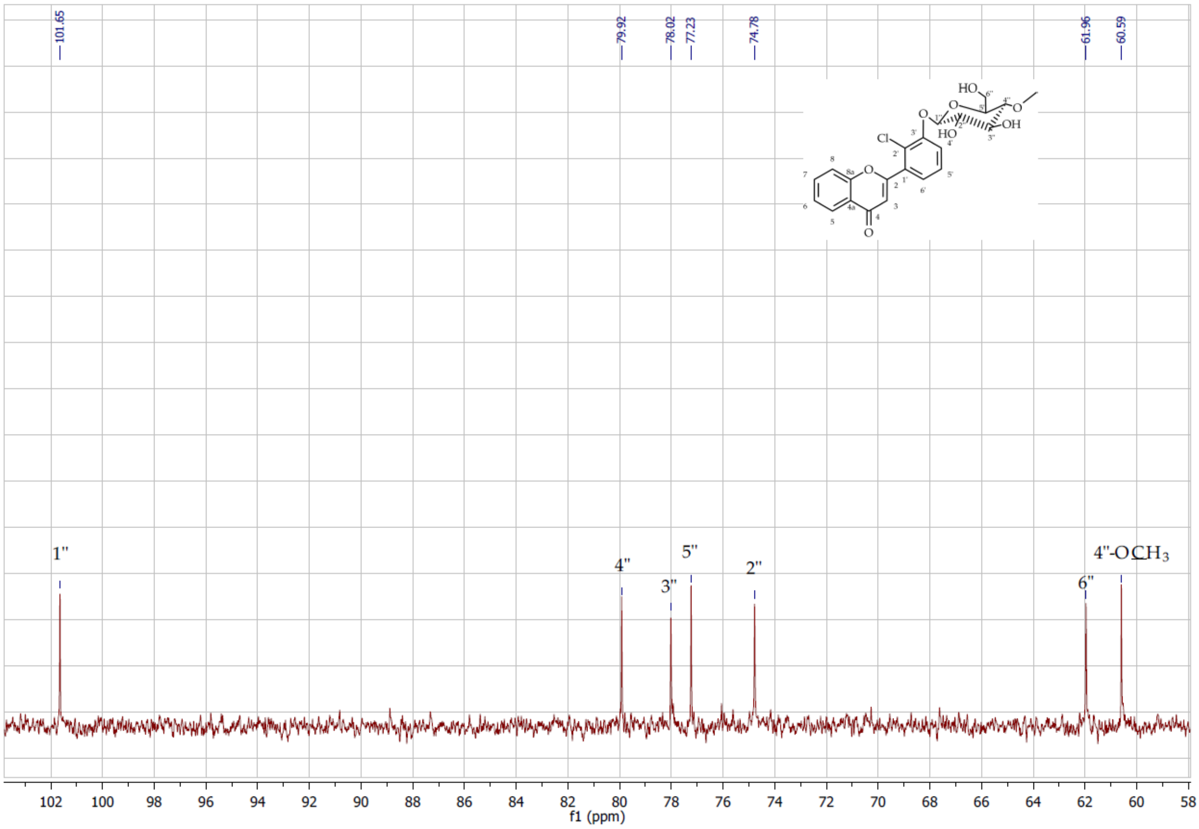

Figure S23. ^{13}C NMR spectrum expansion (*δ*, acetone-d6, 151 MHz) of 2′-chloroflavone 3′-*O*-*β*-D-(4′′-*O*-methyl)-glucopyranoside (**1a**).

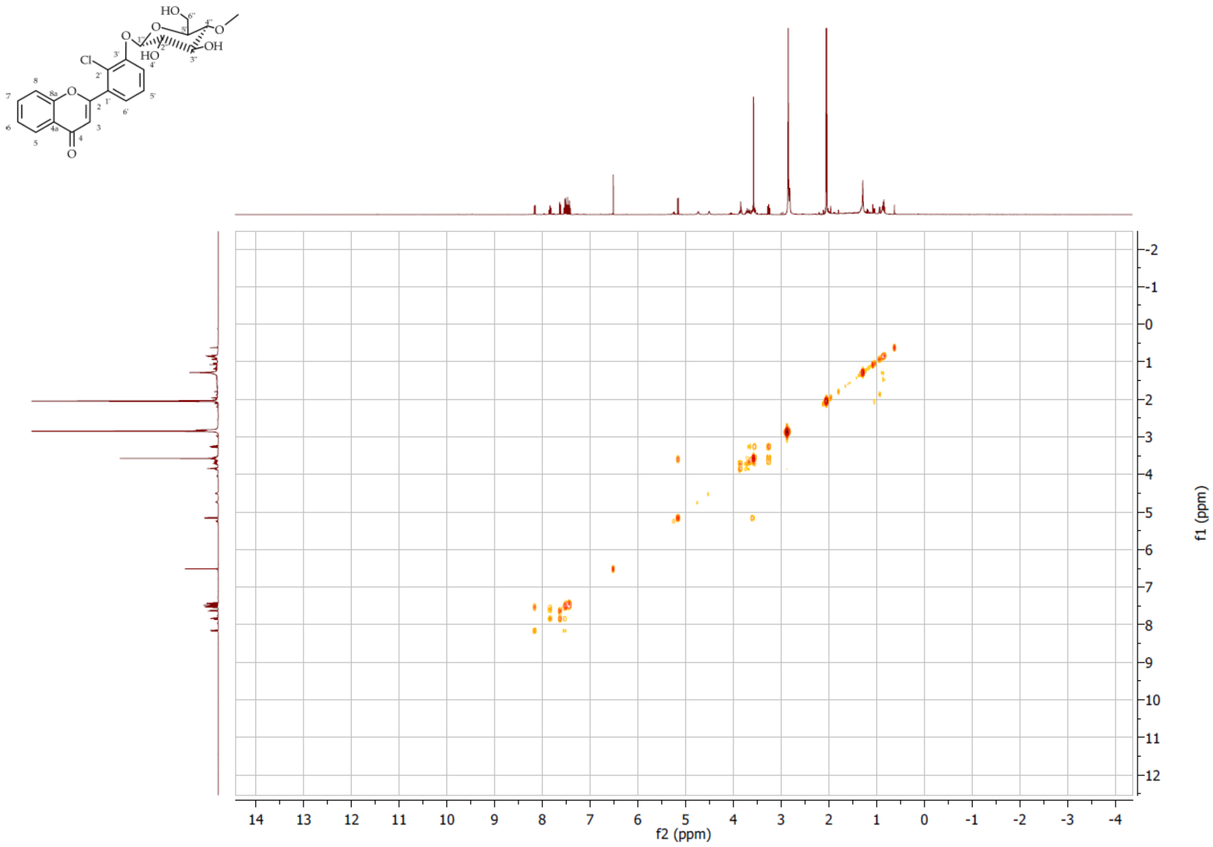

Figure S24. COSY contour map – ^{1}H x ^{1}H of 2′-chloroflavone 3′-*O*-*β*-D-(4′′-*O*-methyl)-glucopyranoside (**1a**).

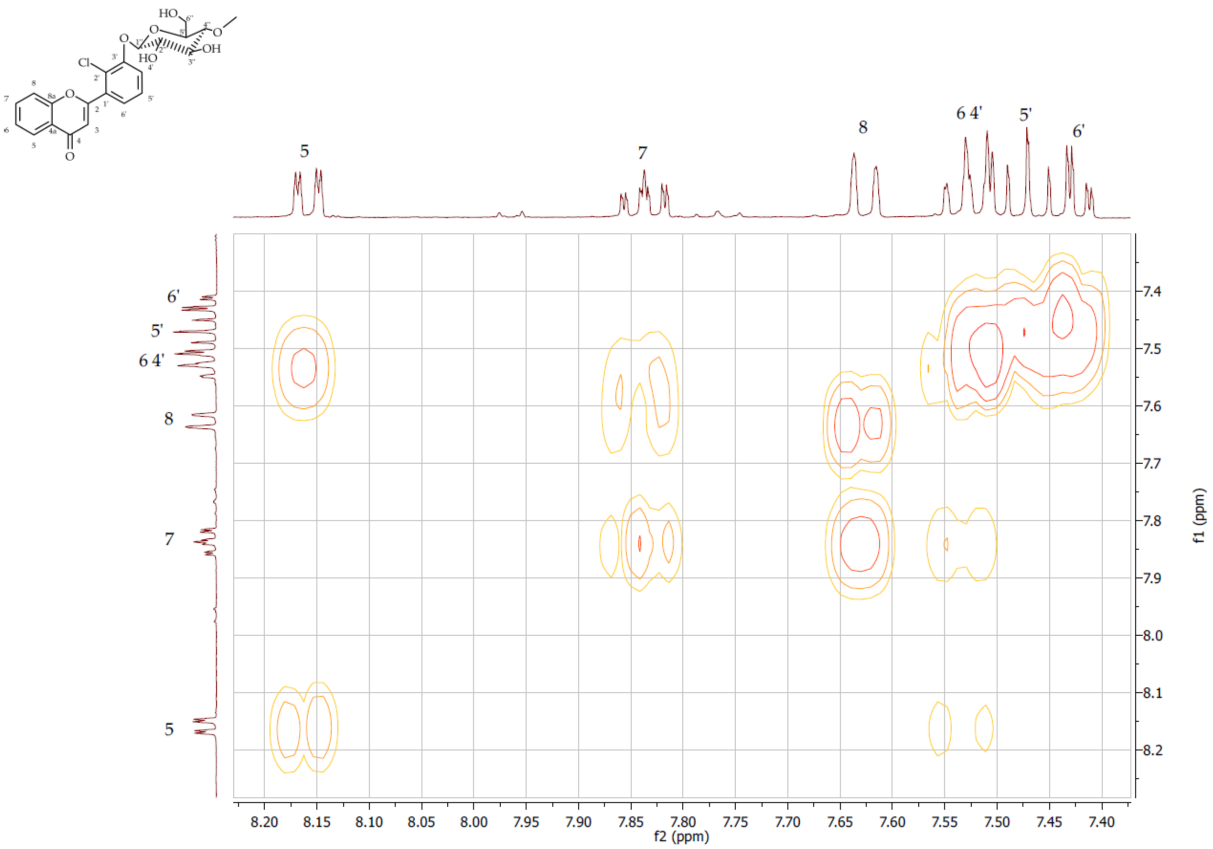

Figure S25. COSY contour map – ^{1}H x ^{1}H expansion of 2′-chloroflavone 3′-*O*-*β*-D-(4′′-*O*-methyl)-glucopyranoside (**1a**).

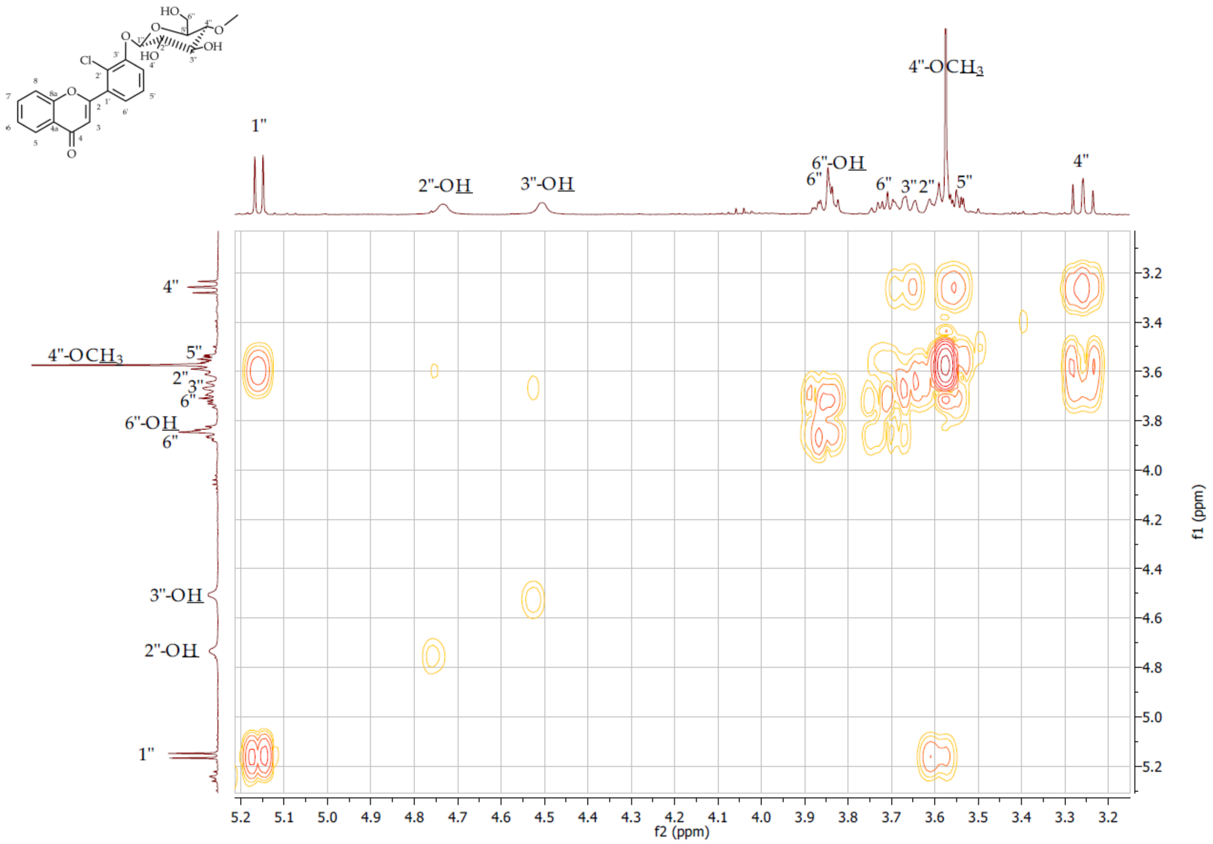

Figure S26. COSY contour map – ^{1}H x ^{1}H expansion of 2′-chloroflavone 3′-*O*-*β*-D-(4′′-*O*-methyl)-glucopyranoside (**1a**).

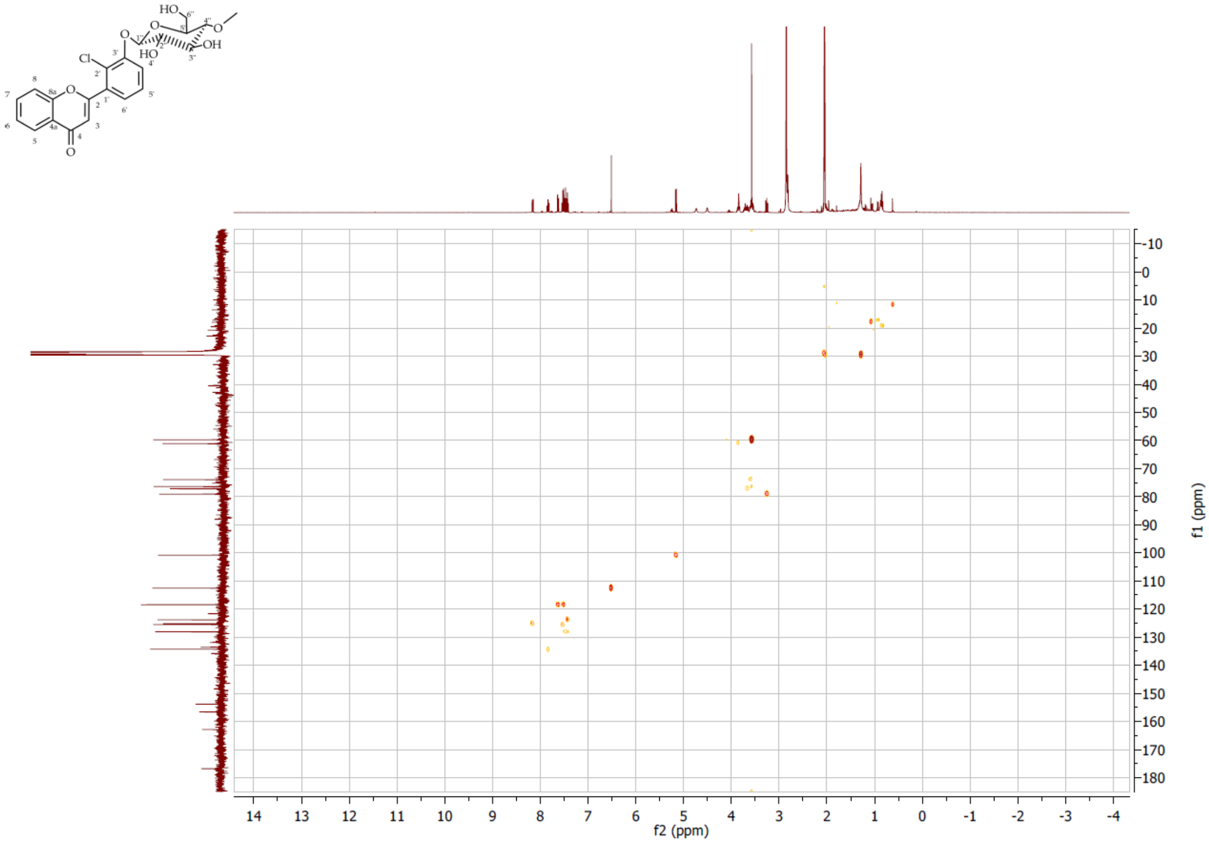

Figure S27. HMQC contour map – ^{1}H x ^{13}C of 2′-chloroflavone 3′-*O*-*β*-D-(4′′-*O*-methyl)-glucopyranoside (**1a**).

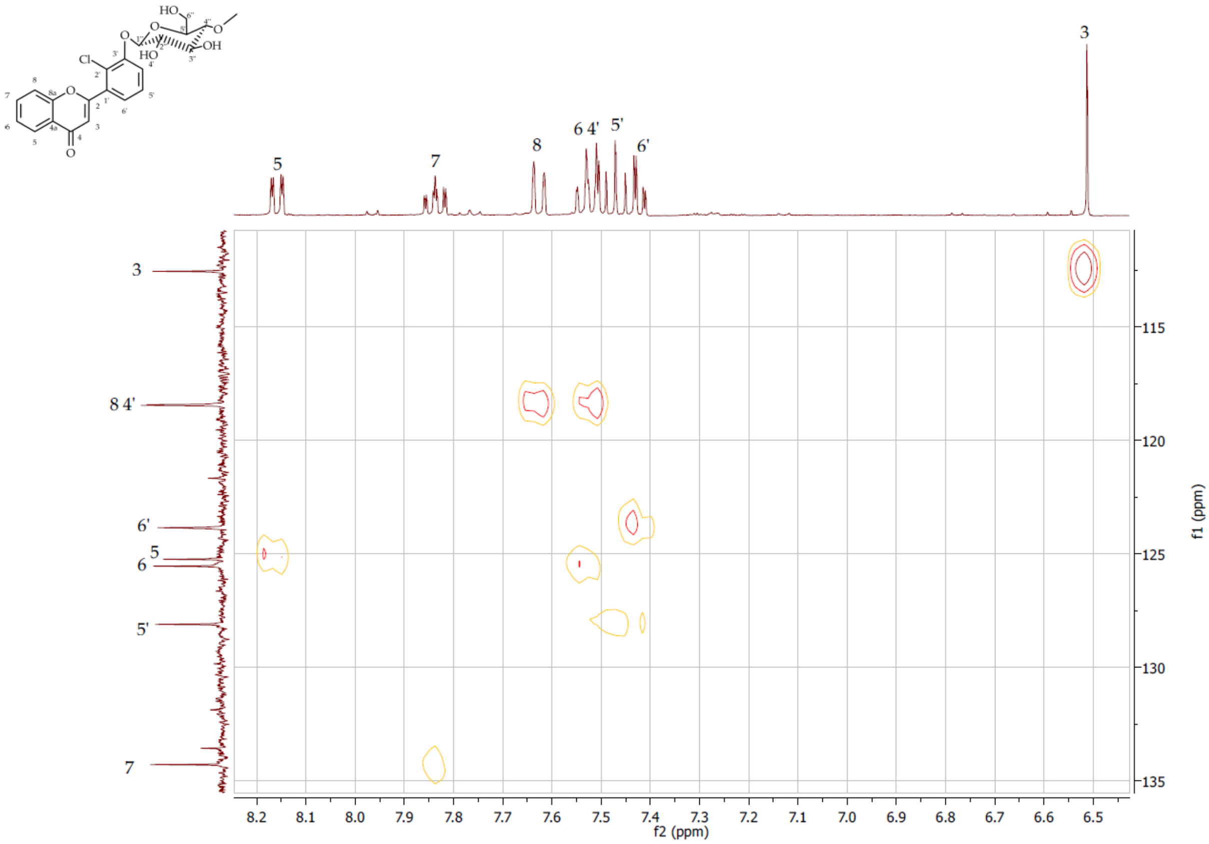

Figure S28. HMQC contour map – ^{1}H x ^{13}C expansion of 2′-chloroflavone 3′-*O*-*β*-D-(4′′-*O*-methyl)-glucopyranoside (**1a**).

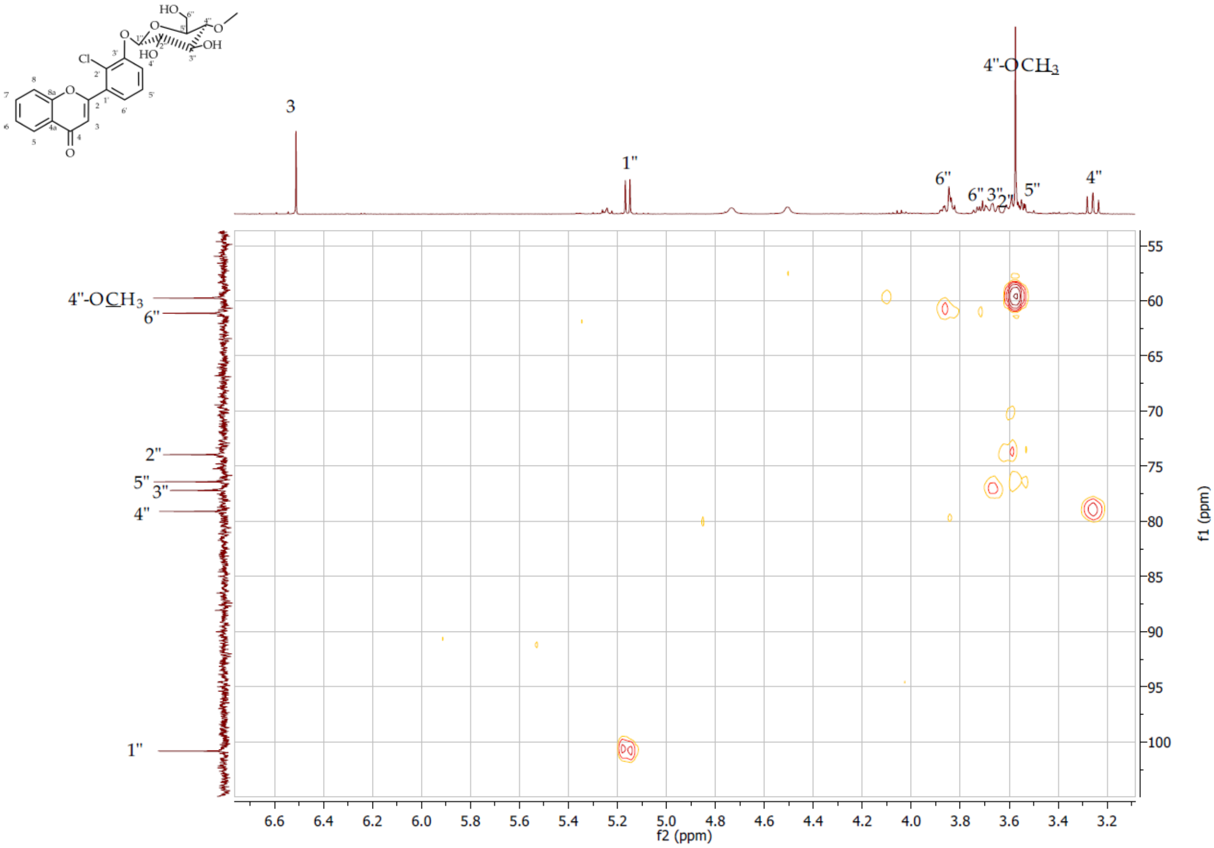

Figure S29. HMQC contour map – [1]H x ^{13}C expansion of 2′-chloroflavone 3′-*O*-*β*-D-(4′′-*O*-methyl)-glucopyranoside (**1a**).

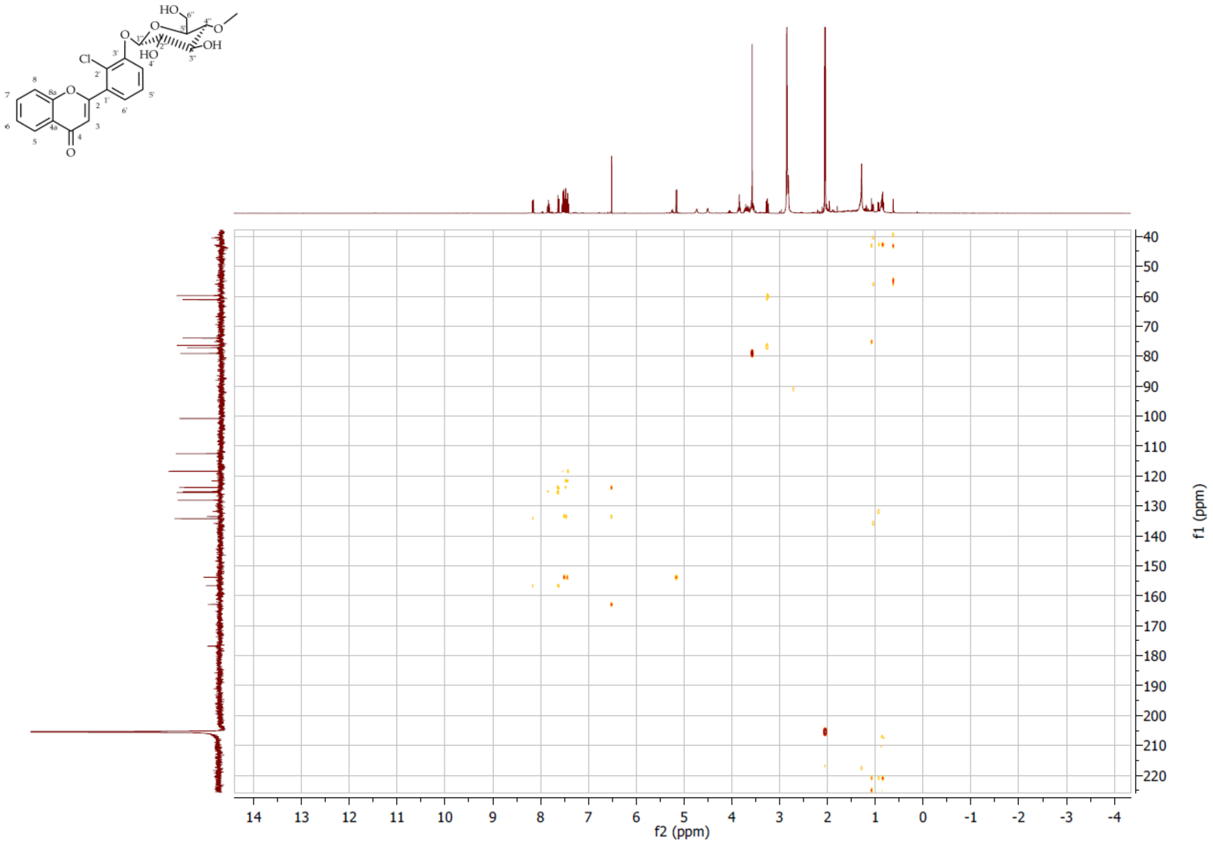

Figure S30. HMBC contour map – ^{1}H x ^{13}C of 2′-chloroflavone 3′-*O*-*β*-D-(4′′-*O*-methyl)-glucopyranoside (**1a**).

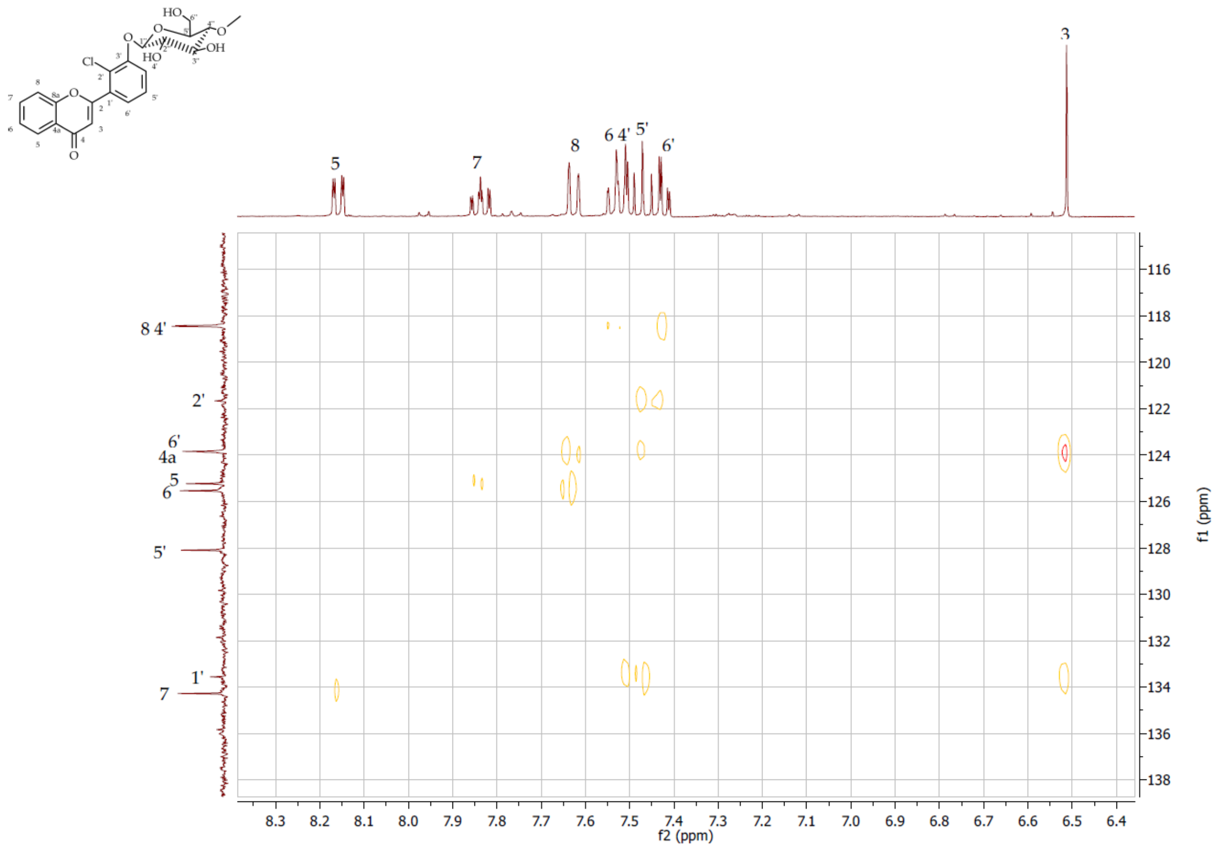

Figure S31. HMBC contour map – ¹H x ¹³C expansion of 2′-chloroflavone 3′-*O*-*β*-D-(4′′-*O*-methyl)-glucopyranoside (**1a**).

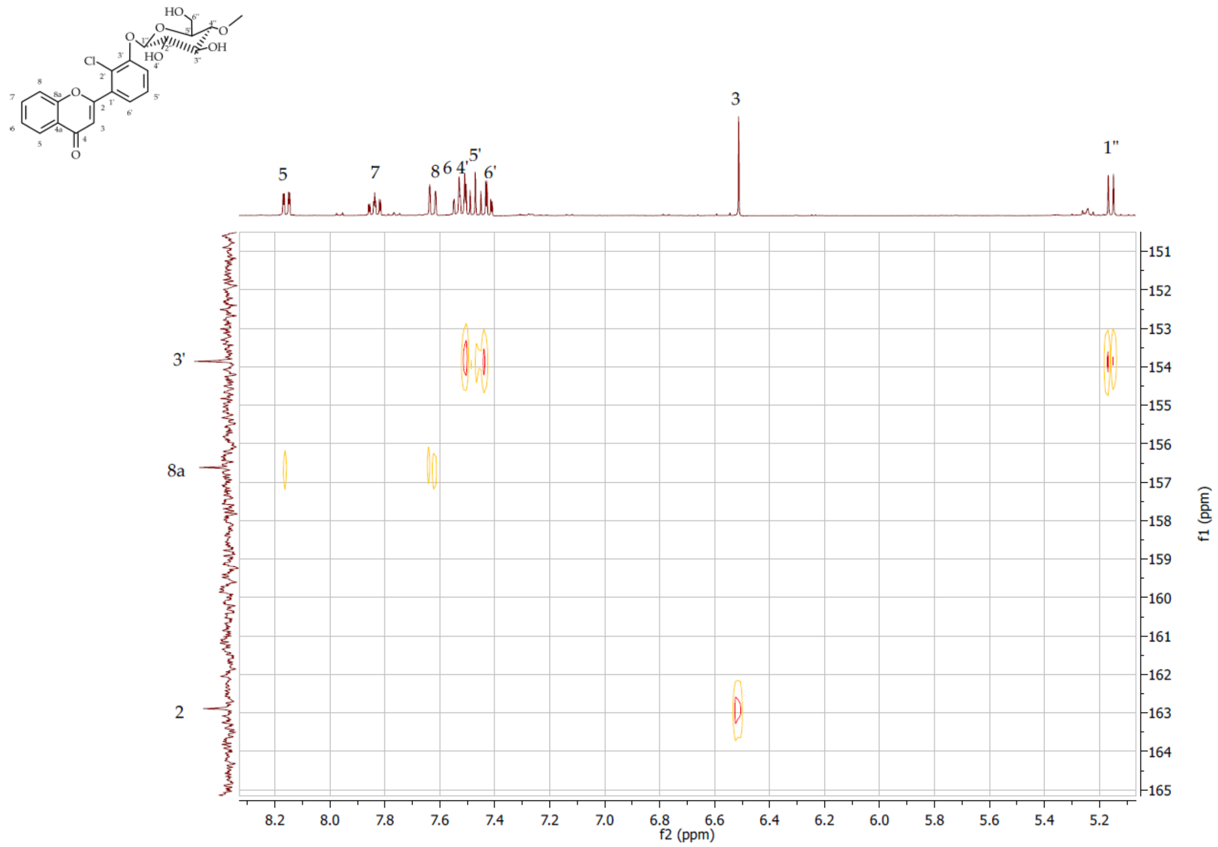

Figure S32. HMBC contour map – ¹H x ¹³C expansion of 2′-chloroflavone 3′-*O*-*β*-D-(4′′-*O*-methyl)-glucopyranoside (**1a**).

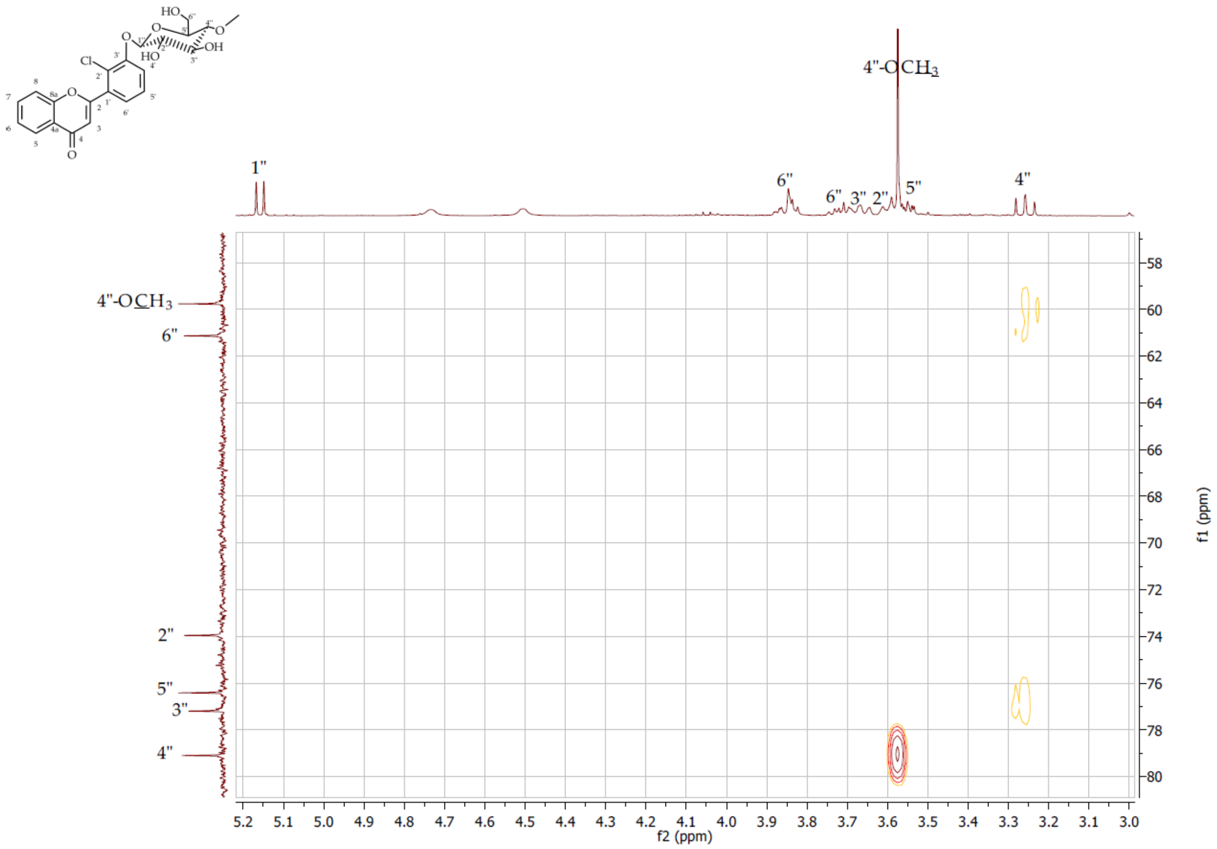

Figure S33. HMBC contour map – ^{1}H x ^{13}C expansion of 2′-chloroflavone 3′-*O*-*β*-D-(4′′-*O*-methyl)-glucopyranoside (**1a**).

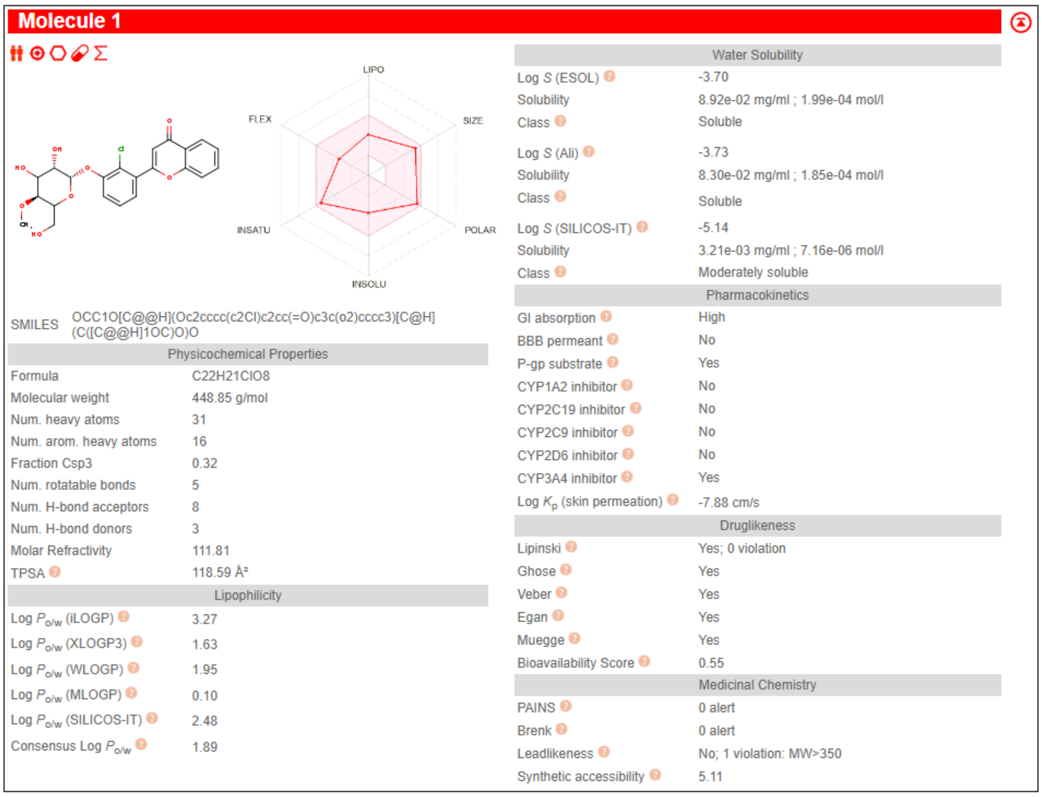

SMILES OCC1O[C@@H](Oc2cccc(c2Cl)c2cc(=O)c3c(o2)cccc3)[C@H](C([C@@H]1OC)O)O

Physicochemical Properties	
Formula	C22H21ClO8
Molecular weight	448.85 g/mol
Num. heavy atoms	31
Num. arom. heavy atoms	16
Fraction Csp3	0.32
Num. rotatable bonds	5
Num. H-bond acceptors	8
Num. H-bond donors	3
Molar Refractivity	111.81
TPSA	118.59 Å²

Lipophilicity	
Log $P_{o/w}$ (iLOGP)	3.27
Log $P_{o/w}$ (XLOGP3)	1.63
Log $P_{o/w}$ (WLOGP)	1.95
Log $P_{o/w}$ (MLOGP)	0.10
Log $P_{o/w}$ (SILICOS-IT)	2.48
Consensus Log $P_{o/w}$	1.89

Water Solubility	
Log *S* (ESOL)	-3.70
Solubility	8.92e-02 mg/ml ; 1.99e-04 mol/l
Class	Soluble
Log *S* (Ali)	-3.73
Solubility	8.30e-02 mg/ml ; 1.85e-04 mol/l
Class	Soluble
Log *S* (SILICOS-IT)	-5.14
Solubility	3.21e-03 mg/ml ; 7.16e-06 mol/l
Class	Moderately soluble

Pharmacokinetics	
GI absorption	High
BBB permeant	No
P-gp substrate	Yes
CYP1A2 inhibitor	No
CYP2C19 inhibitor	No
CYP2C9 inhibitor	No
CYP2D6 inhibitor	No
CYP3A4 inhibitor	Yes
Log K_p (skin permeation)	-7.88 cm/s

Druglikeness	
Lipinski	Yes; 0 violation
Ghose	Yes
Veber	Yes
Egan	Yes
Muegge	Yes
Bioavailability Score	0.55

Medicinal Chemistry	
PAINS	0 alert
Brenk	0 alert
Leadlikeness	No; 1 violation: MW>350
Synthetic accessibility	5.11

Figure S34. 2′-Chloroflavone 3′-*O*-*β*-D-(4′′-*O*-methyl)-glucopyranoside (**1a**) physicochemical and ADME parameters prediction using the SwissADME modelling.

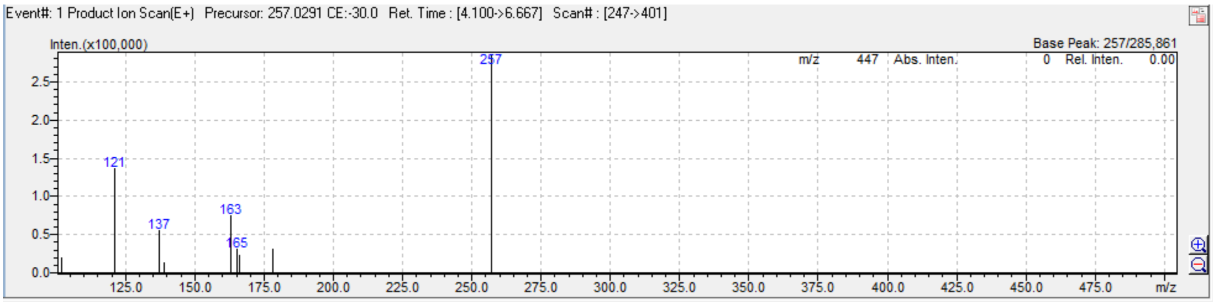

Figure S35. MS analysis of 3′-chloroflavone (**2**).

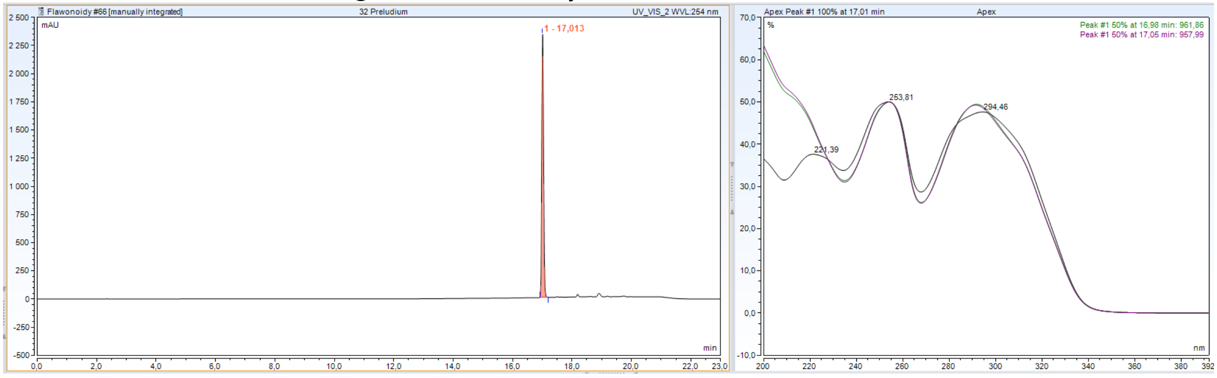

Figure S36. HPLC analysis of 3′-chloroflavone (**2**).

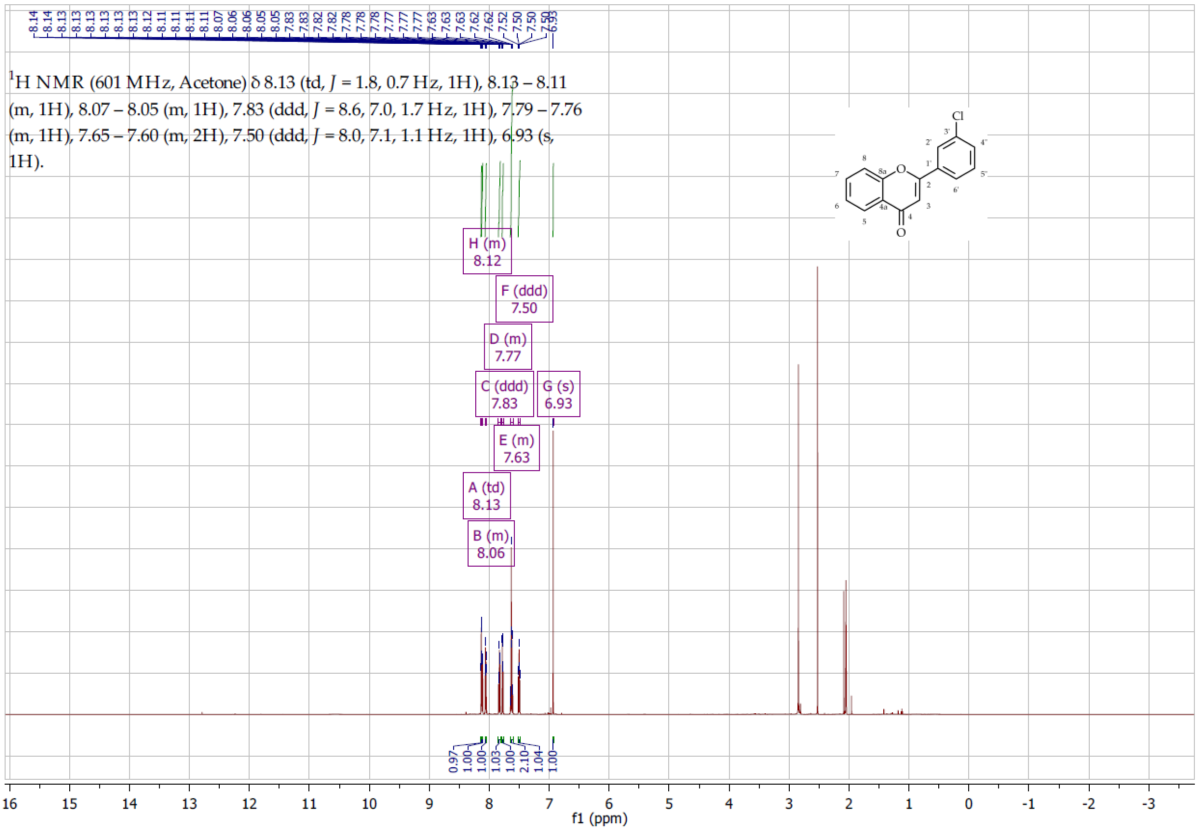

Figure S37. ^{1}H NMR spectrum (*δ*, acetone-d6, 600 MHz) of 3′-chloroflavone (**2**).

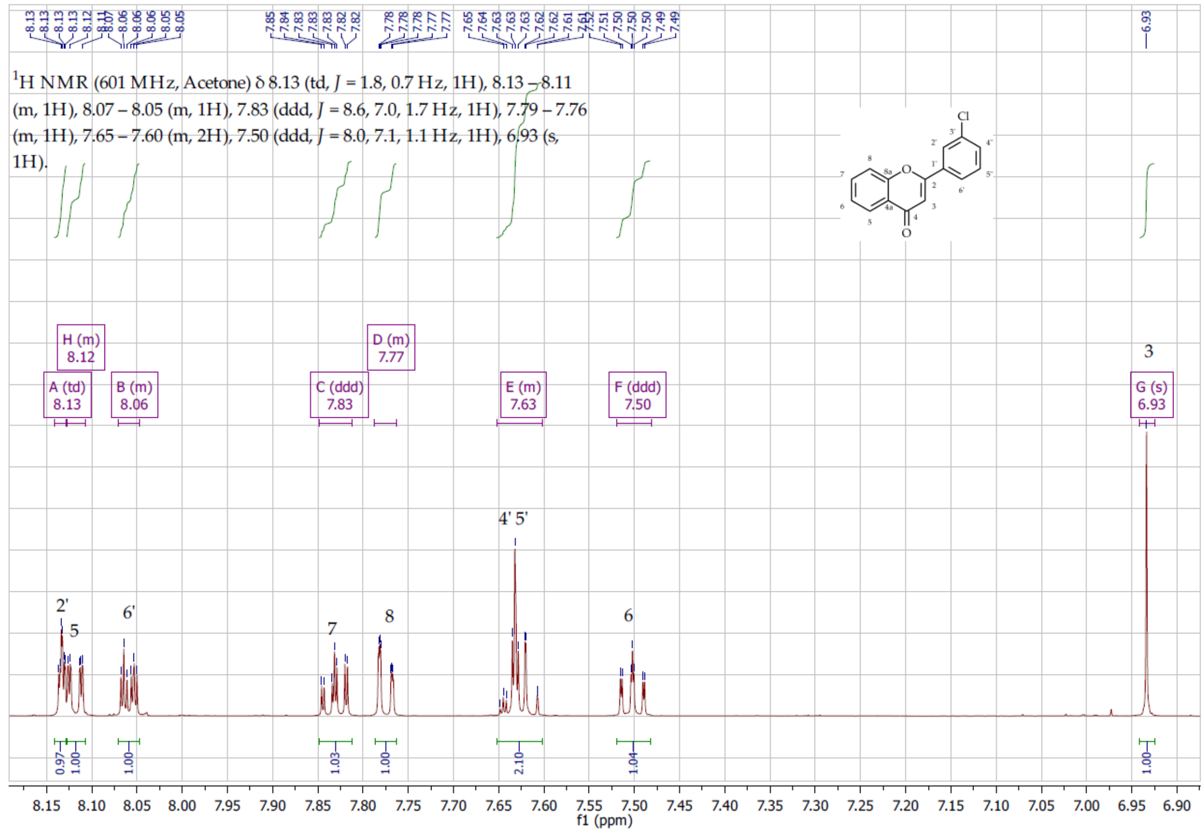

Figure S38. ^{1}H NMR spectrum expansion (δ, acetone-d6, 600 MHz) of 3′-chloroflavone (**2**).

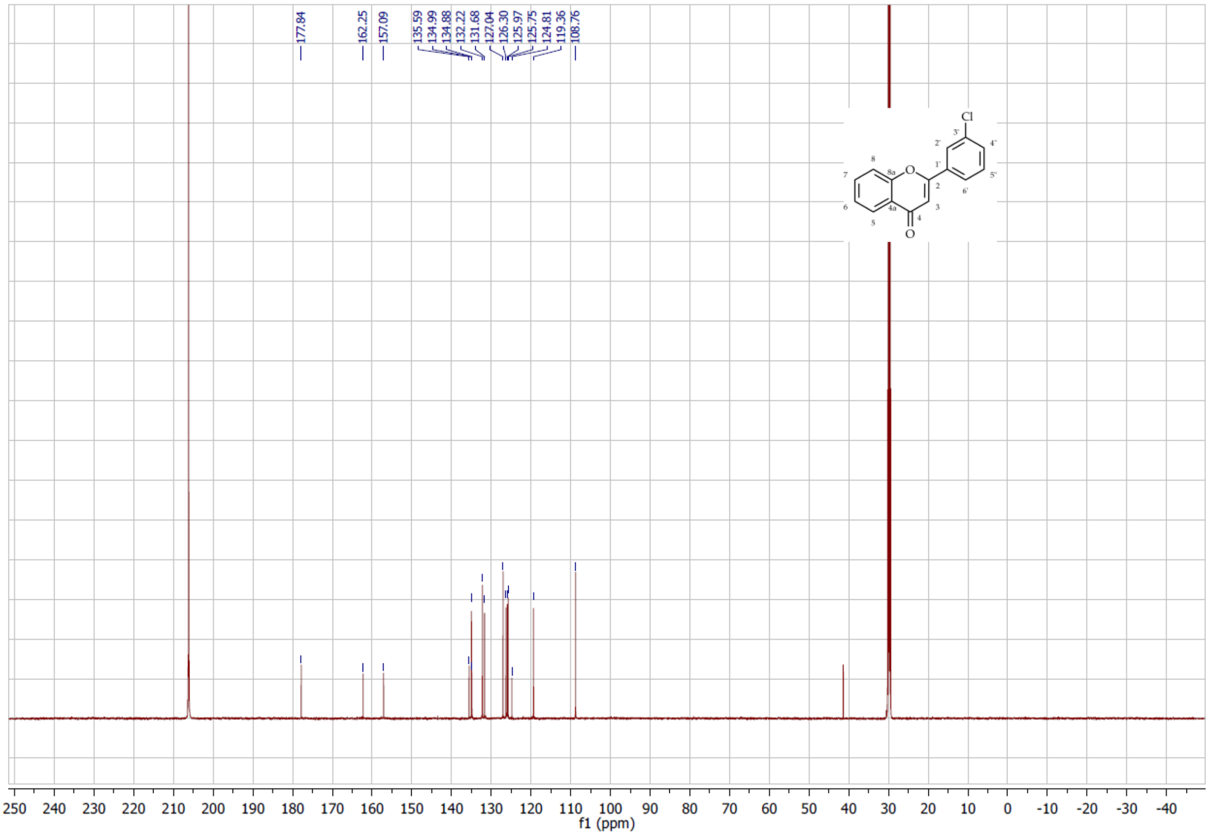

Figure S39. ^{13}C NMR spectrum (δ, acetone-d6, 151 MHz) of 3′-chloroflavone (**2**).

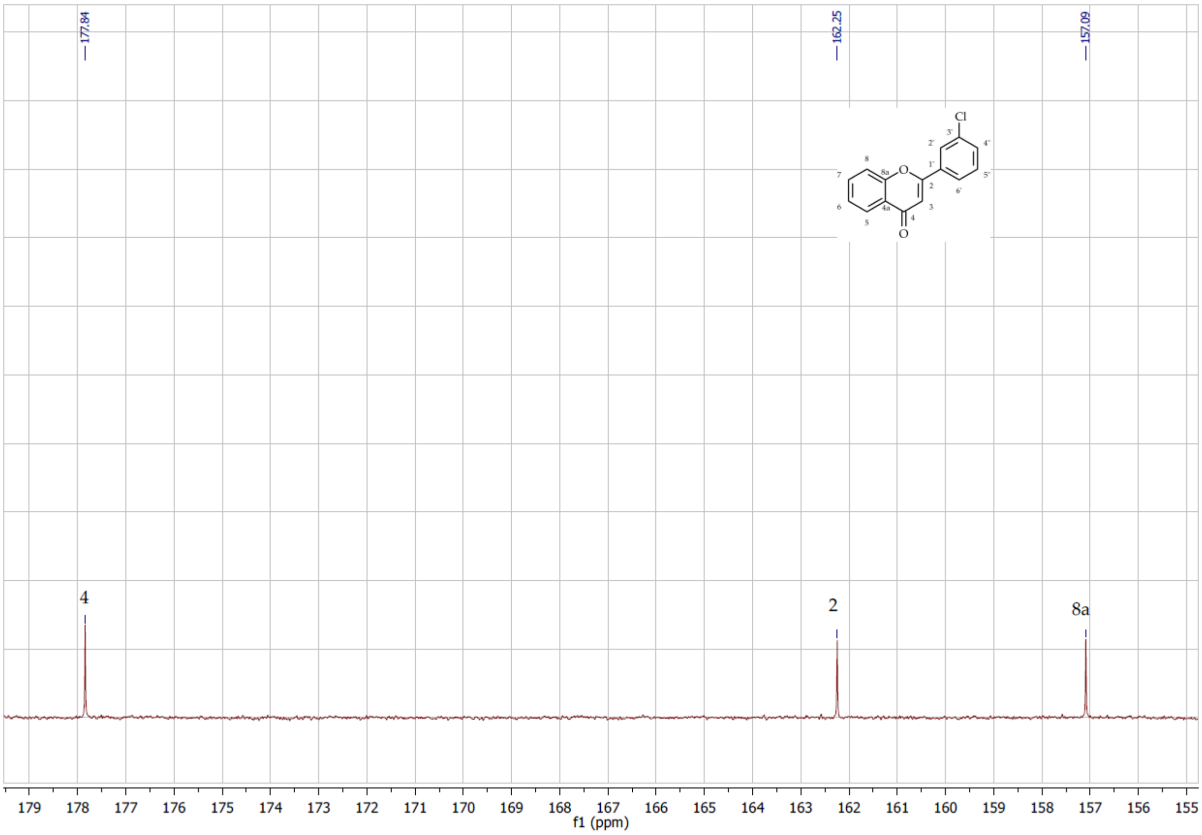

Figure S40. ^{13}C NMR spectrum expansion (*δ*, acetone-d6, 151 MHz) of 3′-chloroflavone (**2**).

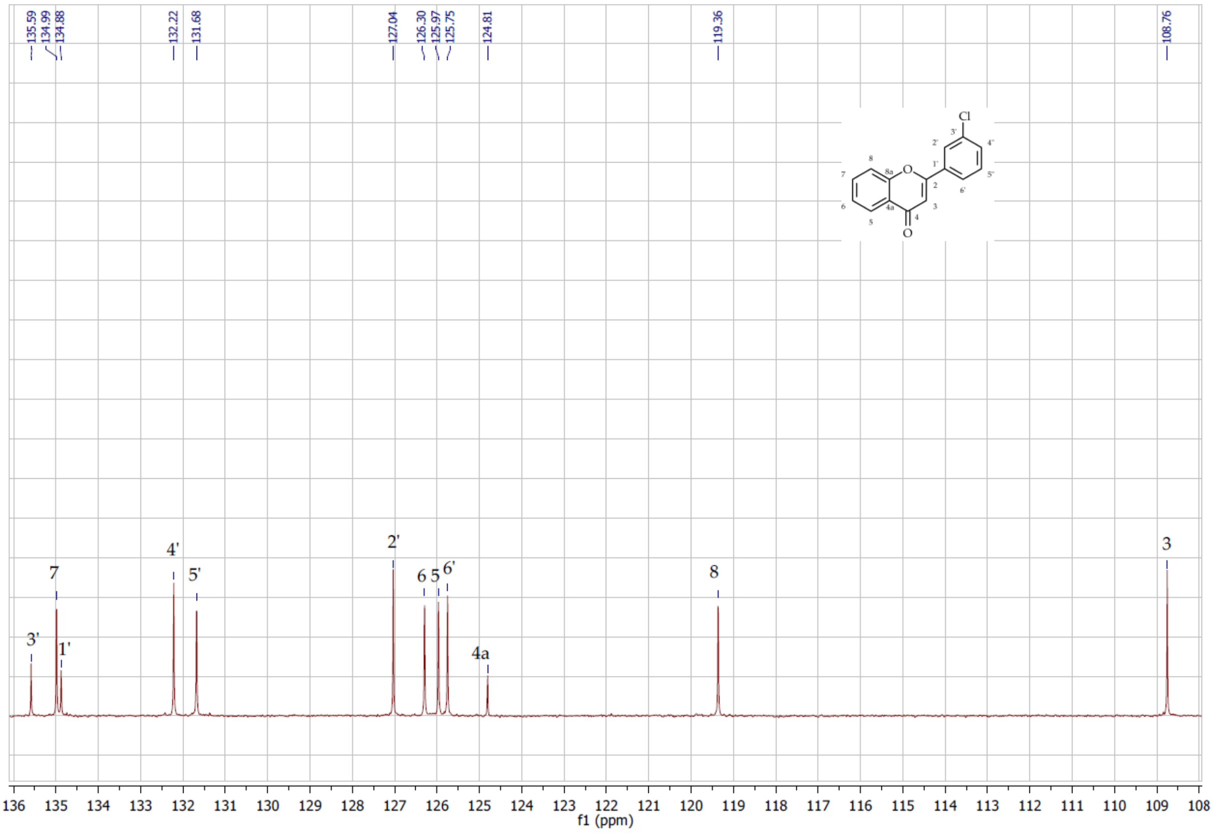

Figure S41. ^{13}C NMR spectrum expansion (*δ*, acetone-d6, 151 MHz) of 3′-chloroflavone (**2**).

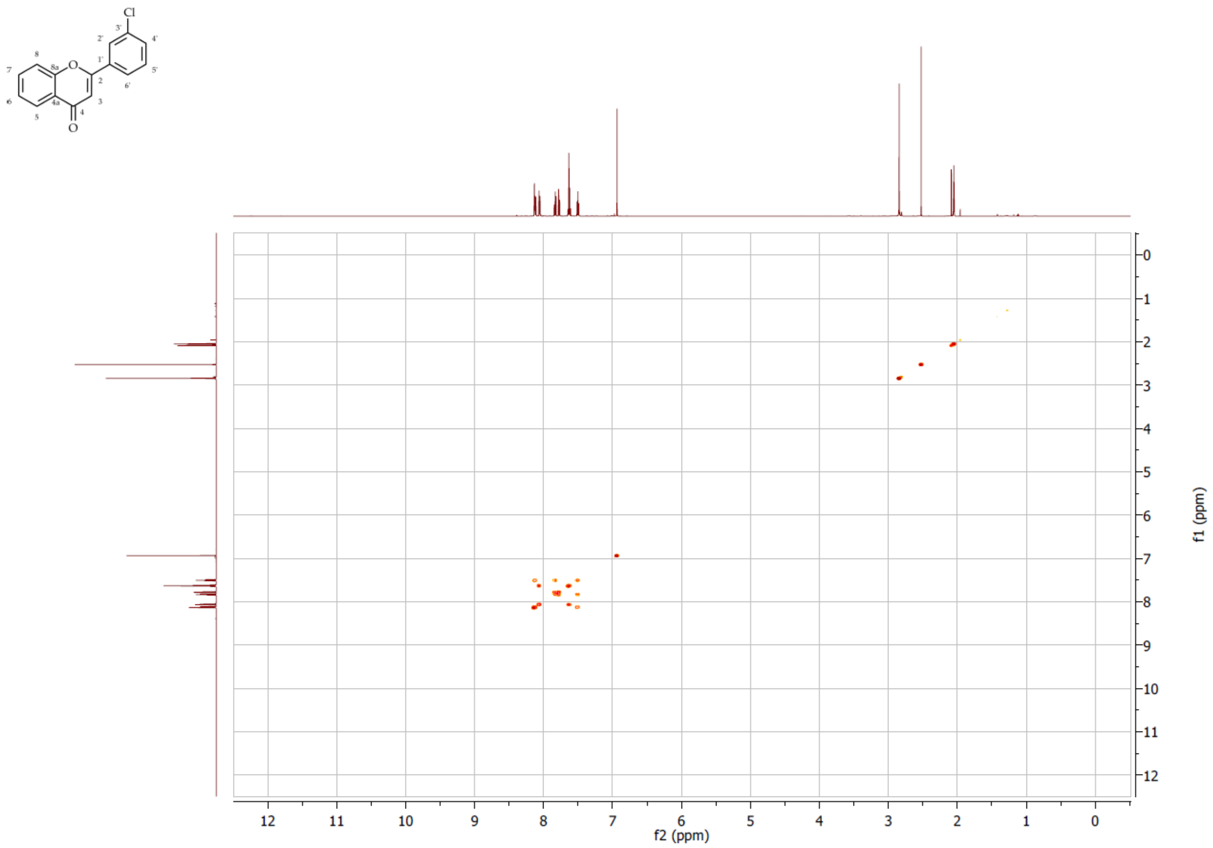

Figure S42. COSY contour map – ^{1}H x ^{1}H of 3′-chloroflavone (**2**).

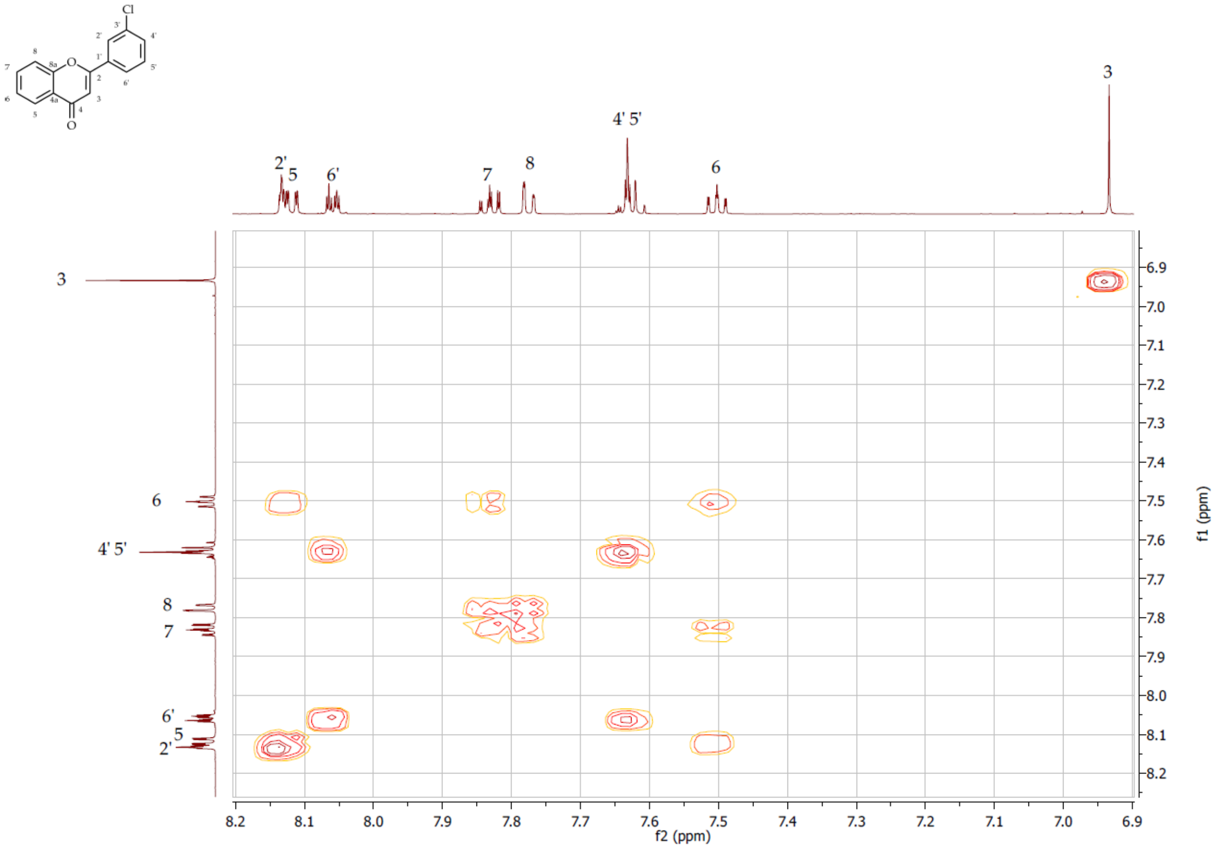

Figure S43. COSY contour map – ^{1}H x ^{1}H expansion of 3′-chloroflavone (**2**).

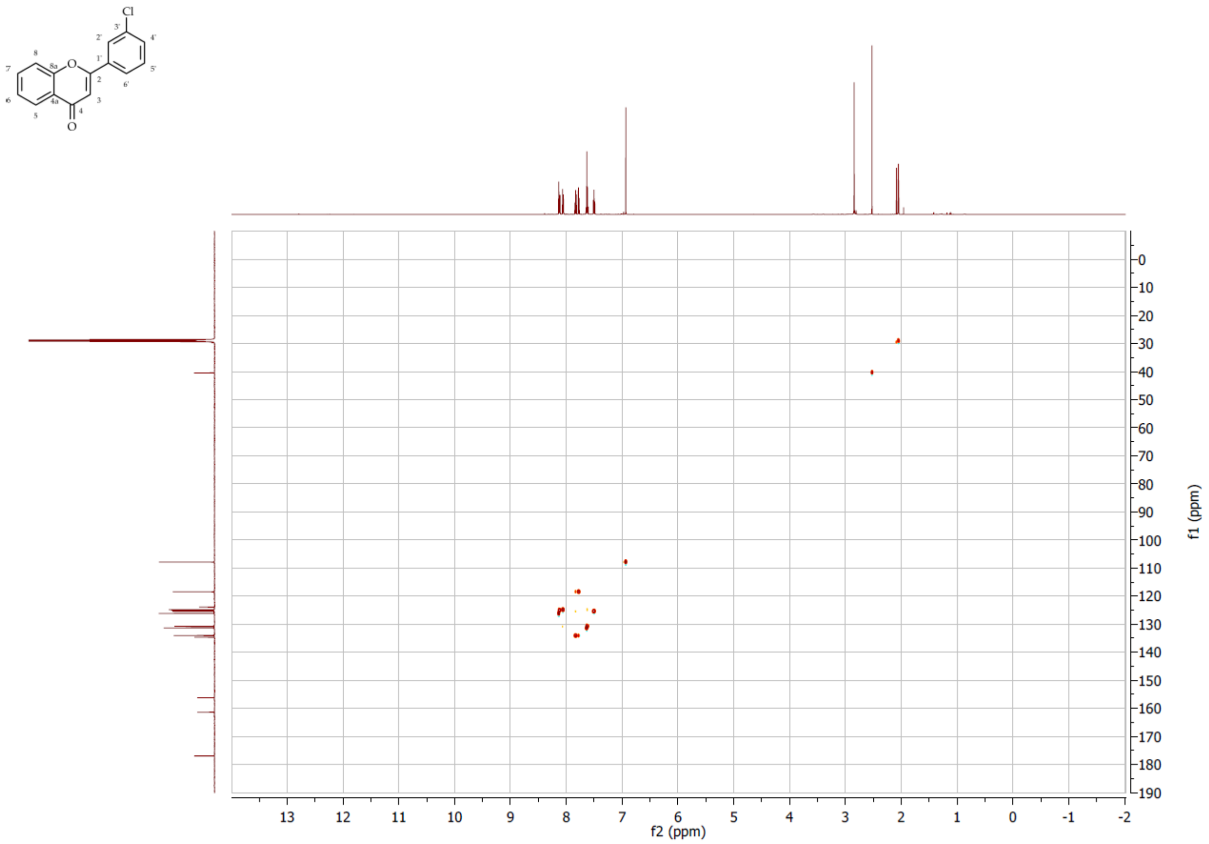

Figure S44. HMQC contour map – [1]H x ^{13}C of 3′-chloroflavone (**2**).

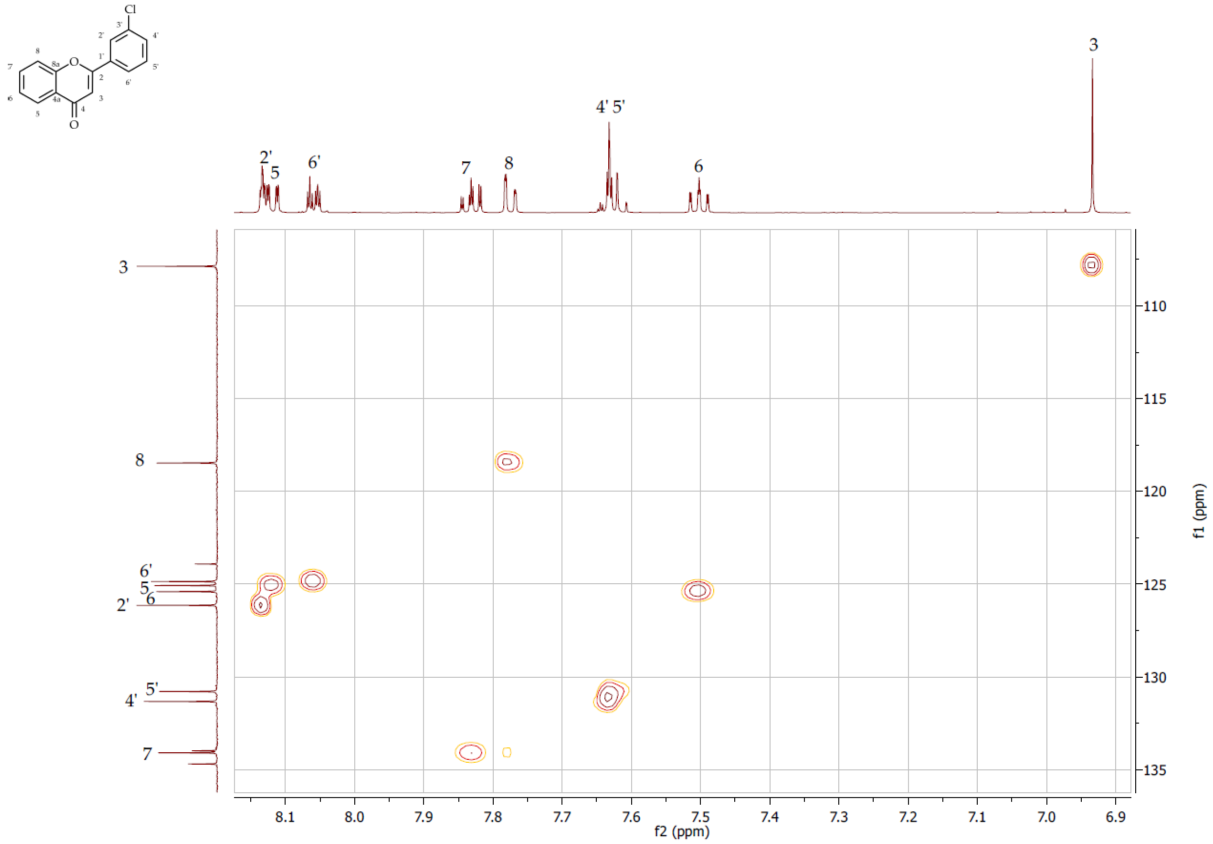

Figure S45. HMQC contour map – [1]H x ^{13}C expansion of 3′-chloroflavone (**2**).

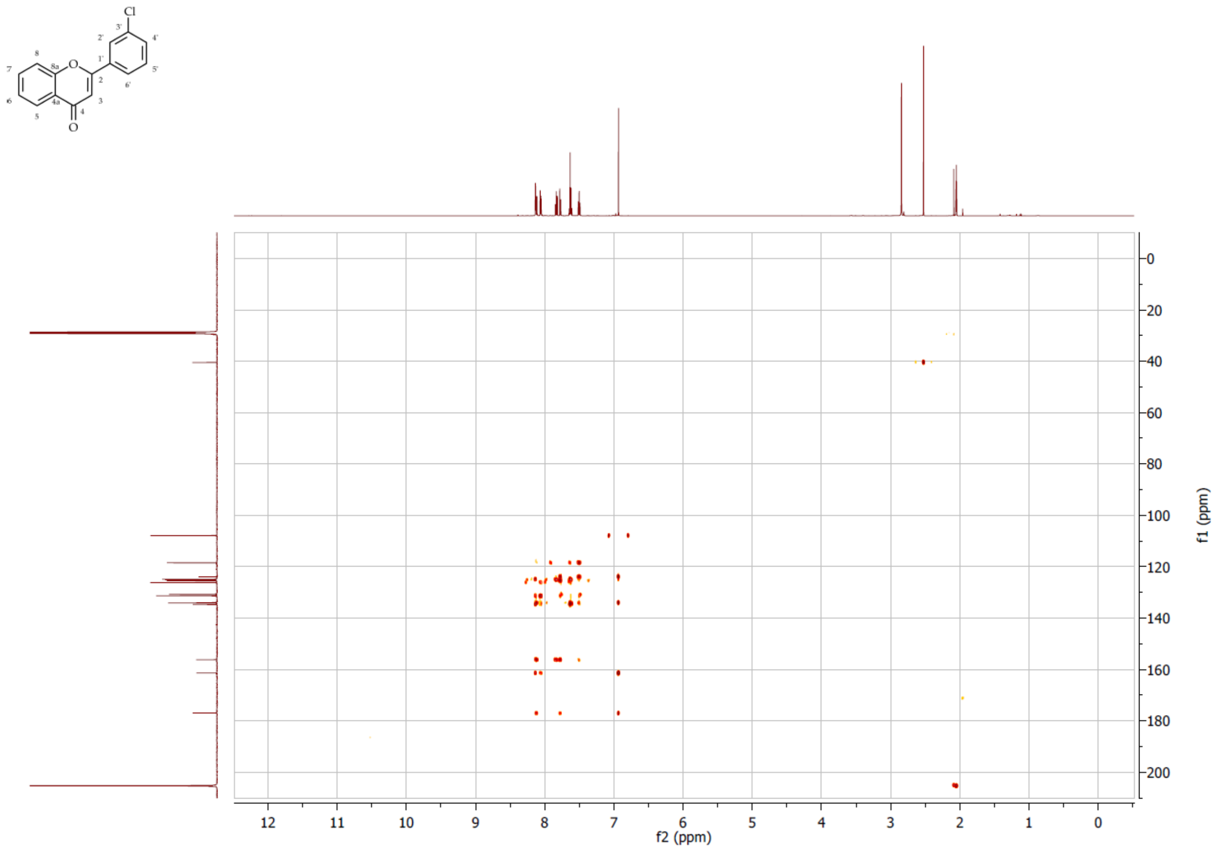

Figure S46. HMBC contour map – ^{1}H x ^{13}C of 3′-chloroflavone (**2**).

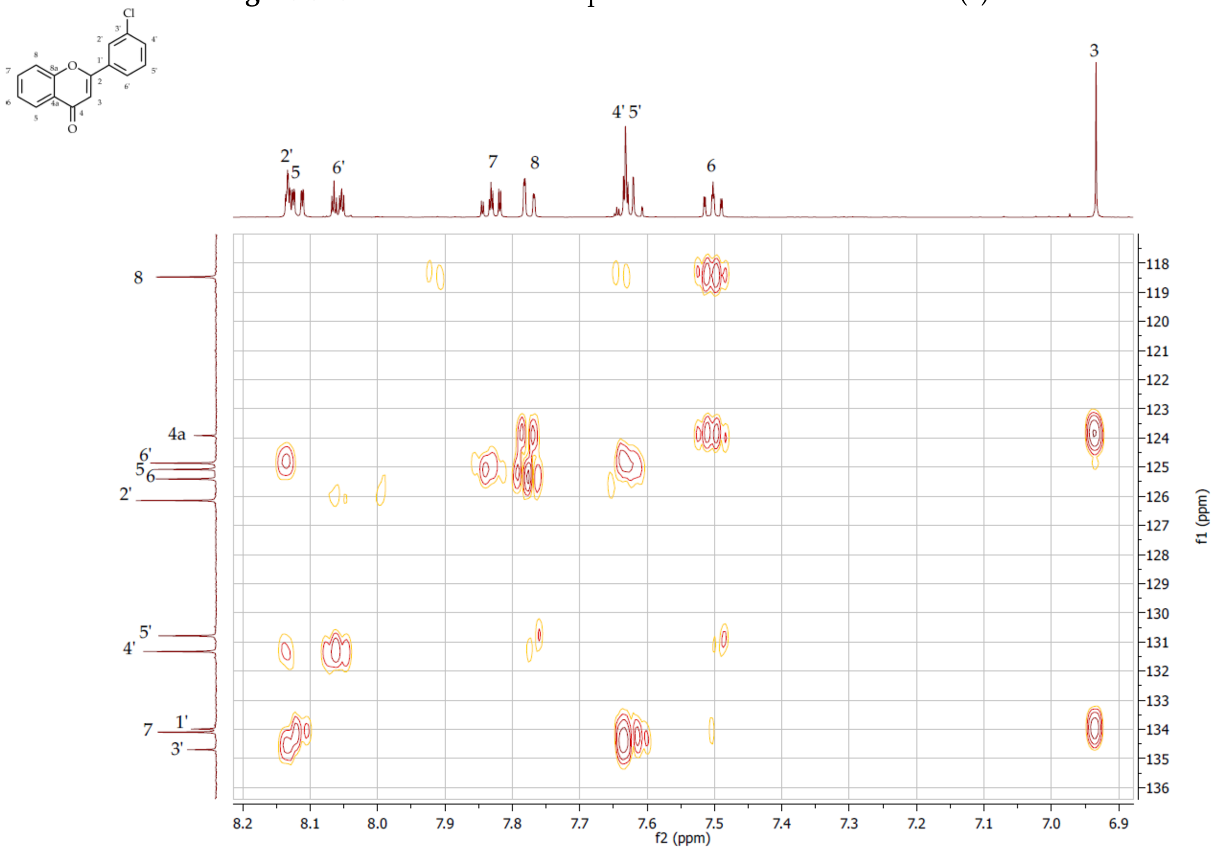

Figure S47. HMBC contour map – ^{1}H x ^{13}C expansion of 3′-chloroflavone (**2**).

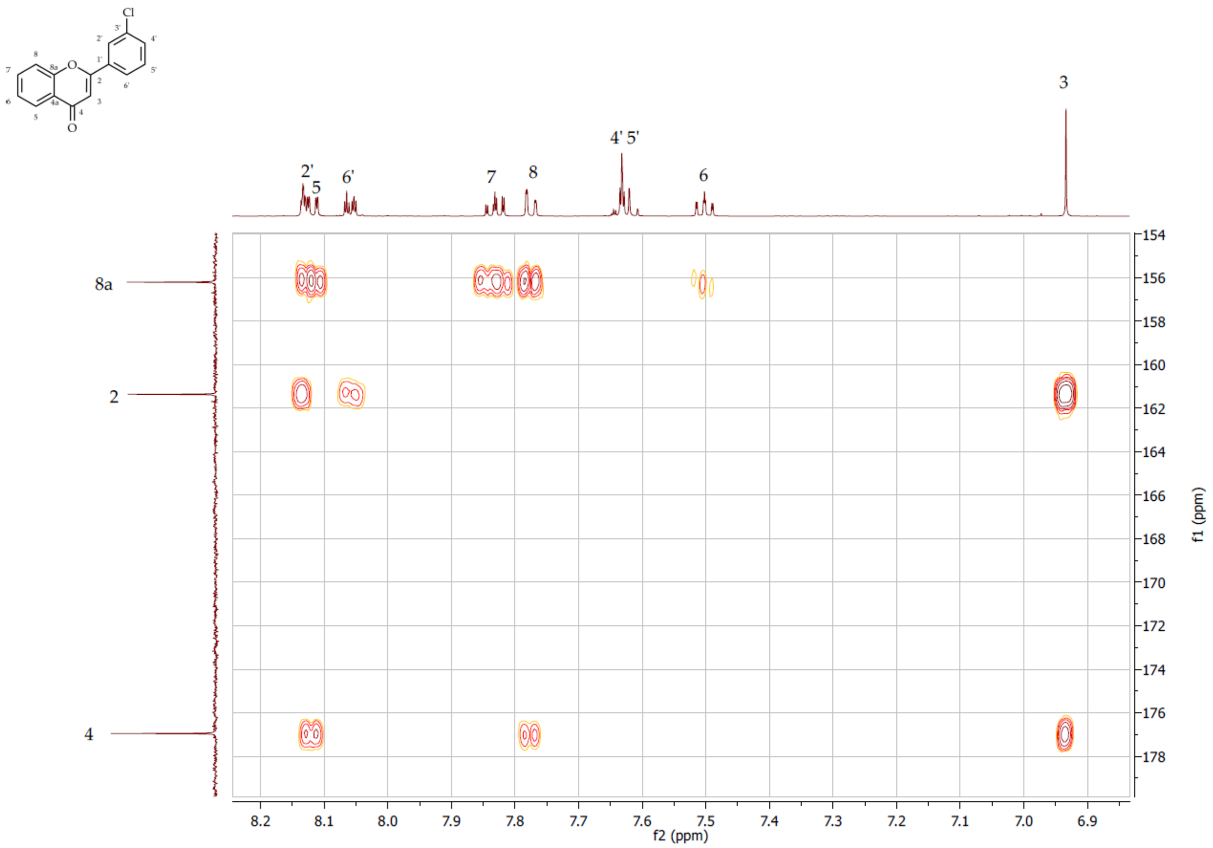

Figure S48. HMBC contour map – ^{1}H x ^{13}C expansion of 3′-chloroflavone (**2**).

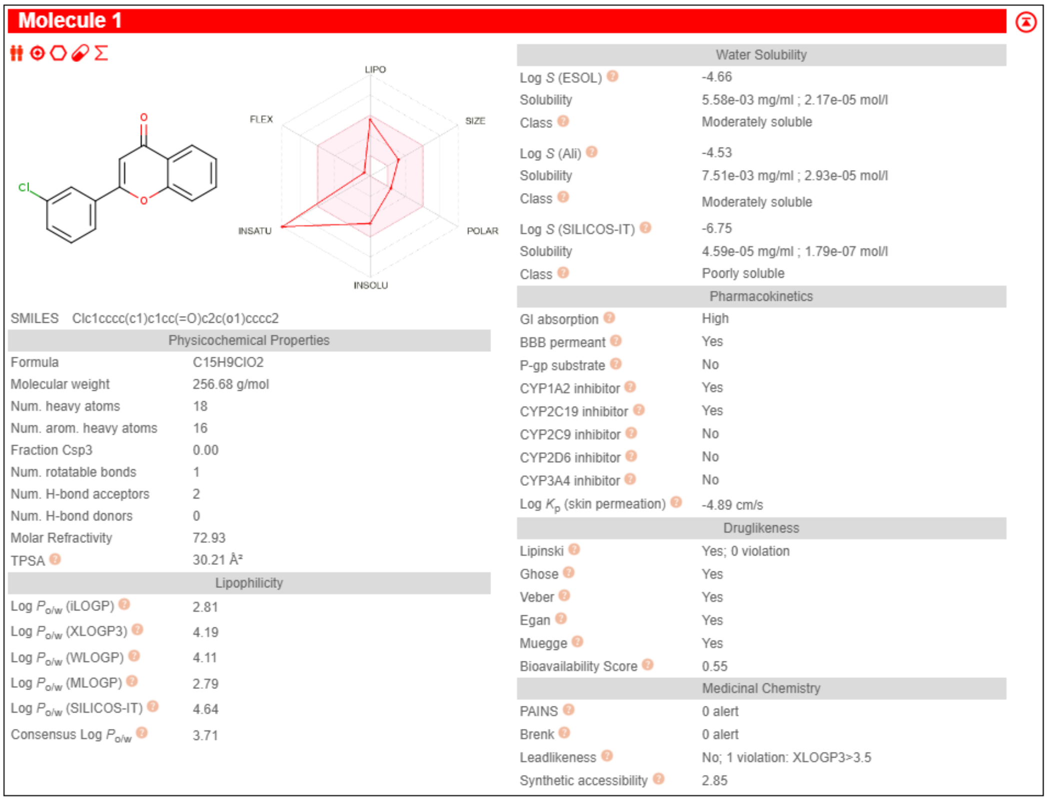

SMILES Clc1cccc(c1)c1cc(=O)c2c(o1)cccc2

Physicochemical Properties	
Formula	C15H9ClO2
Molecular weight	256.68 g/mol
Num. heavy atoms	18
Num. arom. heavy atoms	16
Fraction Csp3	0.00
Num. rotatable bonds	1
Num. H-bond acceptors	2
Num. H-bond donors	0
Molar Refractivity	72.93
TPSA	30.21 Å²

Lipophilicity	
Log $P_{o/w}$ (iLOGP)	2.81
Log $P_{o/w}$ (XLOGP3)	4.19
Log $P_{o/w}$ (WLOGP)	4.11
Log $P_{o/w}$ (MLOGP)	2.79
Log $P_{o/w}$ (SILICOS-IT)	4.64
Consensus Log $P_{o/w}$	3.71

Water Solubility	
Log *S* (ESOL)	-4.66
Solubility	5.58e-03 mg/ml ; 2.17e-05 mol/l
Class	Moderately soluble
Log *S* (Ali)	-4.53
Solubility	7.51e-03 mg/ml ; 2.93e-05 mol/l
Class	Moderately soluble
Log *S* (SILICOS-IT)	-6.75
Solubility	4.59e-05 mg/ml ; 1.79e-07 mol/l
Class	Poorly soluble

Pharmacokinetics	
GI absorption	High
BBB permeant	Yes
P-gp substrate	No
CYP1A2 inhibitor	Yes
CYP2C19 inhibitor	Yes
CYP2C9 inhibitor	No
CYP2D6 inhibitor	No
CYP3A4 inhibitor	No
Log K_p (skin permeation)	-4.89 cm/s

Druglikeness	
Lipinski	Yes; 0 violation
Ghose	Yes
Veber	Yes
Egan	Yes
Muegge	Yes
Bioavailability Score	0.55

Medicinal Chemistry	
PAINS	0 alert
Brenk	0 alert
Leadlikeness	No; 1 violation: XLOGP3>3.5
Synthetic accessibility	2.85

Figure S49. 3′-Chloroflavone (**2**) physicochemical and ADME parameters prediction using the SwissADME modelling.

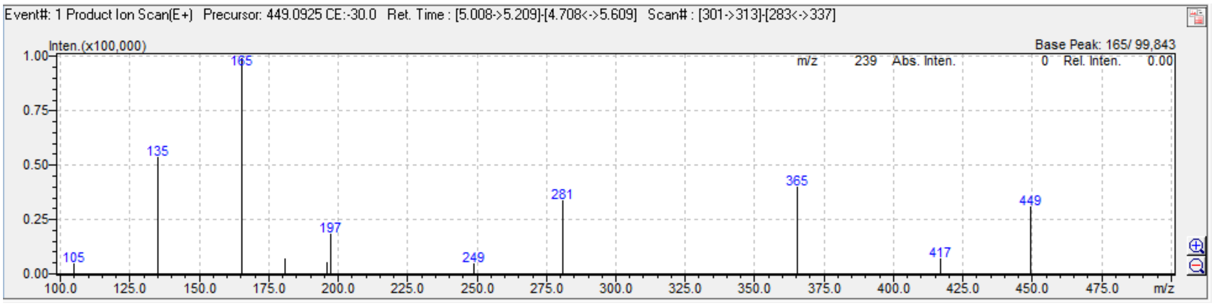

Figure S50. MS analysis of 3′-chloroflavone 4′-*O*-*β*-D-(4′′-*O*-methyl)-glucopyranoside (**2a**).

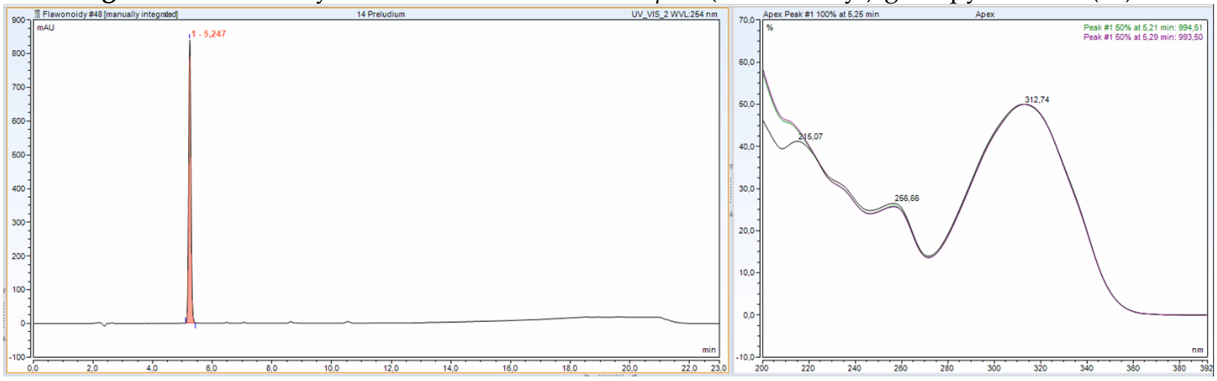

Figure S51. HPLC analysis of 3′-chloroflavone 4′-*O*-*β*-D-(4′′-*O*-methyl)-glucopyranoside (**2a**).

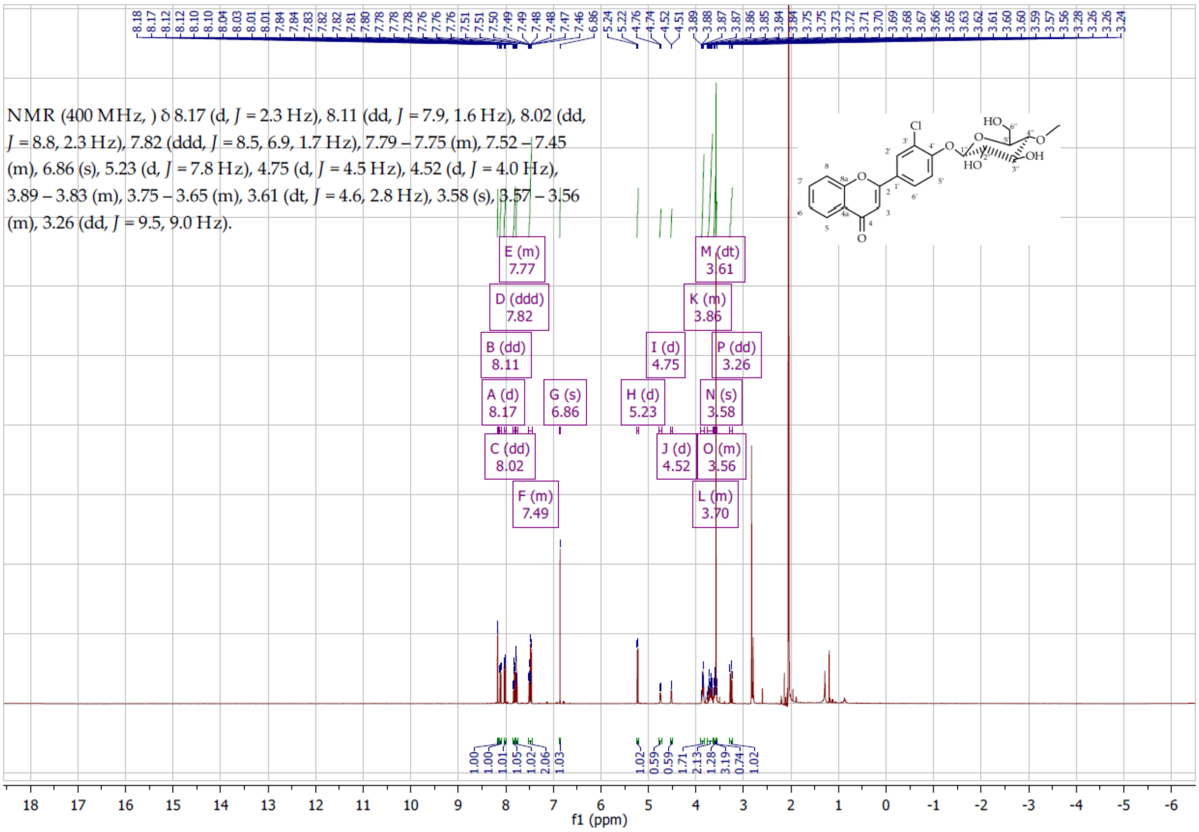

Figure S52. ^{1}H NMR spectrum (*δ*, acetone-d6, 600 MHz) of 3′-chloroflavone 4′-*O*-*β*-D-(4′′-*O*-methyl)-glucopyranoside (**2a**).

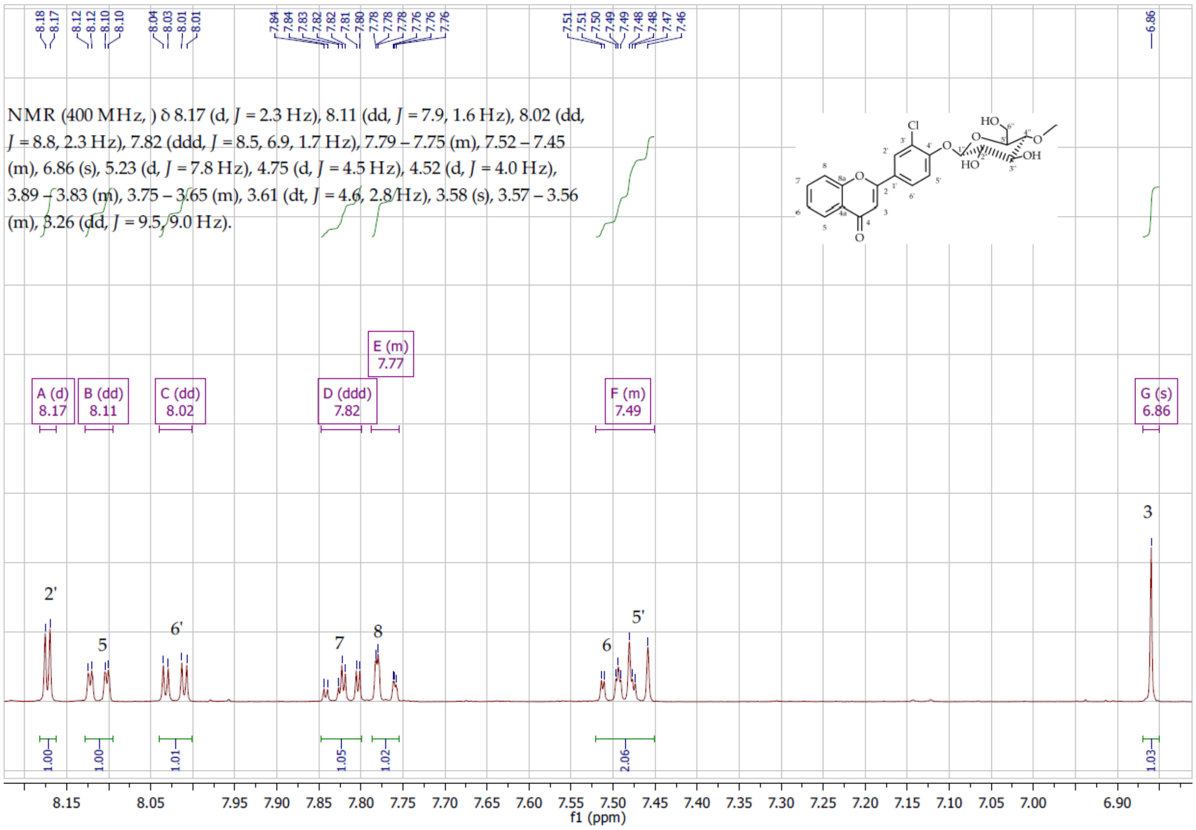

Figure S53. ^{1}H NMR spectrum expansion (*δ*, acetone-d6, 600 MHz) of 3′-chloroflavone 4′-*O*-*β*-D-(4′′-*O*-methyl)-glucopyranoside (**2a**).

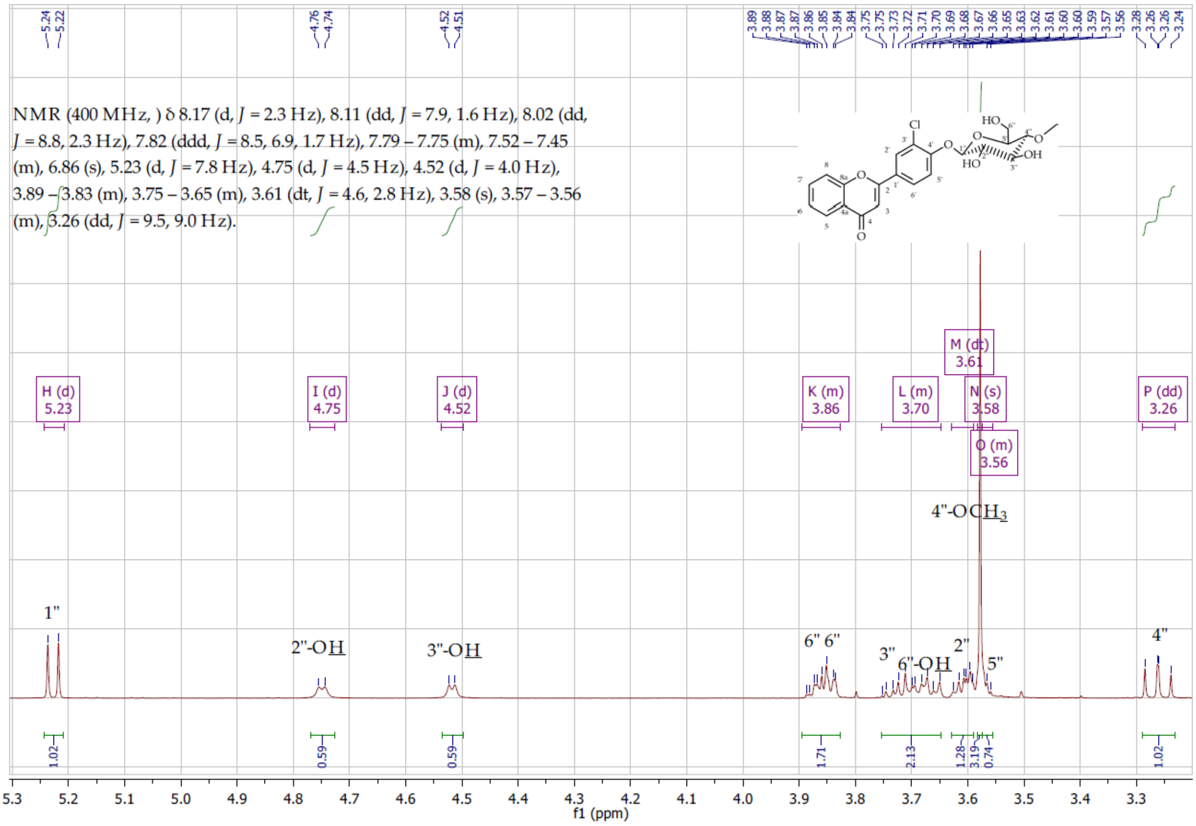

Figure S54. ^{1}H NMR spectrum expansion (*δ*, acetone-d6, 600 MHz) of 3′-chloroflavone 4′-*O*-*β*-D-(4′′-*O*-methyl)-glucopyranoside (**2a**).

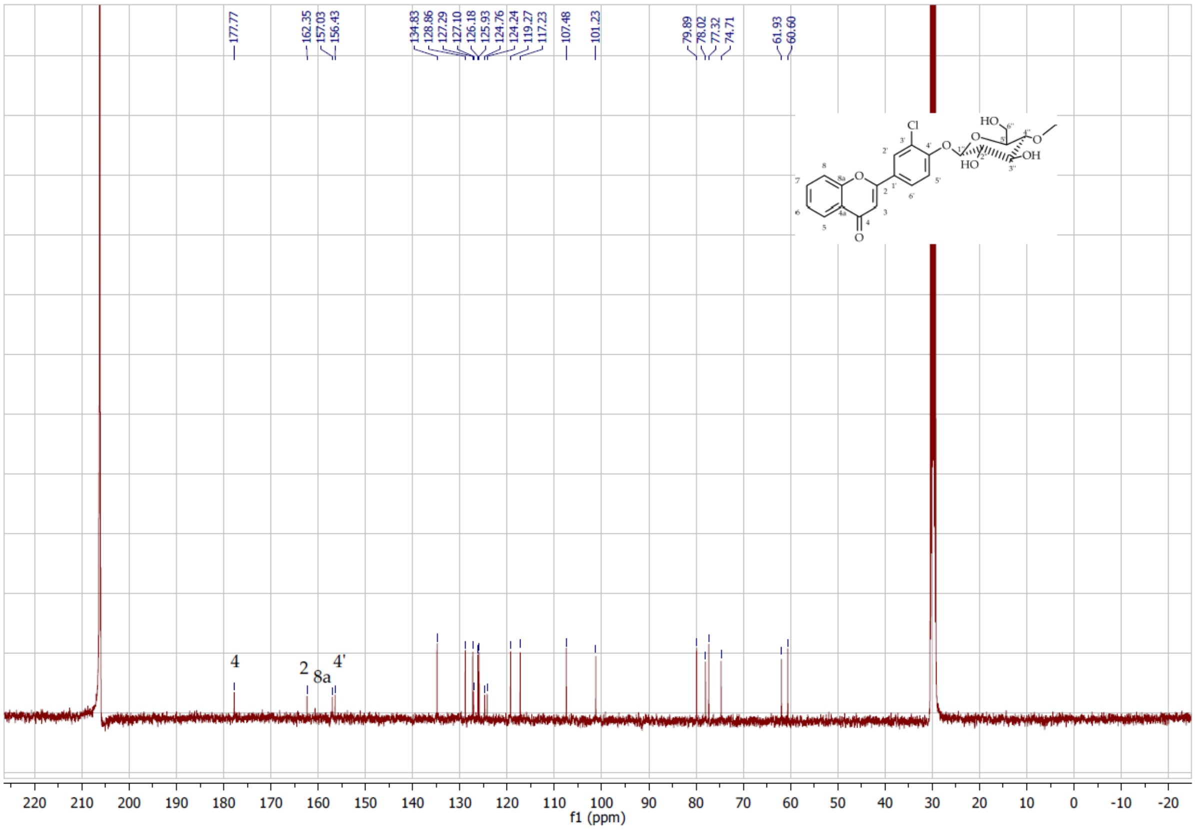

Figure S55. ^{13}C NMR spectrum (*δ*, acetone-d6, 151 MHz) of 3′-chloroflavone 4′-*O*-*β*-D-(4′′-*O*-methyl)-glucopyranoside (**2a**).

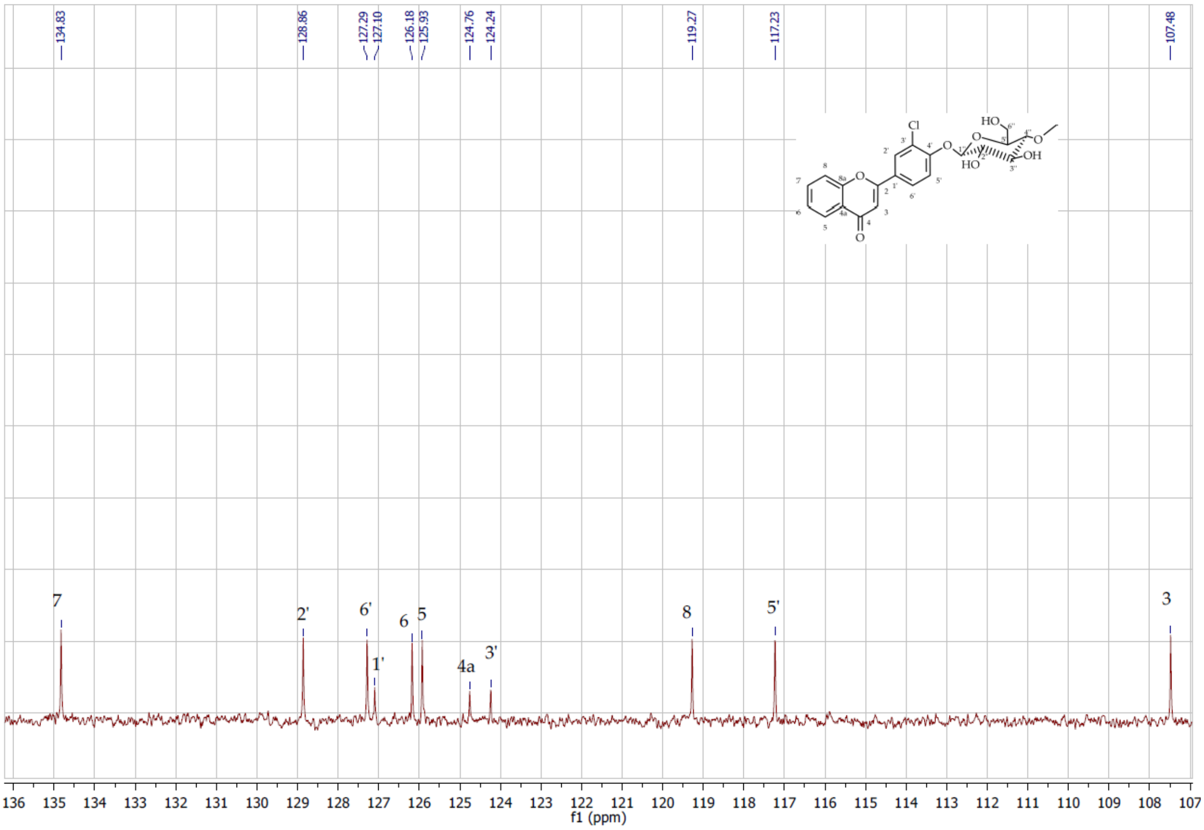

Figure S56. ^{13}C NMR spectrum expansion (*δ*, acetone-d6, 151 MHz) of 3′-chloroflavone 4′-*O*-*β*-D-(4′′-*O*-methyl)-glucopyranoside (**2a**).

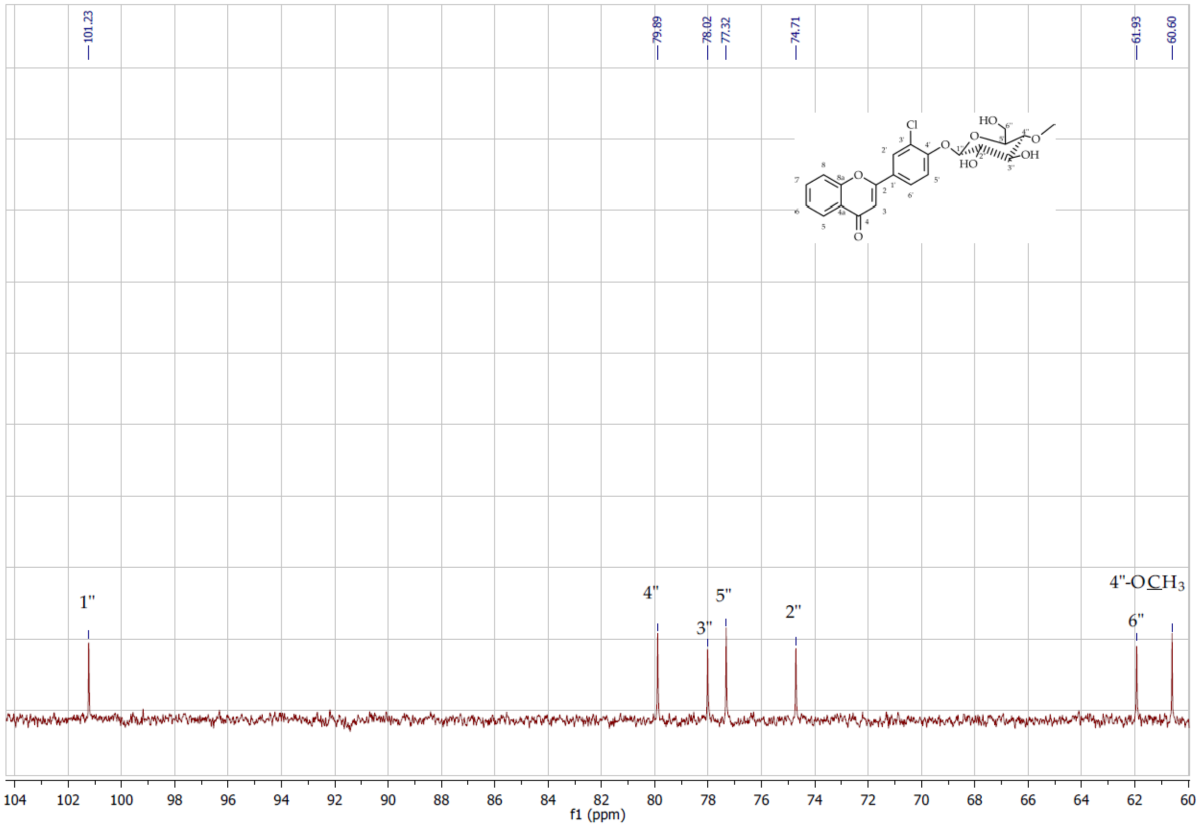

Figure S57. ^{13}C NMR spectrum expansion (*δ*, acetone-d6, 151 MHz) of 3′-chloroflavone 4′-*O*-*β*-D-(4′′-*O*-methyl)-glucopyranoside (**2a**).

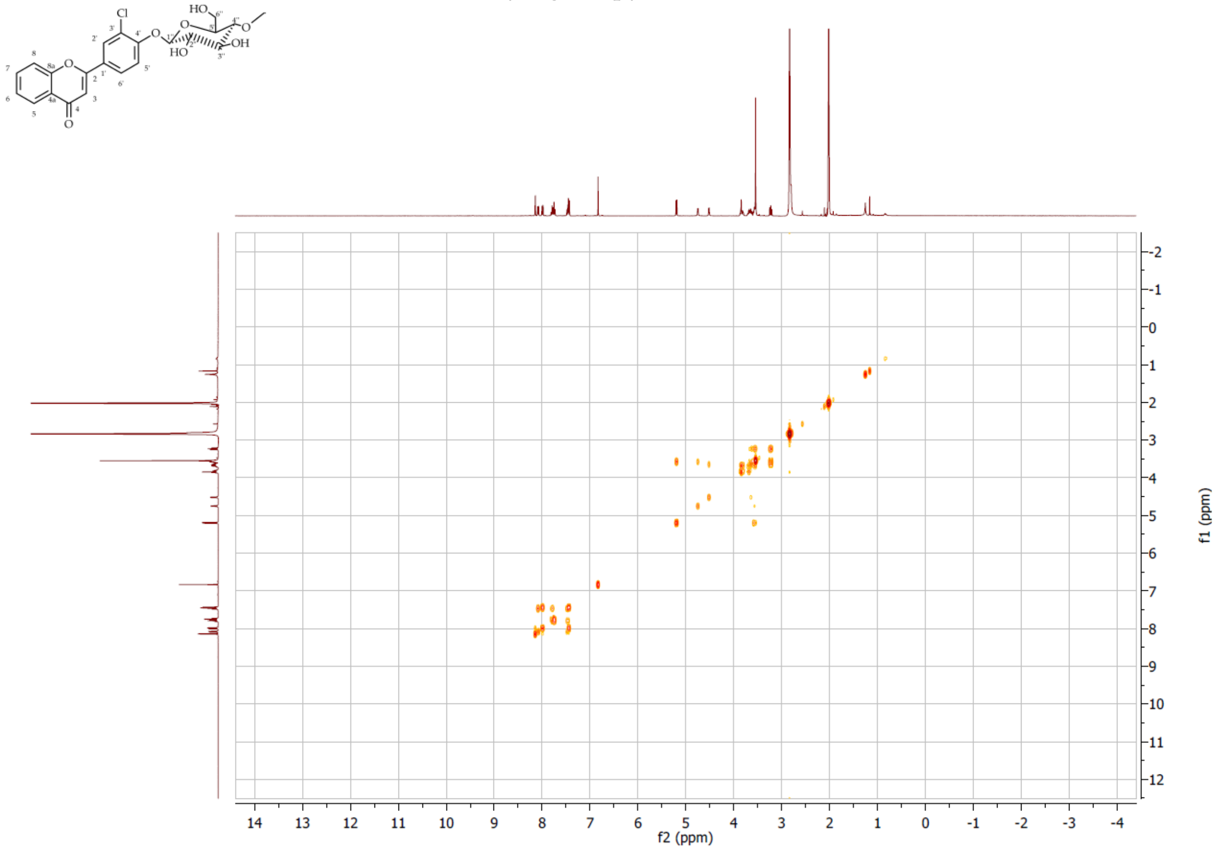

Figure S58. COSY contour map – ^{1}H x ^{1}H of 3′-chloroflavone 4′-*O*-*β*-D-(4′′-*O*-methyl)-glucopyranoside (**2a**).

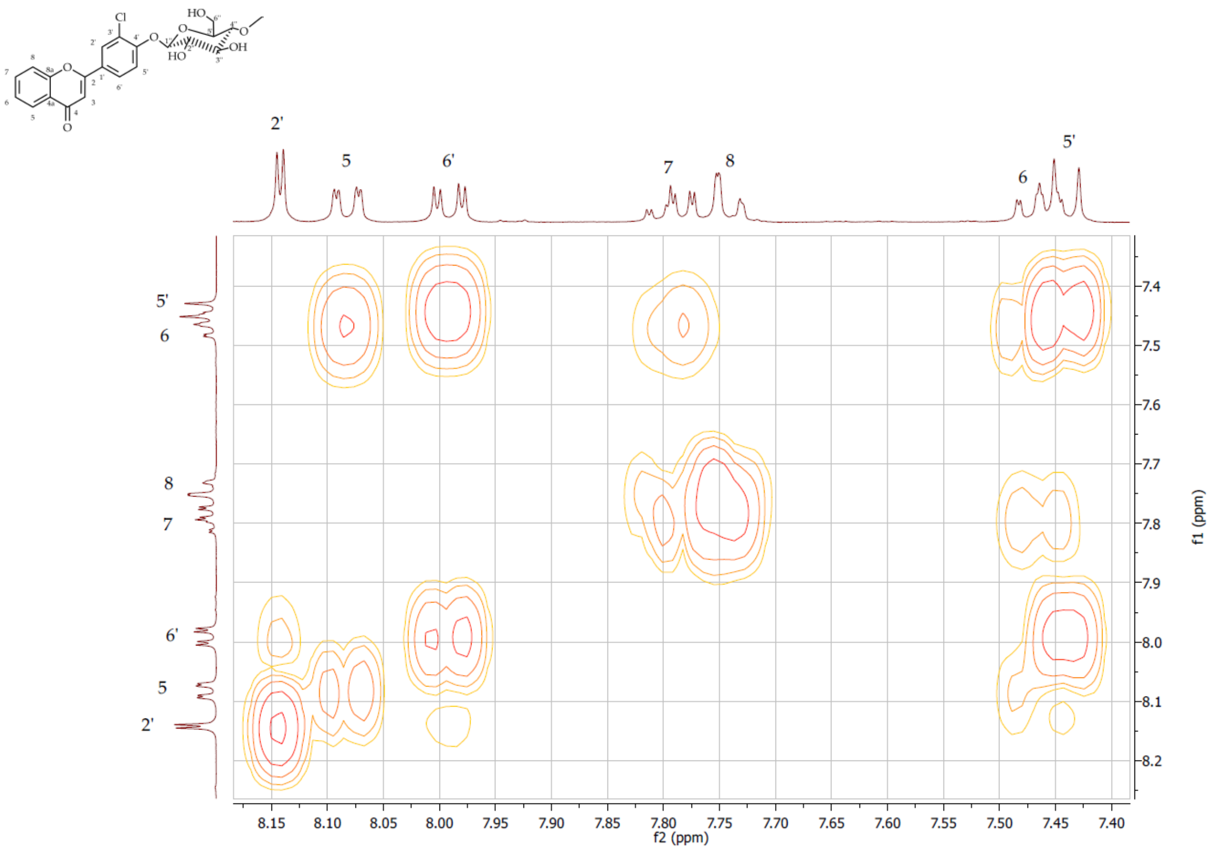

Figure S59. COSY contour map – ^1H x ^1H expansion of 3′-chloroflavone 4′-*O*-*β*-D-(4′′-*O*-methyl)-glucopyranoside (**2a**).

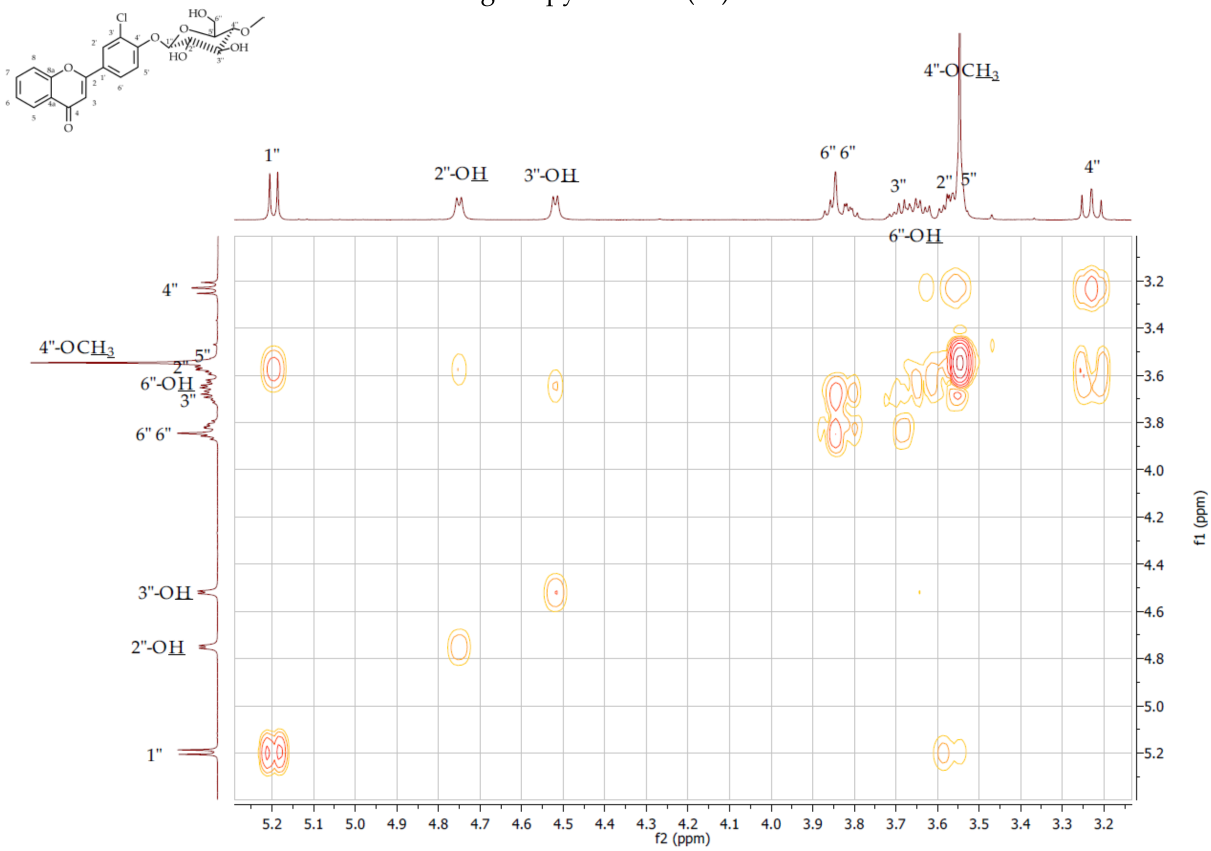

Figure S60. COSY contour map – ^1H x ^1H expansion of 3′-chloroflavone 4′-*O*-*β*-D-(4′′-*O*-methyl)-glucopyranoside (**2a**).

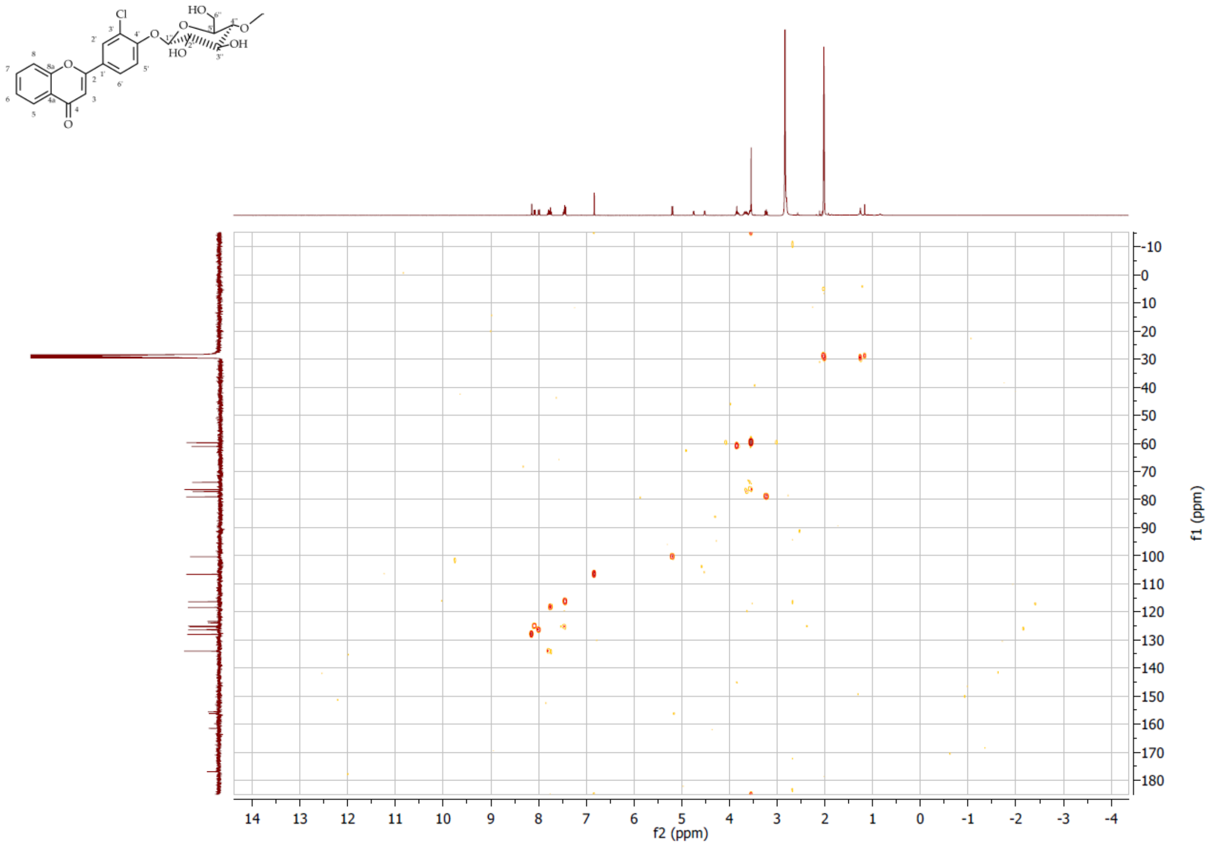

Figure S61. HMQC contour map – ^{1}H x ^{13}C of 3′-chloroflavone 4′-*O*-*β*-D-(4′′-*O*-methyl)-glucopyranoside (**2a**).

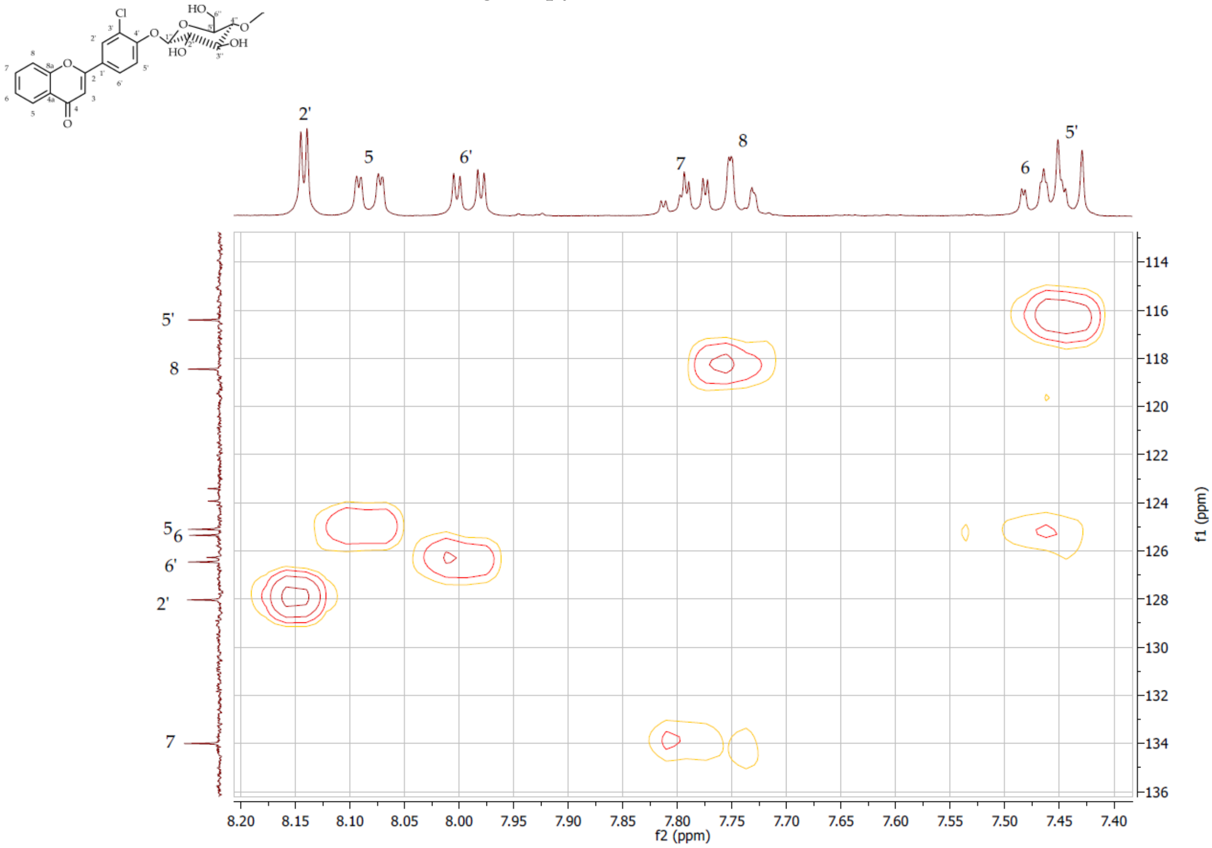

Figure S62. HMQC contour map – ^{1}H x ^{13}C expansion of 3′-chloroflavone 4′-*O*-*β*-D-(4′′-*O*-methyl)-glucopyranoside (**2a**).

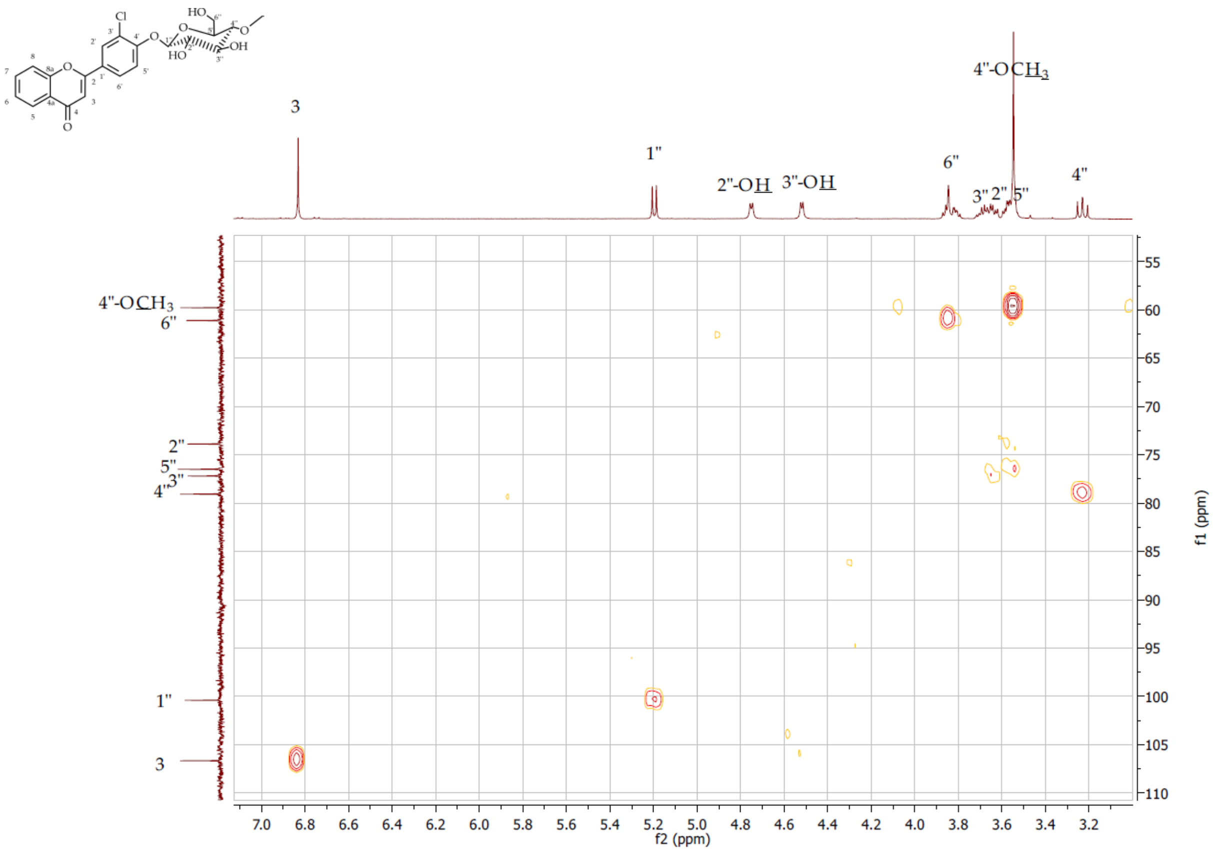

Figure S63. HMQC contour map – ^{1}H x ^{13}C expansion of 3′-chloroflavone 4′-*O*-*β*-D-(4′′-*O*-methyl)-glucopyranoside (**2a**).

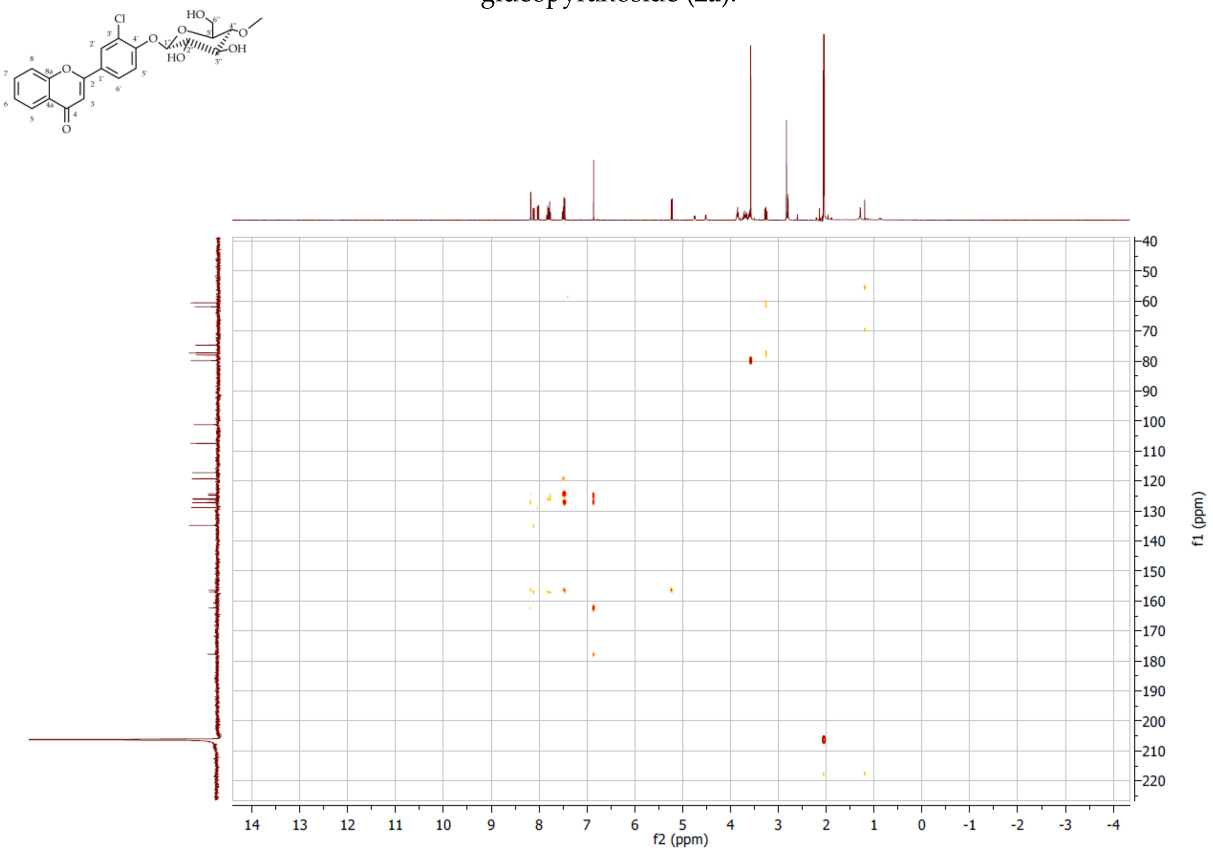

Figure S64. HMBC contour map – ^{1}H x ^{13}C of 3′-chloroflavone 4′-*O*-*β*-D-(4′′-*O*-methyl)-glucopyranoside (**2a**).

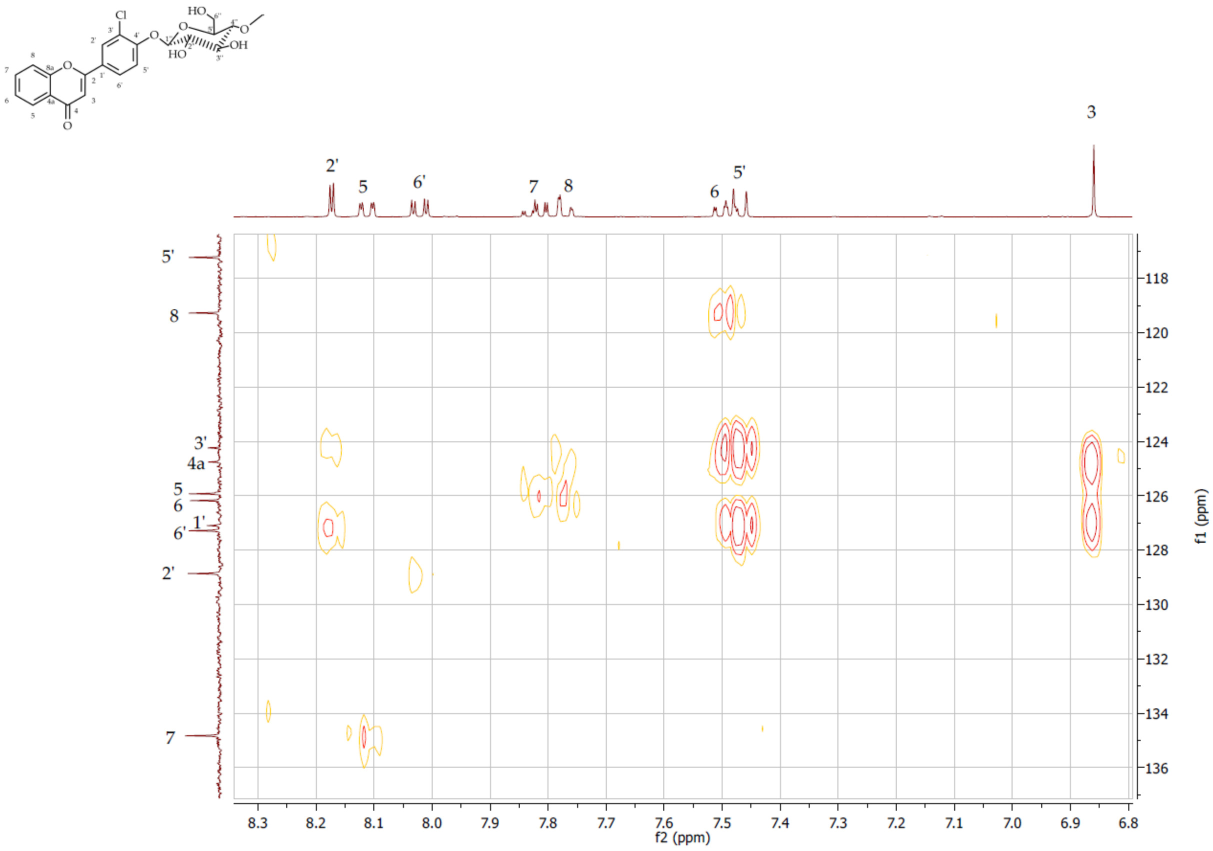

Figure S65. HMBC contour map – ^{1}H x ^{13}C expansion of 3′-chloroflavone 4′-*O*-*β*-D-(4′′-*O*-methyl)-glucopyranoside (**2a**).

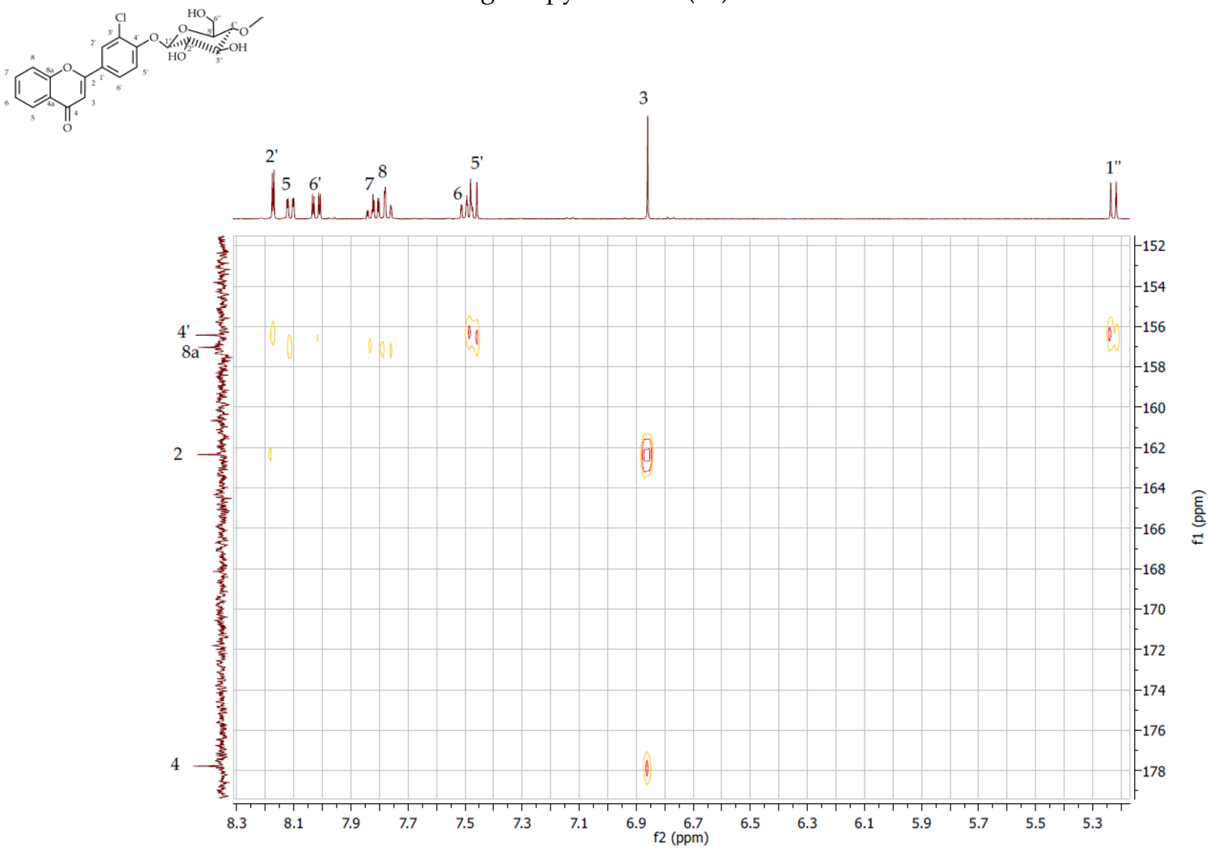

Figure S66. HMBC contour map – ^{1}H x ^{13}C expansion of 3′-chloroflavone 4′-*O*-*β*-D-(4′′-*O*-methyl)-glucopyranoside (**2a**).

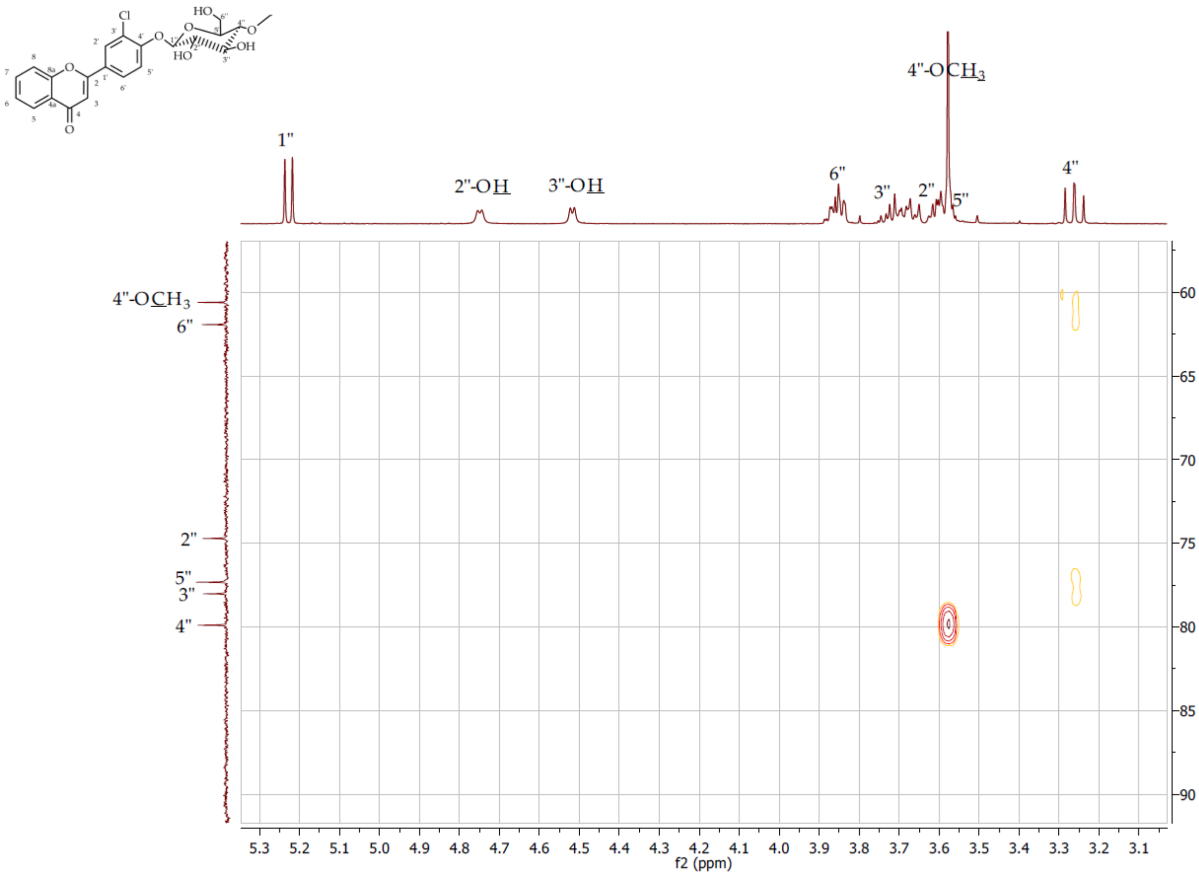

Figure S67. HMBC contour map – ^{1}H x ^{13}C expansion of 3′-chloroflavone 4′-*O*-*β*-D-(4′′-*O*-methyl)-glucopyranoside (**2a**).

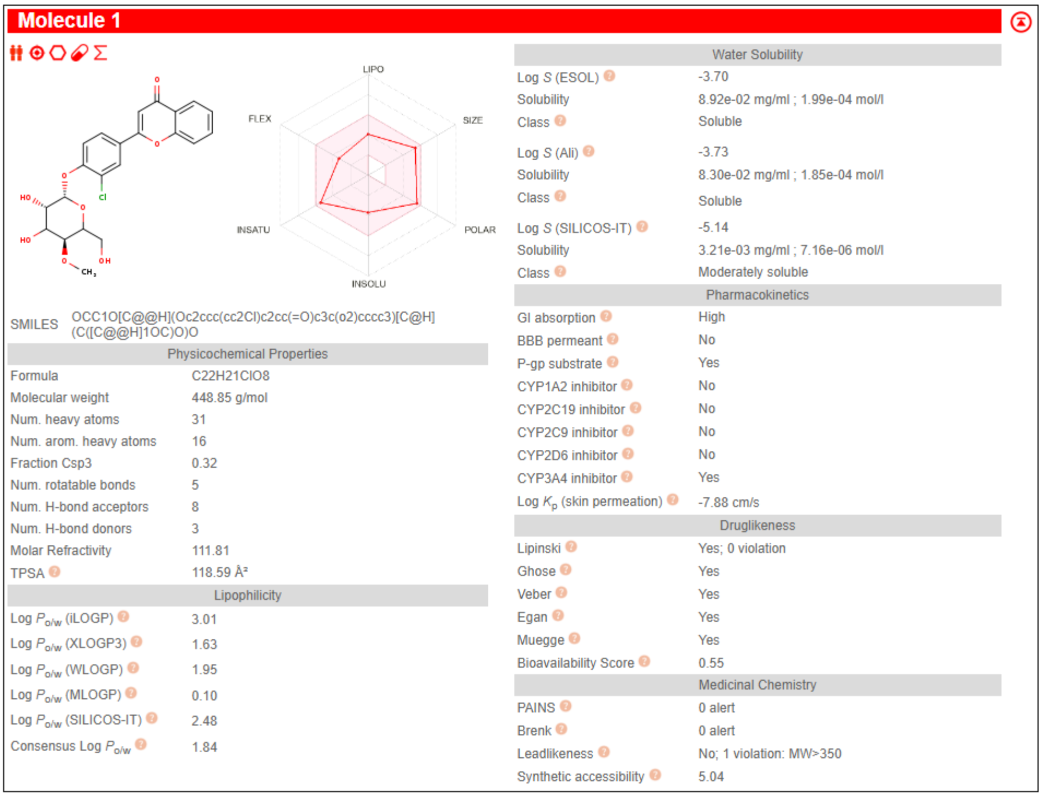

SMILES OCC1O[C@@H](Oc2ccc(cc2Cl)c2cc(=O)c3c(o2)cccc3)[C@H](C([C@@H]1OC)O)O

Physicochemical Properties	
Formula	C22H21ClO8
Molecular weight	448.85 g/mol
Num. heavy atoms	31
Num. arom. heavy atoms	16
Fraction Csp3	0.32
Num. rotatable bonds	5
Num. H-bond acceptors	8
Num. H-bond donors	3
Molar Refractivity	111.81
TPSA	118.59 Å²

Lipophilicity	
Log $P_{o/w}$ (iLOGP)	3.01
Log $P_{o/w}$ (XLOGP3)	1.63
Log $P_{o/w}$ (WLOGP)	1.95
Log $P_{o/w}$ (MLOGP)	0.10
Log $P_{o/w}$ (SILICOS-IT)	2.48
Consensus Log $P_{o/w}$	1.84

Water Solubility	
Log *S* (ESOL)	-3.70
Solubility	8.92e-02 mg/ml ; 1.99e-04 mol/l
Class	Soluble
Log *S* (Ali)	-3.73
Solubility	8.30e-02 mg/ml ; 1.85e-04 mol/l
Class	Soluble
Log *S* (SILICOS-IT)	-5.14
Solubility	3.21e-03 mg/ml ; 7.16e-06 mol/l
Class	Moderately soluble

Pharmacokinetics	
GI absorption	High
BBB permeant	No
P-gp substrate	Yes
CYP1A2 inhibitor	No
CYP2C19 inhibitor	No
CYP2C9 inhibitor	No
CYP2D6 inhibitor	No
CYP3A4 inhibitor	Yes
Log K_p (skin permeation)	-7.88 cm/s

Druglikeness	
Lipinski	Yes; 0 violation
Ghose	Yes
Veber	Yes
Egan	Yes
Muegge	Yes
Bioavailability Score	0.55

Medicinal Chemistry	
PAINS	0 alert
Brenk	0 alert
Leadlikeness	No; 1 violation: MW>350
Synthetic accessibility	5.04

Figure S68. 3′-Chloroflavone 4′-*O*-*β*-D-(4′′-*O*-methyl)-glucopyranoside (**2a**) physicochemical and ADME parameters prediction using the SwissADME modelling.

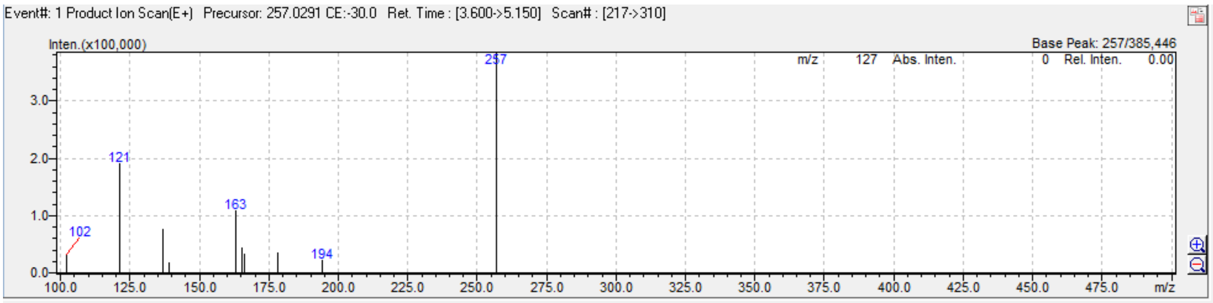

Figure S69. MS analysis of 4′-chloroflavone (**3**).

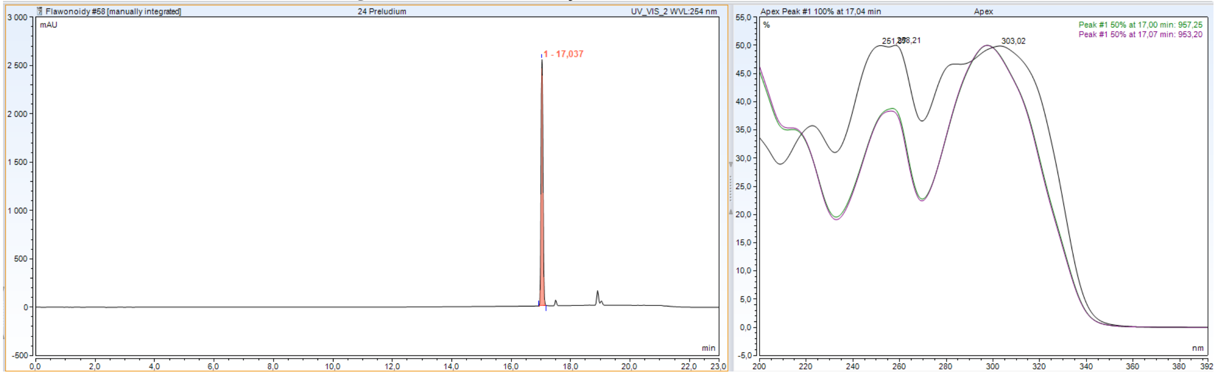

Figure S70. HPLC analysis of 4′-chloroflavone (**3**).

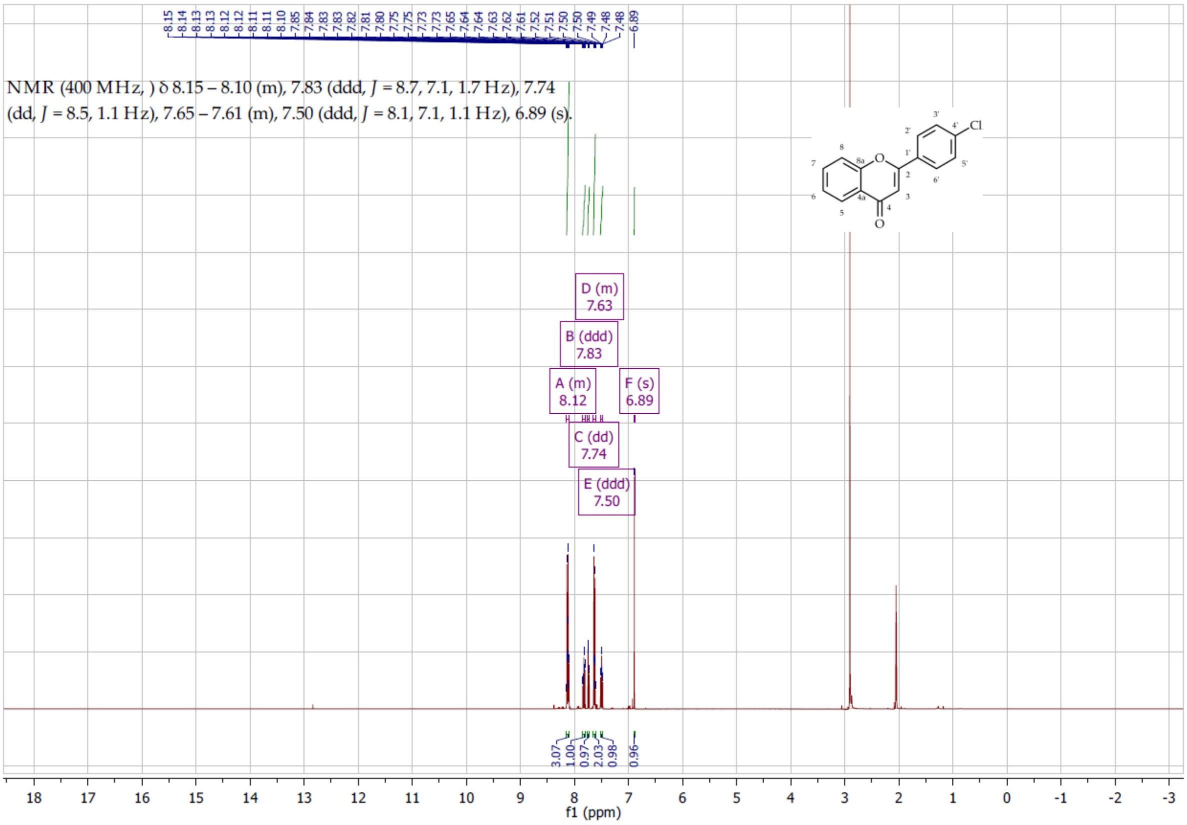

Figure S71. ^{1}H NMR spectrum (*δ*, acetone-d6, 600 MHz) of 4′-chloroflavone (**3**).

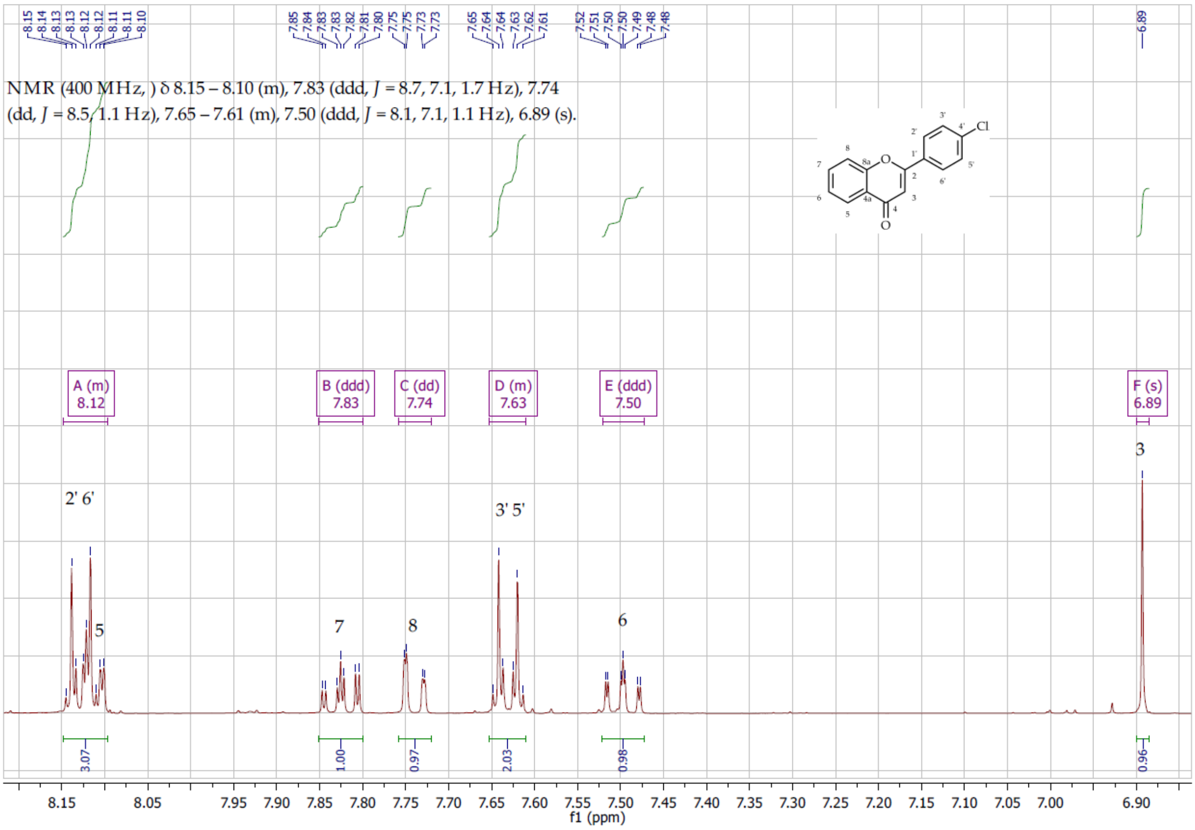

Figure S72. ^{1}H NMR spectrum expansion (δ, acetone-d6, 600 MHz) of 4′-chloroflavone (**3**).

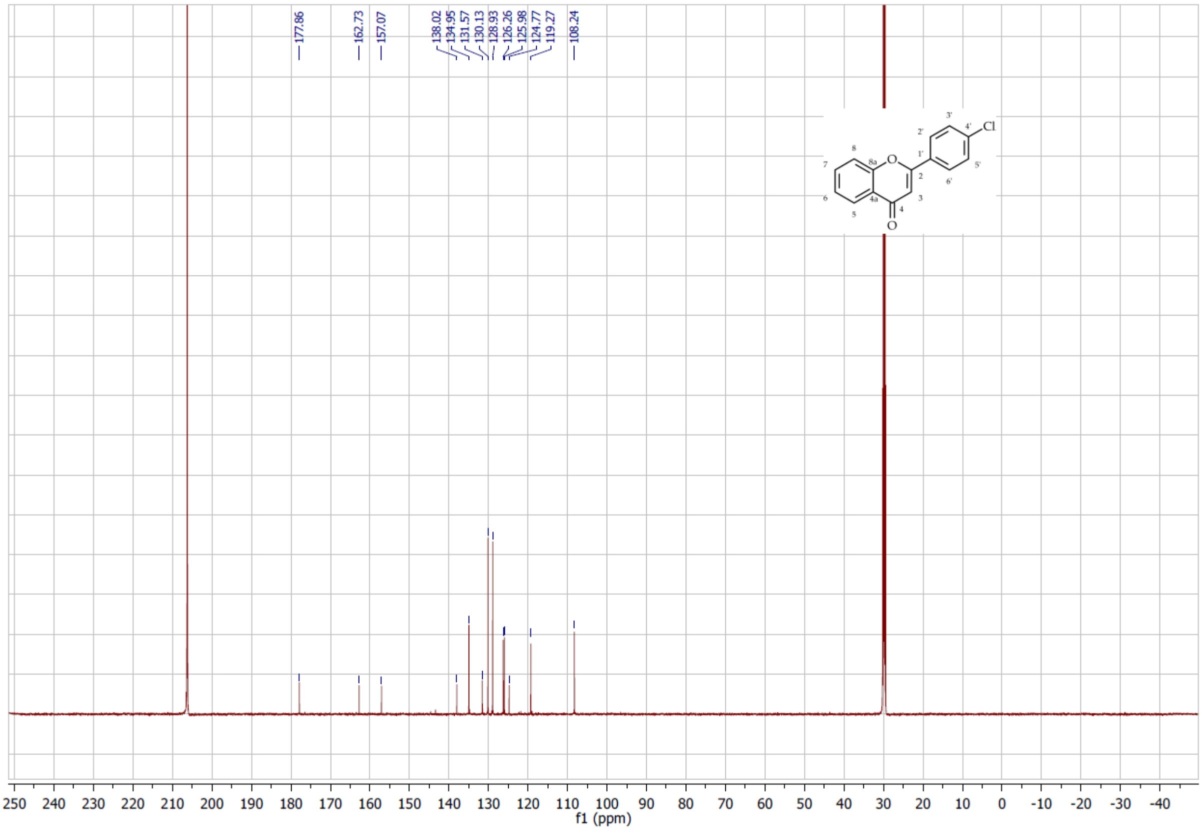

Figure S73. ^{13}C NMR spectrum (δ, acetone-d6, 151 MHz) of 4′-chloroflavone (**3**).

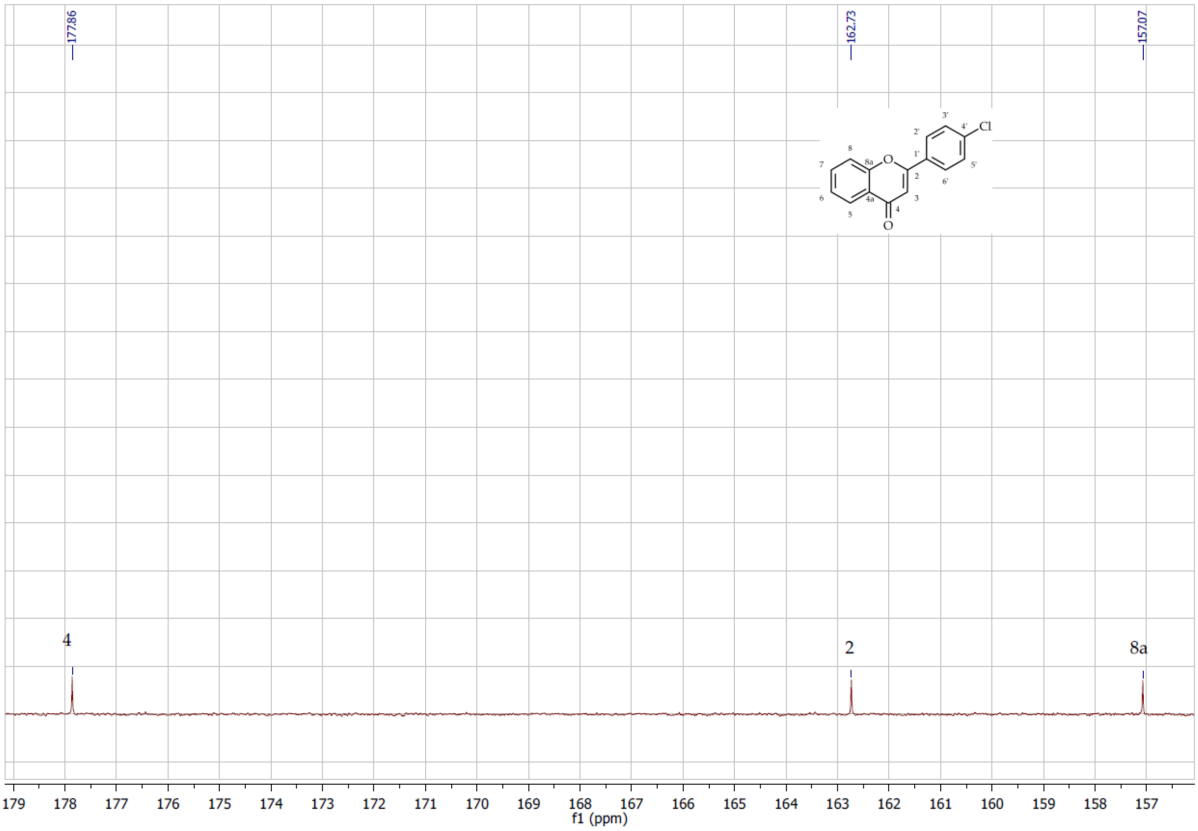

Figure S74. ^{13}C NMR spectrum expansion (δ, acetone-d6, 151 MHz) of 4′-chloroflavone (**3**).

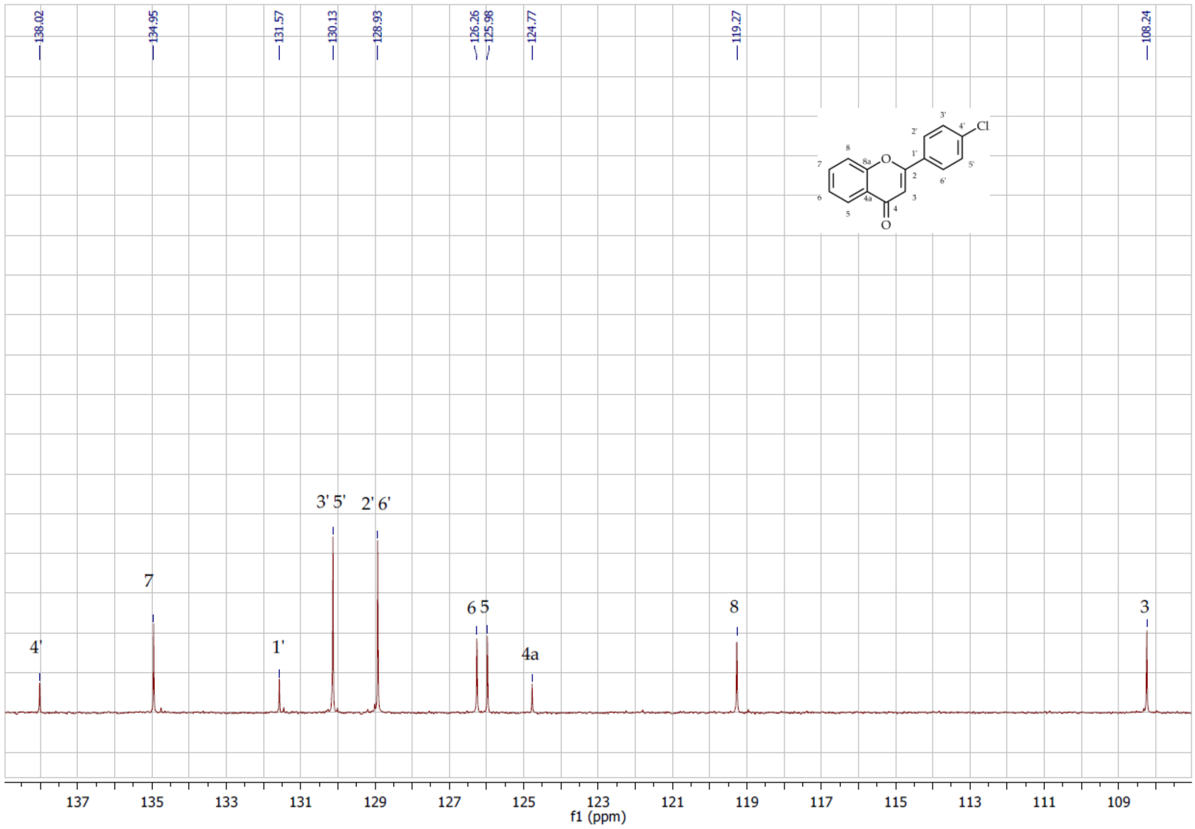

Figure S75. ^{13}C NMR spectrum expansion (δ, acetone-d6, 151 MHz) of 4′-chloroflavone (**3**).

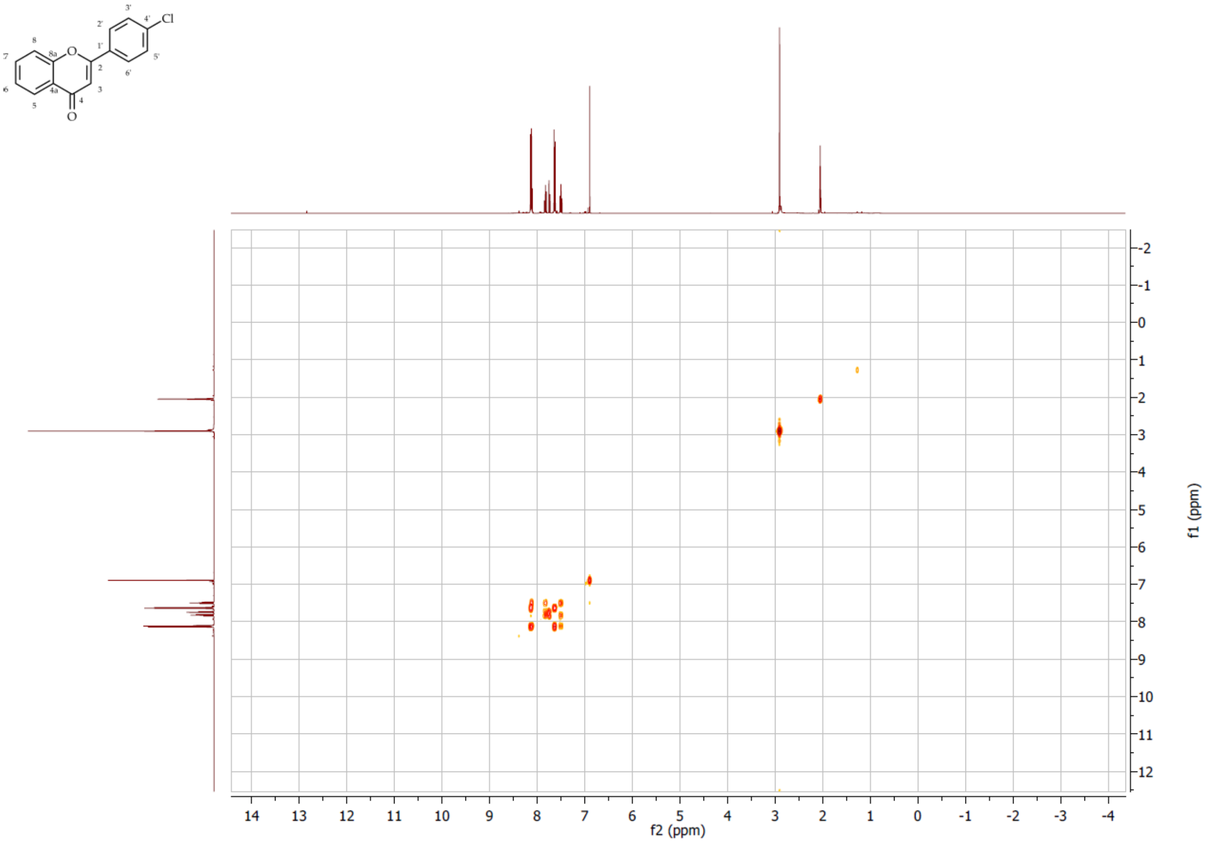

Figure S76. COSY contour map – ¹H x ¹H of 4′-chloroflavone (**3**).

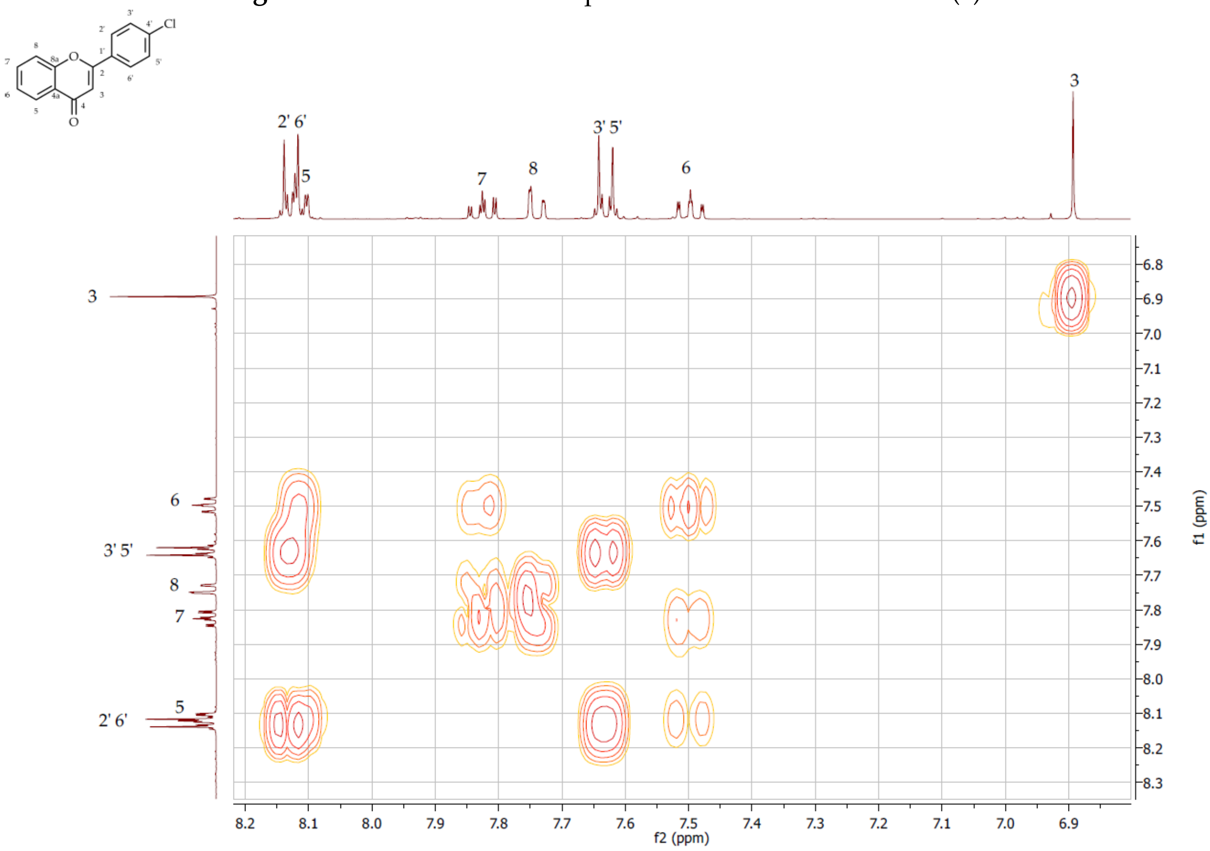

Figure S77. COSY contour map – ^{1}H x ^{1}H expansion of 4′-chloroflavone (**3**).

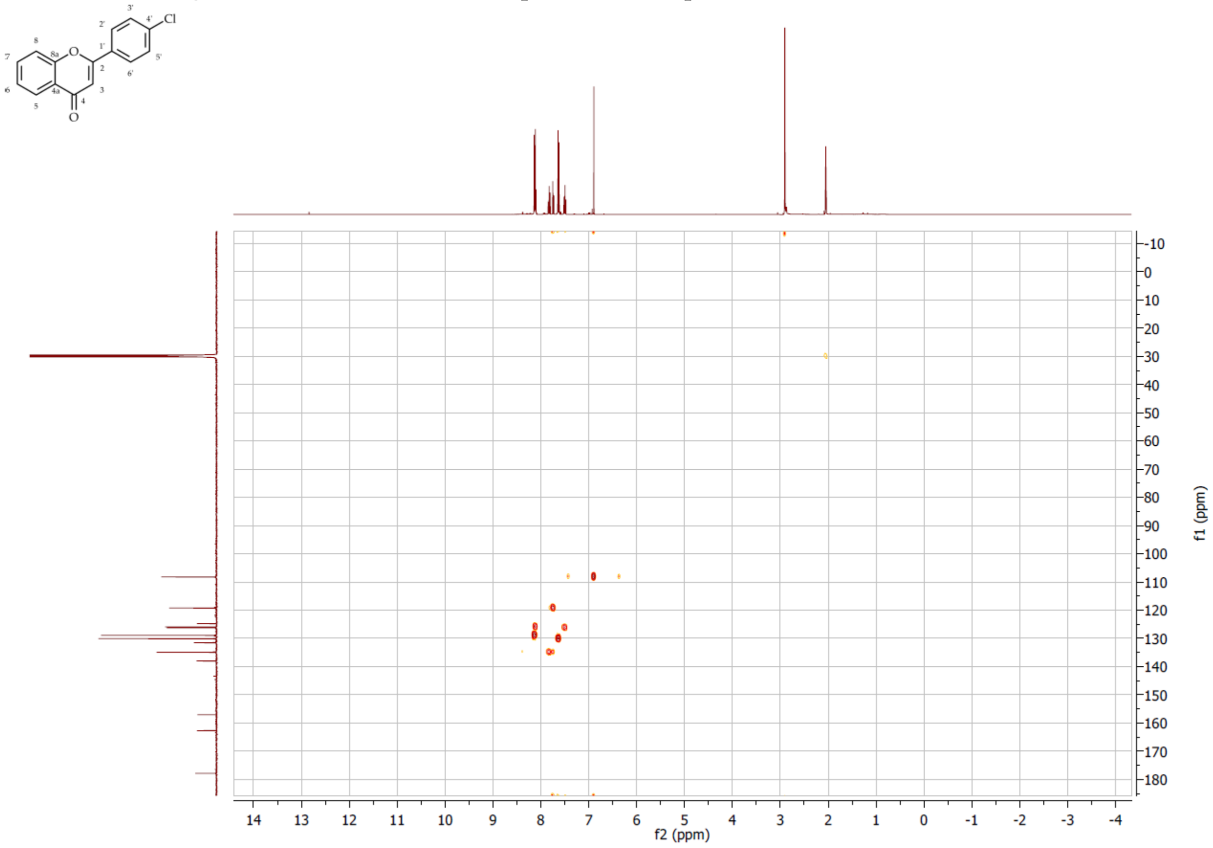

Figure S78. HMQC contour map – ^{1}H x ^{13}C of 4′-chloroflavone (**3**).

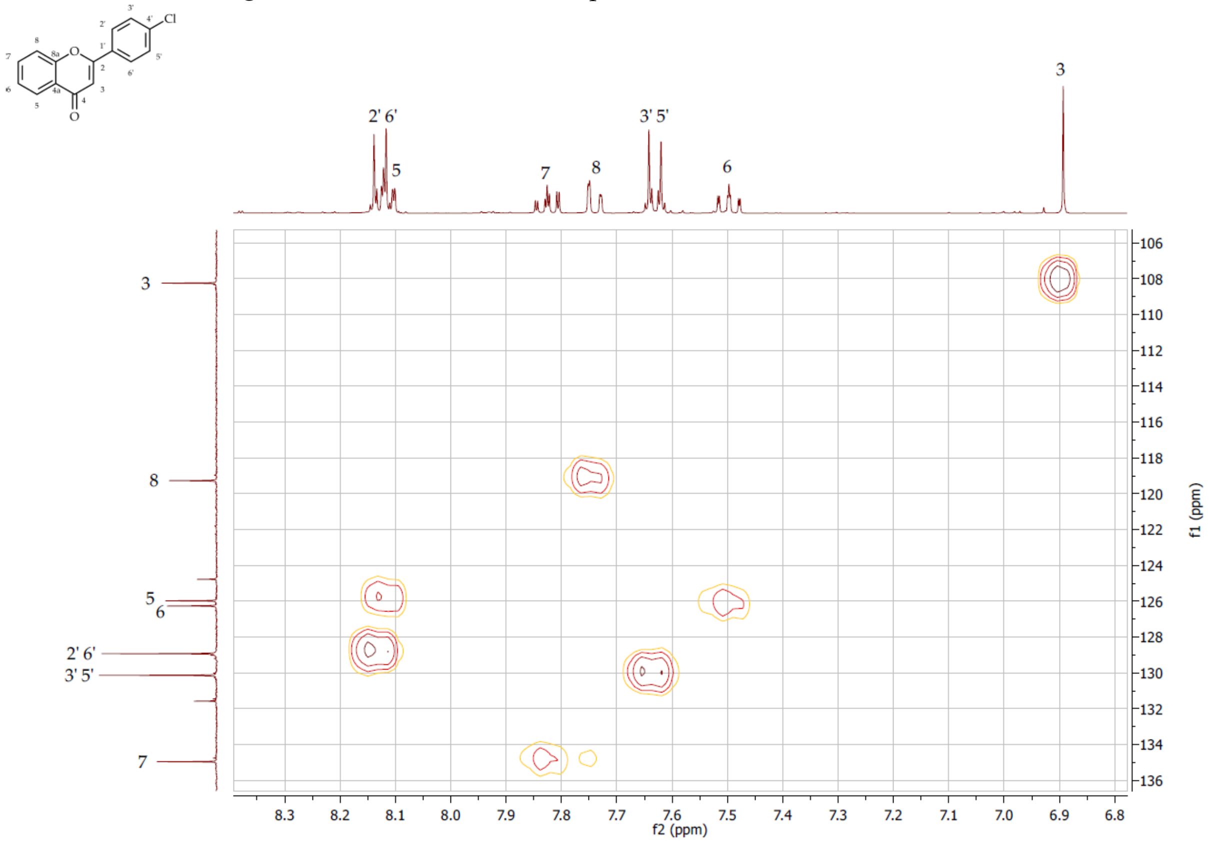

Figure S79. HMQC contour map – ^{1}H x ^{13}C expansion of 4′-chloroflavone (**3**).

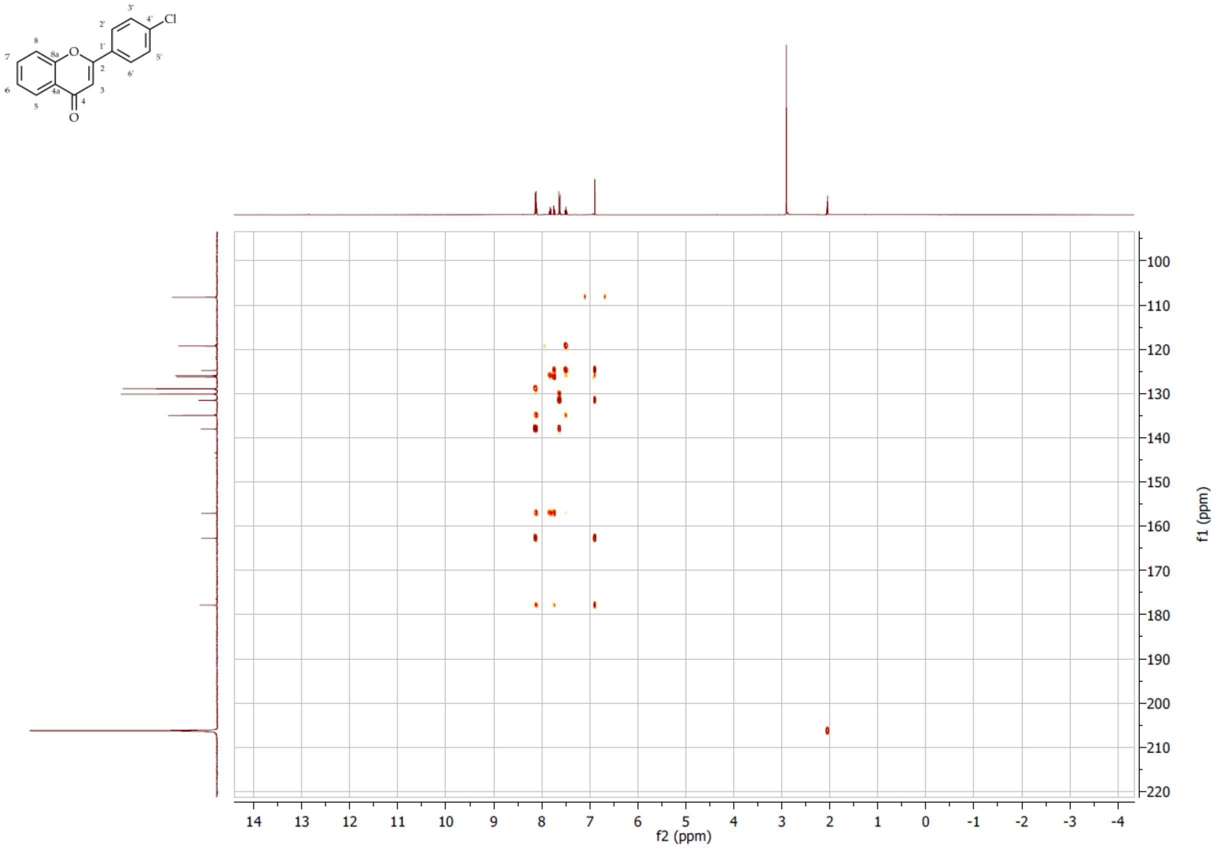

Figure S80. HMBC contour map – ^{1}H x ^{13}C of 4′-chloroflavone (**3**).

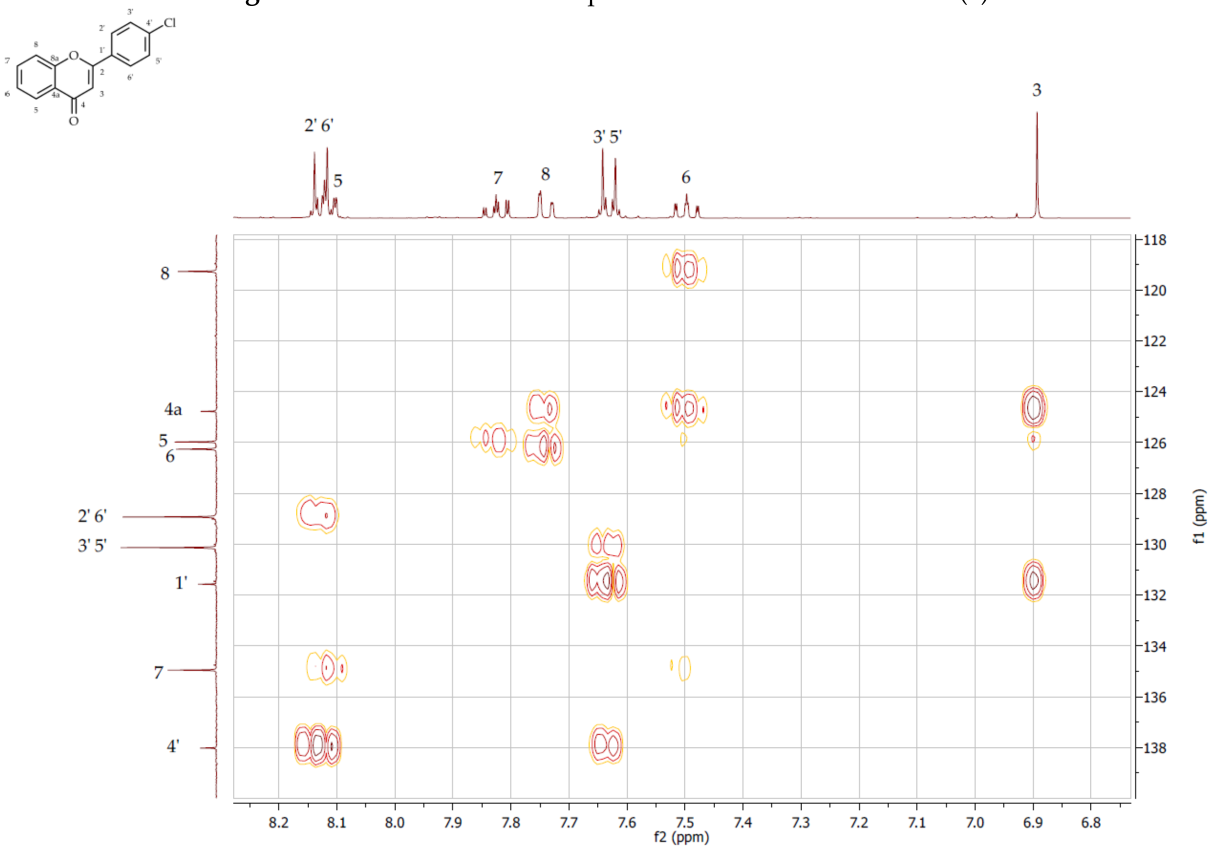

Figure S81. HMBC contour map – ^{1}H x ^{13}C expansion of 4′-chloroflavone (**3**).

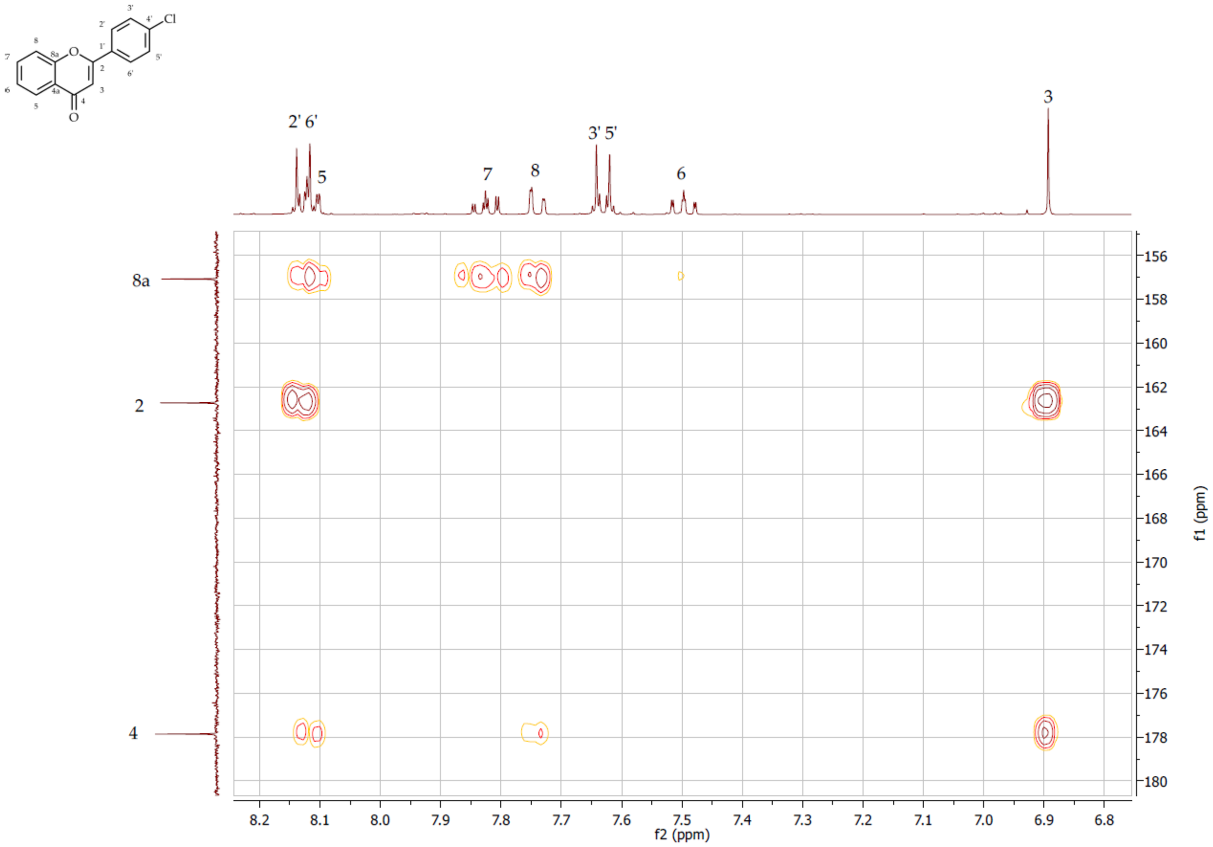

Figure S82. HMBC contour map – $^1H \times ^{13}C$ expansion of 4′-chloroflavone (**3**).

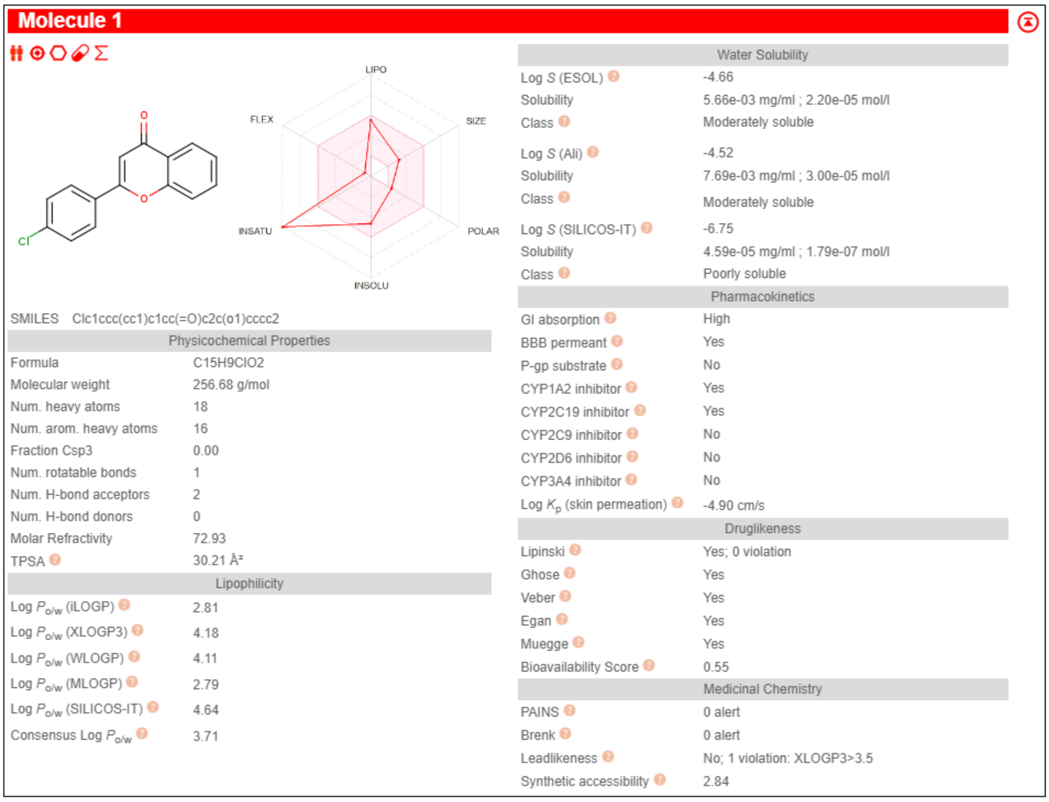

SMILES Clc1ccc(cc1)c1cc(=O)c2c(o1)cccc2

Physicochemical Properties	
Formula	C15H9ClO2
Molecular weight	256.68 g/mol
Num. heavy atoms	18
Num. arom. heavy atoms	16
Fraction Csp3	0.00
Num. rotatable bonds	1
Num. H-bond acceptors	2
Num. H-bond donors	0
Molar Refractivity	72.93
TPSA	30.21 Å²

Lipophilicity	
Log $P_{o/w}$ (iLOGP)	2.81
Log $P_{o/w}$ (XLOGP3)	4.18
Log $P_{o/w}$ (WLOGP)	4.11
Log $P_{o/w}$ (MLOGP)	2.79
Log $P_{o/w}$ (SILICOS-IT)	4.64
Consensus Log $P_{o/w}$	3.71

Water Solubility	
Log *S* (ESOL)	-4.66
Solubility	5.66e-03 mg/ml ; 2.20e-05 mol/l
Class	Moderately soluble
Log *S* (Ali)	-4.52
Solubility	7.69e-03 mg/ml ; 3.00e-05 mol/l
Class	Moderately soluble
Log *S* (SILICOS-IT)	-6.75
Solubility	4.59e-05 mg/ml ; 1.79e-07 mol/l
Class	Poorly soluble

Pharmacokinetics	
GI absorption	High
BBB permeant	Yes
P-gp substrate	No
CYP1A2 inhibitor	Yes
CYP2C19 inhibitor	Yes
CYP2C9 inhibitor	No
CYP2D6 inhibitor	No
CYP3A4 inhibitor	No
Log K_p (skin permeation)	-4.90 cm/s

Druglikeness	
Lipinski	Yes; 0 violation
Ghose	Yes
Veber	Yes
Egan	Yes
Muegge	Yes
Bioavailability Score	0.55

Medicinal Chemistry	
PAINS	0 alert
Brenk	0 alert
Leadlikeness	No; 1 violation: XLOGP3>3.5
Synthetic accessibility	2.84

Figure S83. 4′-Chloroflavone (**3**) physicochemical and ADME parameters prediction using the SwissADME modelling.

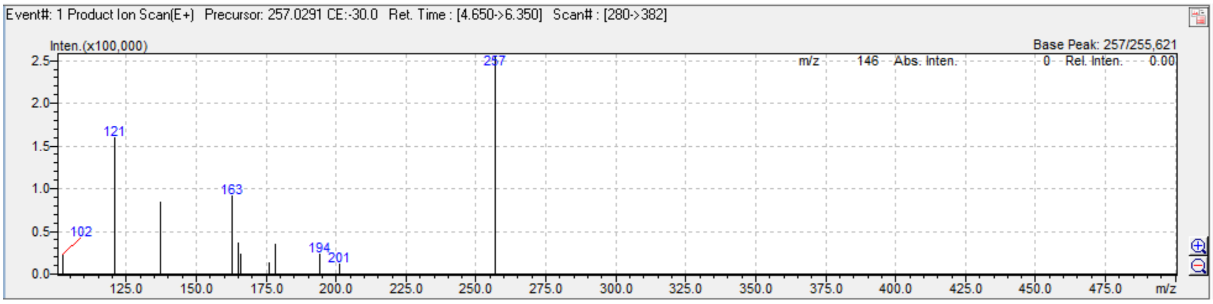

Figure S84. MS analysis of 6-chloroflavone (**4**).

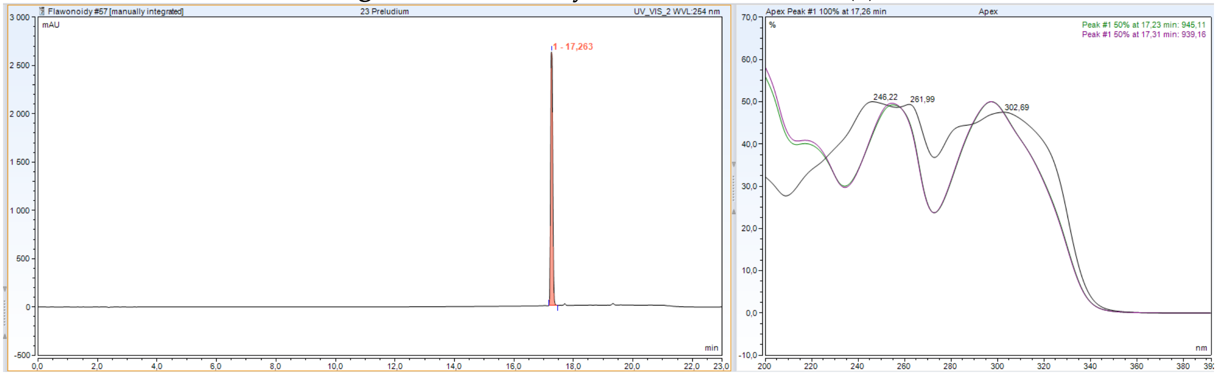

Figure S85. HPLC analysis of 6-chloroflavone (**4**).

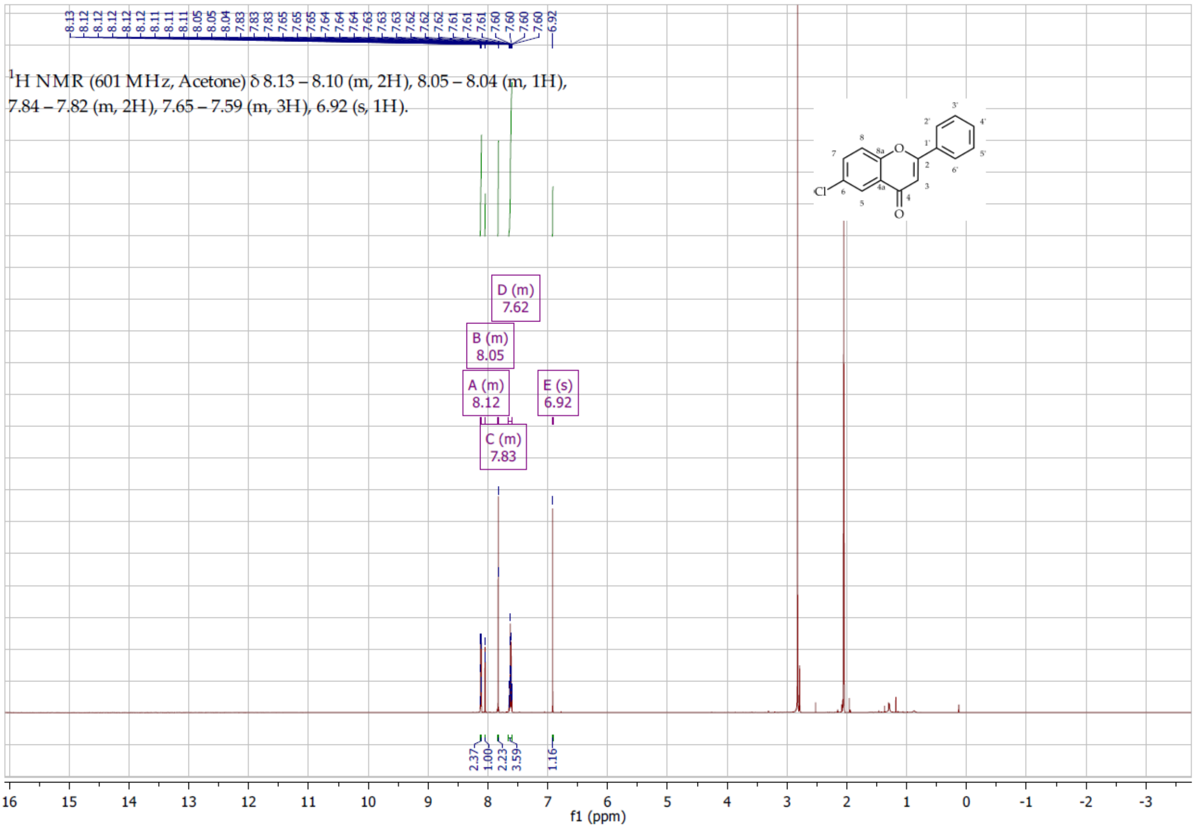

Figure S86. ¹H NMR spectrum (*δ*, acetone-d6, 600 MHz) of 6-chloroflavone (**4**).

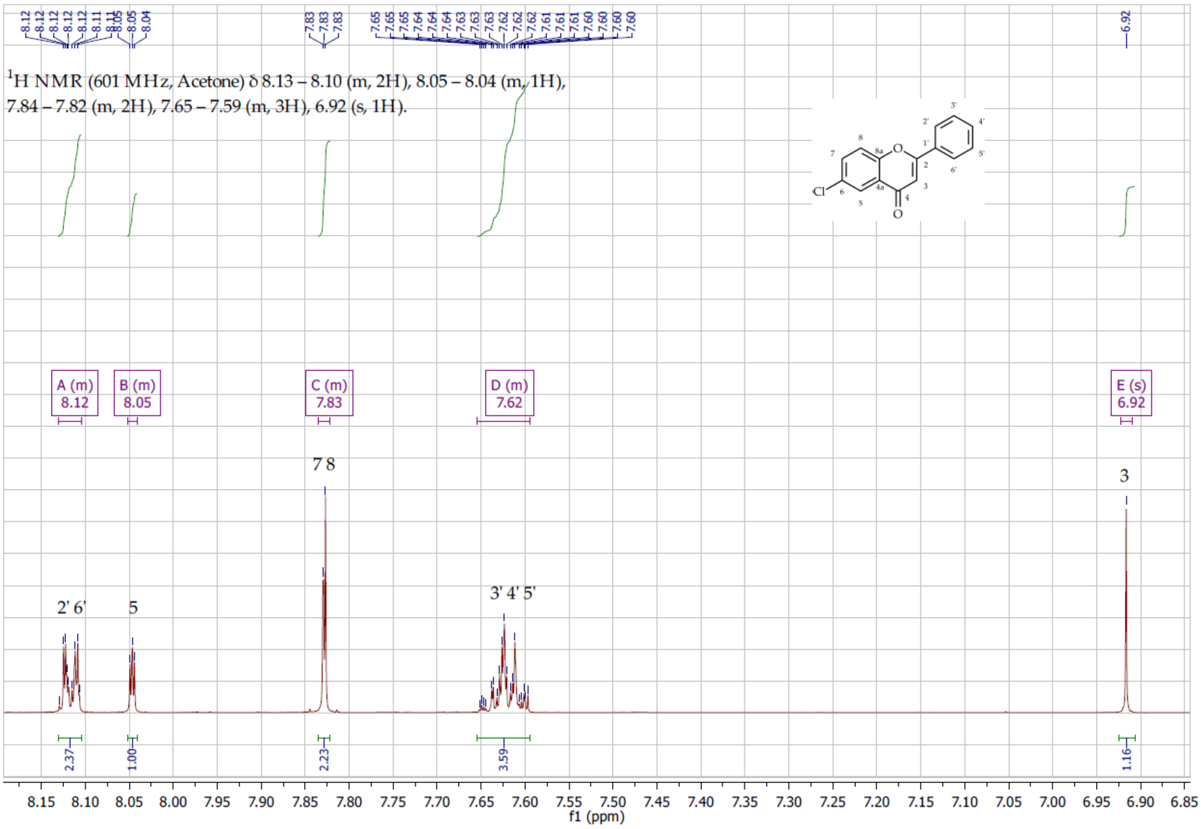

Figure S87. ^{1}H NMR spectrum expansion (*δ*, acetone-d6, 600 MHz) of 6-chloroflavone (**4**).

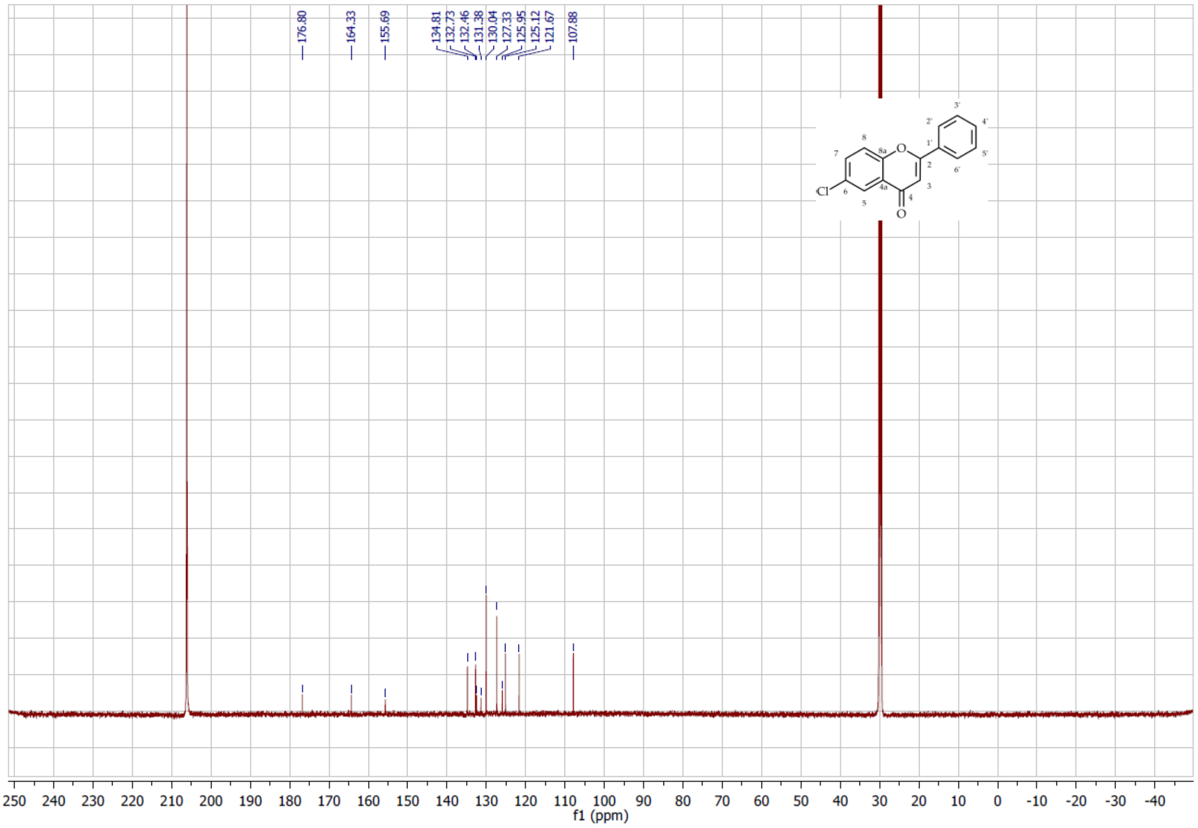

Figure S88. ^{13}C NMR spectrum (*δ*, acetone-d6, 151 MHz) of 6-chloroflavone (**4**).

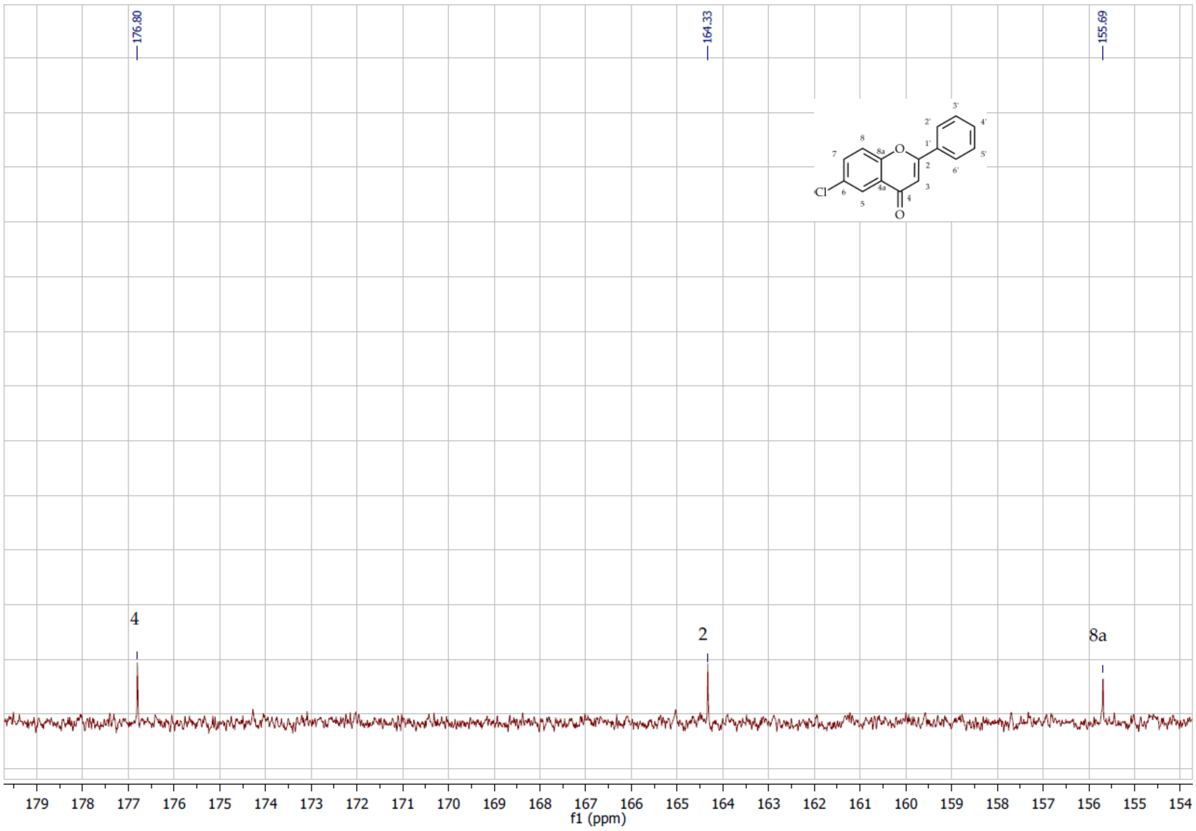

Figure S89. ^{13}C NMR spectrum expansion (δ, acetone-d6, 151 MHz) of 6-chloroflavone (**4**).

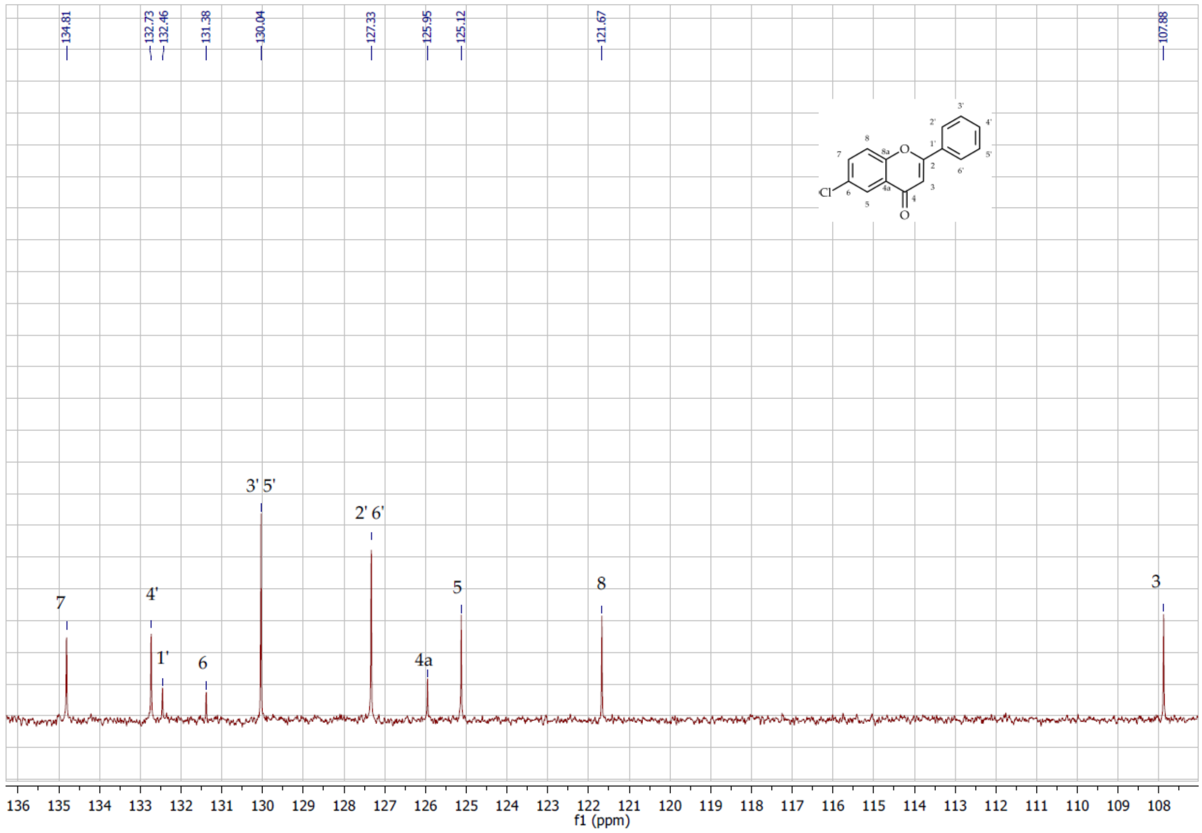

Figure S90. ^{13}C NMR spectrum expansion (δ, acetone-d6, 151 MHz) of 6-chloroflavone (**4**).

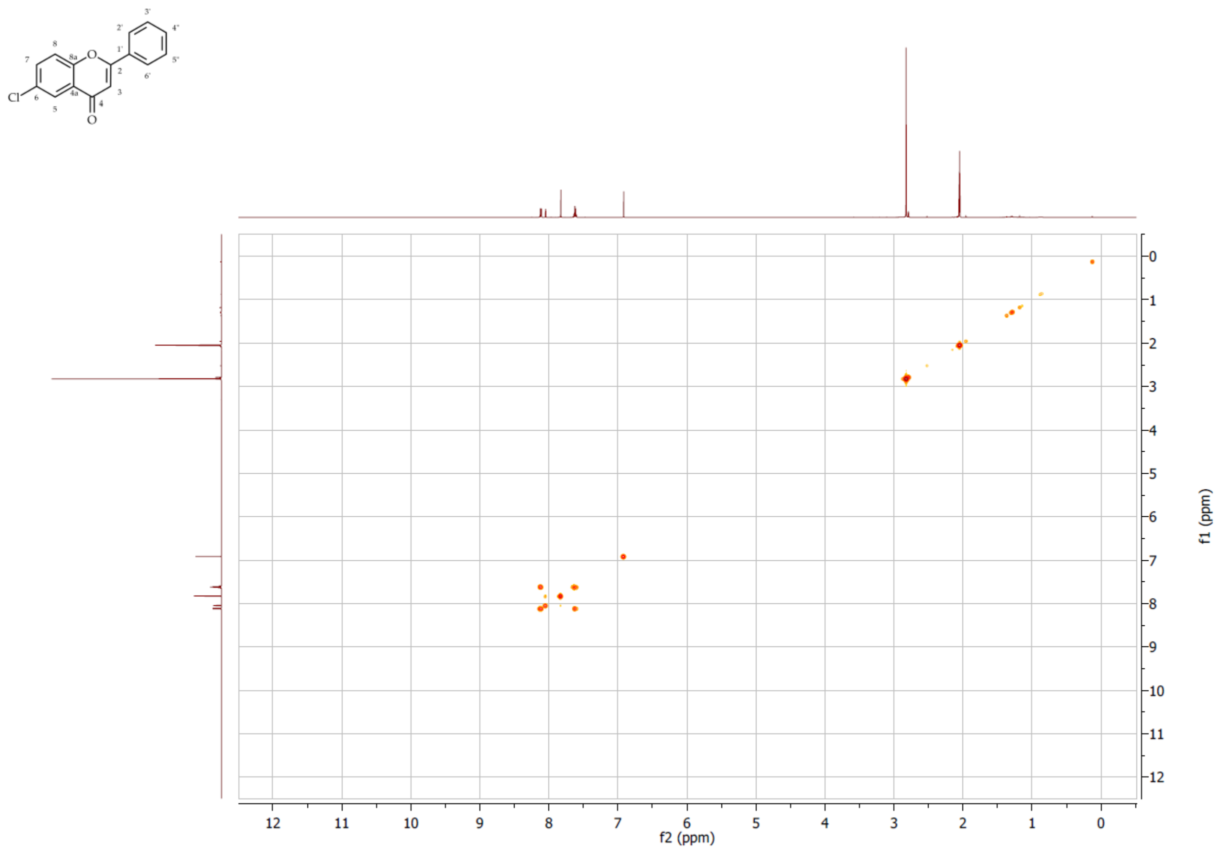

Figure S91. COSY contour map – ^{1}H x ^{1}H of 6-chloroflavone (**4**).

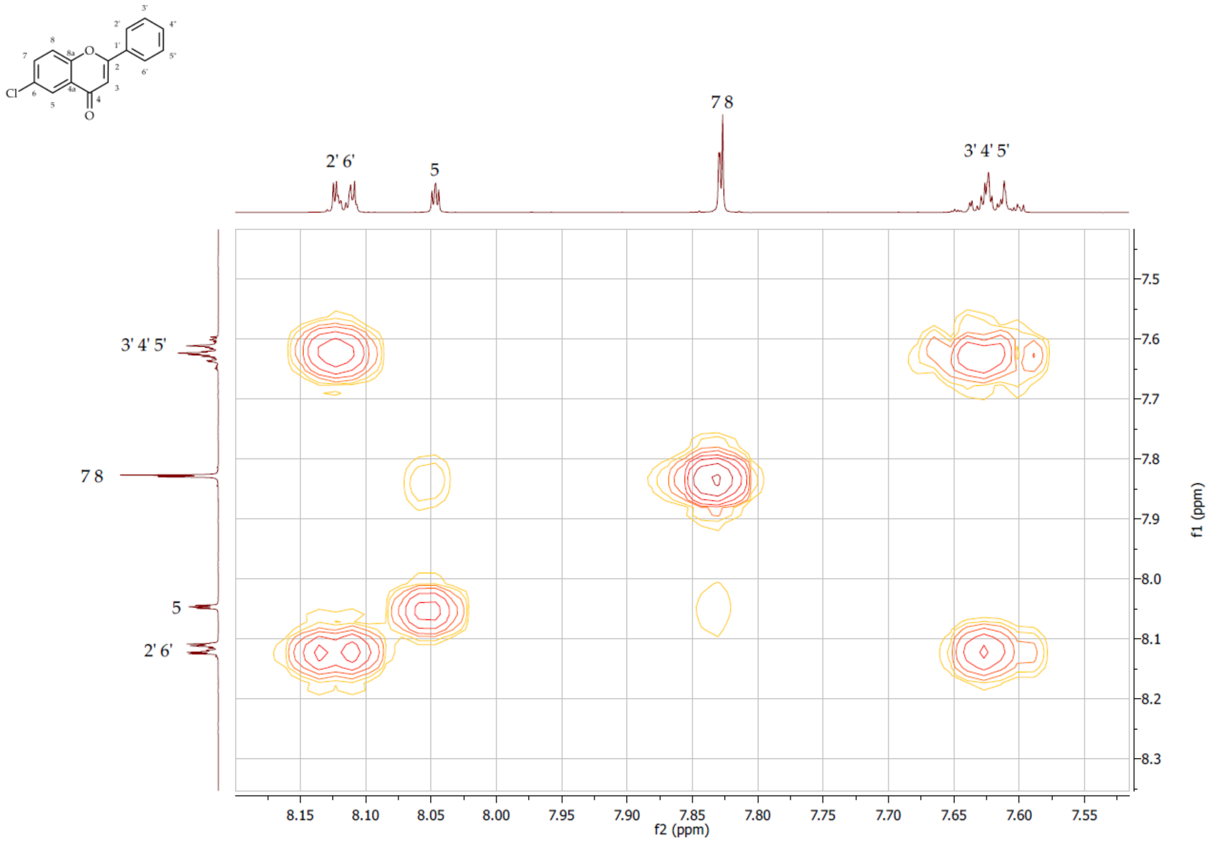

Figure S92. COSY contour map – ^{1}H x ^{1}H expansion of 6-chloroflavone (**4**).

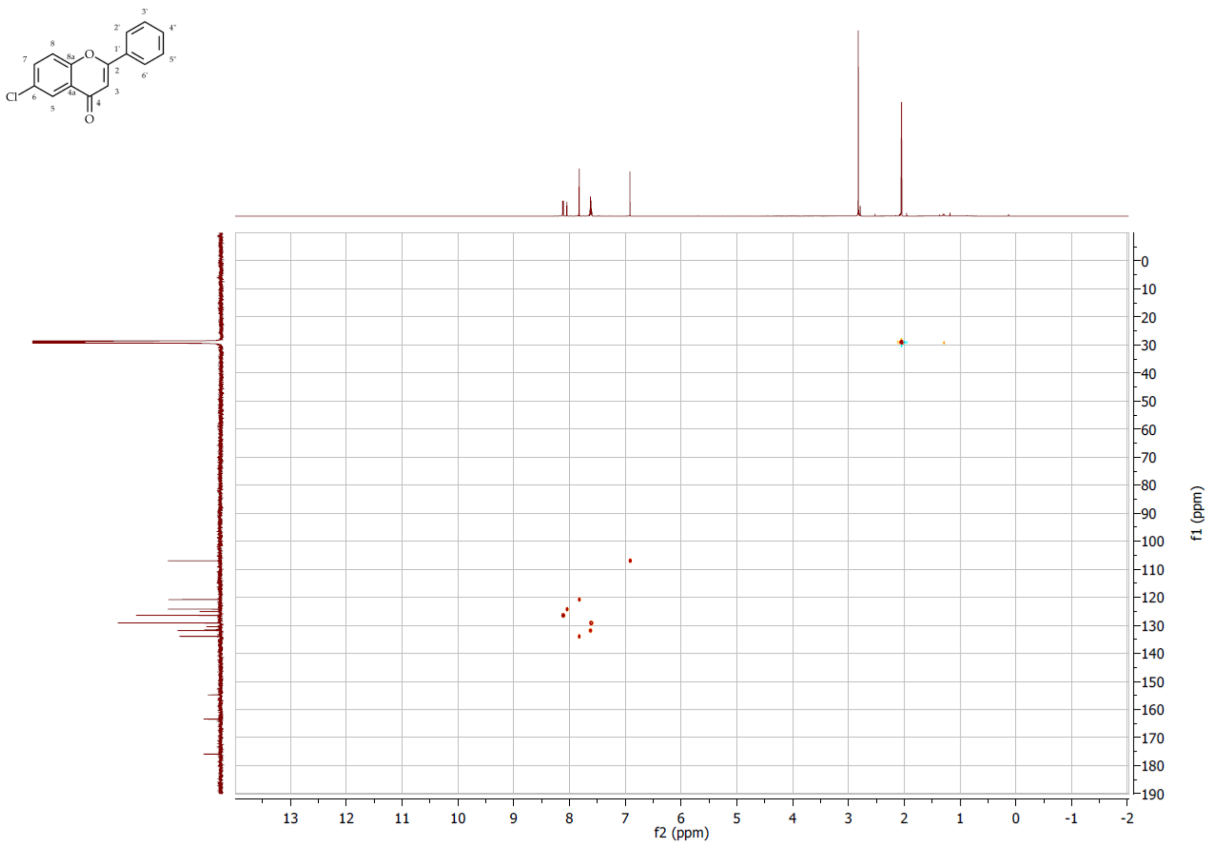

Figure S93. HMQC contour map – ^1H x ^{13}C of 6-chloroflavone (**4**).

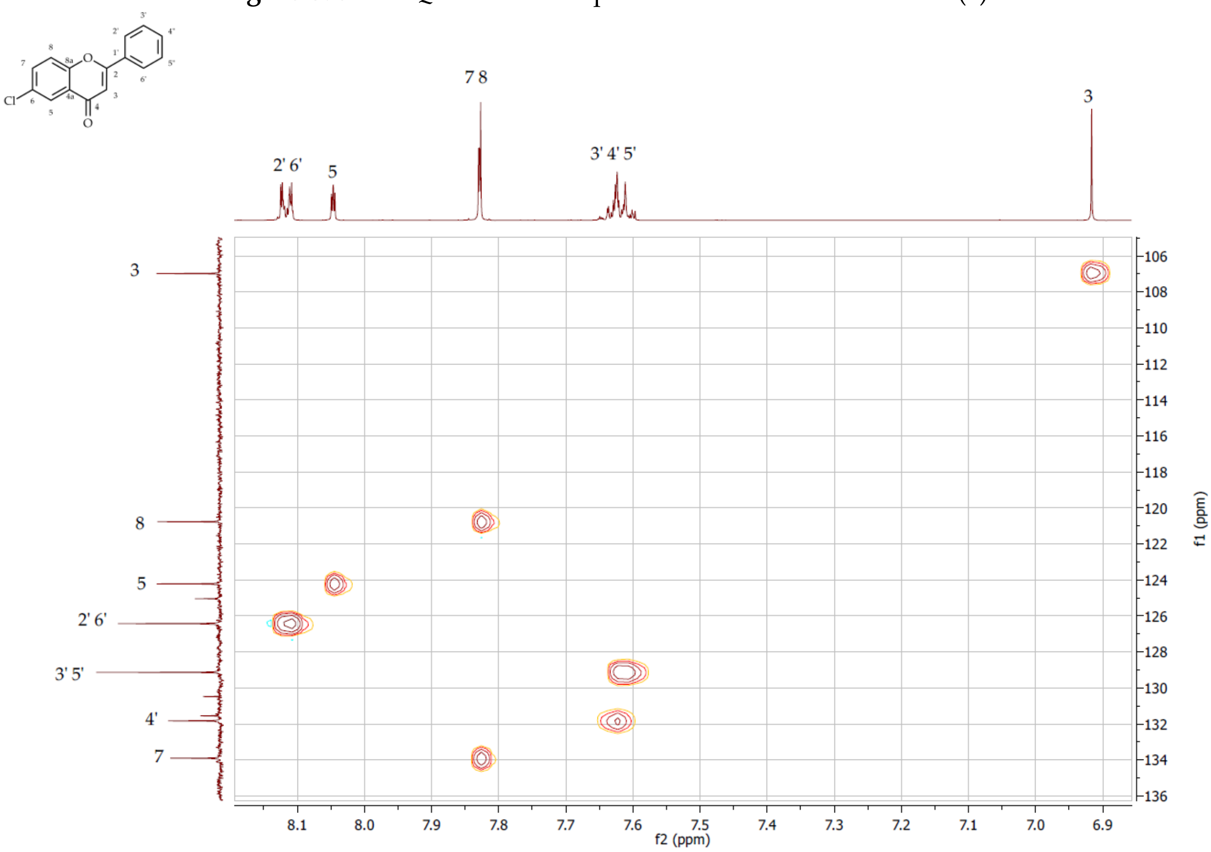

Figure S94. HMQC contour map – ^1H x ^{13}C expansion of 6-chloroflavone (**4**).

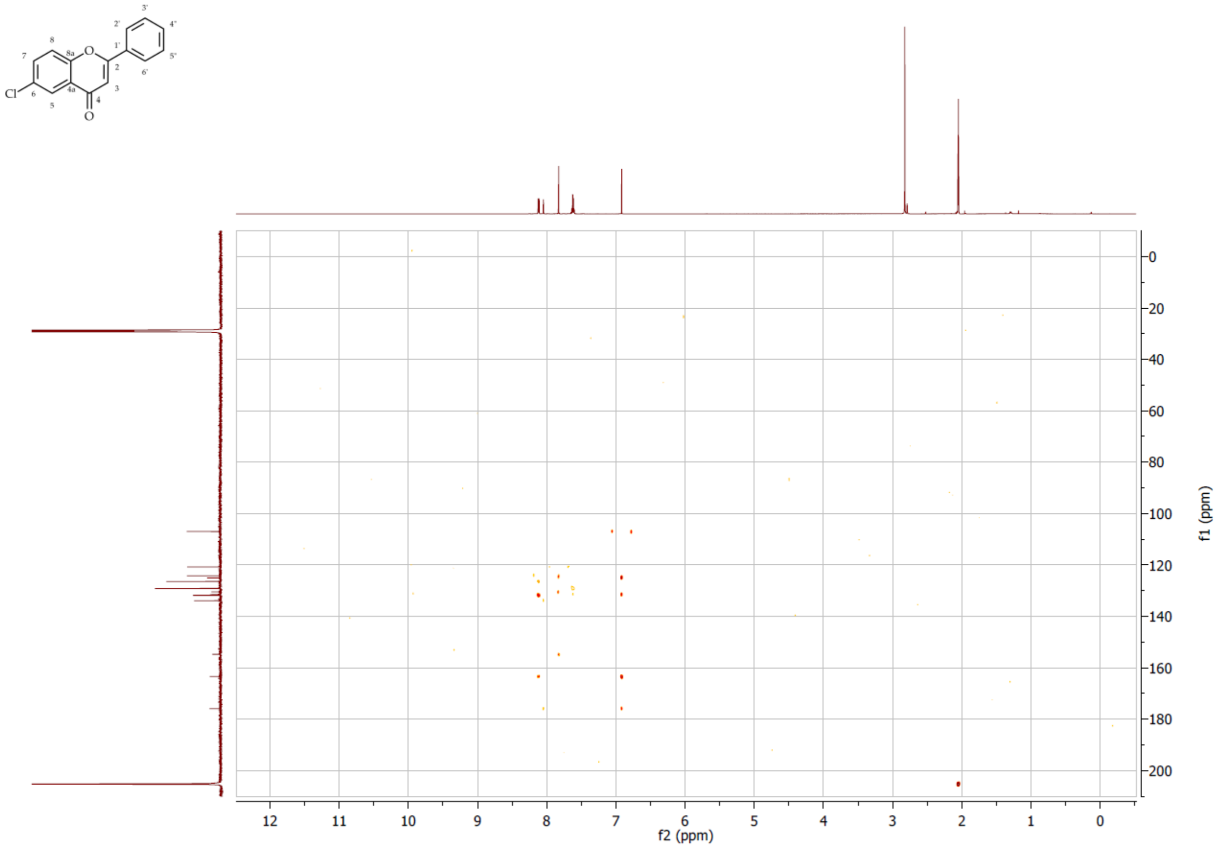

Figure S95. HMBC contour map – ^{1}H x ^{13}C of 6-chloroflavone (**4**).

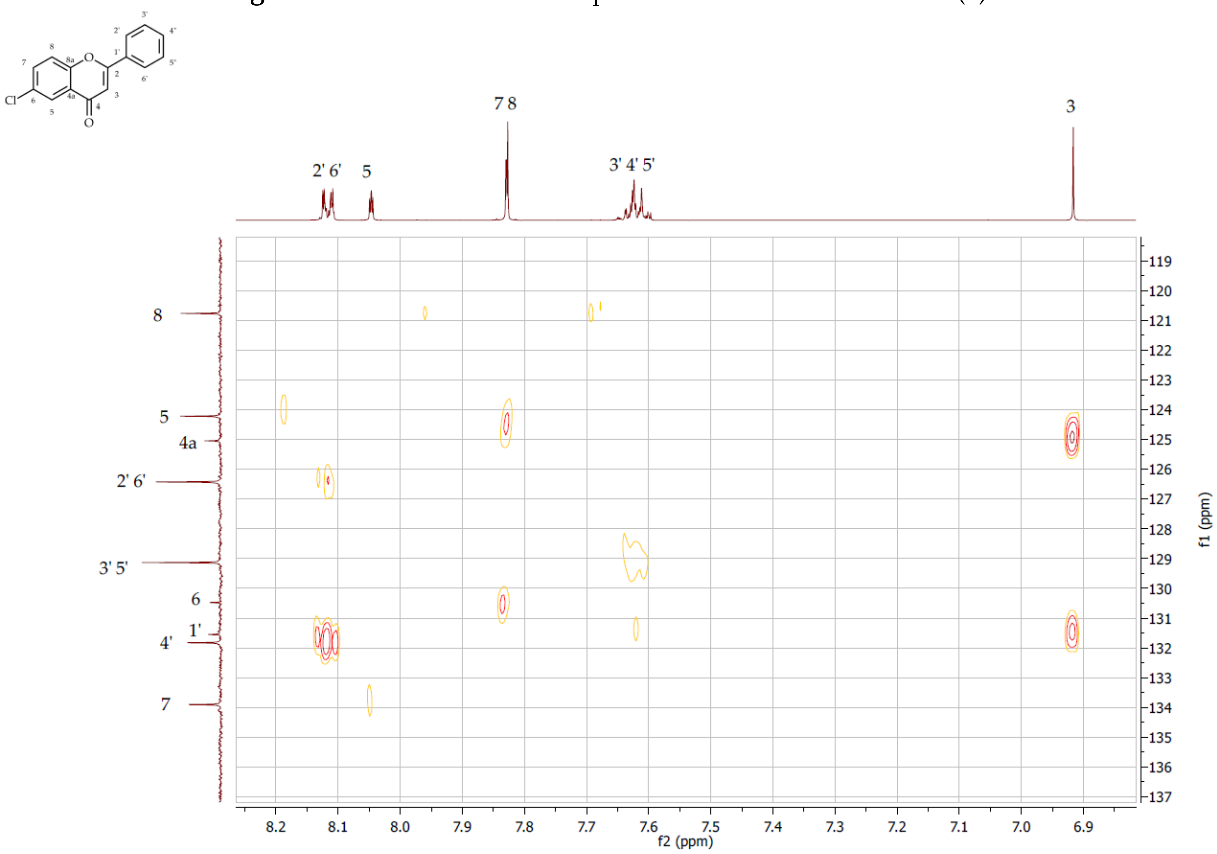

Figure S96. HMBC contour map – ^{1}H x ^{13}C expansion of 6-chloroflavone (**4**).

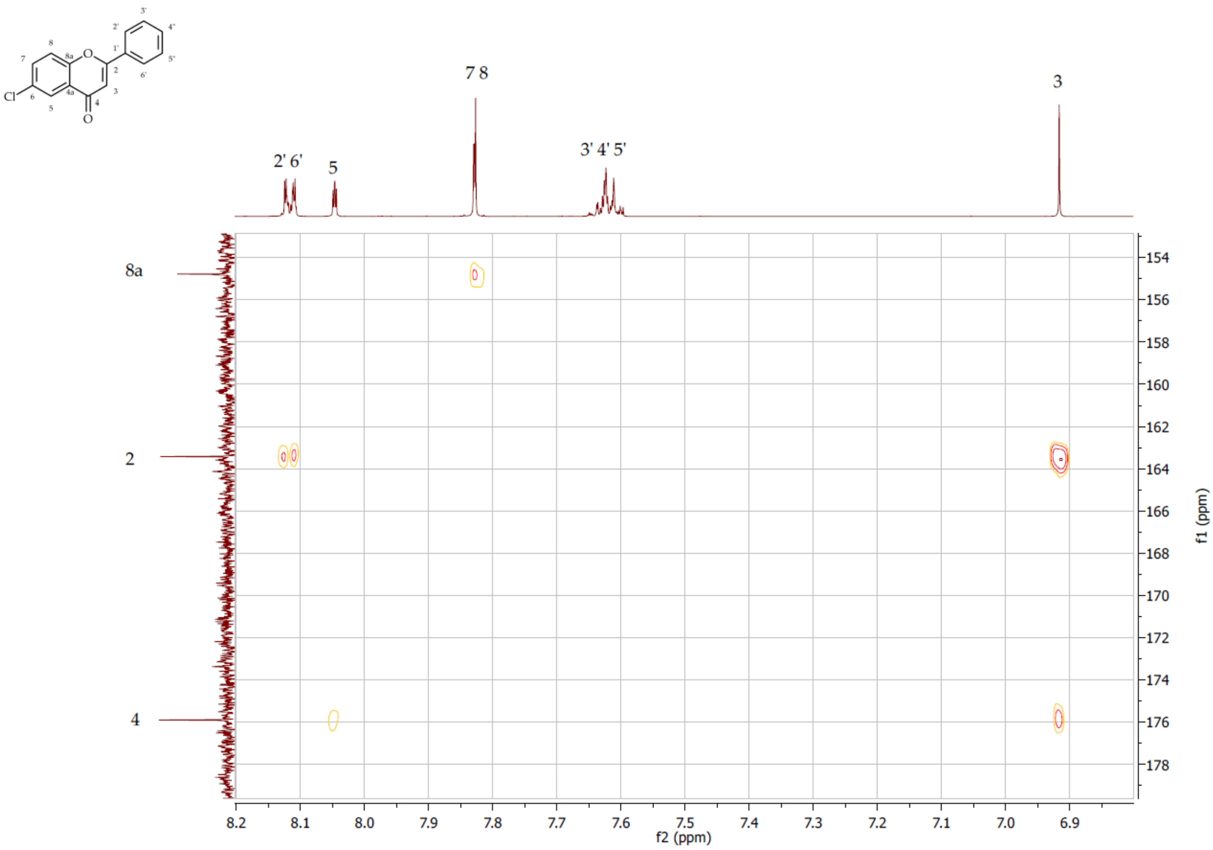

Figure S97. HMBC contour map – ^{1}H x ^{13}C expansion of 6-chloroflavone (**4**).

Molecule 1

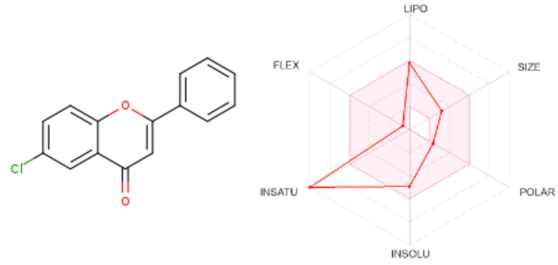

SMILES Clc1ccc2c(c1)c(=O)cc(o2)c1ccccc1

Physicochemical Properties	
Formula	C15H9ClO2
Molecular weight	256.68 g/mol
Num. heavy atoms	18
Num. arom. heavy atoms	16
Fraction Csp3	0.00
Num. rotatable bonds	1
Num. H-bond acceptors	2
Num. H-bond donors	0
Molar Refractivity	72.93
TPSA	30.21 Å²

Lipophilicity	
Log $P_{o/w}$ (iLOGP)	2.77
Log $P_{o/w}$ (XLOGP3)	4.60
Log $P_{o/w}$ (WLOGP)	4.11
Log $P_{o/w}$ (MLOGP)	2.79
Log $P_{o/w}$ (SILICOS-IT)	4.64
Consensus Log $P_{o/w}$	3.78

Water Solubility	
Log *S* (ESOL)	-4.92
Solubility	3.08e-03 mg/ml ; 1.20e-05 mol/l
Class	Moderately soluble
Log *S* (Ali)	-4.96
Solubility	2.82e-03 mg/ml ; 1.10e-05 mol/l
Class	Moderately soluble
Log *S* (SILICOS-IT)	-6.75
Solubility	4.59e-05 mg/ml ; 1.79e-07 mol/l
Class	Poorly soluble

Pharmacokinetics	
GI absorption	High
BBB permeant	Yes
P-gp substrate	No
CYP1A2 inhibitor	Yes
CYP2C19 inhibitor	Yes
CYP2C9 inhibitor	Yes
CYP2D6 inhibitor	No
CYP3A4 inhibitor	No
Log K_p (skin permeation)	-4.60 cm/s

Druglikeness	
Lipinski	Yes; 0 violation
Ghose	Yes
Veber	Yes
Egan	Yes
Muegge	Yes
Bioavailability Score	0.55

Medicinal Chemistry	
PAINS	0 alert
Brenk	0 alert
Leadlikeness	No; 1 violation: XLOGP3>3.5
Synthetic accessibility	2.76

Figure S98. 6-Chloroflavone (**4**) physicochemical and ADME parameters prediction using the SwissADME modelling.

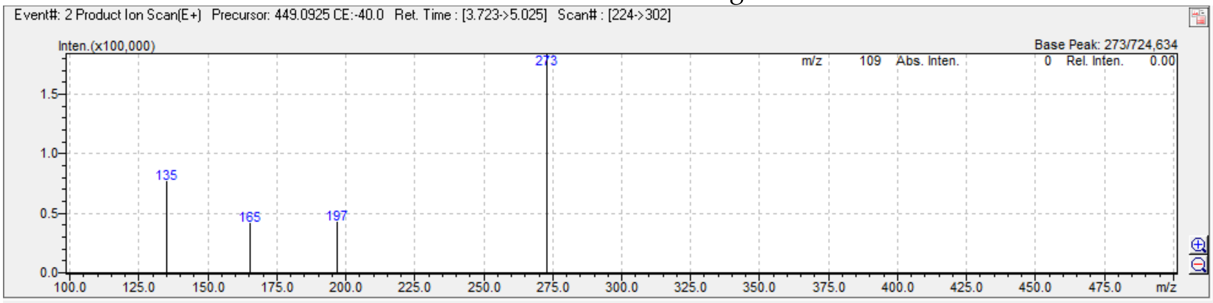

Figure S99. MS analysis of 6-chloroflavone 3′-*O*-*β*-D-(4′′-*O*-methyl)-glucopyranoside (**4a**).

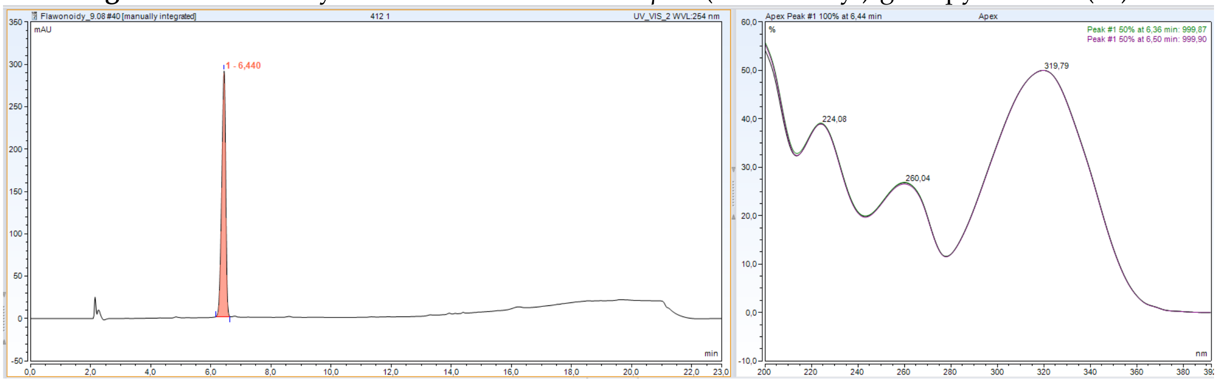

Figure S100. HPLC analysis of 6-chloroflavone 3′-*O*-*β*-D-(4′′-*O*-methyl)-glucopyranoside (**4a**).

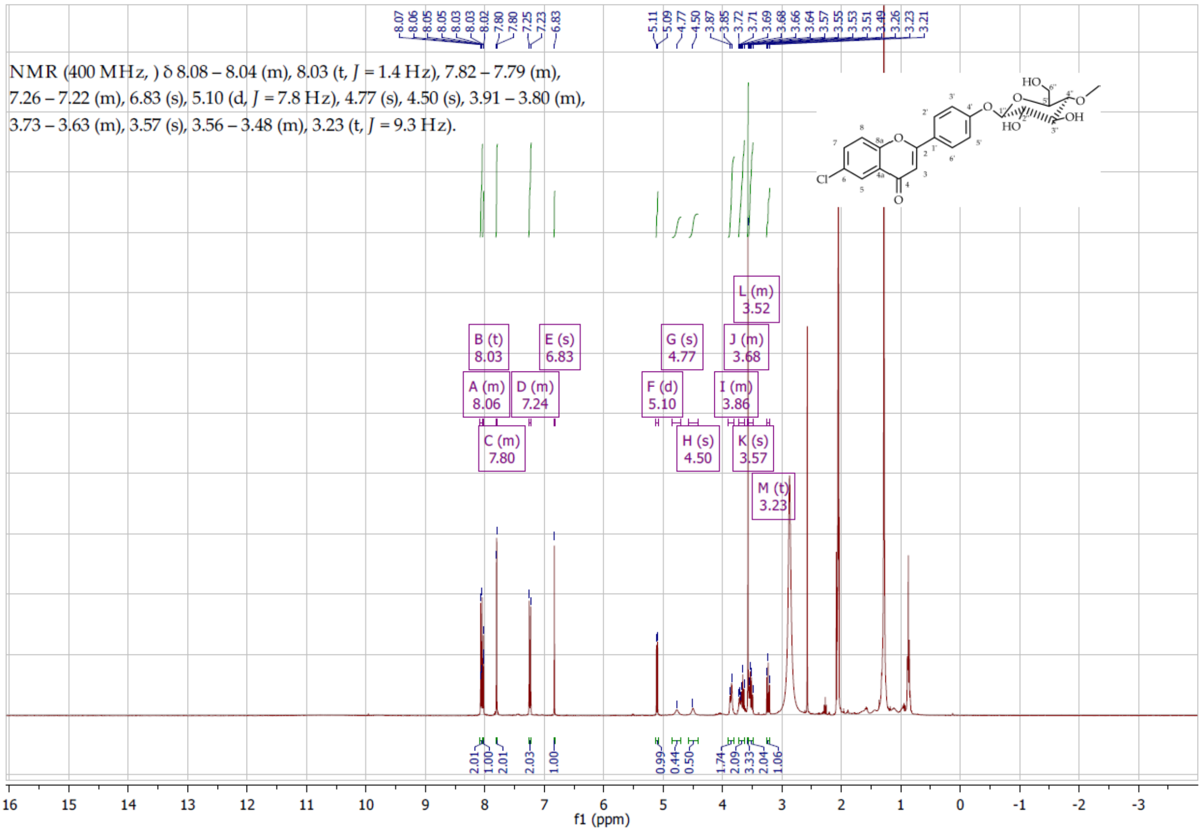

Figure S101. ^{1}H NMR spectrum (*δ*, acetone-d6, 600 MHz) of 6-chloroflavone 3′-*O*-*β*-D-(4′′-*O*-methyl)-glucopyranoside (**4a**).

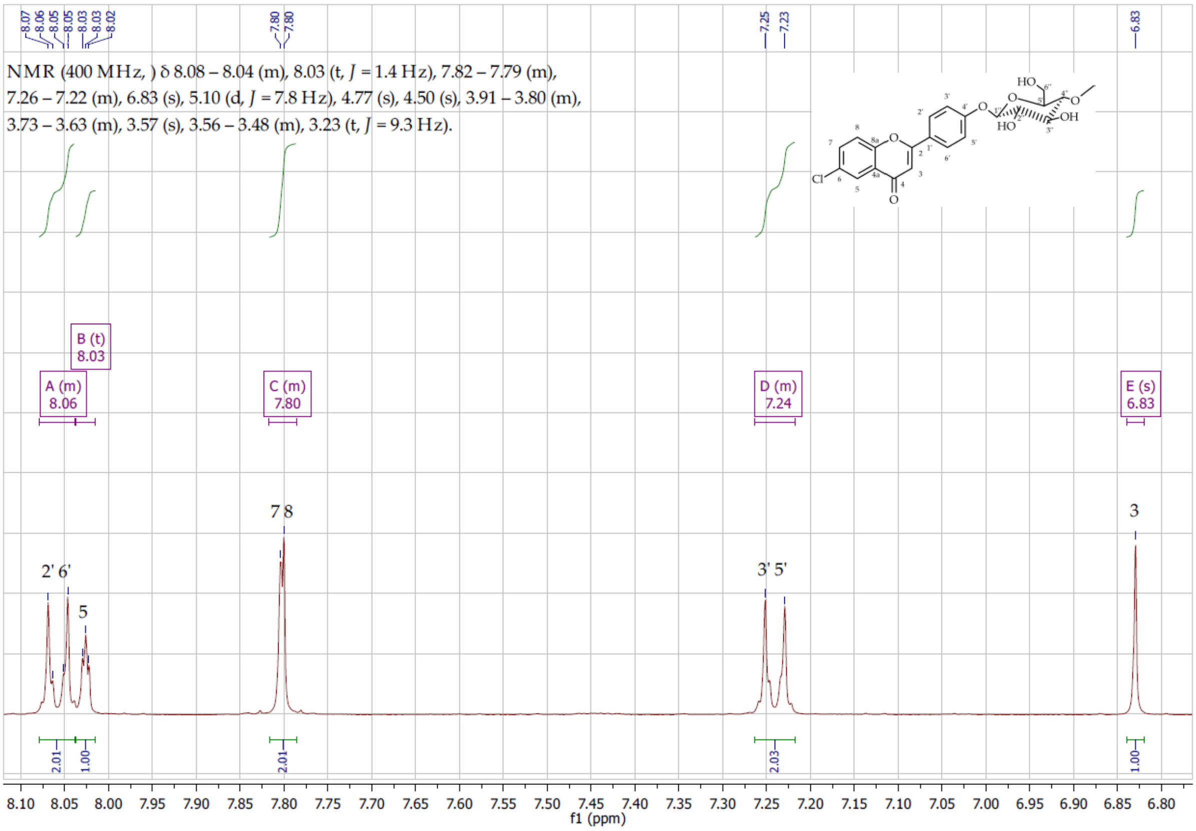

Figure S102. ¹H NMR spectrum expansion (*δ*, acetone-d6, 600 MHz) of 6-chloroflavone 3′-*O*-*β*-D-(4′′-*O*-methyl)-glucopyranoside (**4a**).

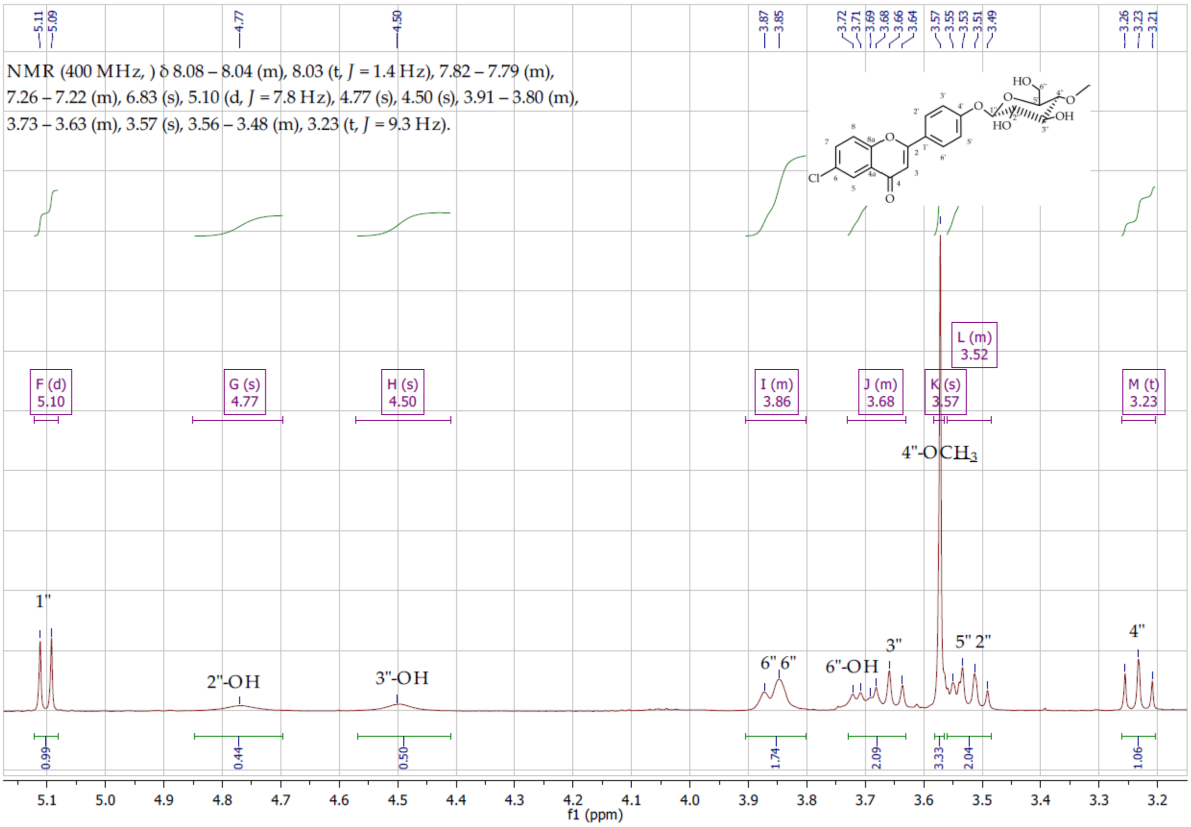

Figure S103. ¹H NMR spectrum expansion (*δ*, acetone-d6, 600 MHz) of 6-chloroflavone 3′-*O*-*β*-D-(4′′-*O*-methyl)-glucopyranoside (**4a**).

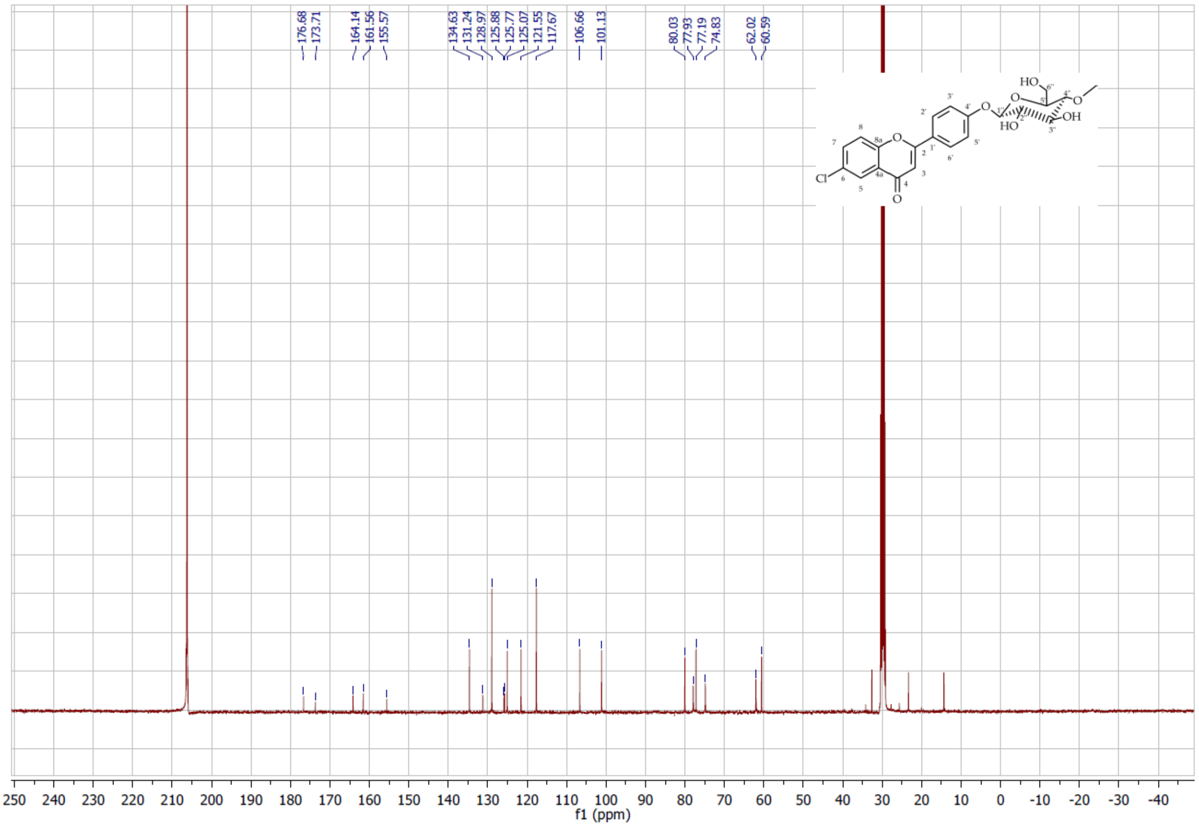

Figure S104. ^{13}C NMR spectrum (*δ*, acetone-d6, 151 MHz) of 6-chloroflavone 3′-*O*-*β*-D-(4′′-*O*-methyl)-glucopyranoside (**4a**).

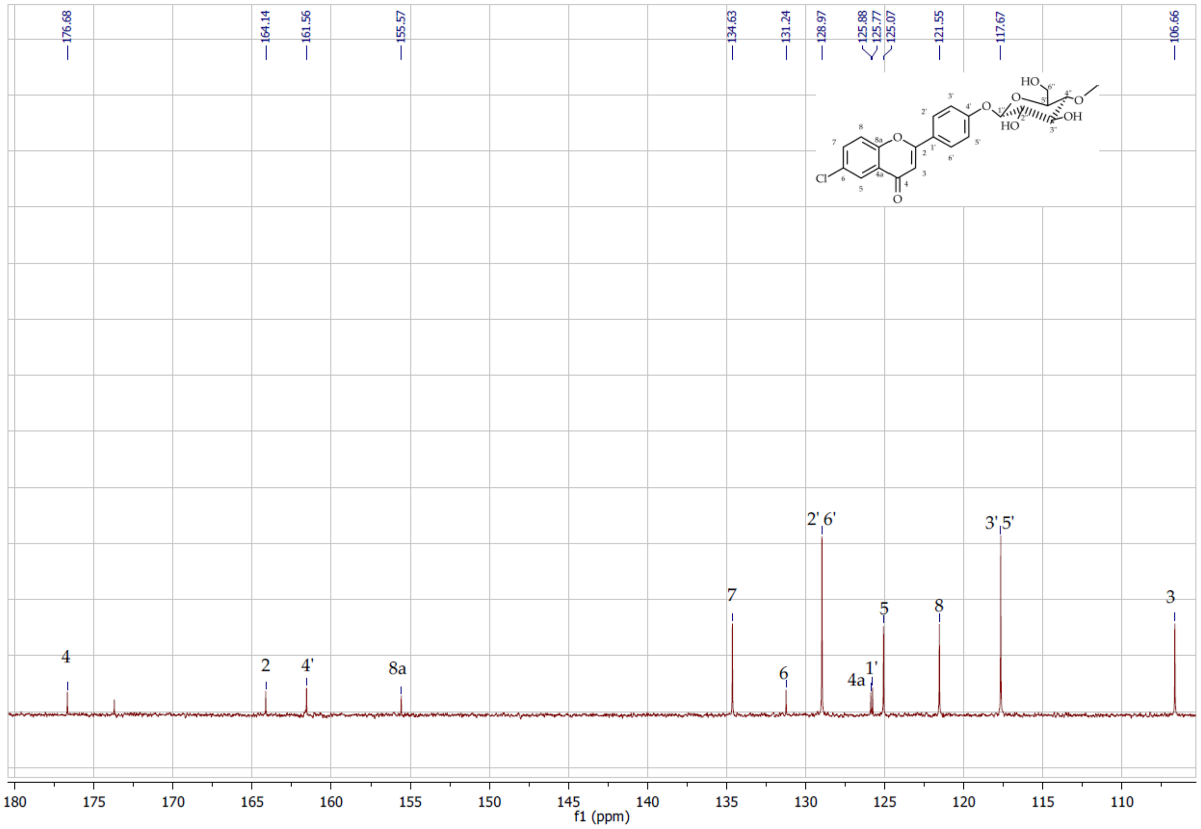

Figure S105. ^{13}C NMR spectrum expansion (*δ*, acetone-d6, 151 MHz) of 6-chloroflavone 3′-*O*-*β*-D-(4′′-*O*-methyl)-glucopyranoside (**4a**).

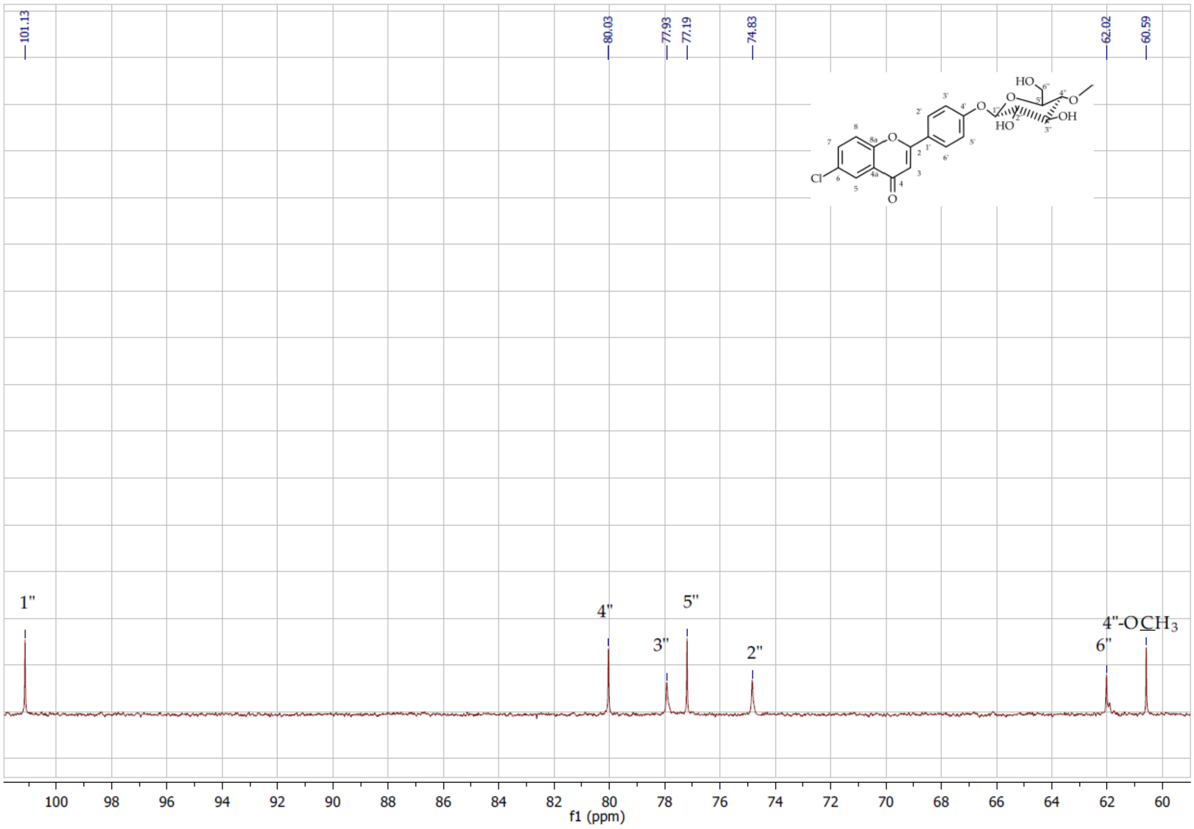

Figure S106. ^{13}C NMR spectrum expansion (*δ*, acetone-d6, 151 MHz) of 6-chloroflavone 3′-*O*-*β*-D-(4′′-*O*-methyl)-glucopyranoside (**4a**).

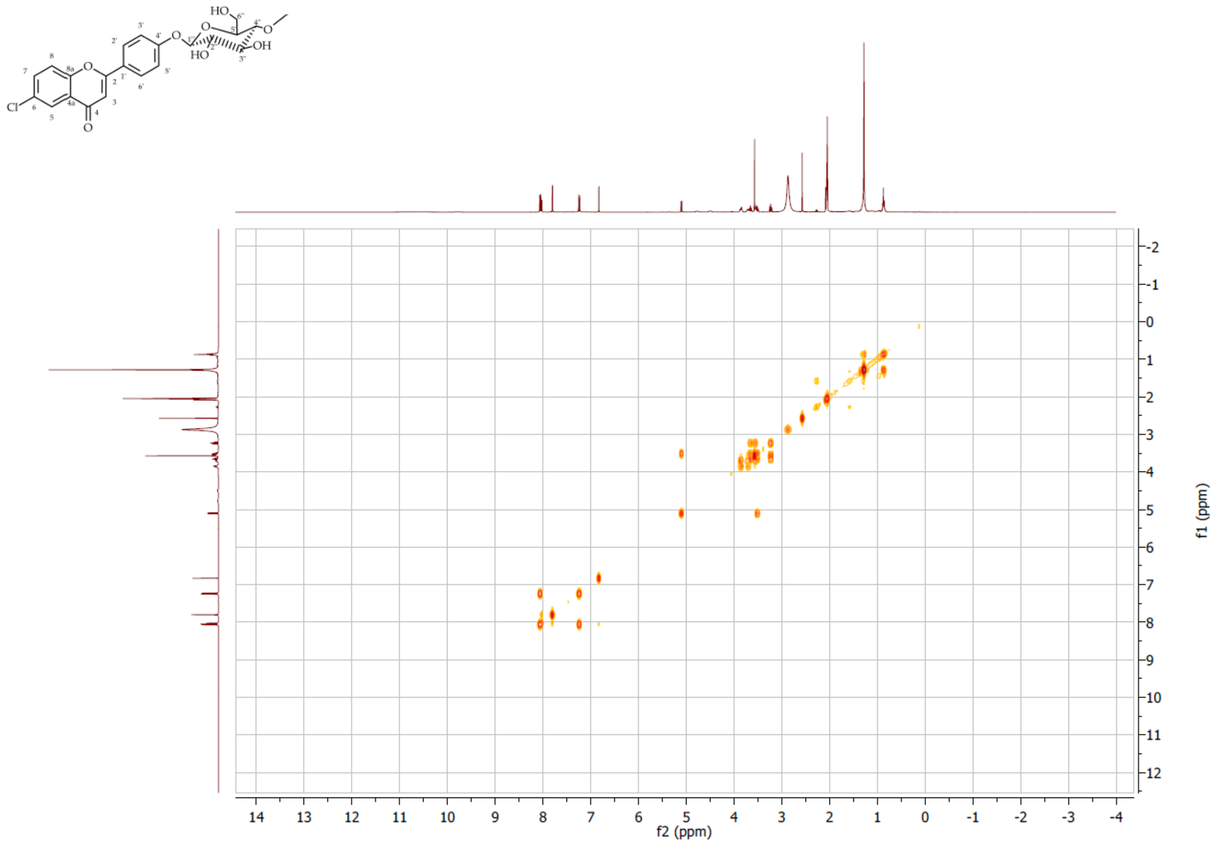

Figure S107. COSY contour map – ^{1}H x ^{1}H of 6-chloroflavone 3′-*O*-*β*-D-(4′′-*O*-methyl)-glucopyranoside (**4a**).

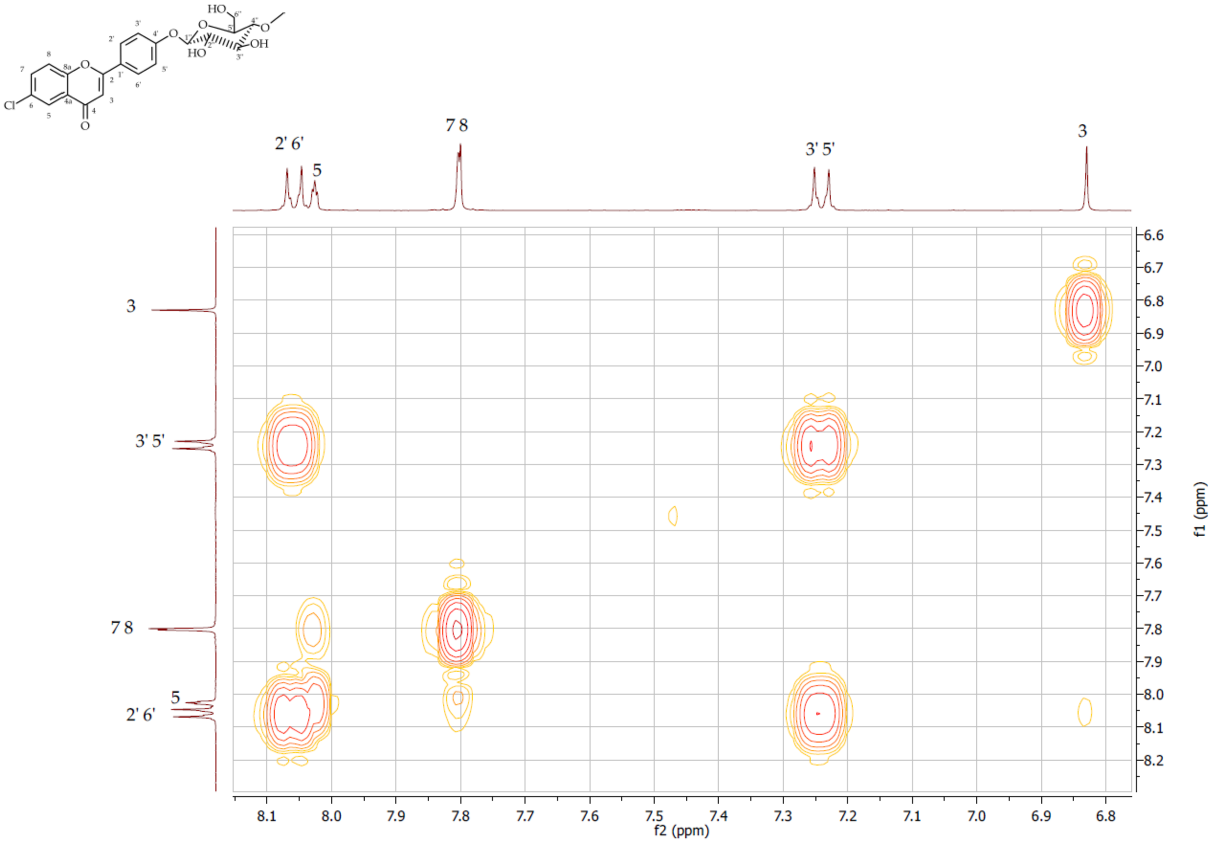

Figure S108. COSY contour map – ^{1}H x ^{1}H expansion of 6-chloroflavone 3′-*O*-*β*-D-(4′′-*O*-methyl)-glucopyranoside (**4a**).

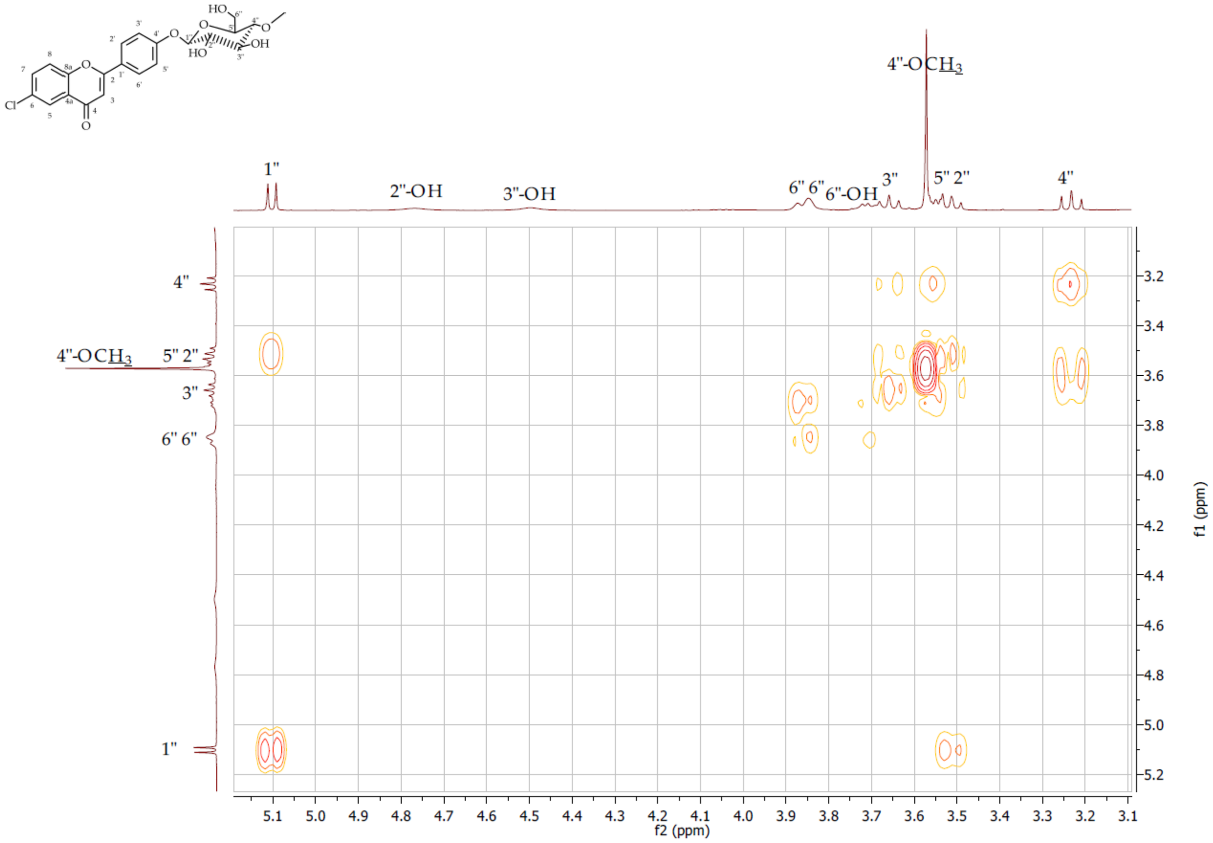

Figure S109. COSY contour map – ^{1}H x ^{1}H expansion of 6-chloroflavone 3′-*O*-*β*-D-(4′′-*O*-methyl)-glucopyranoside (**4a**).

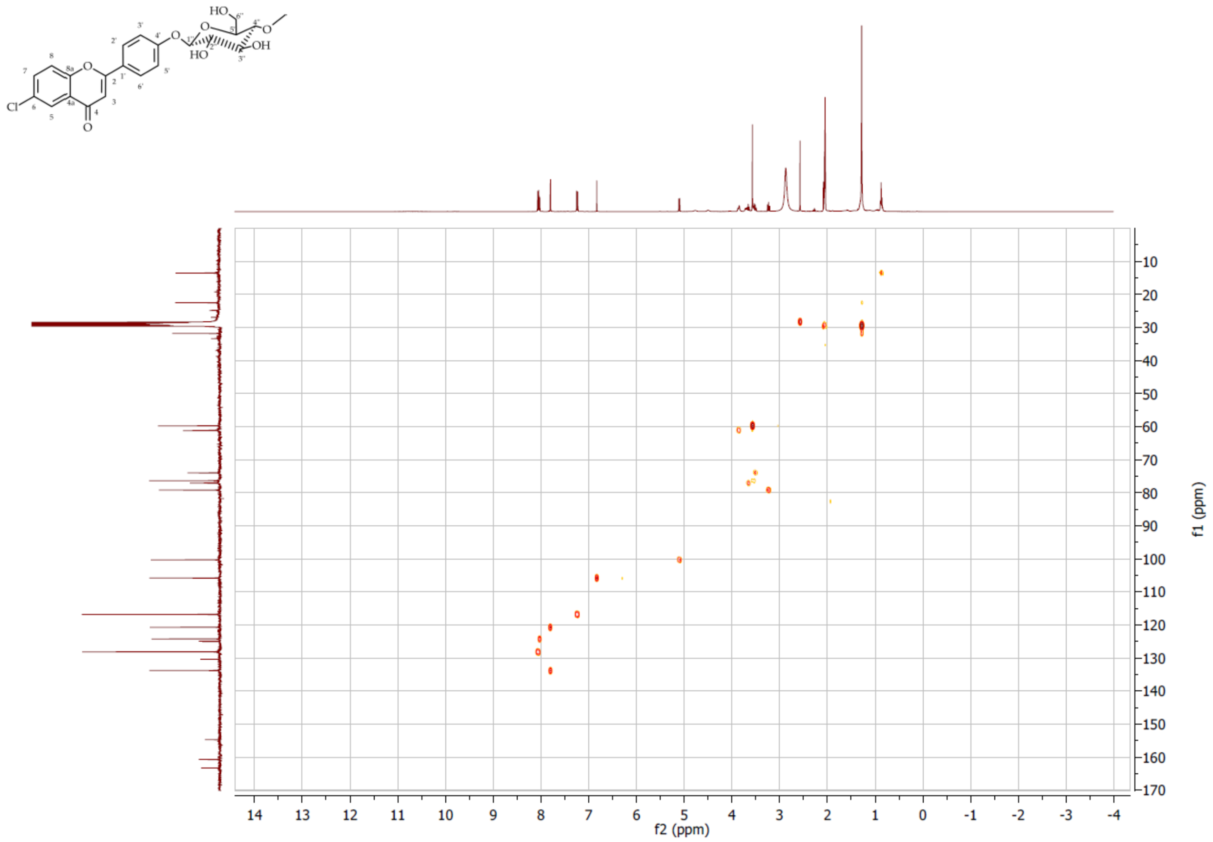

Figure S110. HMQC contour map – ^{1}H x ^{13}C of 6-chloroflavone 3′-*O*-*β*-D-(4′′-*O*-methyl)-glucopyranoside (**4a**).

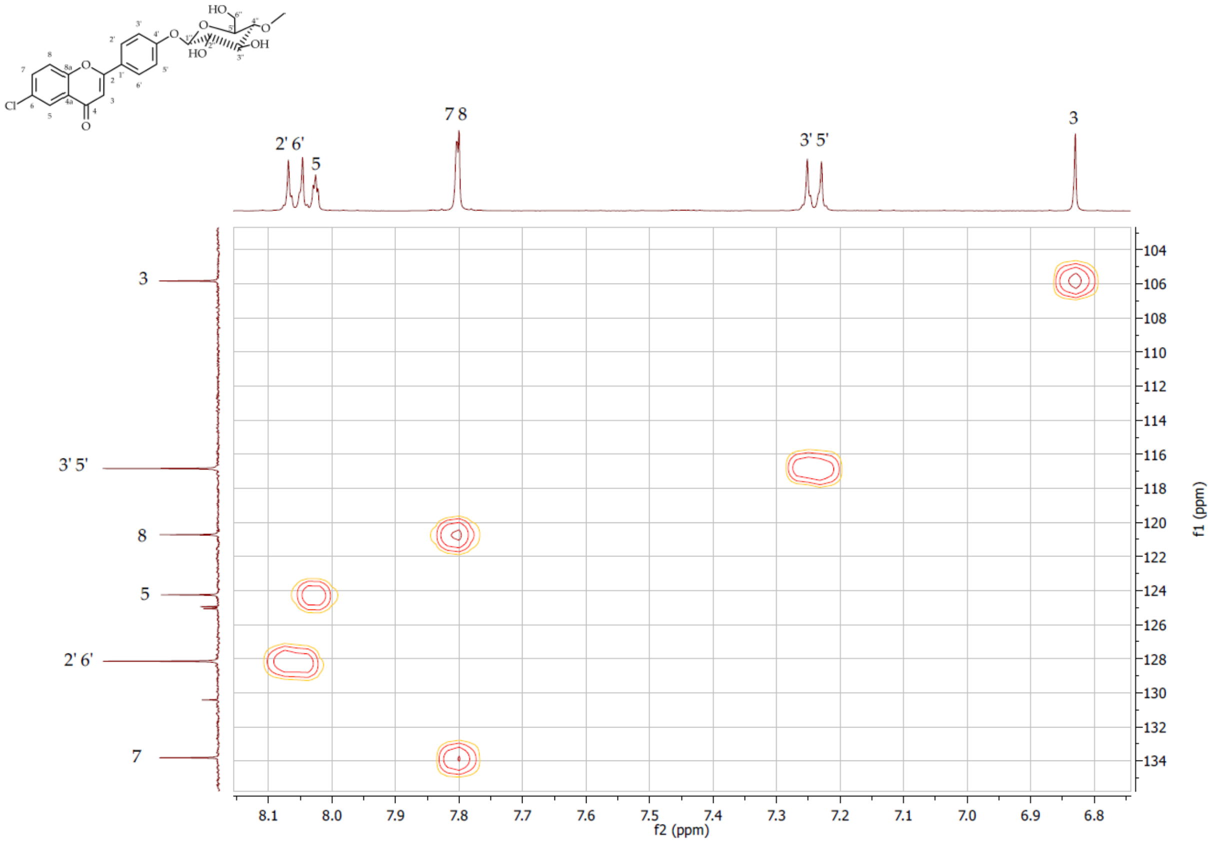

Figure S111. HMQC contour map – ^{1}H x ^{13}C expansion of 6-chloroflavone 3′-*O*-*β*-D-(4′′-*O*-methyl)-glucopyranoside (**4a**).

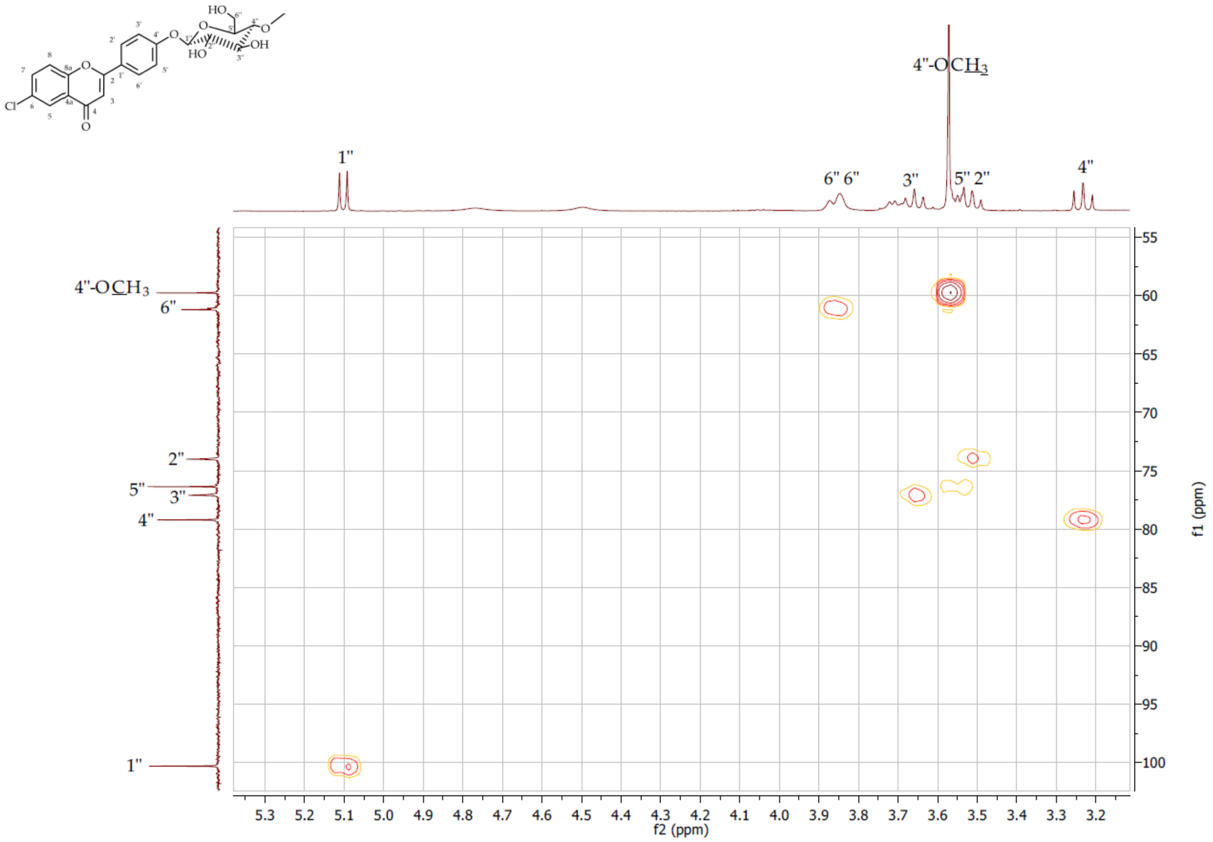

Figure S112. HMQC contour map – ^{1}H x ^{13}C expansion of 6-chloroflavone 3′-*O*-*β*-D-(4′′-*O*-methyl)-glucopyranoside (**4a**).

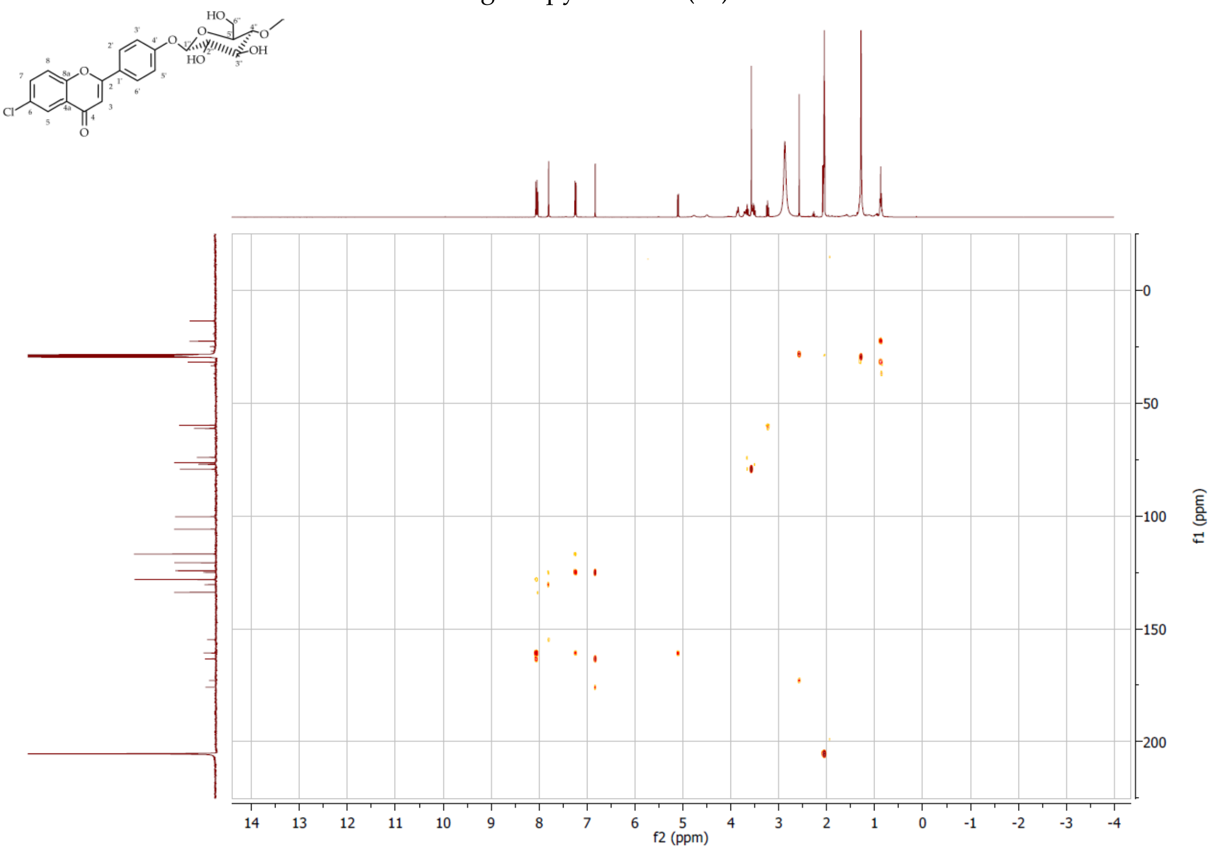

Figure S113. HMBC contour map – ^{1}H x ^{13}C of 6-chloroflavone 3′-*O*-*β*-D-(4′′-*O*-methyl)-glucopyranoside (**4a**).

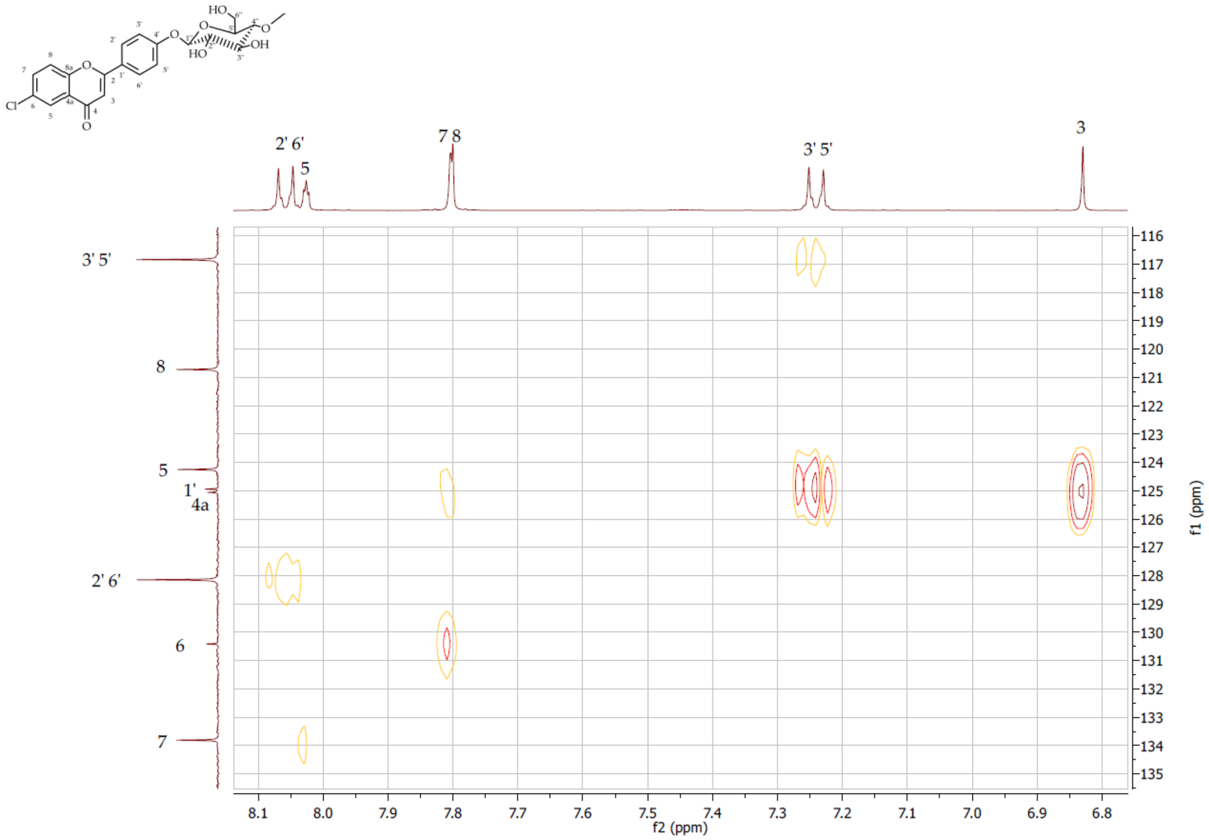

Figure S114. HMBC contour map – ^{1}H x ^{13}C expansion of 6-chloroflavone 3′-*O*-*β*-D-(4′′-*O*-methyl)-glucopyranoside (**4a**).

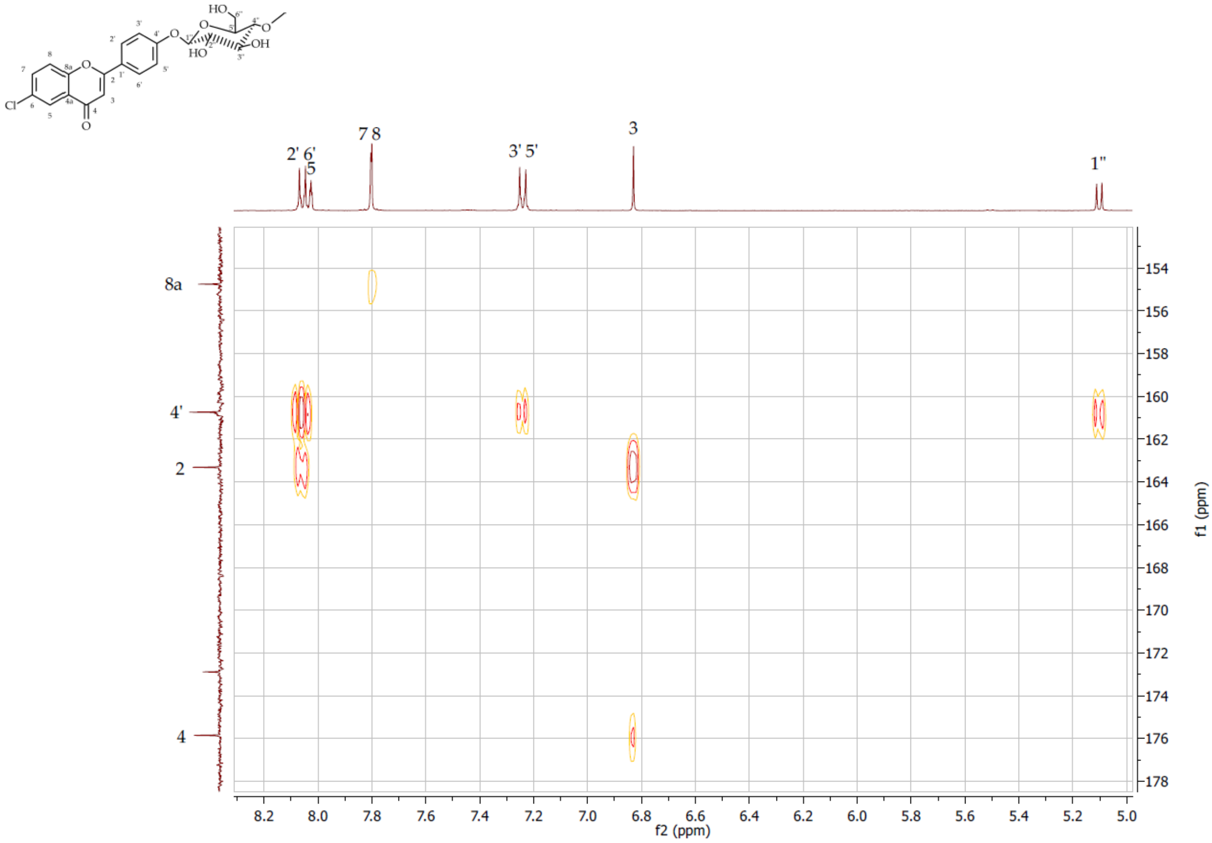

Figure S115. HMBC contour map – ^{1}H x ^{13}C expansion of 6-chloroflavone 3′-*O*-*β*-D-(4′′-*O*-methyl)-glucopyranoside (**4a**).

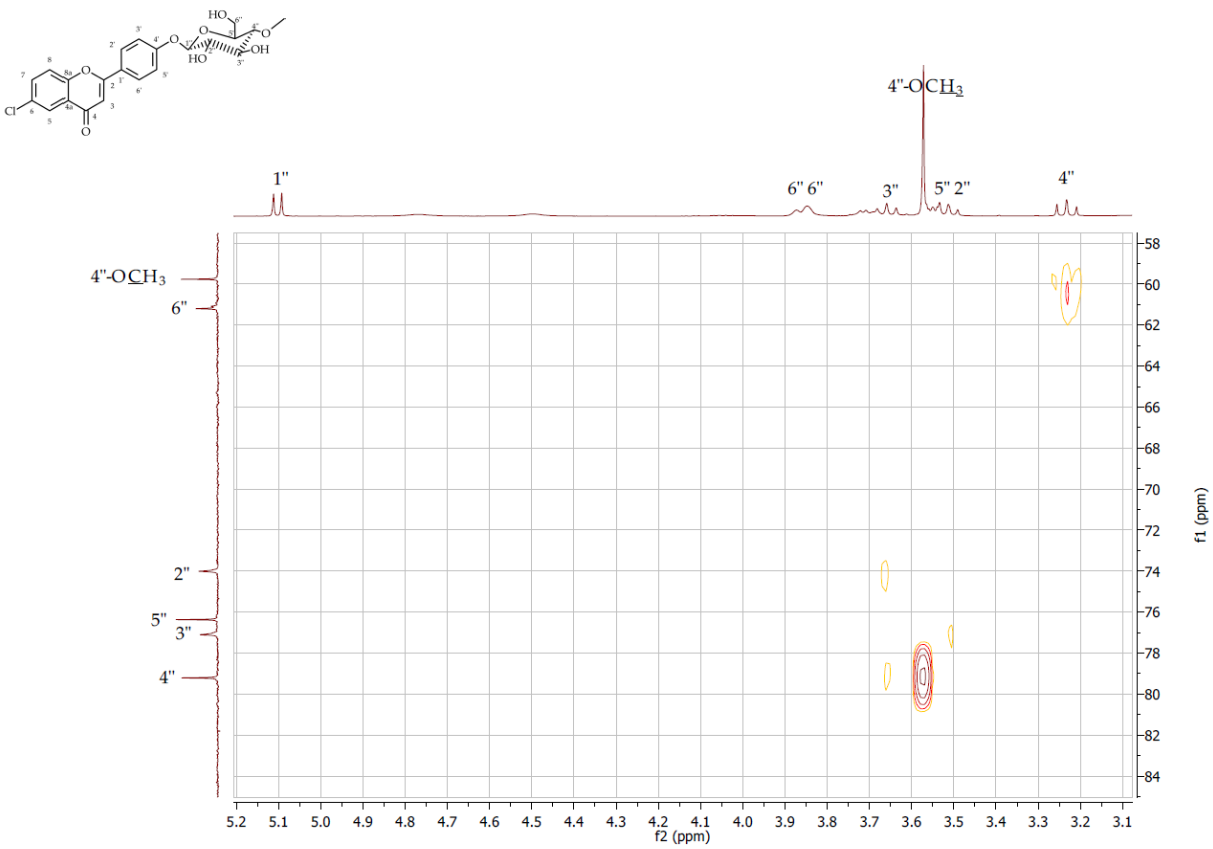

Figure S116. HMBC contour map – ^1H x ^{13}C expansion of 6-chloroflavone 3′-*O*-*β*-D-(4′′-*O*-methyl)-glucopyranoside (**4a**).

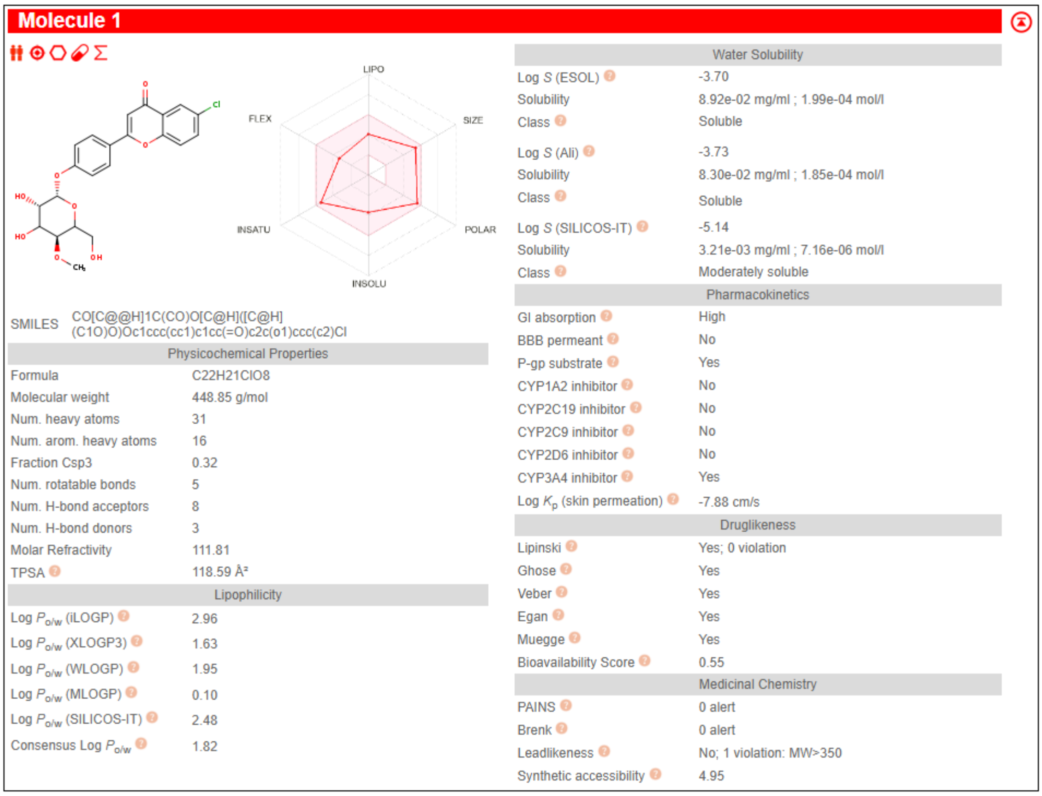

SMILES CO[C@@H]1C(CO)O[C@H]([C@H](C1O)O)Oc1ccc(cc1)c1cc(=O)c2c(o1)ccc(c2)Cl

Physicochemical Properties	
Formula	C22H21ClO8
Molecular weight	448.85 g/mol
Num. heavy atoms	31
Num. arom. heavy atoms	16
Fraction Csp3	0.32
Num. rotatable bonds	5
Num. H-bond acceptors	8
Num. H-bond donors	3
Molar Refractivity	111.81
TPSA	118.59 Å²

Lipophilicity	
Log $P_{o/w}$ (iLOGP)	2.96
Log $P_{o/w}$ (XLOGP3)	1.63
Log $P_{o/w}$ (WLOGP)	1.95
Log $P_{o/w}$ (MLOGP)	0.10
Log $P_{o/w}$ (SILICOS-IT)	2.48
Consensus Log $P_{o/w}$	1.82

Water Solubility	
Log *S* (ESOL)	-3.70
Solubility	8.92e-02 mg/ml ; 1.99e-04 mol/l
Class	Soluble
Log *S* (Ali)	-3.73
Solubility	8.30e-02 mg/ml ; 1.85e-04 mol/l
Class	Soluble
Log *S* (SILICOS-IT)	-5.14
Solubility	3.21e-03 mg/ml ; 7.16e-06 mol/l
Class	Moderately soluble

Pharmacokinetics	
GI absorption	High
BBB permeant	No
P-gp substrate	Yes
CYP1A2 inhibitor	No
CYP2C19 inhibitor	No
CYP2C9 inhibitor	No
CYP2D6 inhibitor	No
CYP3A4 inhibitor	Yes
Log K_p (skin permeation)	-7.88 cm/s

Druglikeness	
Lipinski	Yes; 0 violation
Ghose	Yes
Veber	Yes
Egan	Yes
Muegge	Yes
Bioavailability Score	0.55

Medicinal Chemistry	
PAINS	0 alert
Brenk	0 alert
Leadlikeness	No; 1 violation: MW>350
Synthetic accessibility	4.95

Figure S117. 6-Chloroflavone 3′-*O*-*β*-D-(4′′-*O*-methyl)-glucopyranoside (**4a**) physicochemical and ADME parameters prediction using the SwissADME modelling.

Molecule 1

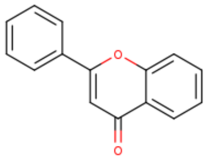

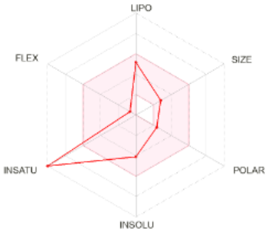

SMILES O=c1cc(oc2c1cccc2)c1ccccc1

Physicochemical Properties	
Formula	C15H10O2
Molecular weight	222.24 g/mol
Num. heavy atoms	17
Num. arom. heavy atoms	16
Fraction Csp3	0.00
Num. rotatable bonds	1
Num. H-bond acceptors	2
Num. H-bond donors	0
Molar Refractivity	67.92
TPSA	30.21 Å²

Lipophilicity	
Log $P_{o/w}$ (iLOGP)	2.55
Log $P_{o/w}$ (XLOGP3)	3.56
Log $P_{o/w}$ (WLOGP)	3.46
Log $P_{o/w}$ (MLOGP)	2.27
Log $P_{o/w}$ (SILICOS-IT)	4.04
Consensus Log $P_{o/w}$	3.18

Water Solubility	
Log *S* (ESOL)	-4.09
Solubility	1.80e-02 mg/ml ; 8.11e-05 mol/l
Class	Moderately soluble
Log *S* (Ali)	-3.88
Solubility	2.93e-02 mg/ml ; 1.32e-04 mol/l
Class	Soluble
Log *S* (SILICOS-IT)	-6.13
Solubility	1.63e-04 mg/ml ; 7.33e-07 mol/l
Class	Poorly soluble

Pharmacokinetics	
GI absorption	High
BBB permeant	Yes
P-gp substrate	No
CYP1A2 inhibitor	Yes
CYP2C19 inhibitor	Yes
CYP2C9 inhibitor	No
CYP2D6 inhibitor	No
CYP3A4 inhibitor	No
Log K_p (skin permeation)	-5.13 cm/s

Druglikeness	
Lipinski	Yes; 0 violation
Ghose	Yes
Veber	Yes
Egan	Yes
Muegge	Yes
Bioavailability Score	0.55

Medicinal Chemistry	
PAINS	0 alert
Brenk	0 alert
Leadlikeness	No; 2 violations: MW<250, XLOGP3>3.5
Synthetic accessibility	2.88

Figure S118. Flavone (**5**) physicochemical and ADME parameters prediction using the SwissADME modelling.
